# Supplementary material for: Development of bifunctional organocatalysts and application to asymmetric total synthesis of naucleofficine I and II
Source: Nat Commun. 2019 Jul 29;10:3394. doi: 10.1038/s41467-019-11382-8 (PMC6662887; doi:10.1038/s41467-019-11382-8)
Supplement: Supplementary file 1 — Supplementary Information [file 41467_2019_11382_MOESM1_ESM.pdf]

Supplementary Information

Development of Bifunctional Organocatalysts and Application to Asymmetric Total Synthesis of Naucleofficine I and II

Yuan et al.

Supplementary Note 1

General information

All reactions requiring anhydrous conditions were carried out under argon atmosphere using oven-dried glassware (130 °C), which was cooled under argon. All solvents were purified and dried by standard techniques, and distilled prior to use. All reactions under standard conditions were monitored by thin-layer chromatography (TLC) on gel F_{254} plates. The products were purified by flash column chromatography on silica gel (200~300 mesh) or neutral alumina (200~300 mesh).

^{1}H NMR, **^{13}C NMR** and **^{19}F NMR** spectra were obtained on Bruker AM-400, JEOL JNM-ECS-400, or Varian Mercury-600.

Chemical shifts (δ) were reported in ppm relative to residual solvent signals ($CDCl_3$: 7.26 ppm for ^{1}H NMR, 77.0 ppm for ^{13}C NMR; DMSO-d_6: 2.50 ppm for ^{1}H NMR, 39.5 ppm for ^{13}C NMR; CD_3OD: 3.31 ppm for ^{1}H NMR, 49.0 ppm for ^{13}C NMR). The following abbreviations were used to indicate the multiplicity in NMR spectra: s, singlet; d, doublet; t, triplet; m, multiplet.

High-resolution mass spectral analysis (**HRMS**) data were measured on the Bruker ApexII with ESI resource.

Infrared (**IR**) spectra were recorded on Nicolet FT-170SX spectrometer.

Melting points were measured on a melting point apparatus and were uncorrected.

The enantiomeric excesses (**ee**) of the products were determined by high performance liquid chromatography (**HPLC**) analysis.

X-ray diffraction data were collected on Agilent SuperNova Eos diffractometer.

Optical rotations were detected on RUDOLPH A21202-J APTV/GW.

Supplementary Note 2

General Synthesis of Cat 1-4:

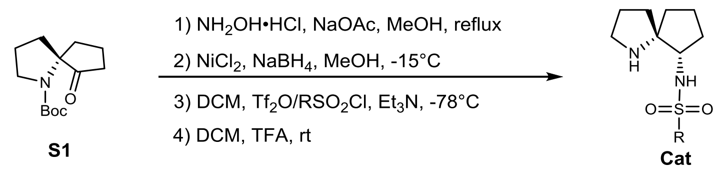

(*5S,6S*)-1-aza-6- trifluoromethanesulfonamidyl- spiro[4.4]nonane (Cat 1):

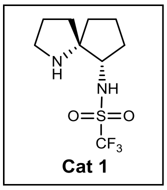

To the stirred solution of (*S*)-**S1**[1] (4.37 g, 18.2 mmol) in MeOH (80 mL) were added NaOAc (4.00 g, 72.8 mmol) and $NH_2OH{\cdot}HCl$ (3.78 g, 54.6 mmol) at room temperature and the mixture was heated to reflux for 4 hours. The resulting solution was concentrated in vacuo and sat. $NaHCO_3$ (100 mL) was added, and then the mixture was extracted with EtOAc (3×80 mL). The combined organic phase was washed with brine, dried over Na_2SO_4 and concentrated in vacuo. The crude product oxime was used to the next step directly without purification.

To a stirred solution of crude oxime product and anhydrous $NiCl_2$ (4.753 g, 36.4 mmol) in anhydrous MeOH (190 mL) was added $NaBH_4$ (10.44 g, 182 mmol) portionwise at -15 °C over a period of 2 hours. After complete addition, the resulting black slurry was stirred at this temperature for 12 hours. Then, the reaction mixture was warmed to 0 °C, and the mixture was then quenched with water. The resulting solution was concentrated in vacuo and the mixture was extracted with DCM (3×80 mL), and the combined organic phase was washed with brine, dried over anhydrous Na_2SO_4, and concentrated in vacuo for the next step directly.

Tf_2O (3.1 mL, 18.2 mmol) was added dropwise to a stirred solution of the crude Boc-SPD-amine product and Et_3N (6.4 mL, 54.6 mmol) in anhydrous DCM (100 mL) at -78 °C. The reaction mixture was stirred 30 minutes, and the mixture was quenched by the portion-wise addition of saturated $NaHCO_3$ aqueous solution. The mixture was extracted with EtOAc (3×100 mL). The combined organic phase was washed with brine, dried over Na_2SO_4 and concentrated in vacuo.

The residue was dissolved in DCM (100 mL), and TFA (trifluoroacetic acid) (9.5 mL, 127 mmol) was added at 0 ℃. About 1.5 hours later, the system was concentrated in vacuo to remove most of TFA, and then NaOH aqueous solution was added to ensure that the PH of the mixture was 10-12. Then the mixture was extracted with EtOAc (8×100 mL). The combined organic phase was dried over Na_2SO_4

and concentrated in vacuo. The crude residue was purified through column chromatography on silica gel (DCM: MeOH = 10: 1) gel to give **Cat 1** (3.387 g, 68% yield for 4 steps) as a white solid. **Cat 1** was purified by further crystallization (in MeOH and Et_2O). M.p. 158-160 ℃; $[\alpha]_D^{24}$ -20 (c 0.5, MeOH); **IR** (neat) ν 3349.8, 2970.9, 2465.5, 1629.4, 1447.2, 1198.6, 1049.4, 881.5, 607.2 cm^{-1}; **^{1}H NMR** (400 MHz, CD_3OD) δ 3.54 (t, *J* = 7.3 Hz, 1H), 3.34-3.16 (m, 2H), 2.07-1.87 (m, 5H), 1.86-1.65 (m, 3H), 1.65-1.40 (m, 2H); **^{13}C NMR** (100 MHz, CD_3OD) δ 127.9, 124.7, 121.5, 118.2, 76.5, 62.8, 45.8, 35.4, 35.1, 34.2, 24.1, 21.4; **^{19}F NMR** (375 MHz, CD_3OD) δ -78.93; **HRMS (ESI)** calcd for $[M+H]^+$ $C_9H_{16}F_3N_2O_2S$, m/z: 273.0885, found: 273.0882.

(*5S,6S*)-1-aza-6-(4′-toluene)sulfonamidyl-spiro[4.4]nonane (Cat 2):

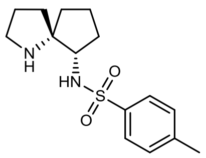

Cat 2

Cat 2 was prepared according to the same procedure as **Cat 1** from **S1** (67.1 mg) and TsCl (*p*-toluenesulfonyl chloride) as a colorless oil in 65% yield (54.2 mg). $[\alpha]_D^{21}$ -26 (c 0.5, MeOH); **IR** (neat) ν 2957.3, 2371.5, 1675.1, 1477.1, 1330.4, 1160.7, 1094.0, 720.5, 663.8, 569.6 cm^{-1}; **^{1}H NMR** (400 MHz, $CDCl_3$) δ 7.75 (d, *J* = 8.2 Hz, 2H), 3.01-2.81 (m, 2H), 2.55-2.49 (m, 1H), 2.42 (s, 3H), 1.87-1.73 (m, 1H), 1.73-1.56 (m, 5H), 1.55-1.40 (m, 4H); **^{13}C NMR** (100 MHz, $CDCl_3$) δ 142.8, 137.8, 129.4, 127.1, 70.6, 59.5, 46.3, 38.0, 37.3, 31.7, 26.2, 21.5, 20.9. **HRMS (ESI)** calcd for $[M+H]^+$ $C_{15}H_{23}N_2O_2S$, m/z: 295.1480, found: 295.1474.

(*5S,6S*)-1-aza-6-(4′-trifluoromethylphenyl)sulfonamidyl-spiro[4.4]nonane (Cat 3):

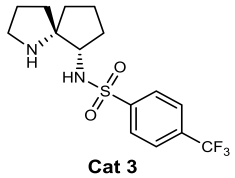

Cat 3

Cat 3 was prepared according to the same procedure as **Cat 1** from **S1** (67.1 mg) and *p*-(trifluoromethyl)benzenesulfonyl chloride as a colorless oil in 56% yield (55.0 mg). $[\alpha]_D^{26}$ -50 (c 0.5, MeOH); **IR** (neat) ν 2957.6, 1608.9, 1324.1, 1167.3, 1132.2, 1062.6, 712.4, 611.4 cm^{-1}; **^{1}H NMR** (400 MHz, $CDCl_3$) δ 8.01 (d, *J* = 8.2 Hz, 2H), 7.77 (d, *J* = 8.3 Hz, 2H), 3.06-2.87 (m, 2H), 2.53 (dt, *J* = 10.4, 6.5 Hz, 1H), 1.85-1.78 (m, 1H), 1.73-1.57 (m, 5H), 1.56-1.37 (m, 4H); **^{13}C NMR** (100 MHz, $CDCl_3$) δ 144.54, 134.09, 133.76, 127.55, 126.04, 126.00, 125.97, 125.93, 124.64, 121.93, 70.43, 59.72, 46.34,

38.16, 37.35, 31.75, 26.23, 20.96; ^{19}**F NMR** (564 MHz, $CDCl_3$) δ -63.07; **HRMS (ESI)** calcd for $[M+H]^+$ $C_{15}H_{20}F_3N_2O_2SNa$, m/z: 349.1192, found: 349.1185.

N-((5*S*,6*S*)-1-azaspiro[4.4]nonan-6-yl)methanesulfonamide (Cat 4) :

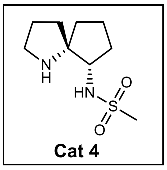

Cat 4 was prepared according to the same procedure as **Cat 1** from **S1** (69.7 mg) and methanesulfonyl chloride as a colorless oil in 66% yield (42.0 mg).

$[\alpha]_D^{20}$ 8 (c 0.5, MeOH); **IR** (neat) ν 2956.2, 1675.5, 1450.4, 1322.0, 1150.7, 981.2, 761.9, 520.6 cm^{-1}; ^{1}**H NMR** (600 MHz, CD_3OD) δ 3.53 (t, *J* = 6.8 Hz, 1H), 3.11 (dt, *J* = 10.7, 7.3 Hz, 1H), 3.07 – 2.97 (m, 4H), 2.18 (m, 1H), 1.95 – 1.87 (m, 3H), 1.84 – 1.63 (m, 6H); ^{13}**C NMR** (150 MHz, CD_3OD) δ 73.0, 60.9, 46.4, 40.6, 36.4, 36.3, 31.5, 25.4, 20.7; [1] **HRMS (ESI)** calcd for $[M+Na]^+$ $C_9H_{18}N_2O_2S$, m/z: 241.0987, found: 241.0989.

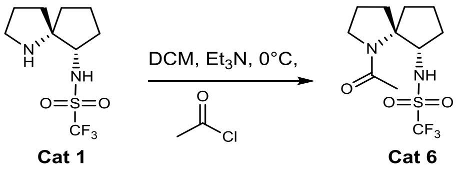

To the stirred solution of the **Cat 1** (97.0 mg, 0.36 mmol) in DCM (5 mL) were added Et_3N (99 μL, 0.71 mmol, 2.0 eq.) and acetyl chloride (38 μL, 0.53 mmol, 1.5 eq.) at 0 °C. Then the reaction stirred at this temperature until the stating materials disappeared via TLC detection. Next, 5 mL water was added with vigorous stirring. The resulting was extracted with DCM, and the combined organic phase was washed with brine, dried over anhydrous Na_2SO_4, and concentrated in vacuo. The residue was purified through column chromatography on silica gel (petroleum ether: EtOAc = 4: 1 to 1: 1) gel to give substrates **Cat 6** (114.2 mg, 86% yield).

$[\alpha]_D^{20}$ -44 (c 0.5, MeOH); **IR** (neat) ν 2973.1, 2877.4, 1628.8, 1422.1, 1183.4, 966.0, 606.3 cm^{-1}; ^{1}**H NMR** (400 MHz, $CDCl_3$) δ 7.11 (d, *J* = 9.3 Hz, 1H), 3.69 (dd, *J* = 16.6, 7.4 Hz, 1H), 3.63-3.50 (m, 2H), 2.63-2.50 (m, 1H), 2.11 (s, 3H), 2.08-1.79 (m, 7H), 1.56-1.32 (m, 2H); ^{13}**C NMR** (100 MHz, CD_3OD) δ 172.2, 124.4, 121.2, 118.0, 114.8, 72.1, 65.1, 50.2, 41.5, 35.4, 33.8, 24.2, 23.1, 22.1; ^{19}**F NMR** (375 MHz, $CDCl_3$) δ -78.23; **HRMS (ESI)** calcd for $[M+H]^+$ $C_{11}H_{18}F_3N_2O_3S$, m/z: 315.0990, found: 315.0983.

General Synthesis of Substrates:

All of the substrates in this article were prepared according to the literature or commercial available.

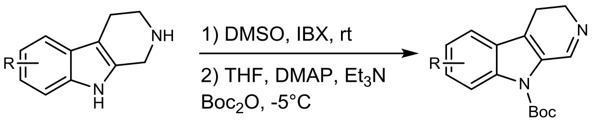

2a-2j

IBX (1.1 eq.) was added to DMSO at room temperature for 30 min, and substituted tetrahydro-beta-carboline (1.0 eq.) of commercially availabe was added to the system when the solution became clear. About 30 min later, $Na_2S_2O_3$ saturated solution was added to the system, and the mixture was extracted with EtOAc. Then the combined organic phase was washed with saturated brine six times, dried over Na_2SO_4 and concentrated in vacuo for the next step directly.

To the stirred solution of the crude product in THF were added DMAP (0.2 eq.), Et_3N (1.5 eq.) and Boc_2O (1.2 eq.) at -5 °C. Then the system stirred at this temperature until the stating materials disappeared via TLC detection (about 12 hours). Next, the reaction mixture was warmed to room temperature, and 10 mL water was added with vigorous stirring. The resulting was extracted with EtOAc, and the combined organic phase was washed with brine, dried over anhydrous Na_2SO_4, and concentrated in vacuo. The residue was purified through column chromatography on silica gel (petroleum ether: EtOAc = 5: 1 to 1: 1) gel to give substrates **2a-2j.**

tert-butyl 3,4-dihydro-9H-pyrido-[3,4-b]-indole-9-carboxylate (2a)

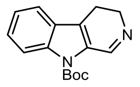

2a

Compound **2a** was obtained as a white solid (3.811 g, two steps for 52% yield). m.p. 83-84 ℃; **IR** (neat) ν 2955.6, 2924.1, 1731.5, 1375.7, 1144.7, 1025.4, 964.3, 836.9, 744.6 cm^{-1}; **^{1}H NMR** (400 MHz, $CDCl_3$) δ 8.89 (t, *J* = 2.1 Hz, 1H), 8.21 (d, *J* = 8.4 Hz, 1H), 7.55 (d, *J* = 7.8 Hz, 1H), 7.43-7.39 (m, 1H), 7.33-7.24 (m, 1H), 3.93-3.80 (m, 2H), 2.86-2.76 (m, 2H), 1.70 (s, 9H); **^{13}C NMR** (100 MHz, $CDCl_3$) δ 152.5, 149.9, 136.6, 128.7, 127.1, 126.9, 123.3, 123.2, 119.7, 116.2, 84.6, 47.0, 28.2, 18.7; **HRMS (ESI)** calcd for $[M+H]^+$ $C_{16}H_{19}N_2O_2$, m/z: 271.1447, found: 271.1443.

tert-butyl 6-methoxy-3,4-dihydro-9H-pyrido-[3,4-b]-indole-9-carboxylate (2b)

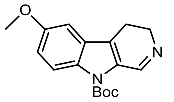

2b

Compound **2b** was obtained as a yellow oil (97.6 mg, two steps for 32% yield). **IR** (neat) ν 2925.4, 1729.0, 1611.4, 1457.6, 1380.8, 1262.0, 1138.2, 806.4, 739.1 cm^{-1}; **^{1}H NMR** (400 MHz, $CDCl_3$) δ 8.87 (t, *J* = 2.0 Hz, 1H), 8.09 (d, *J* = 9.1 Hz, 1H), 7.02 (dd, *J* = 9.1, 2.6 Hz, 1H), 6.94 (d, *J* = 2.5 Hz, 1H), 3.95-3.80 (m, 5H), 2.85-2.69 (m, 2H), 1.69 (s, 9H); **^{13}C NMR** (100 MHz, $CDCl_3$) δ 156.2, 152.5, 149.8,

131.3, 129.2, 127.8, 122.9, 117.0, 116.1, 101.5, 84.4, 55.6, 47.0, 28.2, 18.7; **HRMS (ESI)** calcd for $[M+H]^+$ $C_{17}H_{21}N_2O_3$, m/z: 301.1552, found: 301.1563.

tert-butyl 6-methyl-3,4-dihydro-9H-pyrido-[3,4-b]-indole-9-carboxylate (2c)

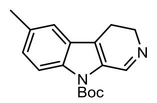

2c

Compound **2c** was obtained as a yellow oil (102.6 mg, two steps for 30% yield). **IR** (neat) ν 2977.4, 2925.5, 1729.5, 1456.7, 1372.0, 1139.4, 808.4, 736.9 cm^{-1}; **^{1}H NMR** (400 MHz, $CDCl_3$) δ 8.87 (s, 1H), 8.07 (d, *J* = 8.6 Hz, 1H), 7.32 (s, 1H), 7.23 (dd, *J* = 8.6, 1.3 Hz, 1H), 3.86 (td, *J* = 8.8, 2.2 Hz, 2H), 2.90-2.69 (m, 2H), 2.45 (s, 3H), 1.69 (s, 9H); **^{13}C NMR** (100 MHz, $CDCl_3$) δ 152.6, 149.9, 134.9, 132.8, 128.9, 128.4, 127.3, 123.2, 119.5, 115.8, 84.4, 47.0, 28.3, 21.3, 18.7; **HRMS (ESI)** calcd for $[M+H]^+$ $C_{17}H_{21}N_2O_2$, m/z: 285.1603 , found: 285.1606.

tert-butyl 6-fluoro-3,4-dihydro-9H-pyrido-[3,4-b]-indole-9-carboxylate (2d)

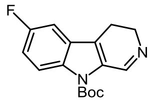

2d

Compound **2d** was obtained as an amorphous solid (102.6 mg, two steps for 31% yield). **IR** (neat) ν 2955.7, 2925.7, 1732.3, 1553.7, 1371.8, 1324.8, 1258.8, 1134.8, 738.4 cm^{-1}; **^{1}H NMR** (400 MHz, $CDCl_3$) δ 8.87 (s, 1H), 8.16 (dd, *J* = 9.1, 4.5 Hz, 1H), 7.21-7.01 (m, 2H), 3.88 (td, *J* = 8.9, 2.2 Hz, 2H), 2.89-2.61 (m, 2H), 1.70 (s, 9H); **^{13}C NMR** (100 MHz, $CDCl_3$) δ 160.4, 158.0, 152.1, 149.5, 132.8, 129.8, 127.9, 127.8, 122.5, 122.5, 117.3, 117.2, 114.8, 114.5, 105.0, 104.7, 84.8, 46.9, 28.1, 18.4; **^{19}F NMR** (375 MHz, $CDCl_3$) δ -119.37; **HRMS (ESI)** calcd for [M+H]+ $C_{16}H_{18}FN_2O_2$, m/z: 289.1352 , found: 289.1344.

tert-butyl 6-chloro-3,4-dihydro-9H-pyrido[3,4-b]indole-9-carboxylate (2e)

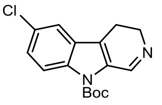

2e

Compound **2e** was obtained as an amorphous solid (261.2 mg, two steps for 51% yield). **IR** (neat) ν 2977.5, 2928.8, 1735.1, 1488.2, 1372.4, 1259.9, 1145.9, 809.1, 764.2 cm^{-1}; **^{1}H NMR** (400 MHz, $CDCl_3$) δ 8.87 (t, *J* = 2.1 Hz, 1H), 8.14 (d, *J* = 8.9 Hz, 1H), 7.50 (d, *J* = 2.0 Hz, 1H), 7.35 (dd, *J* = 8.9, 2.1 Hz, 1H), 3.88 (td, *J* = 8.8, 2.3 Hz, 2H), 2.84-2.72 (m, 2H), 1.70 (s, 9H); **^{13}C NMR** (100 MHz, $CDCl_3$) δ 152.1, 149.5, 134.9, 129.6, 129.0, 128.3, 127.0, 122.2, 119.2, 117.3, 85.1, 46.9, 28.2, 18.5; **HRMS (ESI)** calcd for $[M+H]^+$ $C_{16}H_{18}ClN_2O_2$, m/z: 305.1057, found: 305.1044.

tert-butyl 6-bromo-3,4-dihydro-9H-pyrido-[3,4-b]-indole-9-carboxylate (2f)

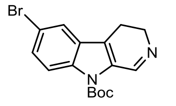

2f

Compound **2f** was obtained as an amorphous solid (102.3 mg, two steps for 42% yield). **IR** (neat) ν 2977.3, 1734.1, 1393.6, 1371.3, 1320.6, 807.3, 738.8, 667.8 cm^{-1}; **^{1}H NMR** (400 MHz, $CDCl_3$) δ 8.87 (s, 1H), 8.09 (d, *J* = 8.9 Hz, 1H), 7.67 (d, *J* = 1.8 Hz, 1H), 7.49 (dd, *J* = 8.9, 1.9 Hz, 1H), 3.88 (td, *J* = 8.9, 2.1 Hz, 2H), 2.82-2.73 (m, 2H), 1.70 (s, 9H); **^{13}C NMR** (100 MHz, $CDCl_3$) δ 152.2, 149.5, 135.3, 129.7, 129.4, 128. 8, 122.4, 122.2, 117.7, 116.6, 85.2, 46.9, 28.2, 18.5; **HRMS (ESI)** calcd for $[M+H]^+$ $C_{16}H_{18}BrN_2O_2$, m/z: 349.0552 , found: 349.0545.

tert-butyl 5-bromo-3,4-dihydro-9H-pyrido-[3,4-b]-indole-9-carboxylate (2g)

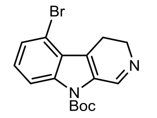

2g

Compound **2g** was obtained as an amorphous solid (550.8 mg, two steps for 47% yield). **IR** (neat) ν 2929.8, 1734.6, 1546.0, 1407.3, 1257.7, 1137.5, 847.6, 738.7 cm^{-1}; **^{1}H NMR** (400 MHz, $CDCl_3$) δ 8.85 (s, 1H), 8.22 (d, *J* = 8.4 Hz, 1H), 7.41 (d, *J* = 7.7 Hz, 1H), 7.25-7.18 (m, 1H), 3.84 (dd, *J* = 8.9, 1.9 Hz, 2H), 3.21 (t, *J* = 8.7 Hz, 2H), 1.70 (s, 9H); **^{13}C NMR** (100 MHz, $CDCl_3$) δ 152.1, 149.4, 137.8, 129.5, 127.4, 127.3, 126.0, 123.6, 115.4, 115.2, 85.3, 47.1, 28.2, 20.3; **HRMS (ESI)** calcd for $[M+H]^+$ $C_{16}H_{18}BrN_2O_2$, m/z: 349.0546, found: 349.0538.

tert-butyl 7-fluoro-3,4-dihydro-9H-pyrido-[3,4-b]-indole-9-carboxylate (2h)

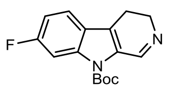

2h

Compound **2h** was obtained as an amorphous solid (174.1 mg, two steps for 45% yield). **IR** (neat) ν 2925.2, 1734.9, 1314.1, 1379.1, 1155.3, 1136.3, 859.8, 738.6 cm^{-1}; **^{1}H NMR** (400 MHz, $CDCl_3$) δ 8.85 (s, 1H), 7.94 (d, *J* = 10.5 Hz, 1H), 7.47 (dd, *J* = 8.6, 5.5 Hz, 1H), 7.04 (td, *J* = 8.9, 2.2 Hz, 1H), 3.87 (td, *J* = 9.0, 2.1 Hz, 2H), 2.79 (t, *J* = 8.7 Hz, 2H), 1.70 (s, 9H); **^{13}C NMR** (100 MHz, $CDCl_3$) δ 163.5, 161.1, 152.2, 149.6, 137.0, 136.9, 129.2, 123.5, 123.1, 120.6, 120.5, 112.0, 111.7, 103.8, 103.5, 85.1, 46.9, 28.2, 18.7; **^{19}F NMR** (375 MHz, $CDCl_3$) δ -112.76; **HRMS (ESI)** calcd for $[M+H]^+$ $C_{16}H_{18}FN_2O_2$, m/z: 289.1352 , found: 289.1338.

tert-butyl 8-bromo-3,4-dihydro-9H-pyrido-[3,4-b]-indole-9-carboxylate (2i)

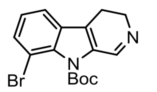

2i

Compound **2i** was obtained as an amorphous solid (1.801 g, two steps for 62% yield). **IR** (neat) ν 2923.1, 1753.1, 1659.2, 1467.3, 1264.4, 1151.7, 739.2 cm^{-1}; **^{1}H NMR** (400 MHz, $CDCl_3$) δ 8.73 (s, 1H), 7.60 (d, *J* = 7.7 Hz, 1H), 7.50 (d, *J* = 7.8 Hz, 1H), 7.15 (t, *J* = 7.8 Hz, 1H), 3.97-3.86 (m, 2H), 2.84-2.77 (m, 2H), 1.68 (s, 9H); **^{13}C NMR** (100 MHz, $CDCl_3$) δ 151.4, 148.2, 135.3, 131.7, 130.7, 130.1, 124.4, 121.7, 119.0, 109.0, 85.4, 47.3, 28.0, 18.6; **HRMS (ESI)** calcd for $[M+H]^+$ $C_{16}H_{18}BrN_2O_2$, m/z: 349.0552, found: 349.0546.

tert-butyl 8-methyl-3,4-dihydro-9H-pyrido-[3,4-b]-indole-9-carboxylate (2j)

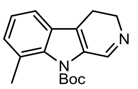

2j

Compound **2j** was obtained as an amorphous solid (178 mg, two steps for 38% yield). **IR** (neat) ν 2922.5, 1743.2, 1467.1, 1314.7, 1150.5, 967.2, 740.4 cm^{-1}; **^{1}H NMR** (400 MHz, $CDCl_3$) δ 8.73 (t, *J* = 2.1 Hz, 1H), 7.39 (t, *J* = 4.5 Hz, 1H), 7.22 (d, *J* = 4.9 Hz, 2H), 3.88 (td, *J* = 9.0, 2.2 Hz, 2H), 2.87-2.75 (m, 2H), 2.53 (s, 3H), 1.67 (s, 9H); **^{13}C NMR** (100 MHz, $CDCl_3$) δ 152.1, 149.4, 136.4, 129.9, 129.4, 128.3, 126.2, 123.6, 123.1, 117.4, 84.5, 47.1, 28.0, 21.4, 18.7; **HRMS (ESI)** calcd for $[M+H]^+$ $C_{17}H_{21}N_2O_2$, m/z: 285.1603, found: 285.1592.

9-tosyl-4,9-dihydro-3H-pyrido-[3,4-b]-indole (2k)

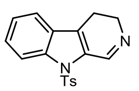

2k

The compound **2k** was prepared using the same procedure as reference.[2] **^{1}H NMR** (400 MHz, $CDCl_3$) δ 9.00 (t, *J* = 2.2 Hz, 1H), 8.18 (d, *J* = 8.5 Hz, 1H), 7.66 (d, *J* = 8.3 Hz, 2H), 7.50-7.37 (m, 2H), 7.29 (t, *J* = 8.0 Hz, 1H), 7.17 (d, *J* = 8.1 Hz, 2H), 3.85 (td, *J* = 8.9, 2.2 Hz, 2H), 2.84-2.71 (m, 2H), 2.32 (s, 3H); **^{13}C NMR** (100 MHz, $CDCl_3$) δ 151.0, 145.1, 136.8, 134.7, 129.8, 128.7, 127.8, 127.3, 126.5, 125.6, 124.2, 120.2, 115.1, 47.2, 21.5, 18.8.

9-methyl-4,9-dihydro-3H-pyrido-[3,4-b]-indole (2l)

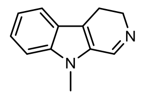

2l

The compound **2l** was prepared using the same procedure as reference.[3] **^{1}H NMR** (600 MHz, $CDCl_3$) δ 8.50 (s, 1H), 7.59 (d, *J* = 8.0 Hz, 1H), 7.32 (s, 2H), 7.14 (t, *J* = 6.8 Hz, 1H), 3.91 (t, *J* = 8.0 Hz, 2H), 3.83 (s, 3H), 2.89 (t, *J* = 8.6 Hz, 2H); **^{13}C NMR** (150 MHz, $CDCl_3$) δ 150.1, 138.0, 129.4, 124.7, 124.3, 120.1, 120.0, 115.6, 109.9, 48.5, 29.4, 19.1.

3,4-dihydroisoquinoline (2q)

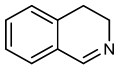

2q

The compound **2q** was prepared using the same procedure as reference.[4] **^{1}H NMR** (400 MHz, $CDCl_3$) δ 8.32 (t, *J* = 2.0 Hz, 1H), 7.39-7.20 (m, 3H), 7.13 (d, *J* = 7.3 Hz, 1H), 3.81-3.69 (m, 2H), 2.79-2.64 (m, 2H); **^{13}C NMR** (100 MHz, $CDCl_3$) δ 160.0, 135.9, 130.7, 128.1, 127.1, 126.8, 126.7, 47.0, 24.7.

5-bromo-3,4-dihydroisoquinoline (2r)

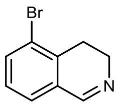

2r

2r was prepared according to the same procedure as **2q**. **^{1}H NMR** (400 MHz, $CDCl_3$) δ 8.25 (s, 1H), 7.58 (dd, *J* = 7.7, 1.2 Hz, 1H), 7.30-7.07 (m, 2H), 3.99-3.66 (m, 2H), 2.95-2.69 (m, 2H); **^{13}C NMR** (100 MHz, $CDCl_3$) δ 159.4, 136.0, 134.8, 130.1, 128.3, 126.3, 123.7, 47.2, 24.5.

Syntheses of acyl halides：

List of acyl halides

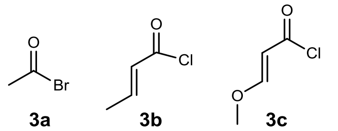

3a **3b** **3c**

3a, 3b were commercial available.

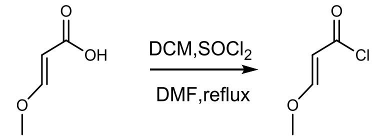

3c' **3c**

(*E*)-3-methoxyacryloyl chloride (3c)

To the stirred solution of **3c'**[5] (1.0 eq.) in DCM were added $SOCl_2$ (4.0 eq.) and DMF (two drops) at room temperature. The reaction solution was refluxed for 1 hour. And the resulting solution was concentrated in vacuo to obtain **3c** for the next step directly.

Note: We try to activate the imine by initial formation of acyl iminium cation follow by Mannich and Wittig type reaction, and the reaction proceeded smoothly (yield = 25%), albeit with the very poor enantioselectivity (ee <10%). (Reaction conditions: reactions were carried out with **2a** (0.1 mmol), crotonoyl chloride (0.15 mmol) in 1 mL DCE, then acetaldehyde (0.15 mmol), catalyst (0.02 mmol) were added until the stating materials disappeared via TLC detection. After reaction is complete, Wittig reagent **4** (0.2 mmol) was added sequentially to the reaction system).

Supplementary Note 3

Screening of reaction conditions

Supplementary Table 1. Screening of reaction conditions[a]

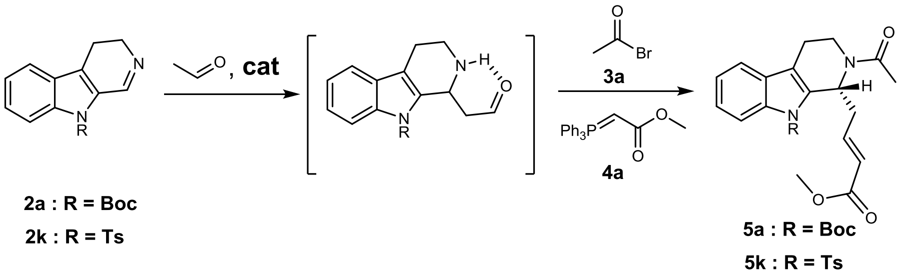

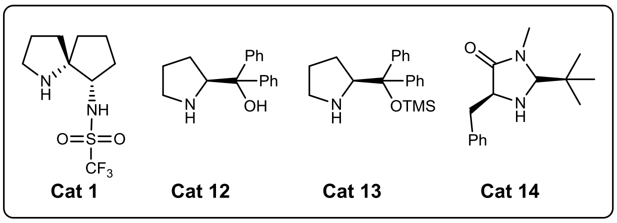

entry	Substrate	catalysts	solvent	additive	yield/%[b]	ee/%[c]
1	**2k**	**Cat 1**	i-PrOH	-	<5	0.6
2	**2k**	**Cat 1**	THF	-	12	52.
3	**2k**	**Cat 1**	DMF	-	11	54
4	**2k**	**Cat 1**	toluene	-	8	21
5	**2k**	**Cat 1**	Et_2O	-	7	35
6	**2k**	**Cat 1**	DCM	-	25	51
7	**2k**	**Cat 1**	DCE	-	28	54
8	**2k**	**Cat 1**	1,4-dioxane	-	-	N.D.[d]
9	**2a**	**Cat 1**	DCE	-	30	67
10	**2a**	**Cat 1**	DCE	Et_3N(0.5eq)	35	83
11	**2a**	**Cat 1**	DCE/ 1eq H_2O	Et_3N(0.5eq)	35	85
12	**2a**	**Cat 1**	DCE/ 5eq H_2O	Et_3N(0.5eq)	47	87
13	**2a**	**Cat 1**	DCE/ 100eq H_2O	Et_3N(0.5eq)	50	89
14	**2a**	**Cat 1**	DCE/ 0.5mL H_2O	Et_3N(0.5eq)	51	90
15	**2a**	**Cat 1**	DCE/ 0.7mL H_2O	Et_3N(0.5eq)	48	85
16	**2a**	**Cat 1**	DCE/ 0.5mL H_2O	PhCOOH (0.5eq)	8	-1
17	**2a**	**Cat 1**	DCE/ 0.5mL H_2O	PTS (0.5eq)	8	-57
18	**2a**	**Cat 1**	DCE/0.5mL H_2O	pyridine (0.5eq)	31	79
19	**2a**	**Cat 1**	DCE/ 0.5mL H_2O	DBU (0.5eq)	47	86
20	**2a**	**Cat 1**	DCE/ 0.5mL H_2O	Na_2CO_3 (0.5eq)	50	83
21	**2a**	**Cat 1**	DCE/ 0.5ml H_2O	Et_3N(0.1eq)	46	88
22	**2a**	**Cat 1**	DCE/ 0.5ml H_2O	Et_3N(0.3eq)	45	87
23	**2a**	**Cat 1**	DCE/ 0.5ml H_2O	Et_3N(1.0eq)	47	89
24	**2a**	**Cat 1**	DCE/ 0.5ml H_2O	Et_3N(2.0eq)	48	89
25	**2a**	**Cat 14**	DCE/ 0.5ml H_2O	Et_3N(0.5eq)	<5	18

[a] Unless otherwise noted, all reactions were carried out with **2** (0.1 mmol), acetaldehyde (0.3 mmol), catalyst (0.02 mmol), in 1 mL solvent, after reaction is complete, the solution was evaporated, then DCM (1mL), K_2CO_3 (0.2 mmol), acetyl bromide **3a** (0.15 mmol) and Wittig reagent **4a** (0.2 mmol) was added sequentislly to the reaction system. [b] Islated yield. [c] Determined by chiral HPLC analysis. [d] Not reaction detected. DCE = 1,2-dichloroethane; PTS = *p*-toluenesulfonic acid; DMAP = 4-dimethylaminopyridine; DBU = 1,8-diazabicyclo[5.4.0]undec-7-ene.

Supplementary Note 5

Products scope:

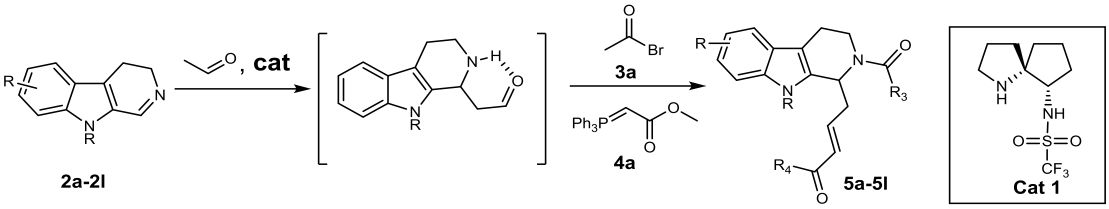

General procedure: To a solution of substituted 3,4-dihydro-β-carboline **2** (0.1 mmol, 1.0 eq.), **Cat 1** (5.4 mg, 0.02 mmol, 0.2 eq.) in DCE/H_2O (1 mL, *v*/*v* 1: 1) were added Et_3N (7 μL, 0.05 mmol, 0.5 eq.) and aldehyde (16 μL, 0.3 mmol, 3.0 eq.) at 0 °C. The mixture was stirred at this temperature until the substrate disappeared via TLC detection. Then the resulting solution was concentrated in vacuo. Then 1 mL DCM, K_2CO_3 (27.6 mg, 0.2 mmol, 2.0 eq.) and acetyl bromide **3a** (10 μL, 0.12 mmol, 1.2eq.) were added at 0 °C sequentially. About 30 min later, **4a** (66.8 mg, 0.2 mmol, 2.0 eq.) was added to the reaction at room temperature and reacted at the same temperature for 12 hours. The mixture was then quenched with water, and the mixture was extracted with DCM. Then the combined organic phase was washed with saturated brine, dried over anhydrous Na_2SO_4, and concentrated in vacuo. The residue was purified through column chromatography on neutral alumina (petroleum ether: EtOAc = 7: 1 to 4: 1) to give substrates **5a-5l**.

tert-butyl-(*R,E*)-2-acetyl-1-(4-methoxy-4-oxobut-2-en-1-yl)-1,2,3,4-tetrahydro-9H-pyrido-[3,4-b]-indole-9-carboxylate (5a)

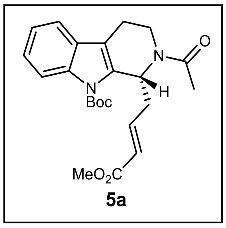

5a was obtained in 51% yield as a colorless oil according to the general procedure. The reaction time for the first step is 4 hours. $[\alpha]_D^{23}$ -58 (c 0.5, $CHCl_3$); **IR** (neat) ν 3360.6, 2923.2, 1725.0, 1654.5, 1455.7, 1421.4, 1312.2, 1260.8, 1141.4, 798.4, 542.4 cm^{-1}; **^{1}H NMR** (400 MHz, $CDCl_3$, mixture of rotamers) : δ 8.13 (d, *J* = 8.2 Hz, 0.5H), 8.04 (d, *J* = 8.2 Hz, 0.5H), 7.42 (dd, *J* = 14.4, 7.4 Hz, 1H), 7.35-7.20 (m, 2H), 7.12-6.89 (m, 1H), 6.55 (dd, *J* = 10.2, 3.0 Hz, 0.5H), 5.91 (dd, *J* = 24.8, 15.6 Hz, 1H), 5.68 (d, *J* = 8.5 Hz, 0.5H), 4.94 (dd, *J* = 13.3, 5.9 Hz, 0.5H), 3.98 (dd, *J* = 12.8, 3.9 Hz, 0.5H), 3.74 (d, *J* = 10.0 Hz, 3H), 3.53-3.45 (m, 0.5H), 3.07-2.53 (m, 4.5H), 2.19 (d, *J* = 7.4 Hz, 3H), 1.71 (d, *J* = 7.6 Hz, 9H); **^{13}C NMR** (100 MHz, $CDCl_3$, mixture of rotamers): δ 169.8, 169.4, 166.5, 166.2, 150.1, 149.7, 144.9, 143.7, 135.9, 135.6, 134.6, 133.9, 128.5, 128.4, 124.6, 124.4, 124.2, 123.0, 122.8, 122.8, 118.2,

117.8, 116.3, 115.8, 115.8, 114.4, 84.5, 84.4, 54.0 51.6, 51.5, 48.0, 39.0, 36.9, 36.6, 33.9, 28.2, 28.2, 22.2, 21.7, 21.6, 20.5; **HRMS (ESI)** calcd for $[M+Na]^+$ $C_{23}H_{28}N_2O_5Na$, m/z: 435.1896, found: 435.1900; Enantiomeric excess was 91% determined by HPLC (IA-3, Hexane/Isopropanol 95/5, flow rate = 1.0 mL/min, 253 nm): major isomer: tr = 18.13 min; minor isomer: tr = 15.28 min.

tert-butyl-(*R,E*)-2-acetyl-6-methoxy-1-(4-methoxy-4-oxobut-2-en-1-yl)-1,2,3,4-tetrahydro-9H-pyrido-[3,4-b]-indole-9-carboxylate (5b)

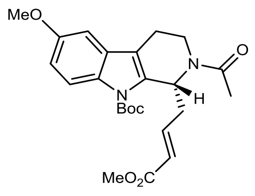

5b

5b was obtained in 45% yield as a colorless oil according to the general procedure. The reaction time for the first step is 8 hours. $[\alpha]_D^{23}$ -30 (c 0.1, $CHCl_3$); **IR** (neat) ν 3358.6, 2926.3, 1724.3, 1644.2, 1459.6, 1373.6, 1321.5, 1225.5, 1136.3, 909.0, 734.2 cm^{-1}; **^{1}H NMR** (600 MHz, $CDCl_3$, mixture of rotamers) δ 8.00 (d, *J* = 9.0 Hz, 0.5H), 7.91 (d, *J* = 9.0 Hz, 0.5H), 7.07-6.93 (m, 1H), 6.93-6.87 (m, 1H), 6.85 (dd, *J* = 16.5, 2.5 Hz, 1H), 6.52 (dd, *J* = 10.1, 3.0 Hz, 0.4H), 5.90 (dd, *J* = 38.0, 15.6 Hz, 1H), 5.66 (d, *J* = 8.8 Hz, 0.5H), 4.94 (dd, *J* = 13.3, 6.1 Hz, 0.5H), 3.98 (dd, *J* = 14.1, 5.6 Hz, 0.5H), 3.86 (d, *J* = 2.9 Hz, 3H), 3.74 (t, *J* = 9.7 Hz, 3H), 3.53-3.43 (m, 0.5H), 3.06-2.96 (m, 1H), 2.91 (dd, *J* = 13.0, 7.1 Hz, 0.5H), 2.83-2.56 (m, 3H), 2.18 (d, *J* = 11.6 Hz, 3H), 1.70 (d, *J* = 9.7 Hz, 9H); **^{13}C NMR** (150 MHz, $CDCl_3$, mixture of rotamers) δ 169.9, 169.5, 166.6, 166.3, 156.2, 156.1, 150.1, 149.7, 144.9, 143.7, 135.5, 134.8, 130.7, 130.4, 129.6, 129.4, 124.3, 123.0, 116.8, 116.7, 116.2, 114.3, 113.1, 112.6, 101.1, 101.0, 84.4, 84.4, 55.7, 54.1, 51.6, 51.5, 48.2, 39.1, 37.0, 36.6, 34.0, 28.3, 22.2, 21.7, 21.7, 20.6; **HRMS (ESI)** calcd for $[M+Na]^+$ $C_{24}H_{30}N_2O_6Na$, m/z: 465.2002, found: 465.2007; Enantiomeric excess was 88% determined by HPLC (IA-3, Hexane/Isopropanol 95/5, flow rate = 1.0 mL/min, 250 nm): major isomer: tr = 23.02 min; minor isomer: tr = 20.74 min.

tert-butyl-(*R,E*)-2-acetyl-1-(4-methoxy-4-oxobut-2-en-1-yl)-6-methyl-1,2,3,4-tetrahydro-9H-pyrido-[3,4-b]-indole-9-carboxylate (5c)

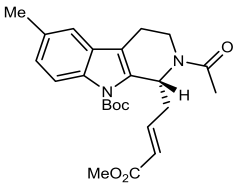

5c

5c was obtained in 45% yield as a colorless oil according to the general procedure. The reaction time for

the first step is 8 hours. $[\alpha]_D^{24}$ -54 (c 0.5, $CHCl_3$); **IR** (neat) ν 3359.3, 2923.0, 2374.6, 1722.7, 1630.1, 1421.4, 1261.5, 1133.9, 1023.0, 736.7, 605.9 cm^{-1}; **^{1}H NMR** (400 MHz, $CDCl_3$,mixture of rotamers) δ 7.98 (d, *J* = 8.5 Hz, 0.5H), 7.90 (d, *J* = 8.5 Hz, 0.5H), 7.20 (d, *J* = 15.7 Hz, 1H), 7.14-7.10 (m, 1H), 7.09-6.92 (m, 1H), 6.53 (dd, *J* = 10.1, 3.1 Hz, 0.5H), 5.90 (dd, *J* = 25.0, 15.6 Hz, 1H), 5.66 (d, *J* = 8.3 Hz, 0.5H), 4.93 (dd, *J* = 13.3, 5.9 Hz, 0.5H), 4.01-3.93 (m, 0.5H), 3.79-3.69 (m, 3H), 3.52-3.44 (m, 0.5H), 3.06-2.87 (m, 1.5H), 2.86-2.52 (m, 3H), 2.44 (s, 3H), 2.18 (d, *J* = 7.5 Hz, 3H), 1.70 (d, *J* = 6.6 Hz, 9H); **^{13}C NMR** (100 MHz, $CDCl_3$, mixture of rotamers) δ 169.9, 169.5, 166.6, 166.3, 150.2, 149.7, 145.0, 143.8, 134.6, 134.1, 134.0, 133.8, 132.6, 132.3, 128.7, 128.6, 125.9, 125.7, 124.2, 122.8, 118.3, 117.8, 116.1, 115.5, 115.5, 114.2, 84.3, 84.3, 54.0, 51.6, 51.5, 48.0, 39.1, 37.0, 36.6, 34.0, 28.2, 28.2, 22.3, 21.8, 21.6, 21.2, 21.2, 20.5; **HRMS (ESI)** calcd for $[M+Na]^+$ $C_{24}H_{30}N_2O_5Na$, m/z: 449.2052, found: 449.2043; Enantiomeric excess was 88% determined by HPLC (IA-3, Hexane/Isopropanol 95/5, flow rate = 1.0 mL/min, 270 nm): major isomer: tr = 16.29 min; minor isomer: tr = 14.24 min.

tert-butyl-(*R,E*)-2-acetyl-6-fluoro-1-(4-methoxy-4-oxobut-2-en-1-yl)-1,2,3,4-tetrahydro-9H-pyrido-[3,4-b]-indole-9-carboxylate (5d)

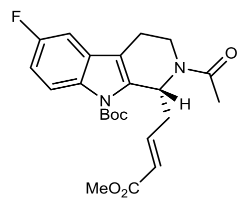

5d

5d was obtained in 52% yield as a colorless oil according to the general procedure. The reaction time for the first step is 8 hours. $[\alpha]_D^{23}$ -68 (c 0.5, $CHCl_3$); **IR** (neat) ν 3356.1, 2924.5, 1726.3, 1645.5, 1372.2, 1265.5, 1131.3, 1023.7, 848.3, 737.8, 604.4 cm^{-1}; **^{1}H NMR** (400 MHz, $CDCl_3$, mixture of rotamers) δ 8.07 (dd, *J* = 9.0, 4.5 Hz, 0.5H), 7.98 (dd, *J* = 9.0, 4.4 Hz, 0.5H), 7.12-6.88 (m, 3H), 6.54 (dd, *J* = 10.2, 3.1 Hz, 0.5H), 5.91 (dd, *J* = 24.5, 15.6 Hz, 1H), 5.66 (d, *J* = 8.3 Hz, 0.5H), 4.95 (dd, *J* = 13.4, 6.0 Hz, 0.5H), 3.99 (dd, *J* = 14.3, 5.0 Hz, 0.5H), 3.74 (d, *J* = 9.6 Hz, 3H), 3.49 (m, 0.5H), 3.05-2.97 (m, 1H), 2.94-2.54 (m, 3.5H), 2.18 (d, *J* = 9.4 Hz, 3H), 1.71 (d, *J* = 7.2 Hz, 9H); **^{13}C NMR** (100 MHz, $CDCl_3$, mixture of rotamers) δ 169.8, 169.5, 166.5, 166.3, 149.8, 149.5, 144.7, 143.5, 136.3, 135.7, 132.2, 131.9, 129.6, 129.5, 129.4, 129.3, 124.3, 123.0, 117.0, 116.9, 116.8, 116.1, 116.1, 114.2, 114.2, 112.3, 112.1, 111.9, 104.1, 103.9, 103.7, 103.4, 84.9, 84.8, 54.0, 51.7, 51.6, 48.0, 38.9, 36.9, 36.5, 33.8, 28.2, 28.2, 22.3, 21.7, 21.6, 20.4; **^{19}F NMR** (375 MHz, $CDCl_3$, mixture of rotamers) δ -119.90, -120.28; **HRMS (ESI)** calcd for $[M+H]^+$ $C_{23}H_{28}N_2O_5F$, m/z: 431.1982, found: 431.1988; Enantiomeric excess was 90% determined by HPLC (IA-3, Hexane/Isopropanol 95/5, flow rate = 1.0 mL/min, 250 nm): major isomer: tr = 19.03 min; minor isomer: tr = 15.66 min.

tert-butyl-(*R,E*)-2-acetyl-6-chloro-1-(4-methoxy-4-oxobut-2-en-1-yl)-1,2,3,4-tetrahydro-9H-pyrido-[3,4-b]-indole-9-carboxylate (5e)

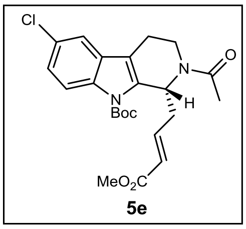

5e was obtained in 54% yield as a colorless oil according to the general procedure. The reaction time for the first step is 8 hours. $[\alpha]_D^{24}$ -42 (c 0.5, $CHCl_3$); **IR** (neat) ν 2926.6, 1728.2, 1647.3, 1438.5, 1371.8, 1264.9, 1145.2, 1021.8, 740.4 cm^{-1}; **^{1}H NMR** (400 MHz, $CDCl_3$, mixture of rotamers) δ 8.04 (d, *J* = 8.9 Hz, 0.5H), 7.95 (d, *J* = 8.9 Hz, 0.5H), 7.40 (d, *J* = 2.0 Hz, 0.5H), 7.36 (d, *J* = 2.0 Hz, 0.5H), 7.27-7.23 (m, 1H), 7.09-6.86 (m, 1H), 6.54 (dd, *J* = 10.2, 3.1 Hz, 0.5H), 5.91 (dd, *J* = 24.3, 15.6 Hz, 1H), 5.66 (d, *J* = 8.1 Hz, 0.5H), 4.95 (dd, *J* = 13.4, 6.0 Hz, 0.5H), 3.99 (dd, *J* = 14.3, 4.0 Hz, 0.5H), 3.78-3.68 (m, 3H), 3.53-3.45 (m, 0.5H), 3.06-2.94 (m, 1H), 2.94-2.52 (m, 3.5H), 2.18 (d, *J* = 9.4 Hz, 3H), 1.71 (d, *J* = 7.2 Hz, 9H); **^{13}C NMR** (100 MHz, $CDCl_3$, mixture of rotamers) δ 169.8, 169.5, 166.5, 166.3, 149.8, 149.4, 144.6, 143.5, 136.1, 135.4, 134.3, 134.0, 130.0, 129.8, 129.7, 128.7, 128.5, 124.7, 124.5, 124.4, 123.0, 118.0, 117.6, 117.0, 116.9, 115.8, 113.9, 85.1, 85.0, 53.9, 51.7, 51.6, 47.9, 38.9, 36.9, 36.5, 33.8, 28.2, 28. 2, 22.3, 21.8, 21.5, 20.4; **HRMS (ESI)** calcd for $[M+H]^+$ $C_{23}H_{27}N_2O_5ClNa$, m/z: 469.1506, found: 469.1521; Enantiomeric excess was 92% determined by HPLC (IA-3, Hexane/Isopropanol 95/5, flow rate = 1.0 mL/min, 250 nm): major isomer: tr = 21.35 min; minor isomer: tr = 17.58 min.

tert-butyl-(*R,E*)-2-acetyl-6-bromo-1-(4-methoxy-4-oxobut-2-en-1-yl)-1,2,3,4-tetrahydro-9H-pyrido-[3,4-b]-indole-9-carboxylate (5f)

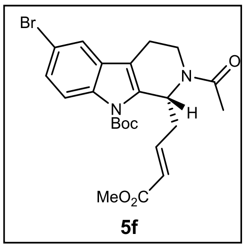

5f was obtained in 53% yield as a colorless oil according to the general procedure. The reaction time for the first step is 8 hours. $[\alpha]_D^{24}$ -32 (c 0.5, $CHCl_3$); **IR** (neat) ν 2926.6, 1728.5, 1647.7, 1371.9, 1265.1, 1144.6, 740.1 cm^{-1}; **^{1}H NMR** (400 MHz, $CDCl_3$, mixture of rotamers) δ 8.00 (d, *J* = 8.9 Hz, 0.5H), 7.90 (d, *J* = 8.9 Hz, 0.5H), 7.55 (d, *J* = 1.9 Hz, 0.5H), 7.51 (d, *J* = 1.9 Hz, 0.5H), 7.41-7.37 (m, 1H), 7.07-6.89 (m, 1H), 6.54 (dd, *J* = 10.2, 3.1 Hz, 0.5H), 5.91 (dd, *J* = 24.3, 15.6 Hz, 1H), 5.66 (d, *J* = 8.2 Hz, 0.5H), 4.94 (dd, *J* = 13.3, 6.0 Hz, 0.5H), 3.99 (dd, *J* = 14.3, 3.8 Hz, 0.5H), 3.74 (d, *J* = 9.5 Hz, 3H), 3.52-3.45 (m, 0.5H), 3.04-2.96 (m, 1H), 2.94-2.52 (m, 3.5H), 2.18 (d, *J* = 9.5 Hz, 3H), 1.71 (d, *J* = 7.0 Hz, 9H); **^{13}C NMR** (100 MHz, $CDCl_3$, mixture of rotamers) δ 169.9, 169.6, 166.6, 166.3, 149.8, 149.4,

144.7, 143.5, 136.0, 135.3, 134.8, 134.5 130.4, 130.2, 127.5, 127.3, 124.5, 123.1, 121.2, 120.7, 117.4, 116.4, 116.2, 115.7, 113.8, 110.0, 85.2, 85.1, 54.0, 51.8, 51.6, 48.0, 39.0, 37.0, 36.6, 33.9, 28.3, 22.3, 21.8, 21.6; **HRMS (ESI)** calcd for $[M+Na]^+$ $C_{23}H_{27}N_2O_5BrNa$, m/z: 513.1001, found: 513.1007; Enantiomeric excess was 92% determined by HPLC (IA-3, Hexane/Isopropanol 95/5, flow rate = 1.0 mL/min, 275 nm): major isomer: tr = 22.27 min; minor isomer: tr = 18.46 min.

tert-butyl-(*R,E*)-2-acetyl-5-bromo-1-(4-methoxy-4-oxobut-2-en-1-yl)-1,2,3,4-tetrahydro-9H-pyrido-[3,4-b-]-indole-9-carboxylate (5g)

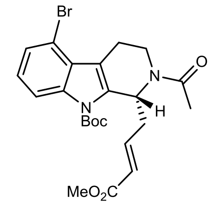

5g

5g was obtained in 51% yield as a colorless oil according to the general procedure. The reaction time for the first step is 6 hours. $[\alpha]_D^{24}$ -46 (c 0.5, $CHCl_3$); **IR** (neat) ν 3359.8, 2922.9, 2247.8, 1728.5, 1656.2, 1425.1, 1310.6, 1278.8, 1135.0, 1026.3, 908.9, 734.6 cm^{-1}; **^{1}H NMR** (400 MHz, $CDCl_3$, mixture of rotamers) δ 8.13 (d, *J* = 8.1 Hz, 0.6H), 8.03 (d, *J* = 8.2 Hz, 0.4H), 7.40-7.32 (m, 1H), 7.13-6.87 (m, 2H), 6.56 (dd, *J* = 10.2, 3.0 Hz, 0.6H), 5.91 (dd, *J* = 23.4, 15.6 Hz, 1H), 5.71 (d, *J* = 7.8 Hz, 0.4H), 4.92 (dd, *J* = 12.7, 5.6 Hz, 0.4H), 3.97 (dd, *J* = 13.5, 6.4 Hz, 0.6H), 3.74 (d, *J* = 9.2 Hz, 3H), 3.51-3.27 (m, 1.6H), 3.07 (m, 1H), 3.02-2.92 (m, 1H), 2.87 (m, 0.4H), 2.77-2.54 (m, 1H), 2.17 (d, *J* = 12.0 Hz, 3H), 1.71 (d, *J* = 6.6 Hz, 9H); **^{13}C NMR** (100 MHz, $CDCl_3$, mixture of rotamers) δ 169.6, 169.4, 166.5, 166.2, 149.7, 149.3, 144.5, 143.4, 137.3, 137.0, 135.9, 135.2, 127.4, 127.3, 127.2, 127.0, 125.2, 125.1, 124.4, 123.1, 116.7, 115.0, 114.9, 114.7, 114.1, 113.5, 85.3, 85.3, 53.8, 51.6, 51.5, 47.8, 39.0, 37.0, 36.6, 33.9, 28.2, 28.2, 24.2, 23.1, 22.1, 21.6; **HRMS (ESI)** calcd for $[M+Na]^+$ $C_{23}H_{27}N_2O_5BrNa$, m/z: 513.1001, found: 513.1002; Enantiomeric excess was 91% determined by HPLC (IA-3, Hexane/Isopropanol 95/5, flow rate = 1.0 mL/min, 280 nm): major isomer: tr =18.22 min; minor isomer: tr = 14.61 min.

tert-butyl-(*R,E*)-2-acetyl-7-fluoro-1-(4-methoxy-4-oxobut-2-en-1-yl)-1,2,3,4-tetrahydro-9H-pyrido-[3,4-b]-indole-9-carboxylate (5h)

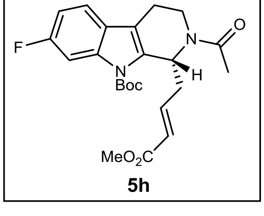

5h

5h was obtained in 52% yield as a colorless oil according to the general procedure. The reaction time

for the first step is 8 hours. $[\alpha]_D^{23}$ -30 (c 0.5, $CHCl_3$); **IR** (neat) ν 3358.7, 2923.6, 2247.4, 1728.2, 1654.8, 1487.5, 1421.9, 1372.1, 1316.9, 1272.1, 1152.9, 1023.0, 909.5, 732.9 cm^{-1}; **^{1}H NMR** (400 MHz, $CDCl_3$, mixture of rotamers) δ 7.86 (dd, *J* = 10.8, 2.2 Hz, 0.5H), 7.76 (d, *J* = 10.8 Hz, 0.5H), 7.33 (ddd, *J* = 17.3, 8.5, 5.5 Hz, 1H), 7.10-6.90 (m, 2H), 6.52 (dd, *J* = 10.2, 3.1 Hz, 0.5H), 5.91 (dd, *J* = 24.3, 15.6 Hz, 1H), 5.65 (d, *J* = 8.4 Hz, 0.5H), 4.94 (dd, *J* = 13.3, 6.0 Hz, 0.5H), 4.03-3.93 (m, 0.5H), 3.74 (d, *J* = 9.5 Hz, 3H), 3.49 (ddd, *J* = 14.3, 10.7, 6.0 Hz, 0.5H), 3.00 (ddd, *J* = 11.6, 8.3, 4.2 Hz, 1H), 2.95-2.53 (m, 3.5H), 2.18 (d, *J* = 8.5 Hz, 3H), 1.71 (d, *J* = 7.6 Hz, 9H); **^{13}C NMR** (100 MHz, $CDCl_3$, mixture of rotamers) δ 169.9, 169.5, 166.6, 166.3, 149.9, 149.5, 144.8, 143.6, 124.9, 124.9, 124.8, 124.7, 124.3, 123.0, 118.4, 118.3, 116.1, 114.2, 111.3, 111.1, 111.1, 110.8, 103.7, 103.7, 103.4, 103.4, 85.1, 85.0, 54.0, 51.7, 51.6, 47.9, 39.0, 37.0, 36.6, 33.9, 28.2, 28.2, 22.3, 21.8, 21.6, 20.5; **^{19}F NMR** (375 MHz, $CDCl_3$, mixture of rotamers) δ -116.49, -116.93; **HRMS (ESI)** calcd for $[M+Na]^+$ $C_{23}H_{27}N_2O_5FNa$, m/z: 453.1802, found: 453.1800; Enantiomeric excess was 91% determined by HPLC (IA-3, Hexane/Isopropanol 95/5, flow rate = 1.0 mL/min, 250 nm): major isomer: tr =20.32 min; minor isomer: tr = 16.12 min.

tert-butyl-(*R,E*)-2-acetyl-8-bromo-1-(4-methoxy-4-oxobut-2-en-1-yl)-1,2,3,4-tetrahydro-9H-pyrido-[3,4-b]-indole-9-carboxylate (5i)

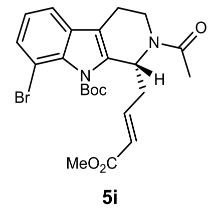

5i

5i was obtained in 43% yield as a colorless oil according to the general procedure. The reaction time for the first step is 30 hours. $[\alpha]_D^{23}$ -166 (c 0.5, $CHCl_3$); **IR** (neat) ν 2925.6, 1726.9, 1647.6, 1422.2, 1370.8, 1306.2, 1149.0, 908.7, 734.0 cm^{-1}; **^{1}H NMR** (600 MHz, $CDCl_3$, mixture of rotamers) δ 7.48 (t, *J* = 7.7 Hz, 1H), 7.38 (d, *J* = 7.7 Hz, 0.4H), 7.34 (d, *J* = 7.7 Hz, 0.6H), 7.11 (q, *J* = 8.0 Hz, 1H), 6.98 (ddt, *J* = 34.9, 14.9, 7.3 Hz, 1H), 6.21 (d, *J* = 7.5 Hz, 0.6H), 5.95 (d, *J* = 15.5 Hz, 0.4H), 5.88 (d, *J* = 15.6 Hz, 0.6H), 5.25 (d, *J* = 9.5 Hz, 0.4H), 4.94 (dd, *J* = 13.3, 6.1 Hz, 0.4H), 3.99 (dd, *J* = 14.2, 5.8 Hz, 0.6H), 3.73 (d, *J* = 15.3 Hz, 3H), 3.49-3.39 (m, 0.6H), 3.17 (d, *J* = 15.4 Hz, 0.6H), 3.09 (dd, *J* = 13.3, 6.8 Hz, 0.4H), 2.97 (td, *J* = 12.7, 4.5 Hz, 0.4H), 2.80 (dt, *J* = 12.1, 6.3 Hz, 1H), 2.76-2.61 (m, 2H), 2.16 (d, *J* = 35.1 Hz, 3H), 1.67 (d, *J* = 5.5 Hz, 9H); **^{13}C NMR** (150 MHz, $CDCl_3$, mixture of rotamers) δ 169.7, 169.5, 166.5, 166.2, 149.5, 148.8, 144.6, 143.3, 137.2, 136.3, 135.7, 135.5, 132.4, 132.3, 129.5, 129.2, 124.5, 124.4, 124.2, 123.1, 117.6, 117.2, 115.6, 113.7, 109.4, 109.3, 85.7, 85.6, 54.0, 51.6, 51.5, 47.9, 39.2, 37.2, 37.0, 34.1, 28.0, 27.9, 22.1, 21.7, 21.7, 20.6; **HRMS (ESI)** calcd for $[M+Na]^+$ $C_{23}H_{27}N_2O_5BrNa$, m/z: 513.1001, found: 513.1003; Enantiomeric excess was 94% determined by HPLC (IA-3, Hexane/Isopropanol 95/5, flow rate = 1.0 mL/min, 250 nm): major isomer: tr = 19.15 min;

minor isomer: tr = 17.76 min.

tert-butyl-(*R,E*)-2-acetyl-1-(4-methoxy-4-oxobut-2-en-1-yl)-8-methyl-1,2,3,4-tetrahydro-9H-pyrido-[3,4-b]-indole-9-carboxylate (5j)

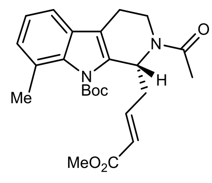

5j

5j was obtained in 37% yield as a colorless oil according to the general procedure. The reaction time for the first step is 10 hours. $[\alpha]_D^{24}$ -134 (c 0.5, $CHCl_3$); **IR** (neat) ν 2925.3, 1724.6, 1659.5, 1459.2, 1371.5, 1264.5, 1148.1, 908.8, 735.3 cm^{-1}; **^{1}H NMR** (600 MHz, $CDCl_3$, mixture of rotamers) δ 7.21 (ddd, *J* = 23.1, 18.0, 7.0 Hz, 2H), 7.10 (t, *J* = 7.6 Hz, 1H), 7.04-6.90 (m, 1H), 6.32 (dd, *J* = 10.2, 3.1 Hz, 0.7H), 5.93 (d, *J* = 15.6 Hz, 0.3H), 5.86 (d, *J* = 15.7 Hz, 0.7H), 5.35 (d, *J* = 8.8 Hz, 0.3H), 4.93 (dd, *J* = 13.3, 6.1 Hz, 0.3H), 3.97 (dd, *J* = 14.2, 5.8 Hz, 0.7H), 3.73 (d, *J* = 15.0 Hz, 3H), 3.49-3.38 (m, 0.7H), 3.13-2.92 (m, 1.3H), 2.86-2.58 (m, 3H), 2.48 (d, *J* = 15.2 Hz, 3H), 2.17 (d, *J* = 23.3 Hz, 3H), 1.67 (d, *J* = 6.1 Hz, 9H); **^{13}C NMR** (150 MHz, $CDCl_3$, mixture of rotamers) δ 169.6, 169.5, 166.5, 166.2, 150.2, 149.8, 144.7, 143.3, 136.6, 136.3, 135.6, 134.7, 130.0, 127.6, 127.4, 126.0, 125.7, 124.4, 123.5, 123.4, 123.0, 116.1, 116.0, 115.5, 114.2, 84.7, 54.1, 51.6, 51.4, 48.0, 39.2, 37.2, 36.9, 34.0, 28.1, 22.1, 21.7, 21.6, 21.1, 21.0, 20.6; **HRMS (ESI)** calcd for $[M+Na]^+$ $C_{24}H_{30}N_2O_5Na$, m/z: 449.2052, found: 449.2048; Enantiomeric excess was 90% determined by HPLC (IA-3, Hexane/Isopropanol 95/5, flow rate = 1.0 mL/min, 245 nm): major isomer: tr = 16.14 min; minor isomer: tr = 13.66 min.

Methyl-(*R,E*)-4-(2-acetyl-9-tosyl-2,3,4,9-tetrahydro-1H-pyrido-[3,4-b]-indol-1-yl)but-2-enoate (5k)

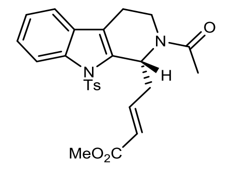

5k

5k was obtained in 52% yield as a colorless oil according to the general procedure. The reaction time for the first step is 6 hours. $[\alpha]_D^{22}$ -96 (c 0.5, $CHCl_3$); **IR** (neat) ν 2925.0, 2852.2, 2246.8, 1721.9, 1649.2, 1451.7, 1421.5, 1212.3, 1173.7, 1147.7, 1023.3, 909.8, 733.2 cm^{-1}; **^{1}H NMR** (400 MHz, $CDCl_3$, mixture of rotamers) δ 8.17 (dd, *J* = 8.0, 3.9 Hz, 1H), 7.73 (d, *J* = 8.4 Hz, 1H), 7.48 (d, *J* = 8.4 Hz, 1H), 7.39-7.21 (m, 3H), 7.15 (dd, *J* = 8.4, 2.1 Hz, 2H), 7.10-6.95 (m, 1H), 6.63 (dd, *J* = 10.4, 2.9 Hz, 0.6H), 6.00 (d, *J* = 15.6 Hz, 0.4H), 5.94 (d, *J* = 15.7 Hz, 0.6H), 5.45 (d, *J* = 9.6 Hz, 0.4H), 4.89 (dd, *J* = 13.5, 6.0 Hz, 0.4H), 4.03 – 3.90 (m, 0.6H), 3.74 (d, *J* = 8.7 Hz, 3H), 3.55-3.40 (m, 0.6H), 3.38-3.18 (m, 1H),

3.04-2.90 (m, 0.4H), 2.83-2.57 (m, 3H), 2.30 (d, J = 18.7 Hz, 3H), 2.15 (d, J = 27.6 Hz, 3H); **^{13}C NMR** (100 MHz, $CDCl_3$, mixture of rotamers) δ 169.8, 169.7, 166.7, 166.4, 145.4, 144.9, 144.8, 143.5, 137.2, 136.6, 135.2, 134.9, 134.3, 134.0, 130.0, 129.8, 129.7, 126.9, 126.0, 125.5, 125.1, 124.6, 124.4, 124.0, 123.0, 120.1, 118.9, 118.4, 117.8, 115.5, 54.5, 51.7, 51.5, 48.2, 38.7, 37.7, 37.4, 33.7, 22.2, 21.8, 21.8, 21.6, 21.5, 20.6; **HRMS (ESI)** calcd for $[M+Na]^+$ $C_{25}H_{26}N_2O_5SNa$, m/z: 489.1460, found: 489.1461; Enantiomeric excess was 83% determined by HPLC (OD-H, Hexane/Isopropanol 80/20, flow rate = 1.0 mL/min, 250 nm): major isomer: tr = 12.12 min; minor isomer: tr = 10.70 min.

Methyl-(*R*,*E*)-4-(2-acetyl-9-methyl-2,3,4,9-tetrahydro-1H-pyrido[3,4-b]indol-1-yl)but-2-enoate (5l)

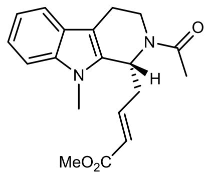

5l

5l was obtained in 32% yield as a colorless oil according to the general procedure. The reaction time for the first step is 10 hours. $[\alpha]_D^{21}$ -35 (c 0.2, $CHCl_3$); **IR** (neat) ν 2924.0, 1661.5, 1436.2, 1333.4, 1106.5, 723.7, 692.7, 541.8 cm^{-1}; **^{1}H NMR** (600 MHz, $CDCl_3$) δ 7.48 (d, J = 7.8 Hz, 1H), 7.30 (d, J = 8.2 Hz, 1H), 7.25-7.20 (m, 1H), 7.17-7.07 (m, 1H), 7.04-6.89 (m, 1H), 6.04 (dd, J = 9.4, 4.1 Hz, 1H), 5.91 (dt, J = 15.6, 1.4 Hz, 1H), 4.07-3.97 (m, 1H), 3.72 (dd, J = 22.8, 14.9 Hz, 6H), 3.52 (ddd, J = 14.1, 10.9, 5.6 Hz, 1H), 2.92-2.76 (m, 3H), 2.72 (ddd, J = 15.3, 9.3, 1.4 Hz, 1H), 2.21 (s, 3H); **^{13}C NMR** (150 MHz, $CDCl_3$) δ 169.6, 166.4, 144.0, 137.5, 134.4, 126.2, 123.5, 121.8, 119.4, 118.1, 109.1, 107.3, 51.6, 46.6, 40.6, 37.0, 30.2, 21.8, 21.7; **HRMS (ESI)** calcd for $[M+Na]^+$ $C_{19}H_{22}N_2O_3Na$, m/z: 349.1523, found: 349.1516; Enantiomeric excess was 76% determined by HPLC (IA-3, Hexane/Isopropanol 90/10, flow rate = 1.0 mL/min, 280 nm): major isomer: tr = 18.47 min; minor isomer: tr = 23.40 min.

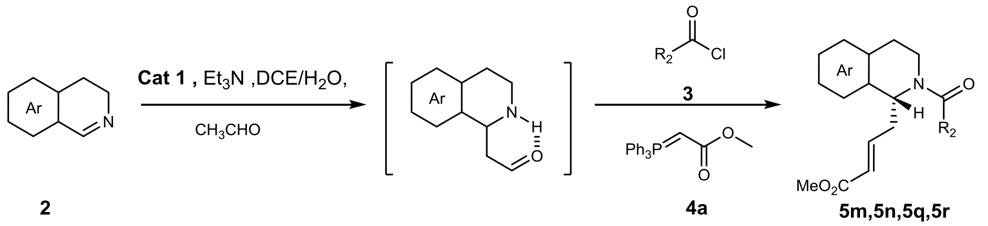

General procedure: To a solution of 3,4-dihydro-β-carboline **2** (0.1 mmol, 1.0 eq.), **Cat 1** (5.4 mg, 0.02 mmol, 0.2 eq.) in DCE/H_2O (1 mL, *v*/*v* 1: 1) were added Et_3N (7 μL, 0.05 mmol, 0.5 eq.) and CH_3CHO (16 μL, 0.3 mmol, 3.0 eq.) at 0 °C. The mixture was stirred at this temperature until the substrate disappeared via TLC detection (about 4 hours). Then the resulting solution was concentrated in vacuo. Then 1 mL DCM, pyridine (16 μL, 0.2 mmol, 2.0 eq.) and acyl chloride **3** (1.5 eq.) were added at 0 °C sequentially. About 30 minutes later, **4a** (66.8 mg, 0.2 mmol, 2.0 eq.) was added to the

reaction at room temperature and reacted at the same temperature for 12 hours. The mixture was then quenched with water, and the mixture was extracted with DCM. Then the combined organic phase was washed with saturated brine, dried over anhydrous Na_2SO_4, and concentrated in vacuo. The residue was purified through column chromatography on neutral alumina (petroleum ether: EtOAc = 5: 1 to 3: 1) to give substrates **5m**, **5n**, **5q**, **5r**.

tert-butyl-(*R*)-1-((*E*)-4-methoxy-4-oxobut-2-en-1-yl)-2-((*E*)-3-methoxyacryloyl)-1,2,3,4-tetrahydro-9H-pyrido-[3,4-b]-indole-9-carboxylate (5m)

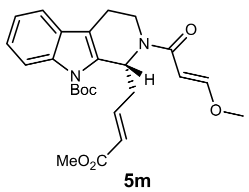

5m

5m was obtained in 45% yield as a colorless oil according to the general procedure. $[\alpha]_D^{18}$ -78 (c 0.5, $CHCl_3$); **IR** (neat) ν 3397.2, 2925.4, 2369.2, 1725.1, 1655.4, 1595.0, 1457.5, 1370.1, 1260.7, 1138.3, 1025.8, 739.6 cm^{-1}; **^{1}H NMR** (600 MHz, $CDCl_3$, mixture of rotamers) δ 8.09 (d, *J* = 68.2 Hz, 1H), 7.60 (d, *J* = 9.3 Hz, 1H), 7.40 (d, *J* = 22.8 Hz, 1H), 7.30 (t, *J* = 7.5 Hz, 1H), 7.24 (s, 1H), 7.03 (s, 1H), 6.62 (s, 0.4H), 5.91 (t, *J* = 19.2 Hz, 1H), 5.83 – 5.54 (m, 1.6H), 4.96 (s, 0.5H), 4.06 (s, 0.5H), 3.72 (s, 6H), 3.50 (d, *J* = 23.1 Hz, 0.5H), 3.03 – 2.91 (m, 1.5H), 2.82 – 2.69 (m, 3H),1.72 (s, 9H); **^{13}C NMR** (150 MHz, $CDCl_3$, mixture of rotamers) δ 166.9, 166.4, 163.0, 150.0, 144.9, 144.3, 136.2, 134.9, 134.4, 128.7, 124.5, 124.0, 122.9, 118.2, 117.9, 115.9, 95.9, 95.4, 84.5, 57.9, 53.3, 51.4, 48.4, 38.6, 37.0, 34.5, 28.3, 22.0, 20.7; **HRMS (ESI)** calcd for $[M+Na]^+$ $C_{25}H_{30}N_2O_6Na$, m/z: 477.1996, found: 477.2000; Enantiomeric excess was 91% determined by HPLC (IF-3, Hexane/Isopropanol 90/10, flow rate = 1.0 mL/min, 246 nm): major isomer: tr = 41.71 min; minor isomer: tr = 49.59 min.

tert-butyl(*R*)-2-((*E*)-but-2-enoyl)-1-((*E*)-4-methoxy-4-oxobut-2-en-1-yl)-1,2,3,4-tetrahydro-9H-pyrido-[3,4-b]-indole-9-carboxylate (5n)

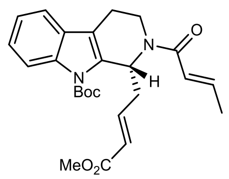

5n

5n was obtained in 46% yield as a colorless oil according to the general procedure. $[\alpha]_D^{24}$ -8 (c 0.5, $CHCl_3$); **IR** (neat) ν 3359.3, 2923.2, 1726.1, 1659.5, 1455.3, 1370.7, 1312.9, 1140.6, 738.9 cm^{-1}; **^{1}H NMR** (400 MHz, $CDCl_3$, mixture of rotamers) δ 8.14 (d, *J* = 7.9 Hz, 0.5H), 8.04 (d, *J* = 8.1 Hz, 0.5H), 7.40 (dd, *J* = 17.6, 7.1 Hz, 1H), 7.27 (dt, *J* = 14.2, 6.7 Hz, 2H), 7.09-6.78 (m, 2H), 6.60 (d, *J* = 8.6 Hz,

0.5H), 6.33 (t, J = 14.1 Hz, 1H), 5.88 (dd, J = 27.6, 12.4 Hz, 1.5H), 5.05-4.83 (m, 0.5H), 4.17 (d, J = 12.9 Hz, 0.5H), 3.71 (d, J = 4.9 Hz, 3H), 3.50 (d, J = 15.5 Hz, 0.5H), 3.04 (d, J = 12.9 Hz, 1H), 2.97-2.52 (m, 3.5H), 1.89 (s, 3H), 1.72 (d, J = 6.0 Hz, 9H); **^{13}C NMR** (100 MHz, $CDCl_3$, mixture of rotamers) δ 166.7, 166.5, 166.4, 166.2, 150.2, 149.7, 144.9, 143.9, 142.3, 142.1, 136.1, 135.8, 134.6, 134.0, 128.6, 128.5, 124.6, 124.4, 124.1, 123.0, 122.9, 122.8, 122.2, 121.6, 118.3, 117.8, 116.5, 115.9, 115.8, 114.6, 84.8, 53.1, 51.4, 48.5, 38.5, 37.1, 36.7, 34.5, 28.2, 22.0, 20.5, 18.2; **HRMS (ESI)** calcd for $[M+Na]^+$ $C_{25}H_{30}N_2O_5Na$, m/z: 461.2052, found: 461.2053; Enantiomeric excess was 91% determined by HPLC (IA-3, Hexane/Isopropanol 95/5, flow rate = 1.0 mL/min, 220 nm): major isomer: tr = 22.13 min; minor isomer: tr = 16.24 min.

methyl(*E*)-4-((*R*)-2-((*E*)-but-2-enoyl)-1,2,3,4-tetrahydroisoquinolin-1-yl)-but-2-enoate (5q)

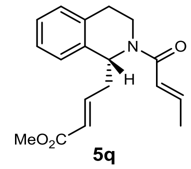

5q

5q was obtained in 41% yield as a colorless oil according to the general procedure. The reaction time for first step is 12 hours. $[\alpha]_D^{24}$ -86 (c 0.5, $CHCl_3$); **IR** (neat) ν 3430.6, 2926.8, 1722.3, 1615.7, 1509.3, 1433.7, 1275.0, 1200.3, 1104.5, 1024.7, 959.9, 733.5 cm^{-1}; **^{1}H NMR** (400 MHz, $CDCl_3$, mixture of rotamers) δ 7.16 (ddd, J = 12.6, 10.8, 5.3 Hz, 4H), 6.90 (tt, J = 14.0, 7.2 Hz, 2H), 6.30 (t, J = 15.3 Hz, 1H), 5.81 (dt, J = 13.1, 10.7 Hz, 1.6H), 5.16 – 5.01 (m, 0.4H), 4.66 (d, J = 10.6 Hz, 0.4H), 3.93 (dt, J = 13.0, 4.9 Hz, 0.6H), 3.71 (d, J = 8.6 Hz, 3H), 3.62-3.49 (m, 0.6H), 3.12 (dd, J = 16.7, 7.6 Hz, 0.4H), 3.05-2.60 (m, 4H), 1.90 (d, J = 6.7 Hz, 3H); **^{13}C NMR** (100 MHz, $CDCl_3$, mixture of rotamers) δ 166.5, 166.0, 144.9, 143.7, 142.2, 141.9, 136.1, 135.5, 134.7, 133.7, 129.3, 128.6, 127.3, 127.2, 126.9, 126.6, 126.5, 126.2, 124.1, 123.2, 121.9, 121.7, 55.6, 52.0, 51.5, 51.4, 40.6, 40.1, 39.3, 35.9, 29.2, 28.0, 26.8, 18.2; **HRMS (ESI)** calcd for $[M+Na]^+$ $C_{18}H_{21}NO_3Na$, m/z: 322.1414, found: 322.1420; Enantiomeric excess was 81% determined by HPLC (OD-H, Hexane/Isopropanol 80/20, flow rate = 1.0 mL/min, 220 nm): major isomer: tr = 8.53 min; minor isomer: tr = 10.79 min.

(*E*)-methyl4-((*R*)-5-bromo-2-((*E*)-but-2-enoyl)-1,2,3,4-tetrahydroisoquinolin-1-yl)-but-2-enoate (5r)

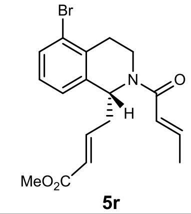

5r

5r was obtained in 36% yield as a colorless oil according to the general procedure. The reaction time for

first step is 10 hours. $[\alpha]_D^{23}$ -112 (c 0.5, $CHCl_3$); **IR** (neat) ν 2925.8, 2367.4, 1721.7, 1659.1, 1437.5, 1292.2, 1210.9, 1131.3, 1026.6, 724.1 cm^{-1}; **^{1}H NMR** (600 MHz, $CDCl_3$, mixture of rotamers) δ 7.54-7.40 (m, 1H), 7.16-7.01 (m, 2H), 6.90 (tt, *J* = 14.5, 7.2 Hz, 2H), 6.29 (dd, *J* = 47.0, 14.7 Hz, 1H), 5.94-5.71 (m, 1.7H), 5.08 (s, 0.3H), 4.77 (d, *J* = 9.1 Hz, 0.3H), 4.04 (d, *J* = 11.5 Hz, 0.7H), 3.72 (d, *J* = 10.9 Hz, 3H), 3.56-3.41 (m, 0.7H), 3.05 (d, *J* = 8.9 Hz, 0.3H), 2.98-2.82 (m, 2H), 2.76 (t, *J* = 7.1 Hz, 2H), 1.90 (d, *J* = 6.8 Hz, 3H); **^{13}C NMR** (150 MHz, $CDCl_3$, mixture of rotamers) δ 166.4, 166.1, 165.9, 144.4, 143.2, 142.6, 142.4, 138.8, 133.5, 131.5, 131.0, 127.7, 127.4, 126.5, 125.9, 125.0, 124.5, 123.6, 121.7, 121.4, 55.6, 51.6, 51.5, 39.8, 39.1, 35.3, 30.0, 28.9, 18.3; **HRMS (ESI)** calcd for $[M+Na]^+$ $C_{18}H_{20}NO_3BrNa$, m/z: 400.0519, found: 400.0516; Enantiomeric excess was 87% determined by HPLC (IA-3, Hexane/Isopropanol 95/5, flow rate = 1.0 mL/min, 240 nm): major isomer: tr = 35.97 min; minor isomer: tr = 38.26 min.

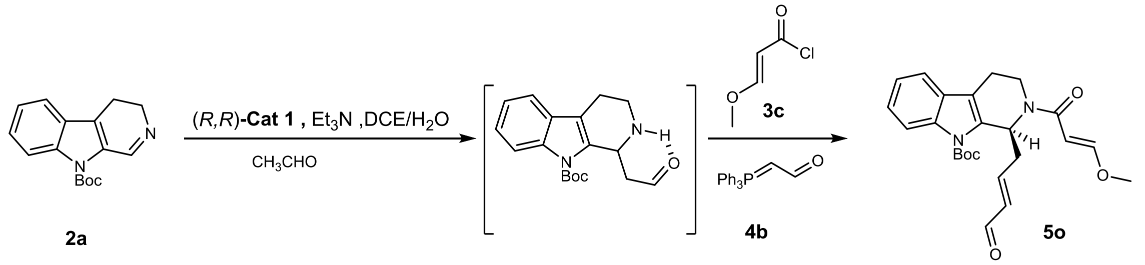

2a 4b 5o

To a solution of 3,4-dihydro-β-carboline **2a** (1.100g, 1.0 eq.), (*R,R*)-**Cat 1** (110.8 mg, 0.1 eq.) in DCE/H_2O (40 mL, *v*/*v* 1: 1) were added Et_3N (285 μL, 0.5 eq.) and CH_3CHO (670 μL, 3.0 eq.) at 0 °C. The mixture was stirred at this temperature until the substrate disappeared via TLC detection (about 4 hours). Then the resulting solution was concentrated in vacuo. Then 40 mL DCM, pyridine (660 μL, 2.0 eq.) and acyl chloride **3c** (1.2 eq.) were added at 0 °C sequentially. The mixture was stirred at room temperature for 5 hours, then the reaction solution was concentrated in vacuo. The residue was dissolved in toluene (40 mL), and **4b** (1.240 g, 2.0 eq.) was added to the reaction at room temperature and the reaction solution was refluxed for 24 hours. The mixture was then quenched with water, and the mixture was extracted with EtOAc. Then the combined organic phase was washed with saturated brine, dried over anhydrous Na_2SO_4, and concentrated in vacuo. The residue was purified through flash chromatography on silica gel (petroleum ether: EtOAc = 3: 1 to 1: 1) to give substrates **5o** (726 mg, 42% yield). $[\alpha]_D^{23}$ +130 (c 0.5, $CHCl_3$); **IR** (neat) ν 2928.5, 1727.5, 1649.4, 1455.4, 1371.2, 1318.2, 1138.8, 752.2 cm^{-1}; **^{1}H NMR** (600 MHz, $CDCl_3$, mixture of rotamers) δ 9.52 (d, J = 7.9 Hz, 1H), 8.10 (dd, J = 40.6, 25.6 Hz, 1H), 7.61 (dd, J = 11.6, 6.1 Hz, 1H), 7.39 (d, J = 6.7 Hz, 1H), 7.31 (t, J = 7.7 Hz, 1H), 7.25 (dd, J = 14.9, 7.6 Hz, 1H), 7.03 – 6.85 (m, 1H), 6.68 (d, J = 9.7 Hz, 0.7H), 6.30 – 6.01 (m, 1H), 5.82 (s, 0.3H), 5.70 (d, J = 11.7 Hz, 1H), 4.99 (s, 0.3H), 4.09 (d, J = 10.4 Hz, 0.7H), 3.72 (d, J = 16.5 Hz, 3H), 3.47 (d, J = 10.7 Hz, 0.7H), 3.23 – 3.02 (m, 1.3H), 2.78 (dd, J = 35.0, 20.8 Hz, 3H), 1.72 (s, 9H). **^{13}C NMR** (150 MHz, $CDCl_3$, mixture of rotamers) δ 194.0, 193.3, 166.7, 163.4, 154.5, 152.9,

149.8, 136.0, 135.8, 135.7, 135.2, 134.6, 134.3, 134.0, 128.5, 124.5, 122.9, 118.3, 117.8, 115.9, 114.8, 95.5, 94.7, 84.7, 84.5, 58.1, 53.1, 48.4, 48.0, 38.4, 37.4, 34.4, 28.2, 21.9, 20.6; **HRMS (ESI)** calcd for $[M+Na]^+$ $C_{24}H_{28}N_2O_5Na$, m/z: 447.1890, found: 447.1902; Enantiomeric excess was 91% determined by **8.**

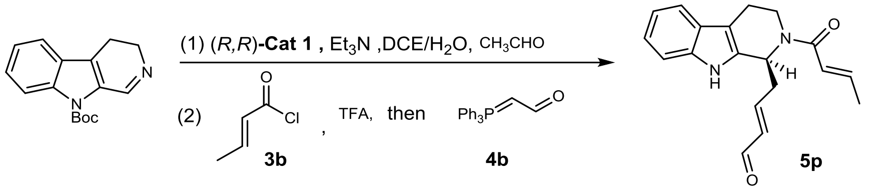

To a solution of 3,4-dihydro-β-carboline **2a** (27.0 mg, 0.1 mmol, 1.0 eq.), (*R,R*)-**Cat 1** (5.4 mg, 0.02 mmol, 0.2 eq.) in DCE/H_2O (1 mL, *v*/*v* 1: 1) was added Et_3N (7 μL, 0.05 mmol, 0.5 eq.) and CH_3CHO (16 μL, 0.3 mmol, 3.0 eq.) at 0 °C. The mixture was stirred at this temperature until the substrate disappeared via TLC detection (about 4 hours). Then the resulting solution was concentrated in vacuo. Then 1 mL DCM, pyridine (16 μL, 0.2 mmol, 2.0 eq.) and acyl chloride **3b** were added at 0 °C sequentially. About 4 hours later, TFA (1 mL) was added to the reaction at 0 °C, and the mixture was stirred at this temperature for 4 hours. And the mixture was then quenched with saturated NH_4Cl aqueous solution and the mixture was extracted with EtOAc. The combined organic phase was washed with brine, dried over Na_2SO_4 and concentrated in vacuo. The residue was dissolved in DCM (1 mL), **4b** (60.8 mg, 0.2 mmol, 2.0 eq.) was added to a stirred solution of the crude product in anhydrous DCM (1 mL) at room temperature. The reaction mixture stirred 12 hours, after which the reaction was quenched by saturated NH_4Cl aqueous solution. The mixture was extracted with DCM. Then the combined organic phase was washed with saturated brine, dried over anhydrous Na_2SO_4, and concentrated in vacuo. The crude product was purified via flash chromatography on silica gel (petroleum ether: EtOAc = 3: 1 to 1: 1) to give compound **5p** (11.7 mg, 38% yield) as a colorless oil. $[\alpha]_D^{19}$ +84 (c 0.5, $CHCl_3$); **IR** (neat) ν 3282.4, 2926.5, 1657.3, 1450.7, 1331.2, 1217.2, 1072.8, 965.6, 739.0 cm^{-1}; **^{1}H NMR** (400 MHz, $CDCl_3$) δ 9.31 (s, 1H), 9.21 (d, *J* = 7.8 Hz, 1H), 7.46 (d, *J* = 7.5 Hz, 1H), 7.35-7.23 (m, 1H), 7.14 (dt, *J* = 26.9, 7.0 Hz, 2H), 6.98 (dd, *J* = 14.5, 7.0 Hz, 1H), 6.84-6.66 (m, 1H), 6.41 (d, *J* = 14.8 Hz, 1H), 6.08 (s, 1H), 5.99 (dd, *J* = 15.4, 7.9 Hz, 1H), 4.23 (d, *J* = 12.9 Hz, 1H), 3.45 (d, *J* = 8.7 Hz, 1H), 2.92 (dd, *J* = 28.8, 22.8 Hz, 4H), 1.96 (d, *J* = 6.3 Hz, 3H); **^{13}C NMR** (100 MHz, $CDCl_3$) δ 193.8, 166.6, 153.2, 143.1, 136.3, 134.8, 132.6, 126.4, 122.1, 121.4, 119.6, 118.1, 111.3, 108.1, 48.7, 40.6, 38.0, 22.2, 18.4; **HRMS (ESI)** calcd for $[M+Na]^+$ $C_{19}H_{20}N_2O_2Na$, m/z: 331.1417, found: 331.1409; Enantiomeric excess was 91% determined by **7**.

Supplementary Note 5

Proposed mechanism:

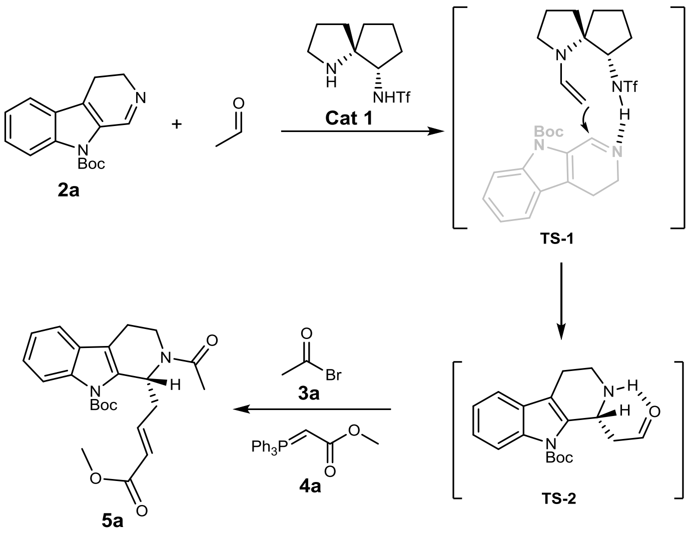

Supplementary Figure 1. The proposed reaction mechanism

Based on screening of the experimental results, a possible reaction mechanism was proposed. **Cat 1** might be proposed as a bifunctional catalyst. Pyrrolidine reacts with aldehyde to form an enamine and the part of TfNH activats imine through hydrogen bonding. The enamine attacks the imine from the *Re* face to afford chiral compound **TS-2**, and the intermediate **TS-2** reacts with acyl halides and Wittig reagents to afford product **5a**.

Supplementary Note 6

Supplementary Table 2. Screening of O-hetero-Diels-Alder reaction conditions[a]

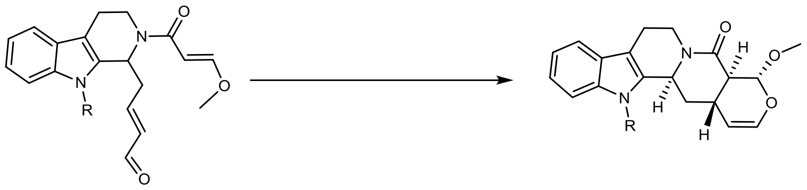

entry	R	solvent	lewis acid	additive	T/°C	time/h	yield/%[b]
1	**H**	Tol	$BF_3{\cdot}Et_2O$	-	-78	12	N.D.[c]
2	**H**	Tol	Et_2AlCl	-	-78	12	N.D. [c]
3	**H**	Tol	$EtAlCl_2$	-	-78	12	<5.
4	**H**	Tol	$ZnCl_2$	-	-78	12	<5
5	**H**	Tol	$ZnBr_2$	-	-78	12	<5
6	**H**	Tol	$AlCl_3$	-	-78	12	N.D.[c]
7	**H**	Tol	$FeCl_3$	-	-78	12	N.D.[c]
8	**H**	DCM	$TiCl_4$	-	rt	4	N.D.[c]
9	**H**	DCM	$Cu(OTf)_2$	-	rt	4	N.D.[c]
10	**H**	DCM	$ZnBr_2$	-	rt	48	12
11	**H**	DCM	$Zn(OTf)_2$	-	rt	48	N.R.
12	**H**	DCM	$InCl_3$	-	rt	48	N.D.[c]
13	**H**	DCM	$SnCl_4$	-	rt	48	N.D.[c]
14	**H**	DCM	$BF_3{\cdot}Et_2O$	-	rt	48	N.D.[c]
15	**H**	DCM	Et_2AlCl	-	rt	48	N.D.[c]
16	**H**	DCM	$EtAlCl_2$	-	rt	48	N.D.[c]
17	**H**	DCM	$ZnCl_2$	-	rt	48	8
18	**H**	DCM	$ZnBr_2$	3Å MS	rt	45	28
19	**H**	DCM	$ZnBr_2$	4Å MS	rt	45	32
20	**H**	DCM	$ZnBr_2$	5Å MS	rt	45	28
21	**Boc**	DCM	$ZnBr_2$	4Å MS	rt	45	40
22	**Boc**	DCM(0.01mol/L)	$ZnBr_2$	4Å MS	rt	45	51
23	**Boc**	DCM(0.01mol/L)	$ZnBr_2$	4Å MS	50	8	51

[a] Unless otherwise noted, all reactions were carried out with the starting material (0.1 mmol, 1.0 eq.) in solvent (0.05 mol/L), lewis acid (0.15 mmol,1.5 eq.), additive (50 mg). [b] Islated yield. [c] The decomposition of the starting material.

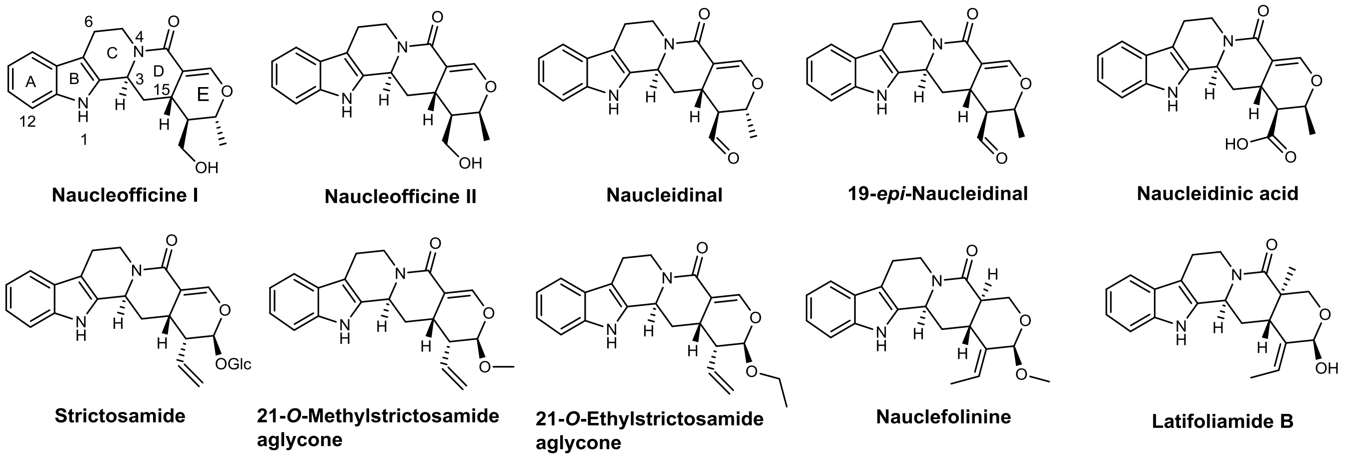

Supplementary Figure 2. Natural products of trans-configuration between C3 and C15

Synthetic utilities :

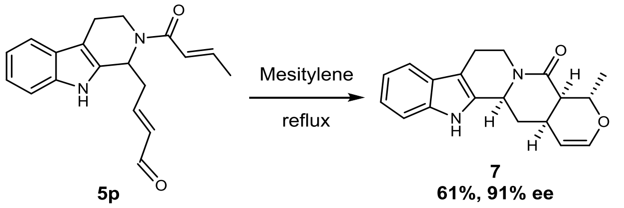

A solution of **5p** (50.1 mg) in mesitylene (15 mL) was heated at 170 °C for 30 hours before cooling to room temperture. The residue was purified through column chromatography on silica gel (petroleum ether: EtOAc = 8: 1 to 3: 1) to give substrates **7** (30.5 mg, 61%) as an amorphous solid. $[\alpha]_D^{20}$ -104 (c 0.5, $CHCl_3$); **IR** (neat) ν 3285.9, 2926.0, 1618.2, 1437.3, 1275.5, 1229.8, 1066.2, 737.0 cm^{-1}; **^{1}H NMR** (400 MHz, $CDCl_3$) δ 8.24 (t, *J* = 8.8 Hz, 1H), 7.49 (d, *J* = 7.7 Hz, 1H), 7.33 (d, *J* = 8.0 Hz, 1H), 7.21-7.15 (m, 1H), 7.15-7.08 (m, 1H), 6.41 (d, *J* = 5.8 Hz, 1H), 5.26-5.11 (m, 1H), 4.75 (dt, *J* = 10.5, 5.2 Hz, 2H), 3.98 (dq, *J* = 9.0, 6.2 Hz, 1H), 2.96-2.71 (m, 3H), 2.71-2.58 (m, 2H), 2.55-2.43 (m, 1H), 1.87-1.68 (m, 1H), 1.45 (d, *J* = 6.3 Hz, 3H); **^{13}C NMR** (100 MHz, $CDCl_3$) δ 167.7, 144.7, 136.3, 132.9, 126.7, 122.2, 119.9, 118.3, 111.0, 109.2, 102.2, 69.8, 53.7, 46.6, 40.4, 34.3, 28.4, 21.1, 19.7; **HRMS (ESI)** calcd for $[M+Na]^+$ $C_{19}H_{20}N_2O_2Na$, m/z: 331.1417, found: 331.1418; Enantiomeric excess was 91% determined by HPLC (IA-3, Hexane/Isopropanol 90/10, flow rate = 1.0 mL/min, 275 nm): major isomer: tr = 15.13 min; minor isomer: tr = 13.20 min.

Total synthesis of (+)-naucleofficine I and (–)-naucleofficine II

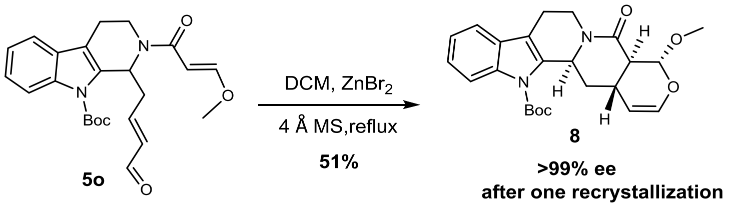

tert-butyl(4*R*,4a*R*,13b*S*,14a*S*)-4-methoxy-5-oxo-4a,7,8,13b,14,14a-hexahydro-4H-indolo[2,3-a]-pyrano-[3,4-g]-quinolizine-13(5H)-carboxylate (8)

To a solution of **5o** (835 mg, 1.0 eq.), 4Å MS (molecular sieves) (2.505 g, 3.0 eq.) in DCM (200 mL) was added $ZnBr_2$ (665 mg, 1.5 eq.). The reaction solution was refluxed for 24 hours, and the reaction mixture was filtered through a plug of diatomite and washed with DCM. The filtrate was concentrated in vacuo. The crude product was purified via flash chromatography on silica gel (petroleum ether: EtOAc = 8: 1 to 3: 1) to give **8** (425 mg, 51% yield) as a yellow solid. The product **8** was purified at >99% enantiopurity after recrystallization (in MeOH and Et_2O). m.p. 186-187 °C; $[\alpha]_D^{18}$ -294 (c 0.5, $CHCl_3$); **IR** (neat) ν 3310.0, 2931.3, 2369.5, 1662.4, 1543.4, 1420.4, 1370.3, 1264.6, 1140.3, 738.0 cm^{-1}; **^{1}H NMR** (600 MHz, $CDCl_3$) δ 8.10 (d, *J* = 8.3 Hz, 1H), 7.44 (d, *J* = 7.6 Hz, 1H), 7.33-7.29 (m, 1H), 7.26 (dd, *J* = 11.3, 3.5 Hz, 1H), 6.33 (dd, *J* = 6.0, 2.1 Hz, 1H), 5.35 (t, *J* = 7.5 Hz, 1H), 5.12 (d, *J* = 8.2

Hz, 1H), 4.97-4.89 (m, 1H), 4.63 (dd, J = 6.0, 1.2 Hz, 1H), 3.71 (s, 3H), 2.90 (td, J = 12.2, 3.6 Hz, 1H), 2.83 – 2.75 (m, 1H), 2.76 – 2.66 (m, 1H), 2.52-2.41 (m, 2H), 2.38-2.26 (m, 1H), 2.00-1.91 (m, 1H), 1.69 (s, 9H); **^{13}C NMR** (150 MHz, $CDCCl_3$) δ 170.9, 149.8, 141.7, 136.6, 134.6, 128.4, 124.7, 123.1, 118.2, 117.2, 115.7, 103.1, 100.4, 84.6, 57.1, 51.0, 45.3, 38.6, 36.2, 29.4, 28.2, 20.8; **HRMS (ESI)** calcd for $[M+Na]^+$ $C_{24}H_{28}N_2O_5Na$, m/z: 447.1890, found: 447.1891; Enantiomeric excess was 91% determined by HPLC (IF-3, Hexane/Isopropanol 90/10, flow rate = 1.0 mL/min, 260 nm): major isomer: tr = 12.88 min; minor isomer: tr = 14.45 min. The product **8** was purified at >99% enantiopurity after recrystallization (in MeOH and Et_2O).

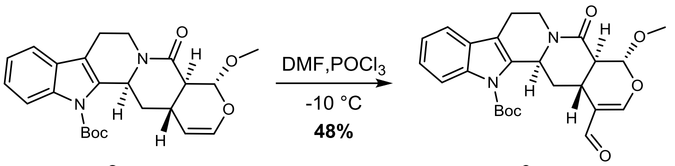

8 9

tert-butyl-(4*R*,4a*R*,13b*S*,14a*R*)-1-formyl-4-methoxy-5-oxo-4a,7,8,13b,14,14a-hexahydro-4H-indolo-[2,3-a]-pyrano-[3,4-g]-quinolizine-13(5H)-carboxylate (9)

$POCl_3$ (180 μL, 3 eq.) was added to DMF (15 mL) at 0 °C for 2 hours before cooling to -10 °C, and then compound **8** (273 mg, 1 eq.) was added to the system. The mixture was stirred at this temperature for 48 hours. Then, the reaction mixture was warmed to 0 °C, and the mixture was then quenched with saturated NH_4Cl aqueous solution and the mixture was extracted with EtOAc. Then the combined organic phase was washed with saturated brine six times, dried over Na_2SO_4 and concentrated in vacuo. The crude product was purified via flash chromatography on silica gel (petroleum ether: EtOAc = 5: 1 to 1: 1) to give **9** (116 mg, 48% yield based on recovered starting material) as an amorphous solid**,** and recovered starting material **8** (52.1 mg, 9% yield). $[\alpha]_D^{18}$ -240 (c 0.1, $CHCl_3$); **IR** (neat) ν 3403.2, 2925.3, 2372.9, 1729.4, 1664.9, 1457.4, 1311.1, 1120.4, 766.9, cm^{-1}; **^{1}H NMR** (400 MHz, $CDCl_3$) δ 9.21 (s, 1H), 8.05 (d, J = 8.2 Hz, 1H), 7.44 (d, J = 7.6 Hz, 1H), 7.30 (dd, J = 11.3, 4.2 Hz, 1H), 7.25 (dd, J = 7.2, 5.7 Hz, 2H), 5.34 (t, J = 7.6 Hz, 1H), 5.23 (d, J = 8.5 Hz, 1H), 4.90 (dd, J = 12.7, 3.6 Hz, 1H), 3.78 (s, 3H), 2.94 (td, J = 12.2, 3.6 Hz, 1H), 2.83 (d, J = 14.3 Hz, 1H), 2.70 (ddd, J = 22.1, 11.9, 6.2 Hz, 2H), 2.57 (ddd, J = 16.9, 8.2, 4.7 Hz, 2H), 2.45 (ddd, J = 14.0, 9.9, 7.2 Hz, 1H), 1.66 (s, 9H); **^{13}C NMR** (100 MHz, $CDCl_3$) δ 189.0, 170.1, 162.8, 149.9, 136.3, 134.3, 128.3, 124.6, 122.9, 120.8, 118.1, 116.7, 115.8, 103.0, 84.5, 57.9, 50.6, 43.9, 38.5, 33.9, 28.9, 28.2, 20.6; **HRMS (ESI)** calcd for $[M+Na]^+$ $C_{25}H_{28}N_2O_6Na$, m/z: 475.1840, found: 475.1843.

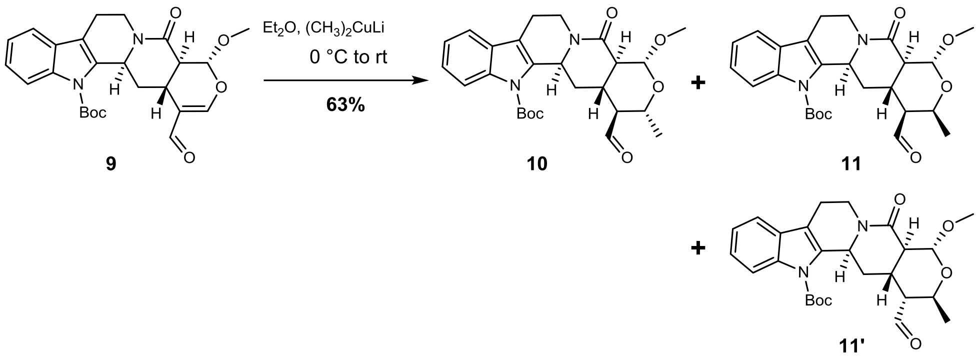

To a stirred mixture of CuI (82.6 mg, 2.0 eq.) in anhydrous Et_2O (3.0 mL) was added CH_3Li (1.3 M, 501 μL, 2.0 eq.) at 0 ºC. The mixture was stirred at 0 ºC for 5 minutes, and gave a colorless solution of $(CH_3)_2CuLi$ in Et_2O (0.58 M in Et_2O). To a stirred solution of compound **9** (97.7 mg, 1.0 eq.) in anhydrous Et_2O (3 mL) at 0 ºC was added above freshly prepared $(CH_3)_2CuLi$. Then the reaction mixture was warmed up to room temperature and stirred until the substrate disappeared via TLC detection (about 10 minutes). The mixture was then quenched with saturated NH_4Cl aqueous solution, and the mixture was extracted with EtOAc. Then the combined organic phase was washed with saturated brine, dried over anhydrous Na_2SO_4, and concentrated in vacuo. The crude product was purified via flash chromatography on silica gel (petroleum ether: EtOAc = 5: 1 to 2: 1) to give compound **10** (28.3 mg, 28% yield) as a colorless oil, compound **11** (25.5 mg, 25% yield) as a colorless oil, and compound **11'** (10.0 mg, 10% yield) as a colorless oil.

tert-butyl(1*S*,2*R*,4*R*,4a*R*,13b*S*,14a*S*)-1-formyl-4-methoxy-2-methyl-5-oxo-1,4a,5,7,8,13b,14,14a-octahydro-4H-indolo-[2,3-a]-pyrano-[3,4-g]-quinolizine-13(2H)-carboxylate (10)

$[\alpha]_D^{18}$ -230 (c 0.2, $CHCl_3$); **IR** (neat) ν 3430.8, 2926.2, 1729.5, 1663.4, 1456.9, 1370.8, 1310.6, 1140.9, 737.9 cm^{-1}; **^{1}H NMR** (600 MHz, $CDCl_3$) δ 9.60 (d, *J* = 3.5 Hz, 1H), 8.04 (d, *J* = 8.3 Hz, 1H), 7.44 (d, *J* = 7.5 Hz, 1H), 7.33-7.29 (m, 1H), 7.27 (s, 1H), 7.25 (d, *J* = 7.3 Hz, 1H), 5.37-5.27 (m, 1H), 4.96-4.86 (m, 1H), 4.70 (d, *J* = 8.1 Hz, 1H), 3.69 (s, 3H), 2.89 (td, *J* = 12.3, 3.5 Hz, 1H), 2.86-2.79 (m, 1H), 2.75-2.65 (m, 1H), 2.33 (tdd, *J* = 17.5, 12.8, 4.9 Hz, 3H), 2.27-2.20 (m, 1H), 1.83 (dt, *J* = 13.3, 8.9 Hz, 1H), 1.70 (s, 9H), 1.35 (d, *J* = 6.2 Hz, 3H); **^{13}C NMR** (150 MHz, $CDCl_3$) δ 201.2, 170.7, 149.9, 136.3, 134.1, 128.3, 124.7, 123.1, 118.2, 117.0, 115.8, 101.8, 84.6, 70.0, 61.8, 57.1, 50.3, 45.5, 38.2, 34.9, 33.2, 28.3, 20.6, 19.8; **HRMS (ESI)** calcd for $[M+H]^+$ $C_{26}H_{33}N_2O_6$, m/z: 469.2333, found: 469.2333.

tert-butyl-(1*S*,2*S*,4*R*,4a*R*,13b*S*,14a*S*)-1-formyl-4-methoxy-2-methyl-5-oxo-1,4a,5,7,8,13b,14,14a-octahydro-4H-indolo-[2,3-a]-pyrano-[3,4-g]-quinolizine-13(2H)-carboxylate (11)

$[\alpha]_D^{18}$ -168 (c 0.5, $CHCl_3$); **IR** (neat) ν 3429.7, 2930.9, 1727.1, 1664.6, 1310.8 1140.5, 737.6 cm^{-1}; **^{1}H NMR** (400 MHz, $CDCl_3$) δ 9.70 (d, *J* = 3.8 Hz, 1H), 7.99 (d, *J* = 8.2 Hz, 1H), 7.44 (d, *J* = 7.2 Hz, 1H), 7.34-7.28 (m, 1H), 7.26 (t, *J* = 3.4 Hz, 1H), 5.33 (d, *J* = 8.9 Hz, 1H), 5.14 (d, *J* = 7.1 Hz, 1H), 4.91 (dd,

J = 12.7, 3.3 Hz, 1H), 4.38 (p, J = 6.7 Hz, 1H), 3.53 (s, 3H), 2.93 (td, J = 12.1, 3.9 Hz, 1H), 2.85-2.66 (m, 2H), 2.58-2.47 (m, 1H), 2.43 (dd, J = 13.3, 7.2 Hz, 1H), 2.21 (ddt, J = 19.7, 11.3, 6.1 Hz, 2H), 1.98-1.83 (m, 1H), 1.69 (s, 9H), 1.32 (d, J = 6.9 Hz, 3H); **^{13}C NMR** (100 MHz, $CDCl_3$) δ 202.2, 170.6, 150.1, 136.2, 134.1, 128.4, 124.6, 123.0, 118.2, 117.5, 115.8, 99.4, 84.6, 64.3, 59.2, 55.6, 51.0, 44.6, 38.9, 35.6, 28.2, 26.8, 20.6, 17.4; **HRMS (ESI)** calcd for $[M+H]^+$ $C_{26}H_{33}N_2O_6$, m/z: 469.2333, found: 469.2338.

tert-butyl-(1*R*,2*S*,4*R*,4a*R*,13b*S*,14a*S*)-1-formyl-4-methoxy-2-methyl-5-oxo-1,4a,5,7,8,13b,14,14a-octahydro-4H-indolo-[2,3-a]-pyrano-[3,4-g]-quinolizine-13(2H)-carboxylate (11')

$[\alpha]_D^{18}$ -240 (c 0.1, $CHCl_3$); **IR** (neat) ν 2926.7, 1719.4, 1661.7, 1371.1, 1315.1, 1263.7, 1139.5, 1045.2, 739.6 cm^{-1}; **^{1}H NMR** (600 MHz, $CDCl_3$) δ 10.10 (d, J = 3.8 Hz, 1H), 7.98 (d, J = 8.2 Hz, 1H), 7.45 (d, J = 7.5 Hz, 1H), 7.31 (t, J = 7.6 Hz, 1H), 7.28 (s, 1H), 5.39 (d, J = 5.1 Hz, 1H), 5.21 (d, J = 6.3 Hz, 1H), 4.94 (dd, J = 12.7, 4.3 Hz, 1H), 4.30 (p, J = 6.4 Hz, 1H), 3.52 (s, 3H), 2.98-2.87 (m, 1H), 2.82 (d, J = 15.4 Hz, 1H), 2.74 (dd, J = 19.7, 7.6 Hz, 1H), 2.67 (dd, J = 13.9, 6.3 Hz, 1H), 2.56-2.42 (m, 2H), 2.24 (dt, J = 20.5, 6.6 Hz, 1H), 1.86 (dt, J = 13.9, 9.8 Hz, 1H), 1.70 (s, 9H), 1.27 (d, J = 6.3 Hz, 3H); **^{13}C NMR** (150 MHz, $cdcl_3$) δ 202.3, 170.5, 150.2, 136.3, 134.2, 128.5, 124.7, 123.1, 118.3, 117.8, 115.7, 99.6, 85.0, 62.4, 58.1, 55.6, 51.8, 44.5, 39.1, 32.8, 28.2, 27.9, 20.8, 20.6; **HRMS (ESI)** calcd for [M+H]+ $C_{26}H_{33}N_2O_6$, m/z: 469.2333, found: 469.2324.

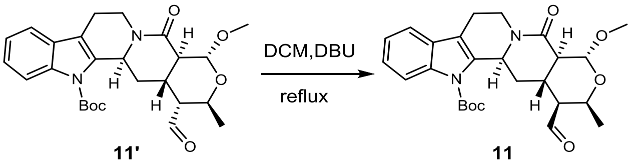

11' 11

To a stirred mixture of **11'** (23.4 mg) in anhydrous DCM (5 mL) was added DBU at room temperature. The reaction solution was refluxed and stirred until the substrate disappeared via TLC detection. The reaction mixture was cooled to room temperature, diluted with EtOAc and saturated NH_4Cl aqueous solution, extracted with EtOAc. The combined organic extract was washed with saturated $NaHCO_3$ aqueous solution and brine, dried with Na_2SO_4 and concentrated in vacuo. The crude product was purified via flash chromatography on silica gel (petroleum ether: EtOAc = 3: 1) to give compound **11** (19.9 mg, 85% yield).

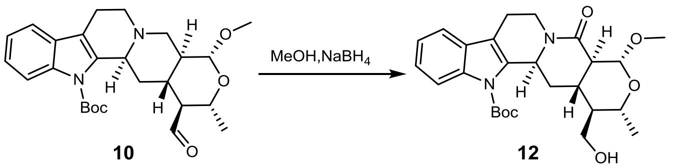

10 12

To a stirred mixture of **10** (52.8 mg, 1.0 eq.) in anhydrous MeOH (2 mL) was added $NaBH_4$ (7.1 mg,

2.0 eq.) at 0 °C. The mixture was stirred at this temperature until the substrate disappeared via TLC detection (about 5 minutes). the mixture was then quenched with saturated $NaHCO_3$ aqueous solution. The resulting solution was concentrated in vacuo and the mixture was extracted with EtOAc (3×80 mL), and the combined organic phase was washed with brine, dried over anhydrous Na_2SO_4, and concentrated in vacuo. The crude product was purified via flash chromatography on silica gel (petroleum ether: EtOAc: = 1: 2) to give compound **12** (45.4 mg, 86% yield). $[\alpha]_D^{23}$ -330 (c 0.1, $CHCl_3$); **IR** (neat) ν 3402.4, 2924.5, 2371.1, 1728.2, 1655.4, 1458.6, 1368.8, 1261.9, 1142.2, 745.2 cm^{-1}; **^{1}H NMR** (600 MHz, $CDCl_3$) δ 8.09 (d, *J* = 8.3 Hz, 1H), 7.48-7.41 (m, 1H), 7.33-7.28 (m, 1H), 7.27-7.24 (m, 1H), 5.30 (t, *J* = 7.9 Hz, 1H), 4.89 (ddd, *J* = 12.7, 5.0, 1.4 Hz, 1H), 4.66 (d, *J* = 8.2 Hz, 1H), 3.76-3.71 (m, 1H), 3.67 (s, 3H), 3.61 (ddt, *J* = 15.7, 12.3, 4.8 Hz, 2H), 2.87 (td, *J* = 12.3, 3.6 Hz, 1H), 2.84-2.77 (m, 1H), 2.71-2.63 (m, 1H), 2.40-2.29 (m, 2H), 2.12-2.01 (m, 1H), 1.92 (dt, *J* = 13.2, 9.3 Hz, 1H), 1.68 (s, 9H), 1.35 (d, *J* = 6.2 Hz, 3H), 1.30-1.22 (m, 2H); **^{13}C NMR** (150 MHz, $CDCl_3$) δ 171.9, 149.9, 136.6, 134.7, 128.5, 124.6, 123.1, 118.2, 116.9, 115.8, 101.8, 84.5, 71.6, 60.0, 56.9, 50.2, 47.1, 38.0, 35.4, 33.5, 28.3, 28.2, 20.7, 19.2; **HRMS (ESI)** calcd for $[M+Na]^+$ $C_{26}H_{34}N_2O_6Na$, m/z: 493.2309, found: 493.2320.

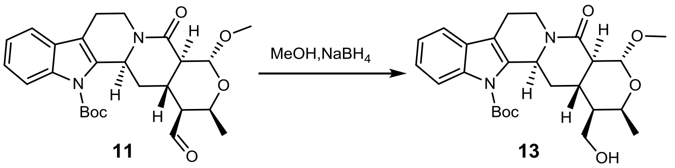

13 (36.6 mg) was prepared according to the same procedure as **12** from **11** (43.9 mg) as an amorphous solid in 83 % yield.

$[\alpha]_D^{23}$ -220 (c 0.1, $CHCl_3$) **IR** (neat) ν 3398.6, 2923.9, 2372.0, 1727.6, 1655.5, 1458.8, 1367.6, 1261.1, 1118.7, 761.5 cm^{-1}; **^{1}H NMR** (600 MHz, $CDCl_3$) δ 8.04 (d, *J* = 8.2 Hz, 1H), 7.45 (d, *J* = 7.4 Hz, 1H), 7.31 (t, *J* = 7.6 Hz, 1H), 7.28-7.25 (m, 1H), 5.31 (s, 1H), 5.05 (d, *J* = 7.4 Hz, 1H), 4.91 (dd, *J* = 12.6, 4.4 Hz, 1H), 4.3-4.24 (m, 1H), 3.73 (d, *J* = 10.8 Hz, 1H), 3.61 (dt, *J* = 11.0, 5.6 Hz, 1H), 3.54 (s, 3H), 2.95-2.85 (m, 1H), 2.81 (d, *J* = 14.2 Hz, 1H), 2.78-2.71 (m, 1H), 2.46 (dd, *J* = 13.6, 7.4 Hz, 1H), 2.36-2.26 (m, 1H), 2.06-1.96 (m, 1H), 1.87 (dt, *J* = 15.9, 8.5 Hz, 1H), 1.81-1.74 (m, 1H), 1.70 (s, 9H), 1.43 (s, 1H), 1.30 (d, *J* = 6.8 Hz, 3H); **^{13}C NMR** (150 MHz, $CDCl_3$) δ 171.5, 150.1, 136.5, 134.7, 128.6, 124.6, 123.1, 118.2, 117.5, 115.8, 99.4, 84.5, 66.2, 61.8, 55.5, 51.1, 47.4, 45.7, 38.8, 37.0, 29.7, 28.3, 20.8, 16.2; **HRMS (ESI)** calcd for $[M+H]^+$ $C_{26}H_{35}N_2O_6$, m/z: 471.2490, found: 471.2491.

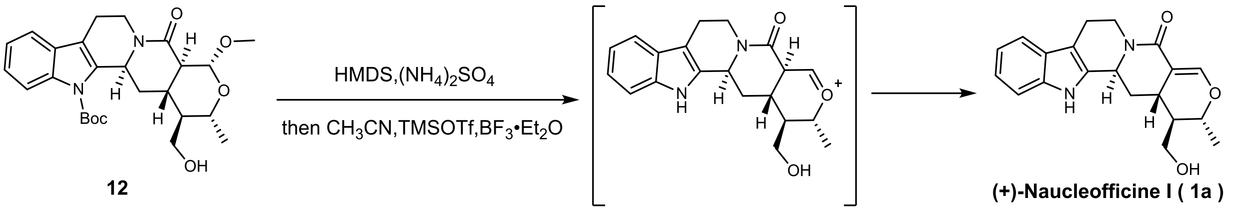

To a stirred mixture of **12 (**45.4 mg, 1.0 eq**)** in anhydrous HMDS (1,1,1,3,3,3-Hexamethyldisilazane) (0.5 mL) was added $(NH_4)_2SO_4$ (catalytic amount) at room temperature. The reaction solution was refluxed and stirred until the substrate disappeared via TLC detection (about 1 hour). The reaction mixture was cooled to room temperature and the solvent was evaporated in vacuo. The residue was dissolved in anhydrous CH_3CN (1 mL) and trimethylsilyl trifluoromethansulfonate (53 μL, 3.0 eq.) was added to this solution followed by adding boron trifluoride diethyl etherate (37 μL, 3.0 eq.) at 0 °C. And then the reaction solution was warmed to room temperature and stirred 10 hours. The mixture was then quenched with saturated $NaHCO_3$ aqueous solution, and the mixture was extracted with EtOAc. Then the combined organic phase was washed with saturated brine, dried over anhydrous Na_2SO_4, and concentrated in vacuo. The crude product was purified via flash chromatography (petroleum ether: EtOAc = 1:1 to EtOAc) to give compound **(+) 1a** (26.5 mg, 81%).

$[\alpha]_D^{20}$ +80 (c 0.2, CH_3OH); **IR** (neat) ν 3346.5, 2925.5, 2371.7, 1847.7, 1738.5, 1655.2, 1579.9, 1449.7, 1240.8, 1107.3, 1047.6 cm^{-1}; **^{1}H NMR** (600 MHz, DMSO-*d6*) δ 10.96 (s, 1H), 7.37 (d, *J* = 7.8 Hz, 1H), 7.34 (d, *J* = 8.1 Hz, 1H), 7.28 (d, *J* = 1.5 Hz, 1H), 7.06 (t, *J* = 7.5 Hz, 1H), 6.97 (t, *J* = 7.3 Hz, 1H), 5.01 (d, *J* = 4.5 Hz, 1H), 4.81 (dd, *J* = 12.6, 5.7 Hz, 1H), 3.78 (dq, *J* = 12.5, 6.2 Hz, 1H), 3.71 (dd, *J* = 11.4, 2.5 Hz, 1H), 3.65 (dd, *J* = 11.4, 3.4 Hz, 1H), 2.95 (ddd, *J* = 32.9, 16.4, 8.4 Hz, 2H), 2.76 (tdd, *J* = 9.5, 5.9, 3.4 Hz, 1H), 2.59 (dd, *J* = 15.1, 4.4 Hz, 1H), 2.16 (t, *J* = 11.6 Hz, 1H), 1.65 (td, *J* = 13.4, 5.9 Hz, 1H), 1.32 (d, *J* = 6.3 Hz, 3H), 1.17-1.13 (m, 1H); **^{13}C NMR** (150 MHz, DMSO-*d6*) δ 164.1, 149.6, 135.7, 134.5, 126.9, 120.8, 118.5, 117.5, 111.2, 109.5, 108.5, 74.3, 57.8, 53.1, 44.6, 42.3, 28.8, 27.4, 20.7, 18.5; **HRMS (ESI)** calcd for $[M+Na]^+$ $C_{20}H_{22}N_2O_3Na$, m/z: 361.1523, found: 361.1521; Enantiomeric excess was >99% determined by HPLC (IA-3, Hexane/Isopropanol 90/10, flow rate = 1.0 mL/min, 245 nm): major isomer: tr = 19.78 min.

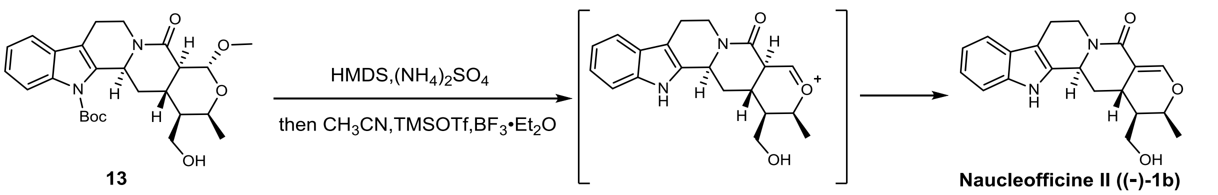

(–)-1b (17.1 mg) was prepared according to the same procedure as **(+)-1a** from **13** (29.7 mg) as an amorphous solid in 80 % yield. $[\alpha]_D^{21}$ -15 (c 0.2, CH_3OH); **IR** (neat) ν 3349.9, 2926.6, 2371.0, 1737.5, 1648.1, 1580.0, 1466.7, 1373.5, 1241.1, 1153.2, 1047.1, 745.1 cm^{-1}; **^{1}H NMR** (600 MHz, DMSO-*d6*) δ 11.00 (s, 1H), 7.35 (dd, *J* = 17.4, 8.0 Hz, 2H), 7.23 (d, *J* = 1.7 Hz, 1H), 7.06 (t, *J* = 7.5 Hz, 1H), 6.97 (t,

J = 7.0 Hz, 1H), 5.01 (s, 1H), 4.80 (dd, *J* = 12.7, 5.6 Hz, 1H), 4.71 (t, *J* = 5.2 Hz, 1H), 4.49 (dd, *J* = 6.6, 4.2 Hz, 1H), 3.85 (dd, *J* = 11.1, 4.4 Hz, 1H), 3.26-3.19 (m, 1H), 2.95 (td, *J* = 12.4, 4.7 Hz, 1H), 2.84 (d, *J* = 13.1 Hz, 1H), 2.75 (d, *J* = 9.5 Hz, 1H), 2.58 (dd, *J* = 15.4, 4.7 Hz, 1H), 1.82 (d, *J* = 11.9 Hz, 1H), 1.80-1.72 (m, 2H), 0.95 (d, *J* = 6.6 Hz, 3H); **^{13}C NMR** (150 MHz, DMSO-*d*6) δ 163.8, 148.5, 135.8, 134.4, 127.0, 120.9, 118.7, 117.6, 111.3, 108.6, 107.6, 71.9, 60.0, 53.0, 42.7, 42.4, 28.7, 24.9, 20.8, 13.7; **HRMS (ESI)** calcd for $[M+Na]^+$ $C_{20}H_{22}N_2O_3Na$, m/z: 361.1523, found: 361.1520; Enantiomeric excess was >99% determined by HPLC (IA-3, Hexane/Isopropanol 90/10, flow rate = 1.0 mL/min, 215 nm): major isomer: tr = 29.59 min.

Supplementary Table 3. Comparison of the ^{13}C NMR (DMSO-*d*6) data of the natural[6] and synthetic

1a

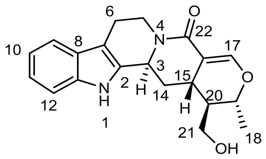

(+)-Naucleofficine I (1a)

NO.	**Natural** ^{13}C NMR (100 MHz, DMSO-d_6)	**Synthetic** ^{13}C NMR (150 MHz, DMSO-d_6)	Δ δ (ppm)
1	-	-	-
2	134.5	134.5	0
3	53.1	53.1	0
4	-	-	-
5	42.3	42.3	0
6	20.7	20.7	0
7	108.5	108.5	0
8	126.9	126.9	0
9	117.5	117.5	0
10	118.6	118.5	-0.1
11	120.8	120.8	0
12	111.3	111.2	-0.1
13	135.7	135.7	0
14	28.8	28.8	0
15	27.4	27.4	0
16	109.5	109.5	0
17	149.6	149.6	0
18	18.5	18.5	0
19	74.3	74.3	0
20	44.6	44.6	0
21	57.8	57.8	0
22	164.2	164.1	- 0.1

Supplementary Table 4. Comparison of the ^{13}C NMR (DMSO-*d*6) data of the natural[6] and synthetic **1b**

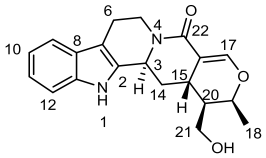

(-)-Naucleofficine I (1b)

NO.	**Natural** ^{13}C NMR (100 MHz, DMSO-d_6)	**Synthetic** ^{13}C NMR (150 MHz, DMSO-d_6)	Δ δ (ppm)
1	-	-	-
2	134.4	134.4	0
3	53.0	53.0	0
4	-	-	-
5	42.4	42.4	0
6	20.8	20.8	0
7	108.6	108.6	0
8	127.0	127.0	0
9	117.6	117.6	0
10	118.7	118.7	0
11	120.9	120.9	0
12	111.3	111.3	0
13	135.7	135.8	+ 0.1
14	28.6	28.7	+ 0.1
15	24.8	24.9	+ 0.1
16	107.5	107.6	+ 0.1
17	148.5	148.5	0
18	13.6	13.7	+ 0.1
19	71.9	71.9	0
20	42.7	42.7	0
21	60.0	60.0	0
22	163.8	163.8	0

X-Ray Crystallographic Data:

Supplementary Table 5. Crystal data and structure refinement for (-)-Cat 1 (CCDC 1875257).

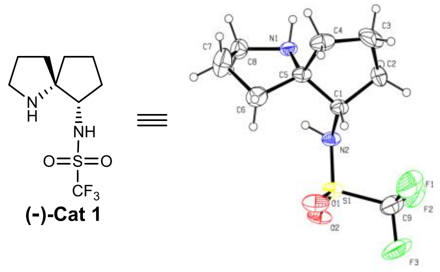

Identification code	(-)-Cat 1
Empirical formula	$C_9H_{15}F_3N_2O_2S$
Formula weight	272.29
Temperature/K	293.36(10)
Crystal system	triclinic
Space group	P1
a/Å	8.2899(9)
b/Å	10.0757(9)
c/Å	15.7006(13)
α/°	98.740(7)
β/°	90.167(8)
γ/°	112.200(9)
Volume/Å^3	1197.5(2)
Z	4
ρ_{calc}g/cm^3	1.510
μ/mm^{-1}	0.302
F(000)	568.0
Crystal size/mm^3	0.22 × 0.17 × 0.14
Radiation	MoKα (λ = 0.71073)
2Θ range for data collection/°	7.234 to 52.044
Index ranges	$-10 \le h \le 9$, $-11 \le k \le 12$, $-19 \le l \le 19$
Reflections collected	8319
Independent reflections	6204 [R_{int} = 0.0331, R_{sigma} = 0.0721]
Data/restraints/parameters	6204/110/640
Goodness-of-fit on F^2	1.029
Final R indexes [I>=2σ (I)]	R_1 = 0.0670, wR_2 = 0.1570
Final R indexes [all data]	R_1 = 0.0898, wR_2 = 0.1808
Largest diff. peak/hole / e Å^{-3}	0.48/-0.38
Flack parameter	0.06(8)

Supplementary Table 6. Crystal data and structure refinement for 8 (CCDC 1875256)

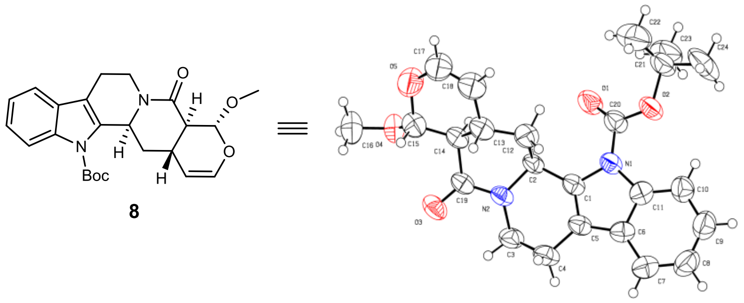

Identification code	Compound **8**
Empirical formula	$C_{24}H_{28}N_2O_5$
Formula weight	424.50
Temperature/K	295.28(10)
Crystal system	orthorhombic
Space group	$P2_12_12_1$
a/Å	9.6529(3)
b/Å	11.7434(3)
c/Å	40.1577(14)
α/°	90
β/°	90
γ/°	90
Volume/Å^3	4552.2(3)
Z	4
ρ_{calc}g/cm^3	1.2387
μ/mm^{-1}	0.710
F(000)	1814.0
Crystal size/mm^3	0.16 × 0.15 × 0.12
Radiation	Cu Kα (λ = 1.54184)
2Θ range for data collection/°	7.84 to 133.18
Index ranges	$-11 \leq h \leq 10$, $-14 \leq k \leq 13$, $-48 \leq l \leq 46$
Reflections collected	16550
Independent reflections	7886 [R_{int} = 0.0403, R_{sigma} = 0.0556]
Data/restraints/parameters	7886/0/567
Goodness-of-fit on F^2	1.034
Final R indexes [I>=2σ (I)]	R_1 = 0.0500, wR_2 = 0.1219
Final R indexes [all data]	R_1 = 0.0624, wR_2 = 0.1343
Largest diff. peak/hole / e Å^{-3}	0.18/-0.20
Flack parameter	0.0(2)

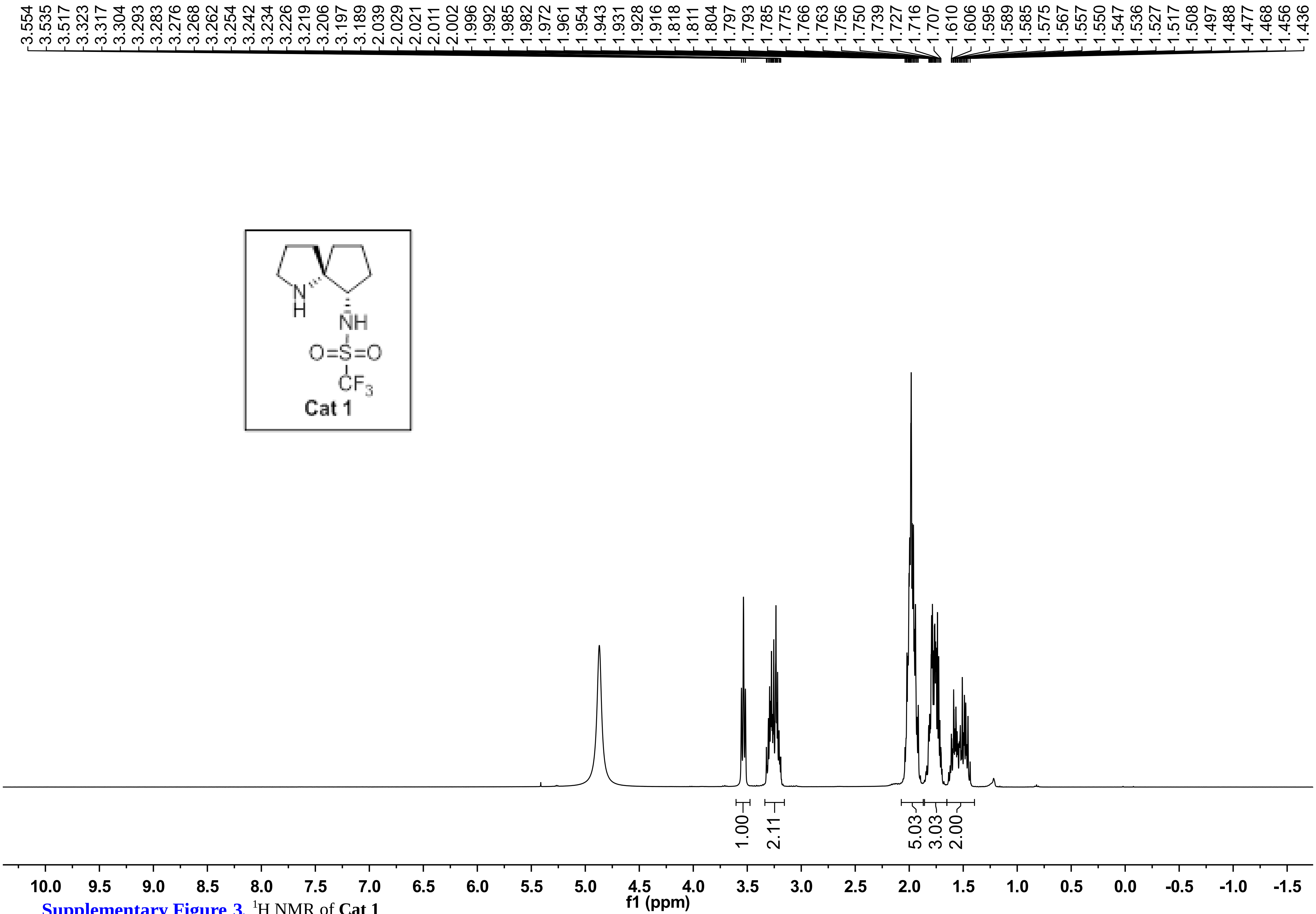

Supplementary Figure 3. ^{1}H NMR of **Cat 1**

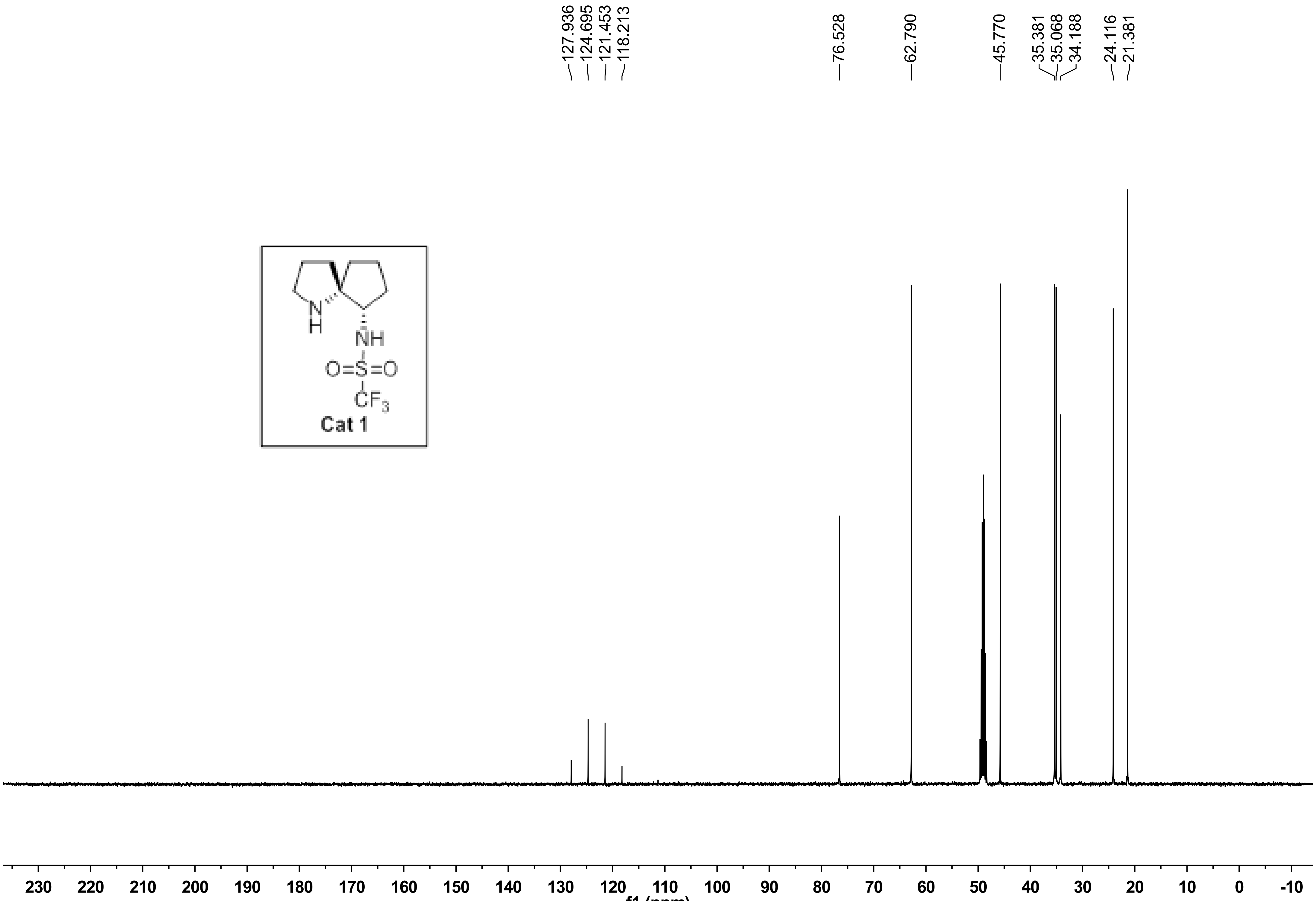

Supplementary Figure 4. ^{13}C NMR of **Cat 1**

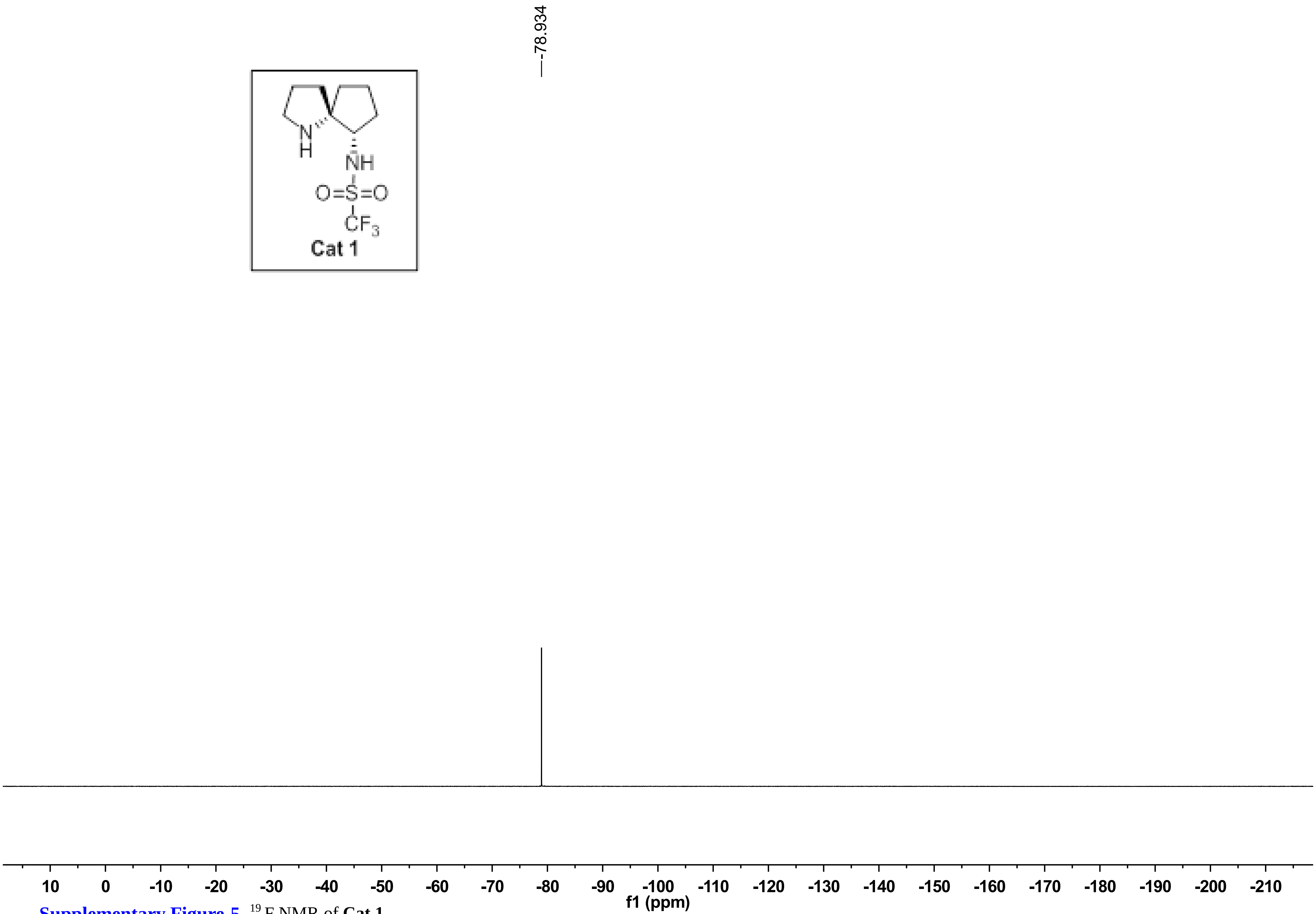

Supplementary Figure 5. ^{19}F NMR of **Cat 1**

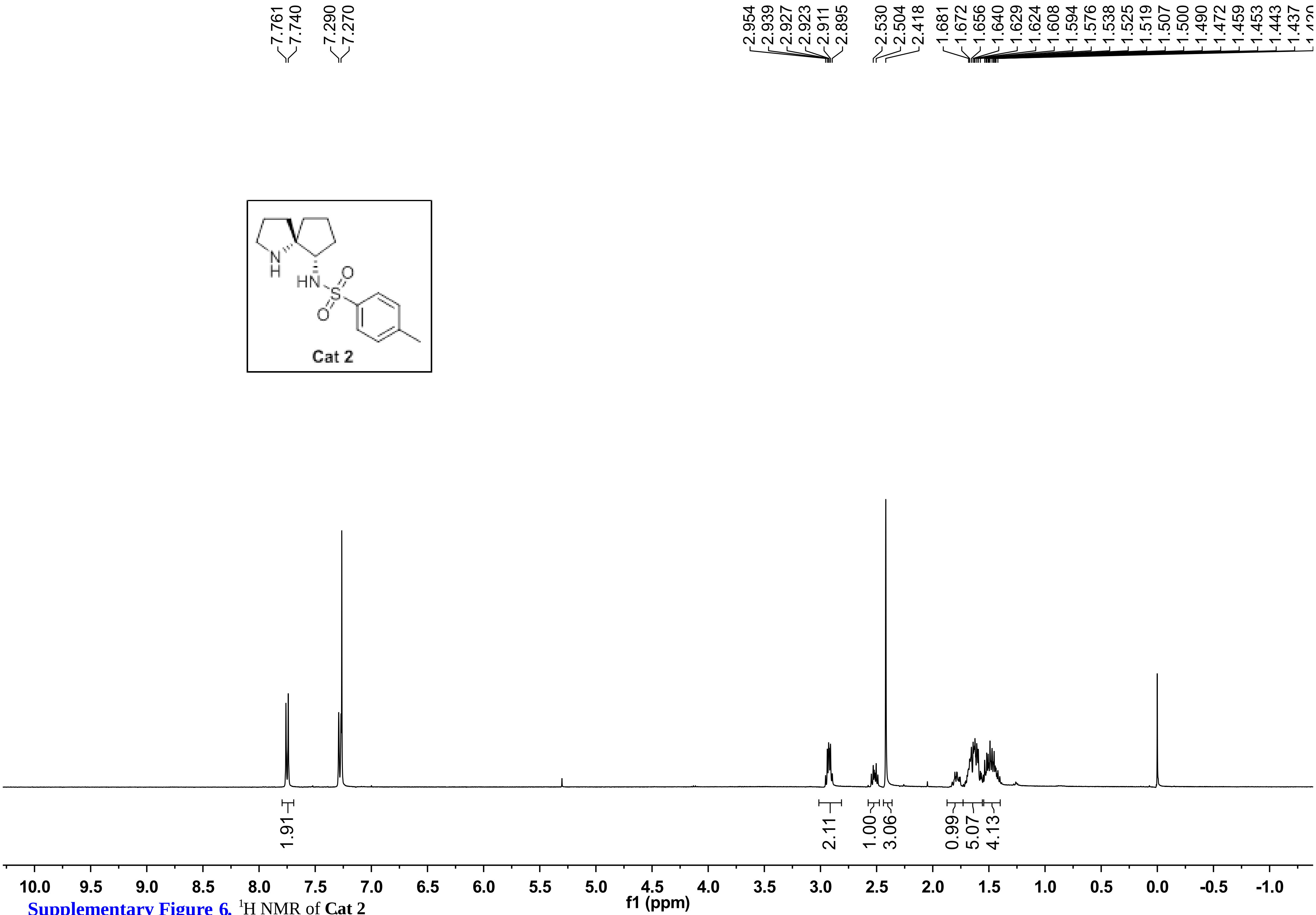

Supplementary Figure 6. ^{1}H NMR of **Cat 2**

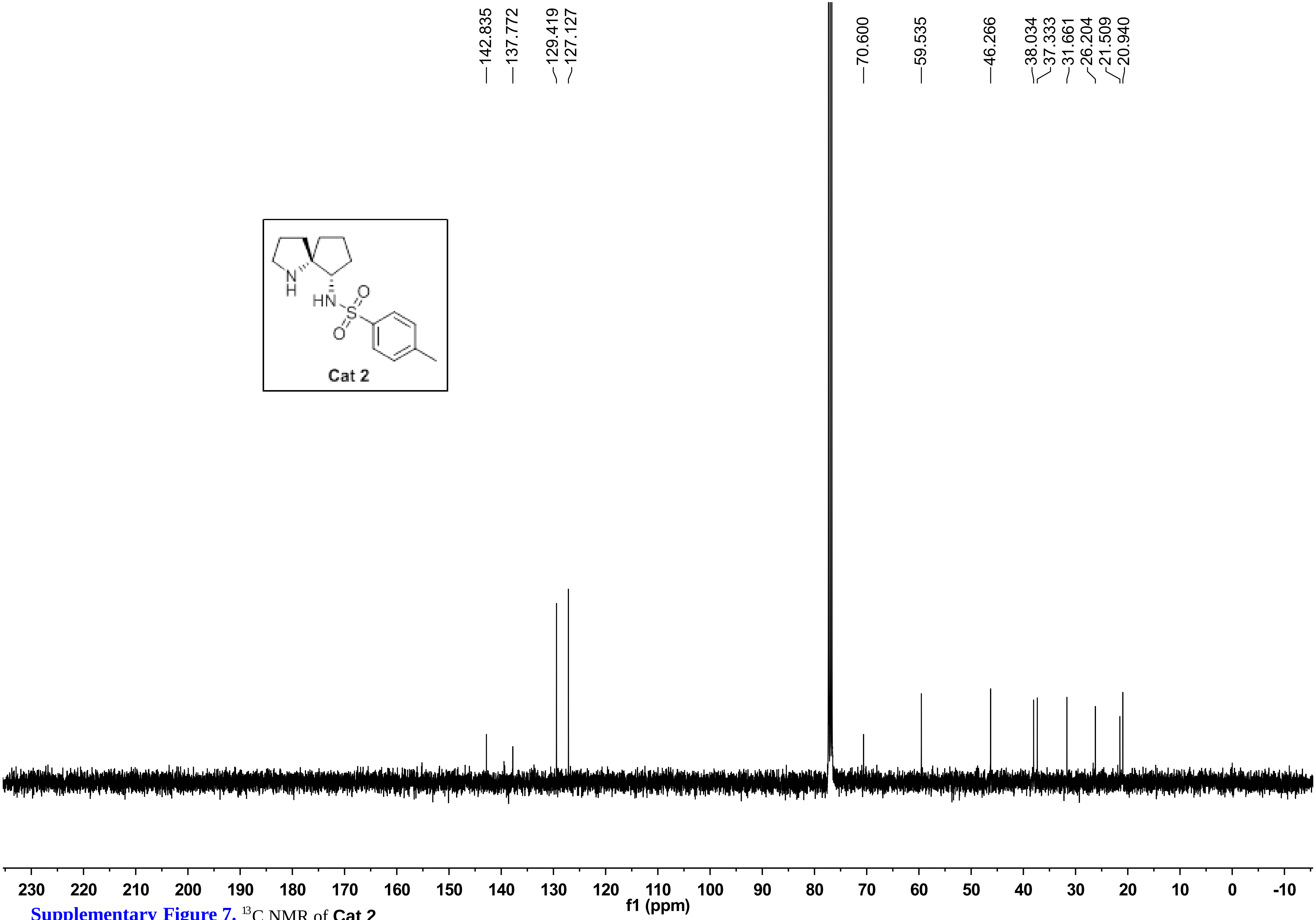

Supplementary Figure 7. ^{13}C NMR of **Cat 2**

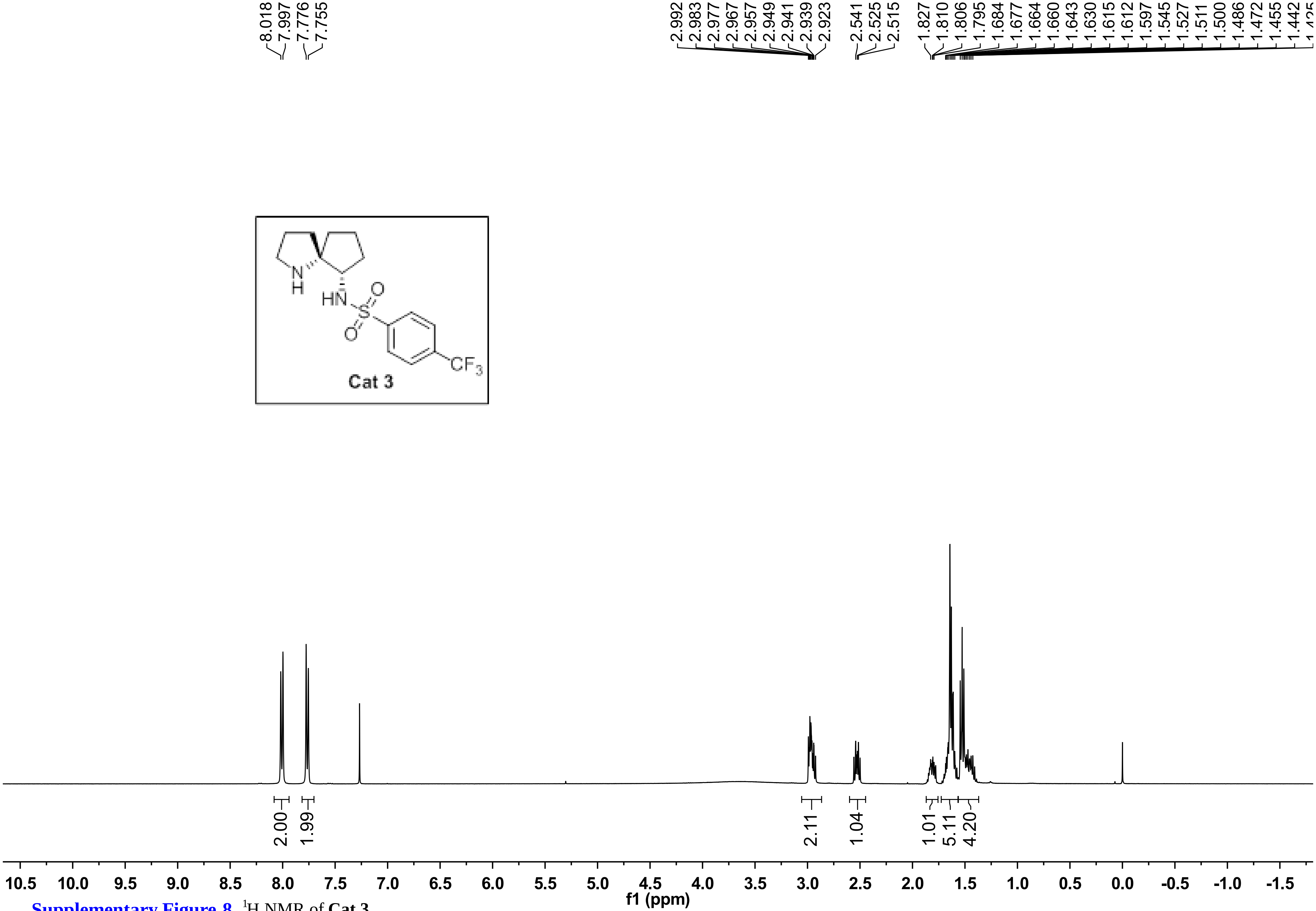

Supplementary Figure 8. ^{1}H NMR of **Cat 3**

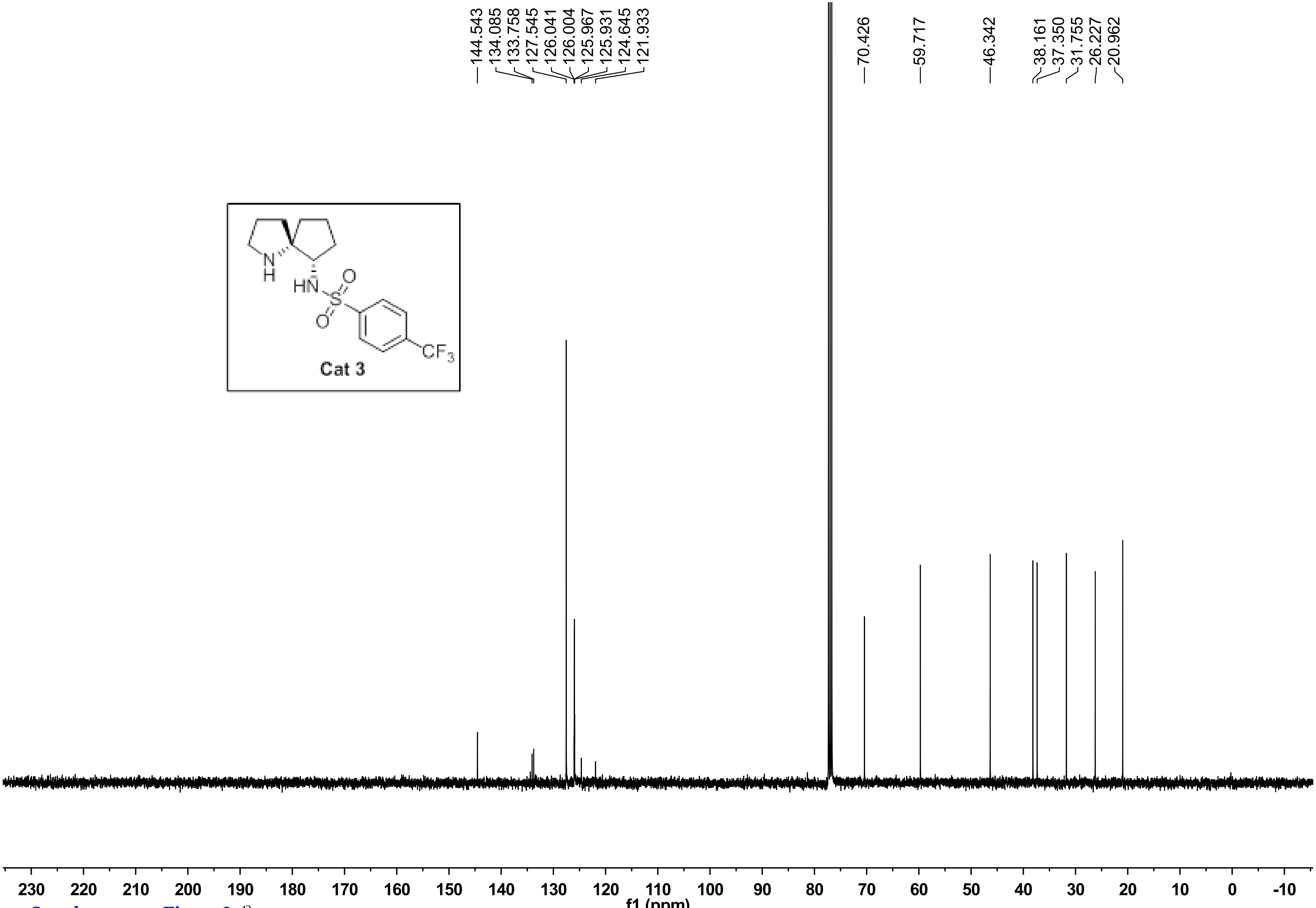

Supplementary Figure 9. ^{13}C NMR of **Cat 3**

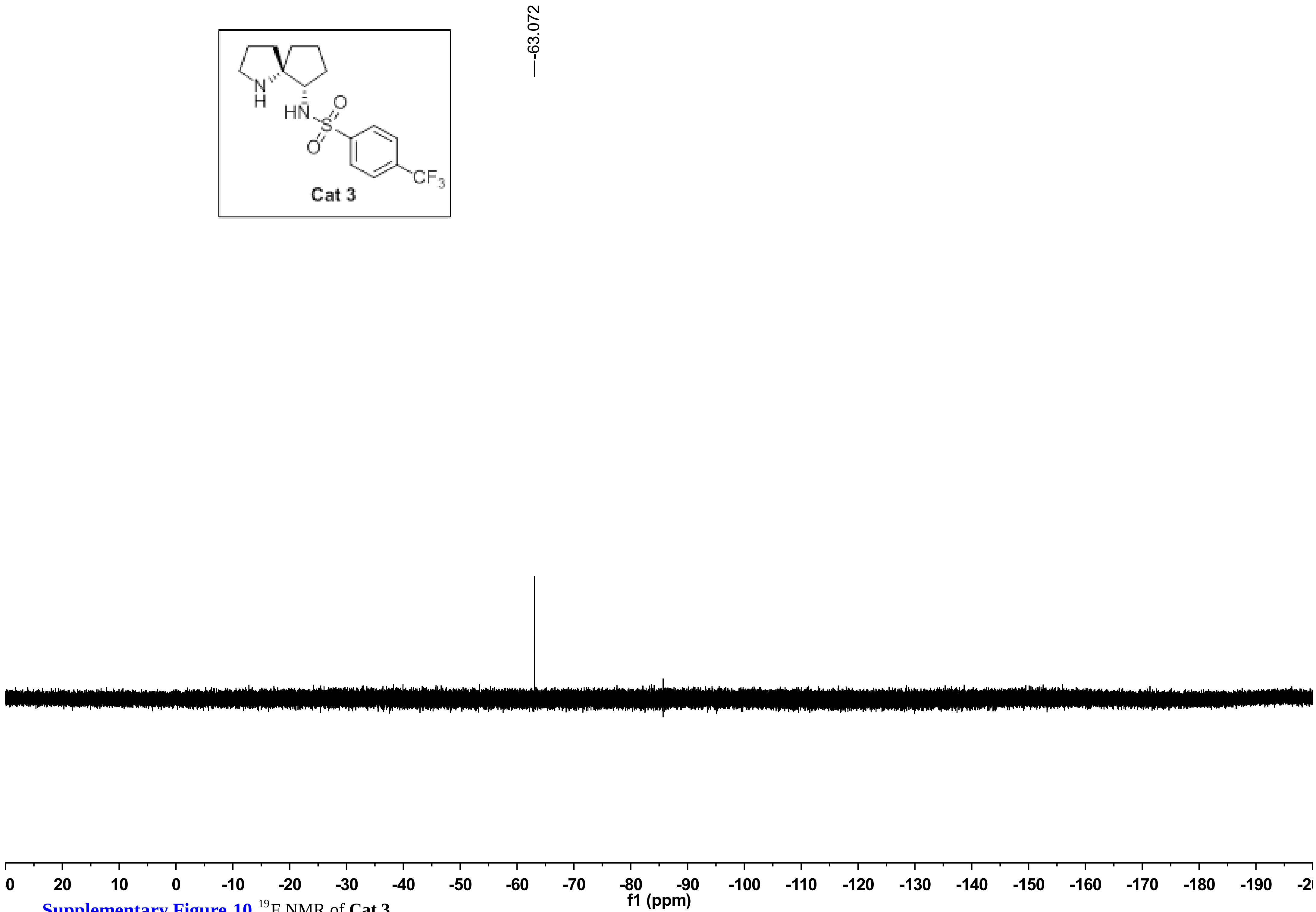

Supplementary Figure 10. ^{19}F NMR of **Cat 3**

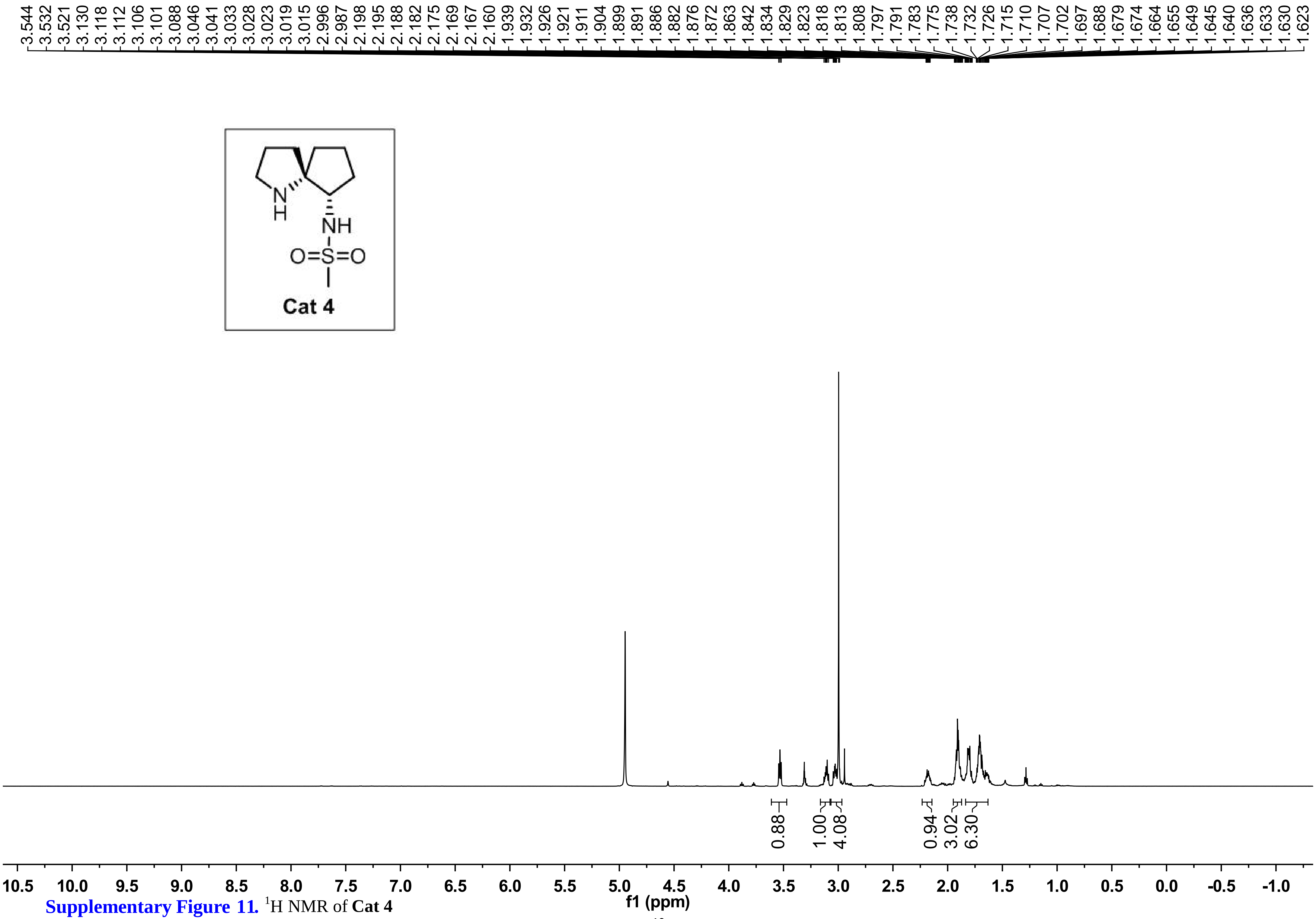

Supplementary Figure 11. ^{1}H NMR of **Cat 4**

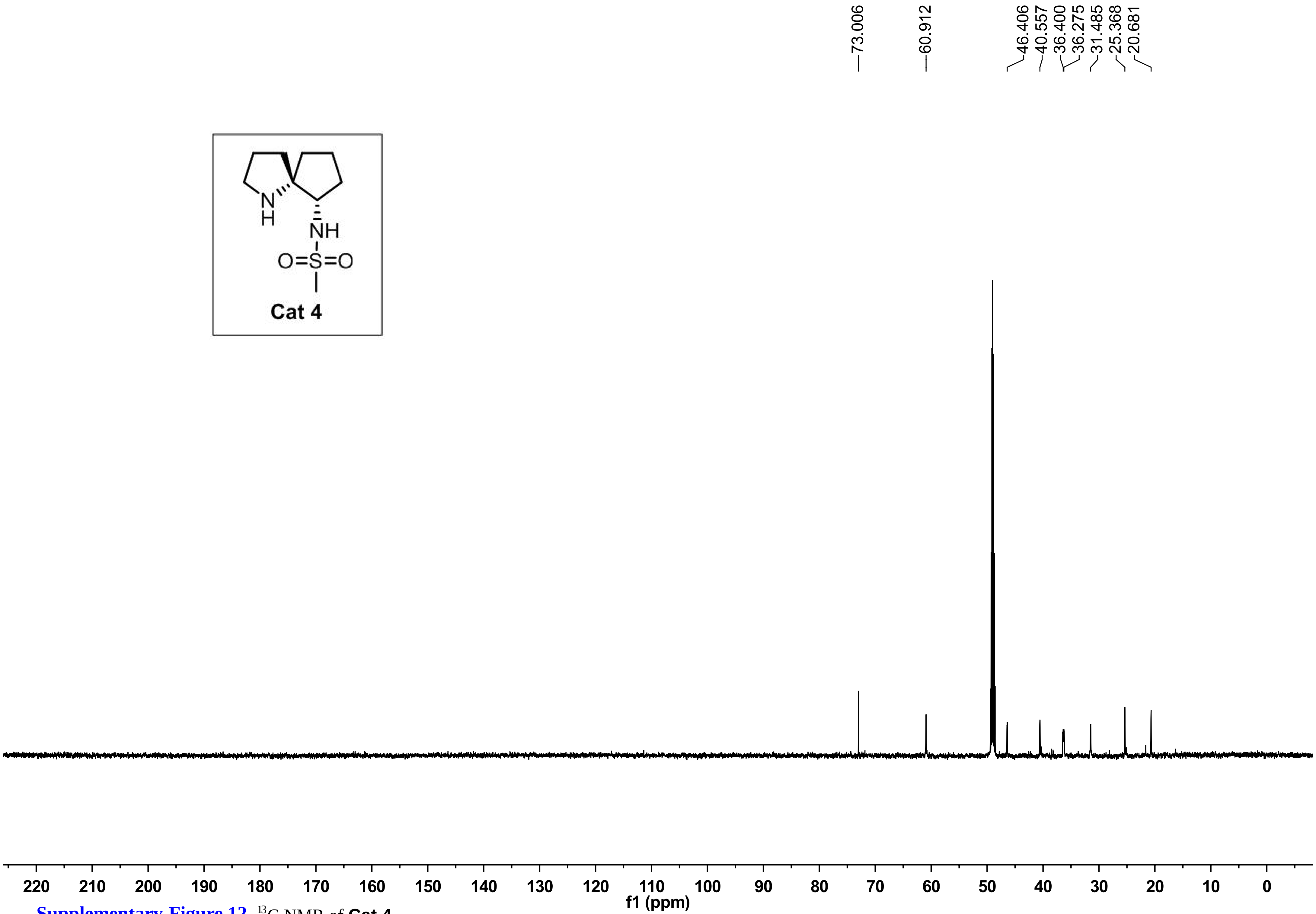

Supplementary Figure 12. ^{13}C NMR of **Cat 4**

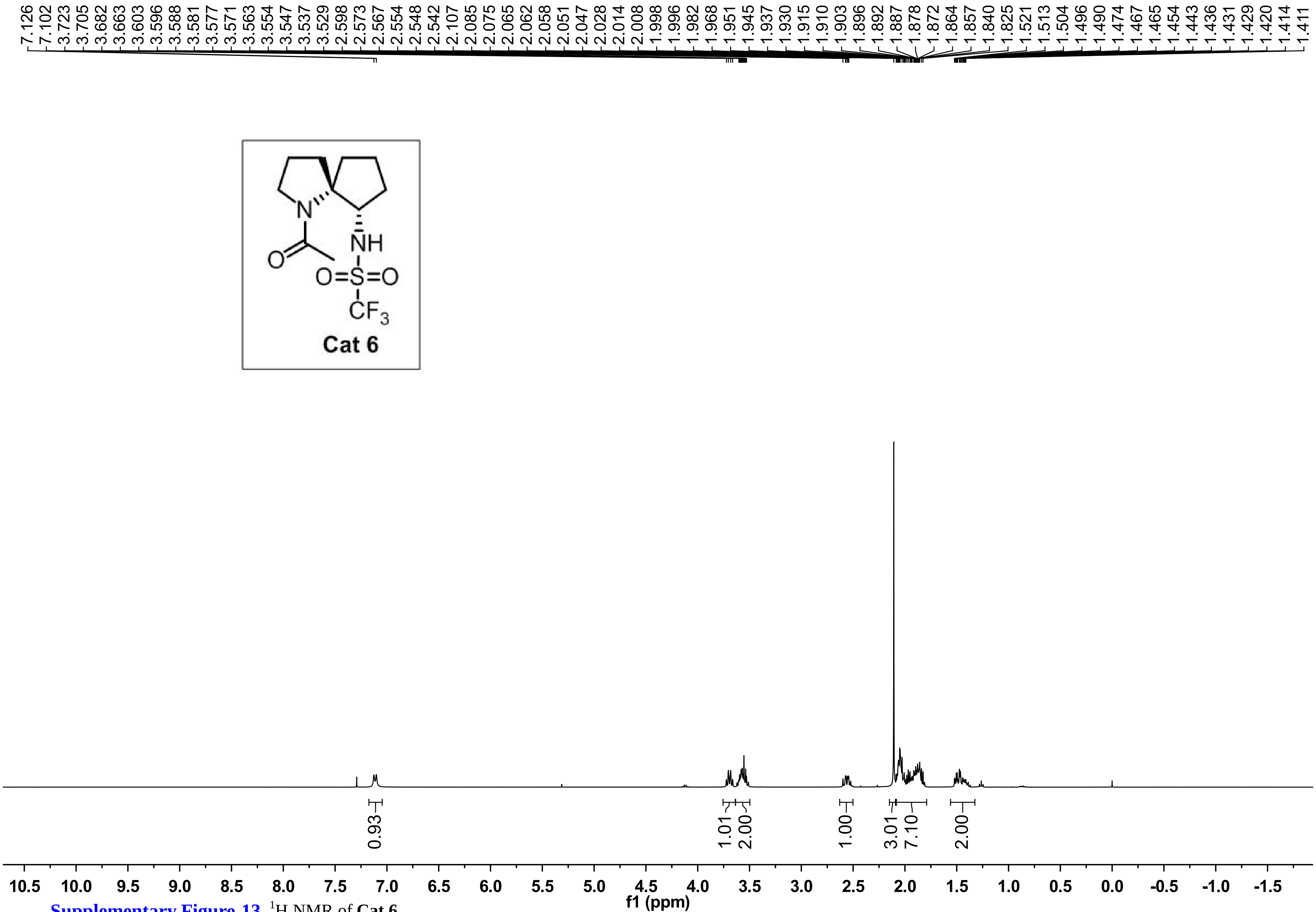

Supplementary Figure 13. ^{1}H NMR of **Cat 6**

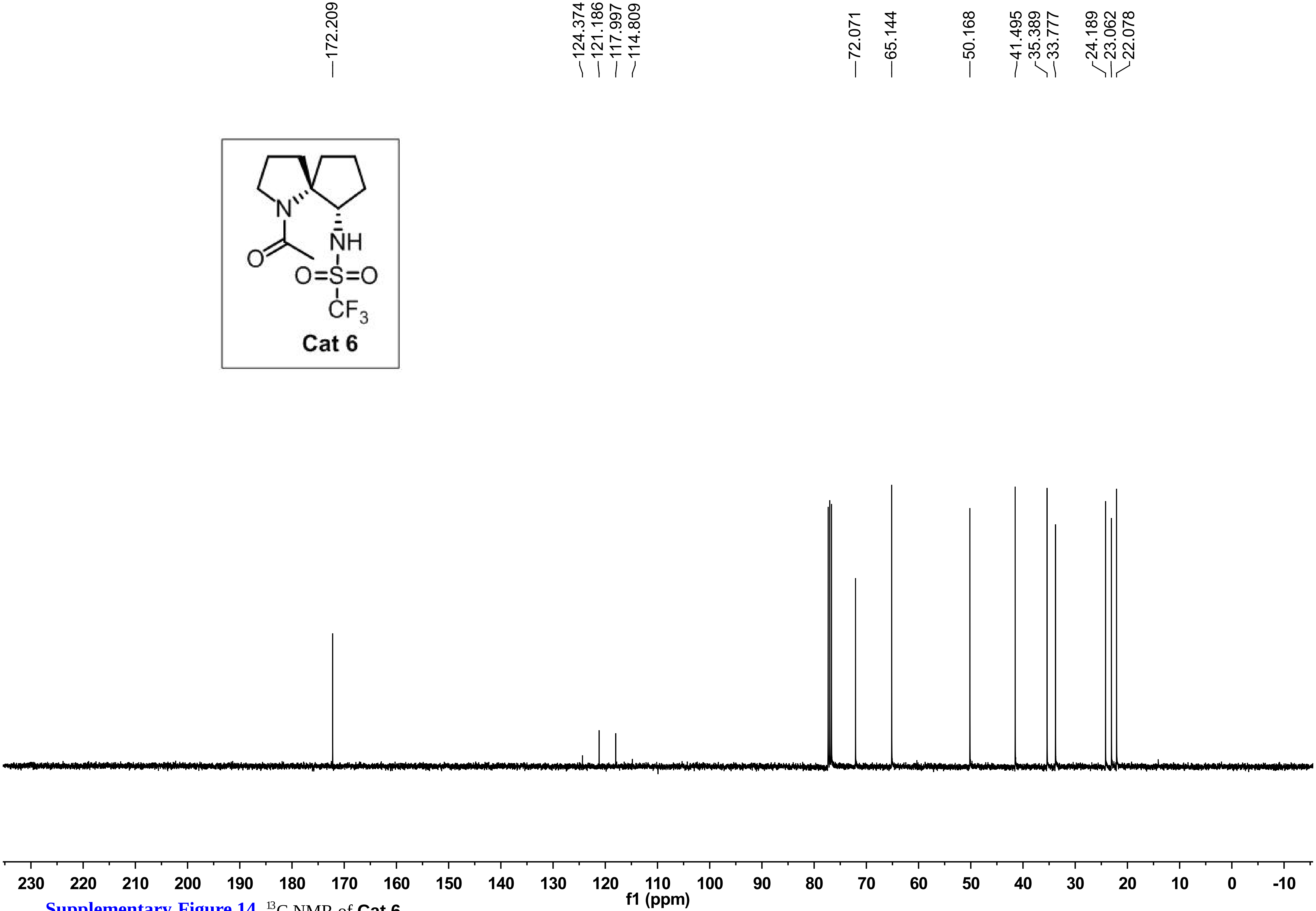

Supplementary Figure 14. ^{13}C NMR of **Cat 6**

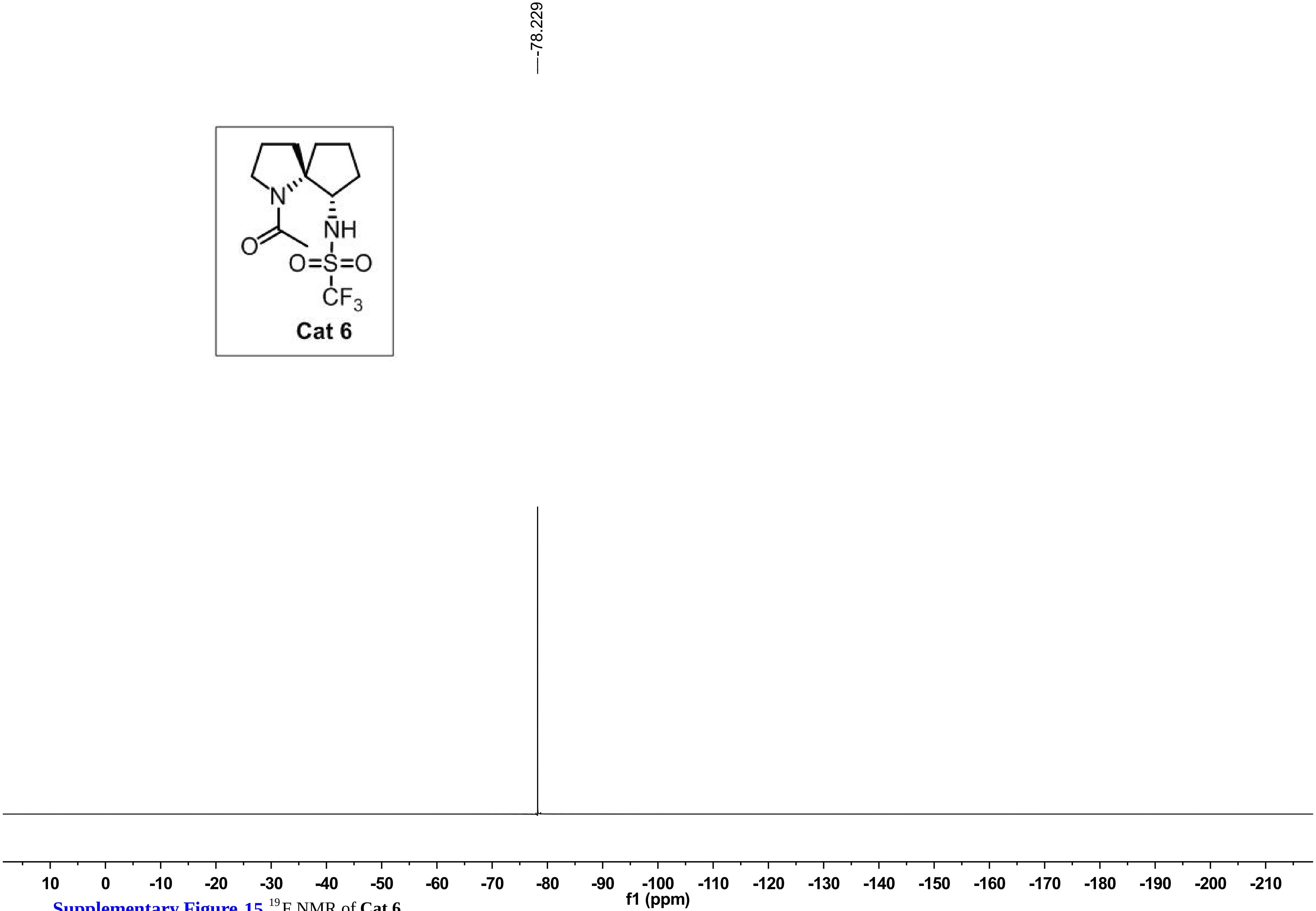

Supplementary Figure 15. ^{19}F NMR of **Cat 6**

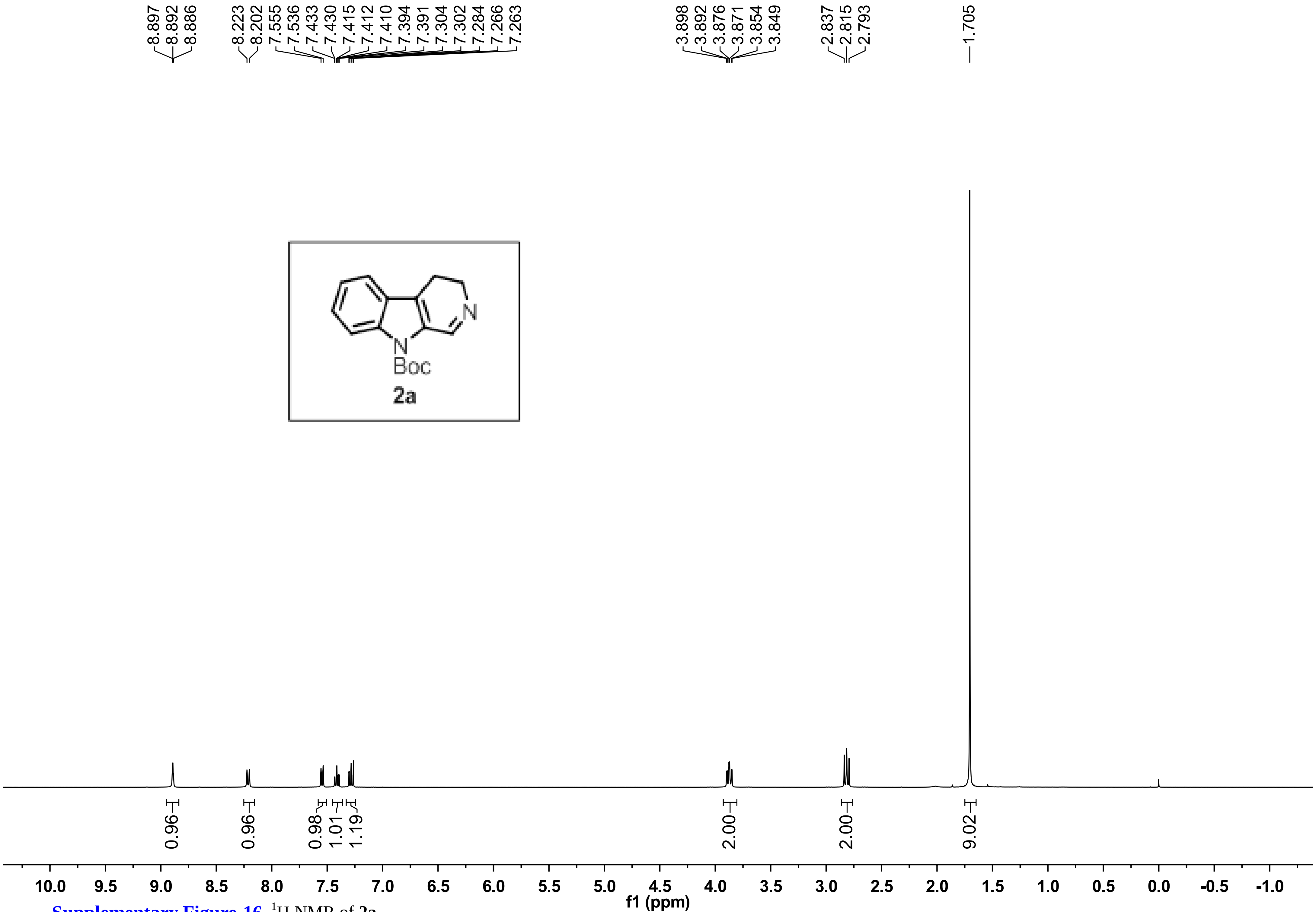

Supplementary Figure 16. ^{1}H NMR of **2a**

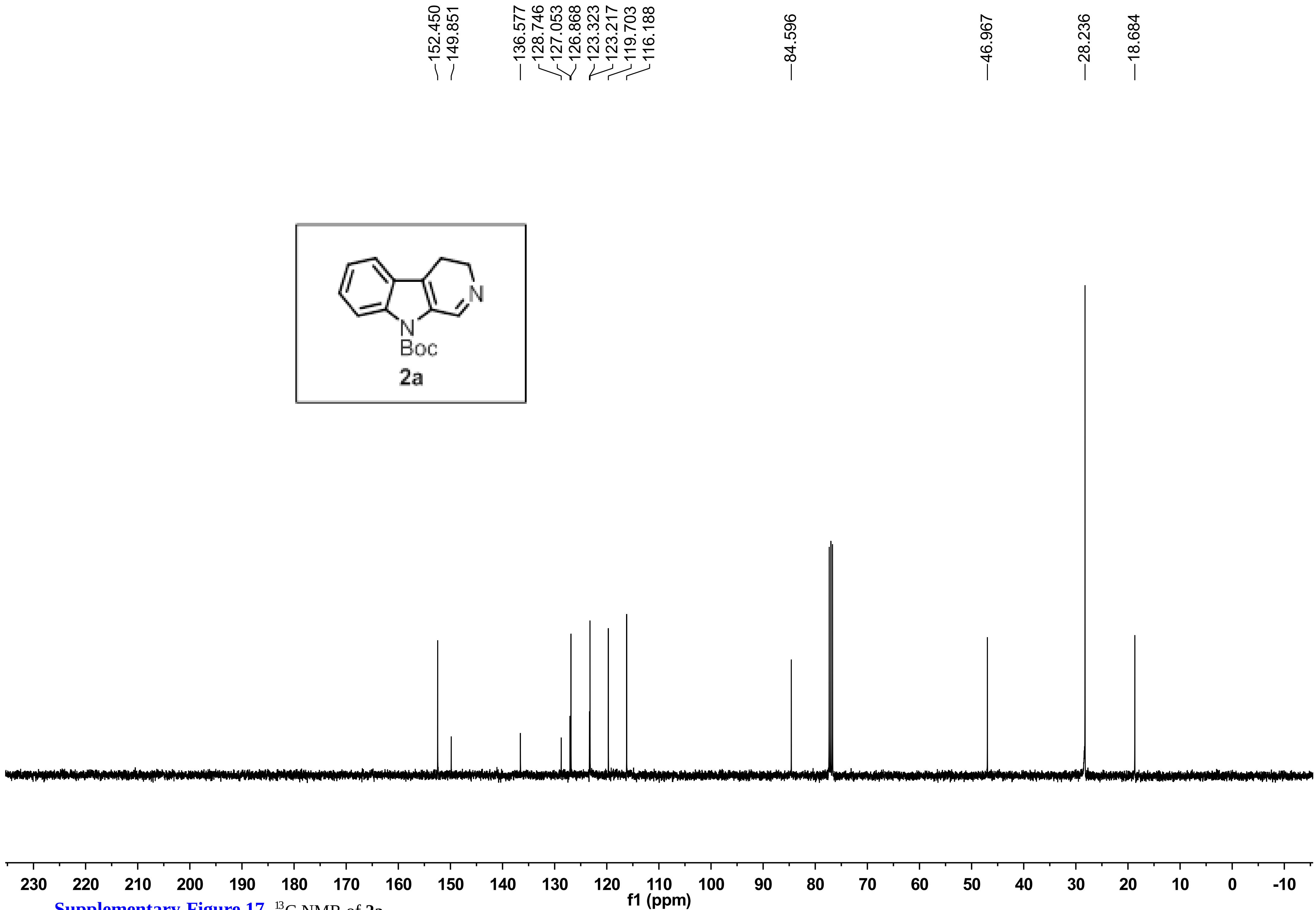

Supplementary Figure 17. ^{13}C NMR of **2a**

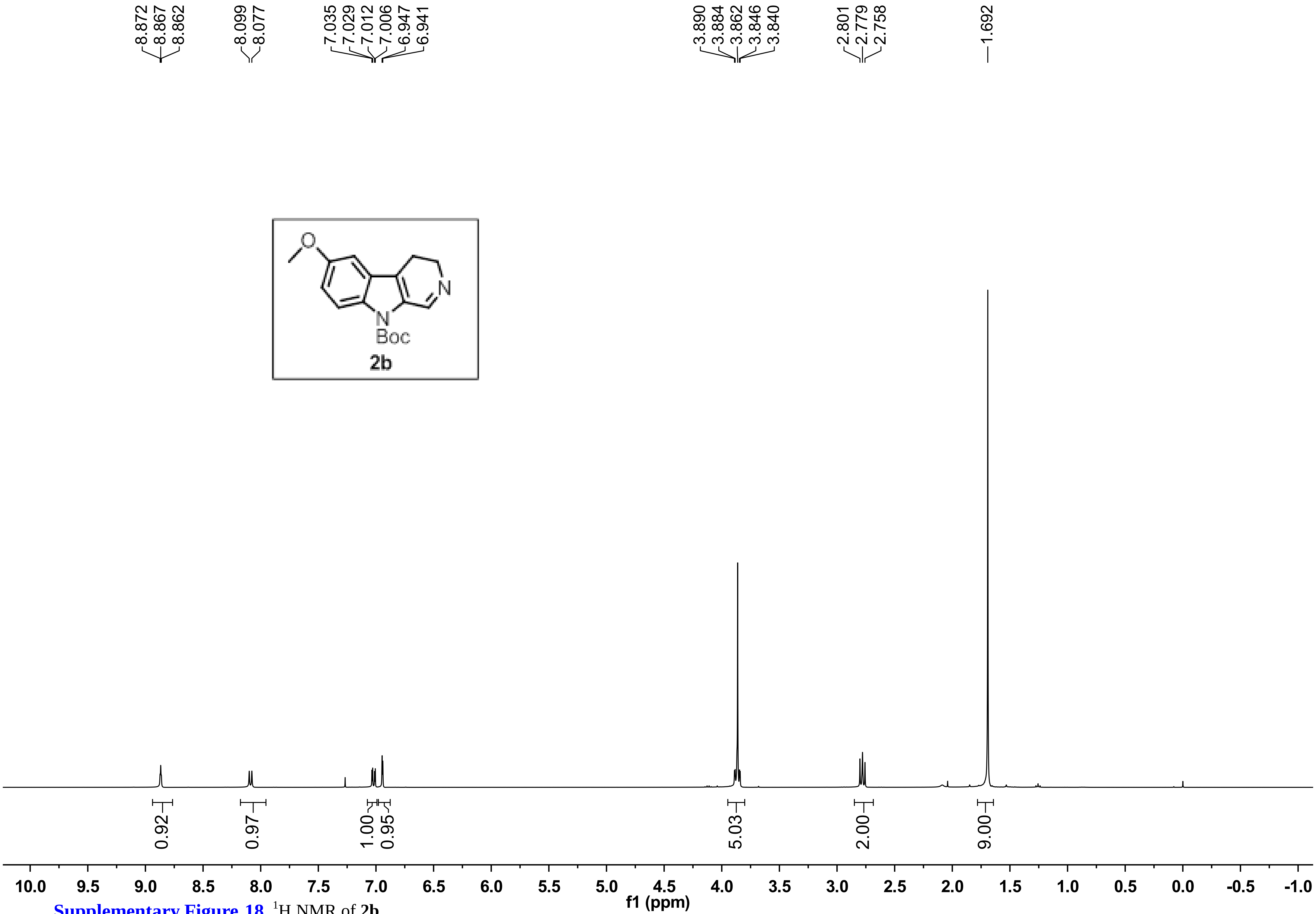

Supplementary Figure 18. ^{1}H NMR of **2b**

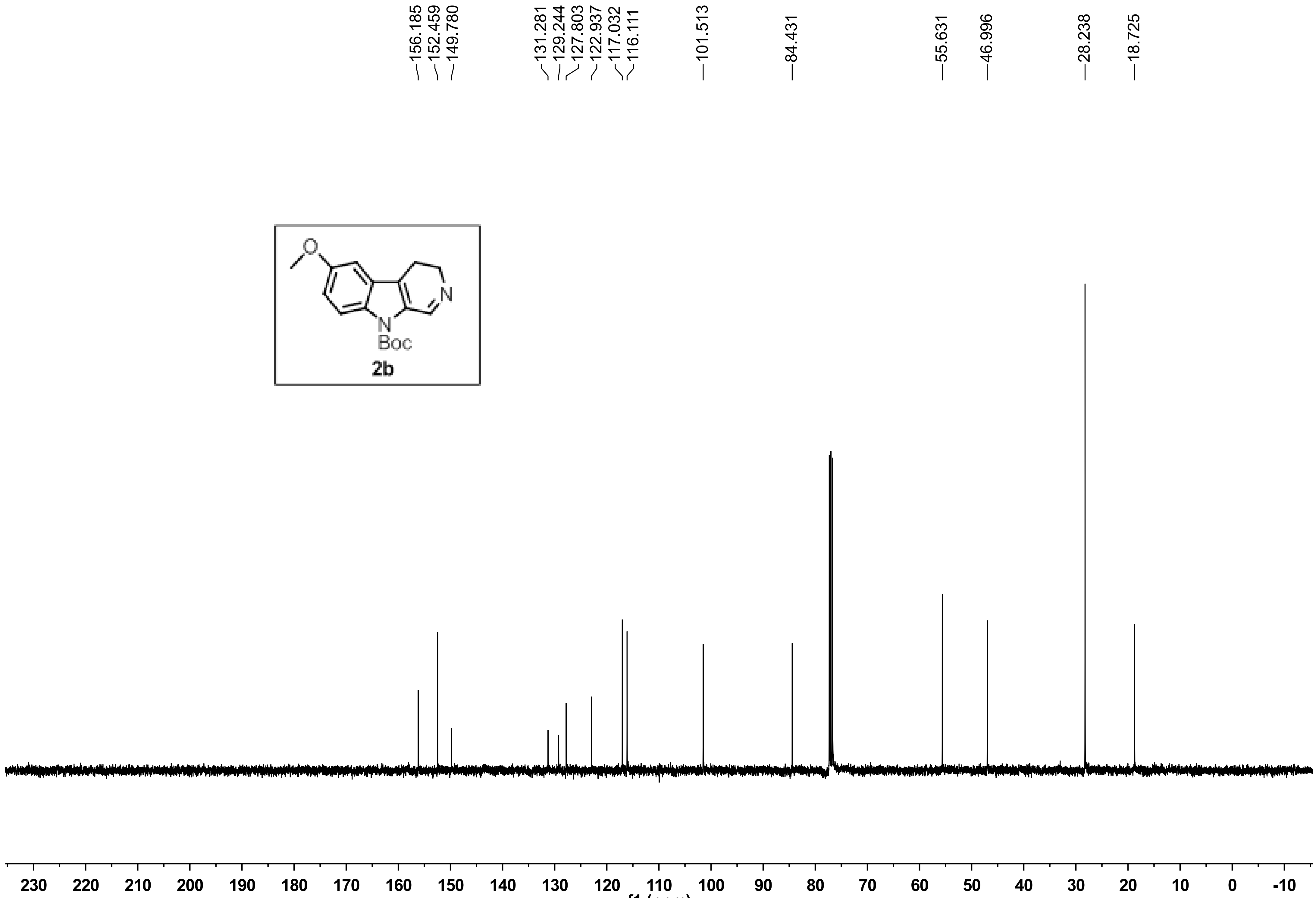

Supplementary Figure 19. ^{13}C NMR of **2b**

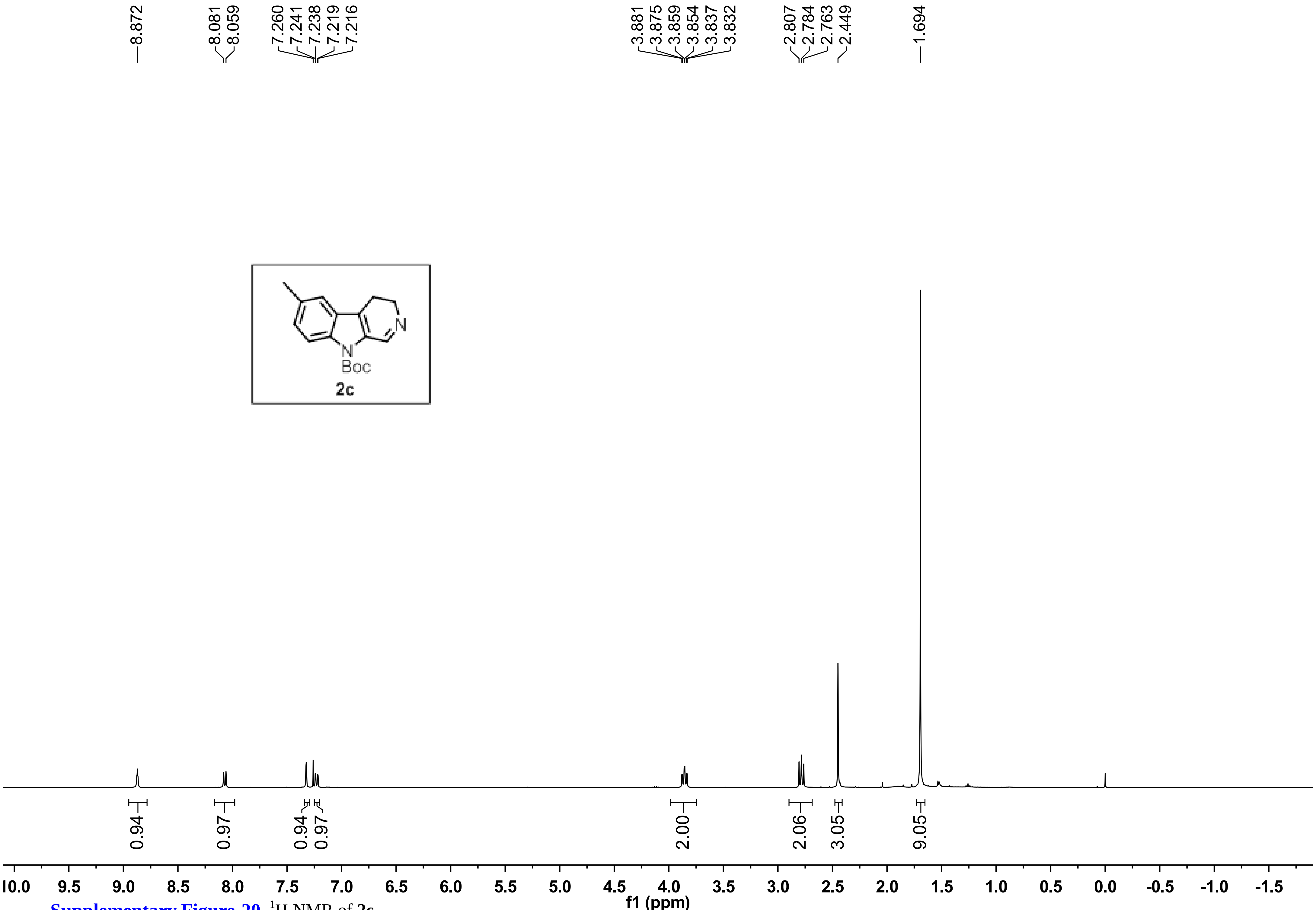

Supplementary Figure 20. ^{1}H NMR of **2c**

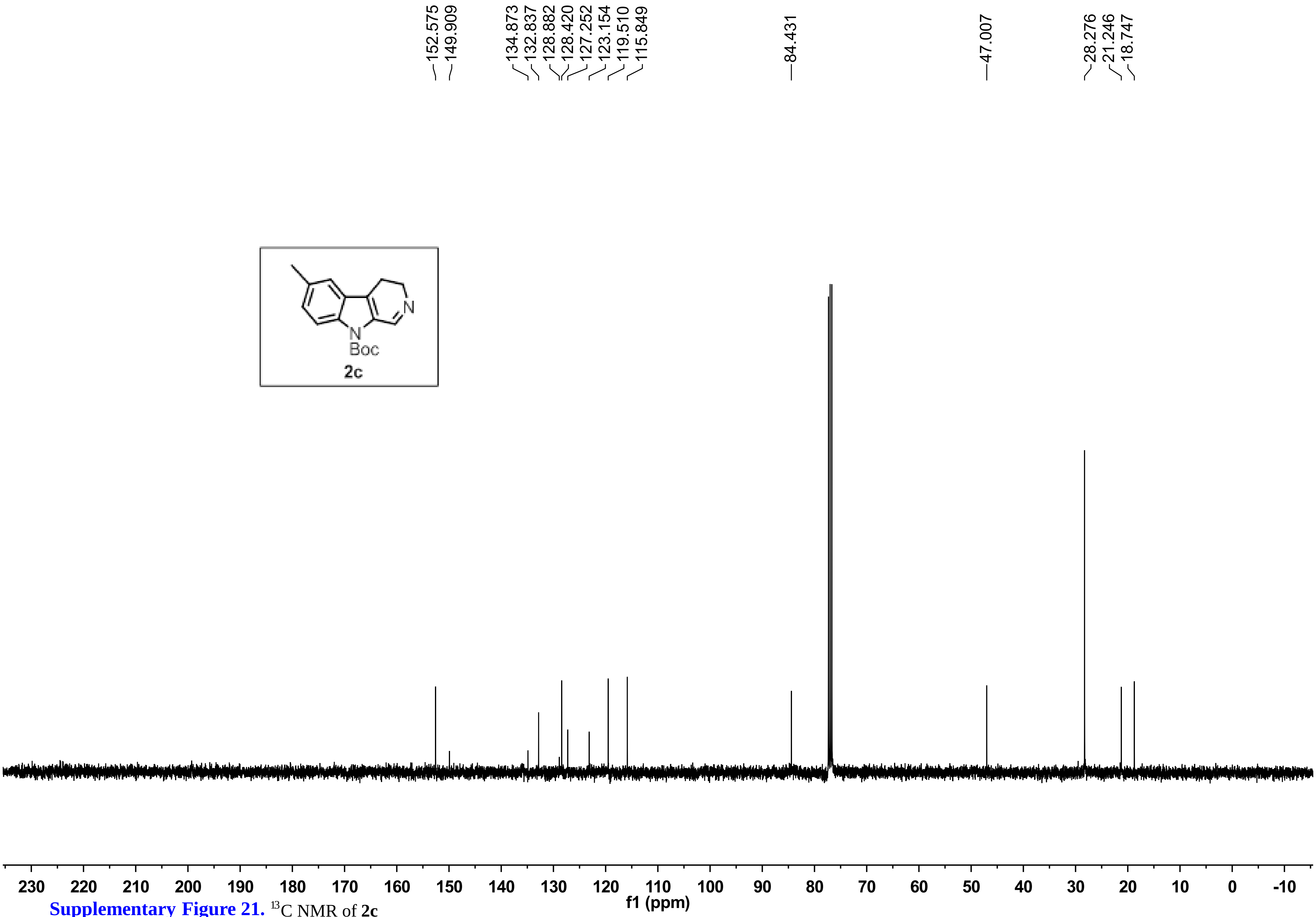

Supplementary Figure 21. ^{13}C NMR of **2c**

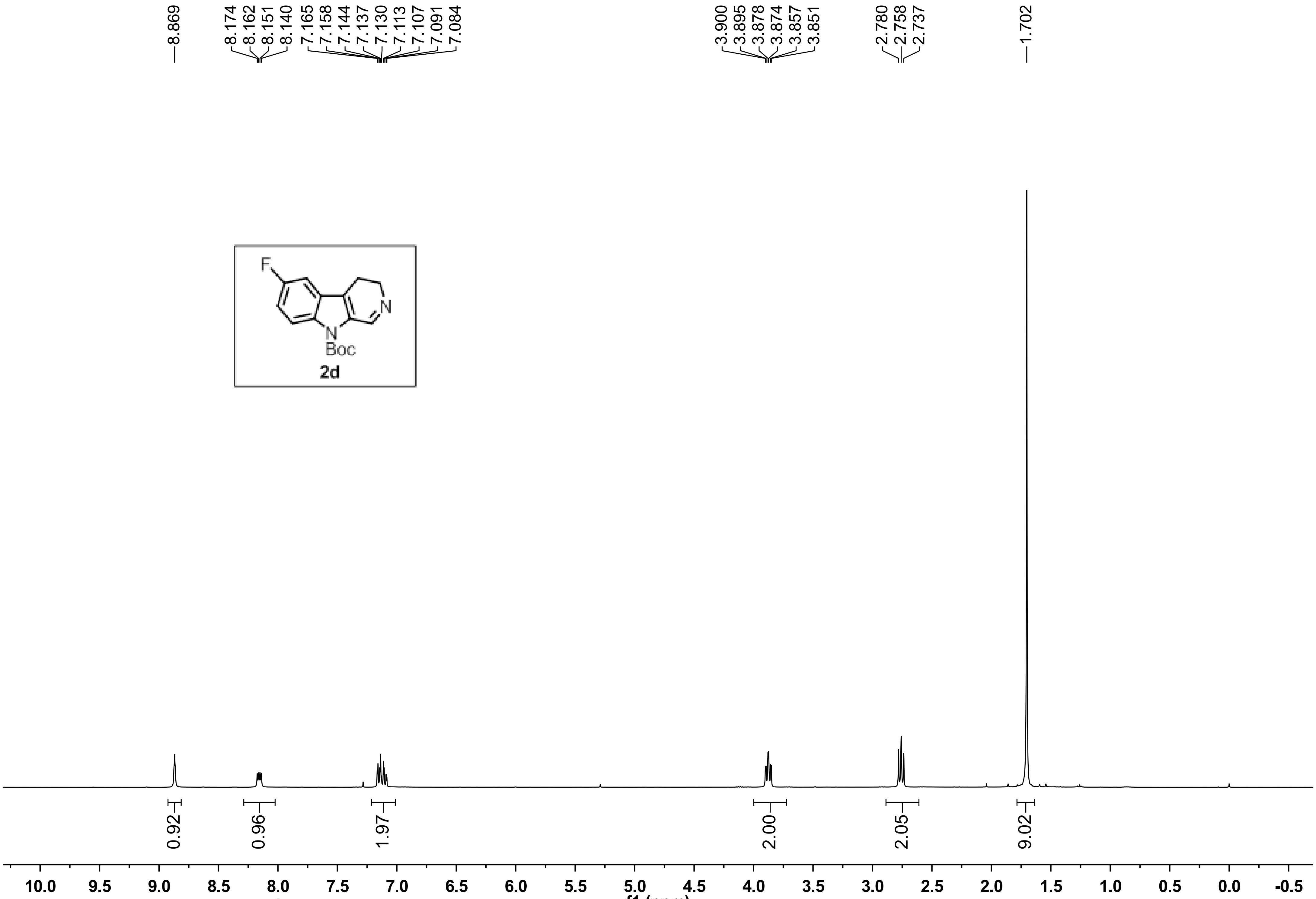

Supplementary Figure 22. ^{1}H NMR of **2d**

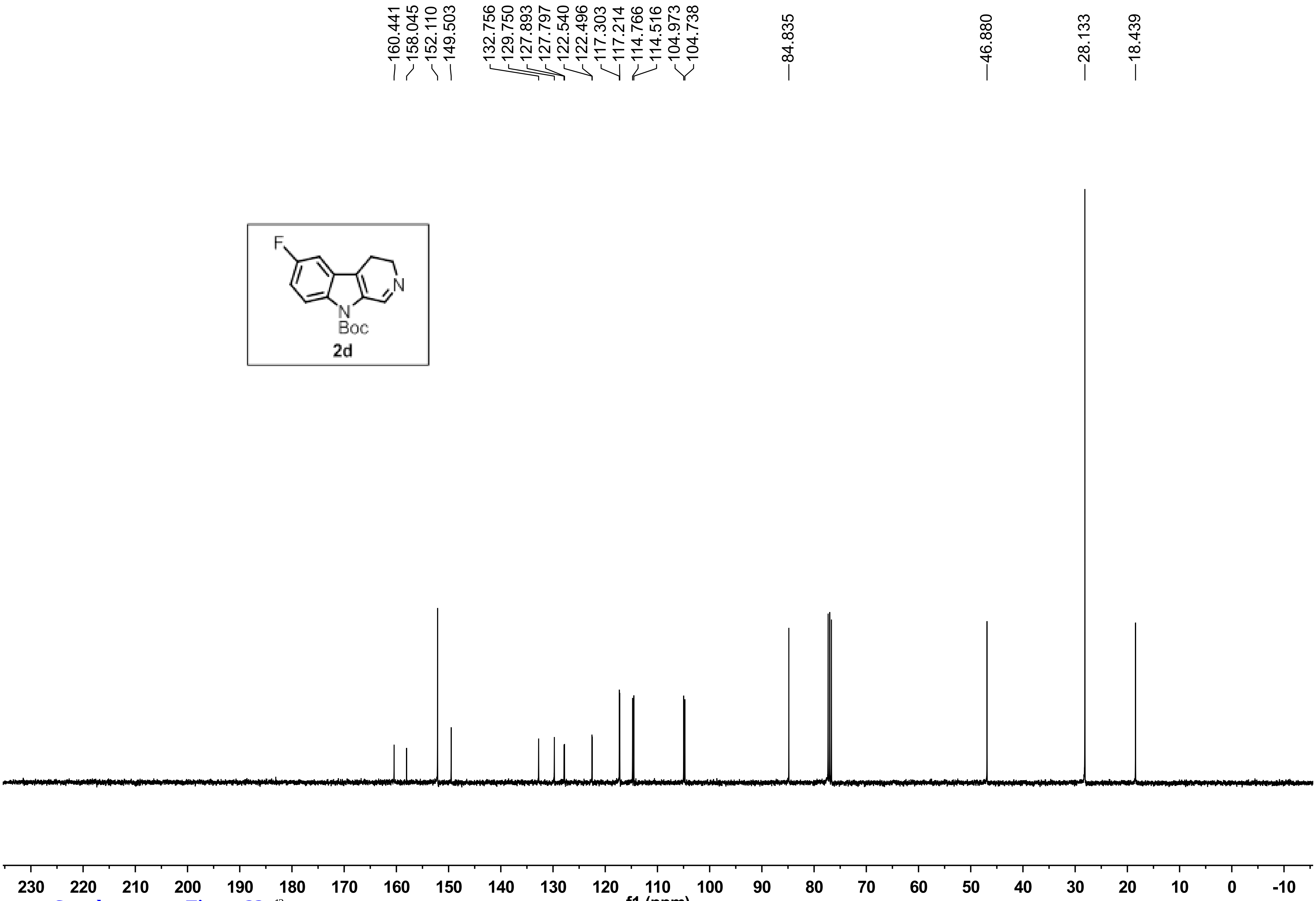

Supplementary Figure 23. ^{13}C NMR of **2d**

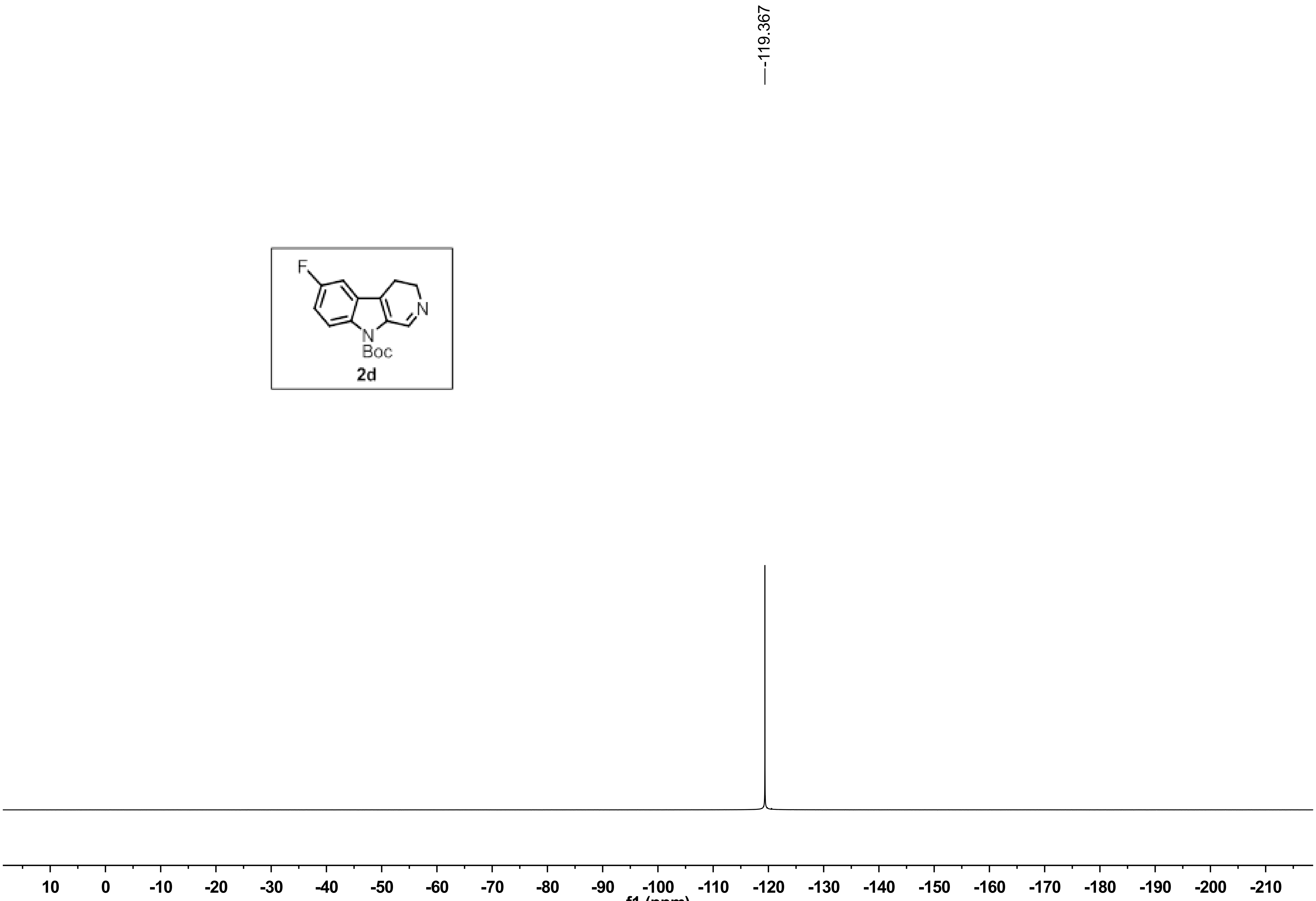

Supplementary Figure 24. ^{19}F NMR of **2d**

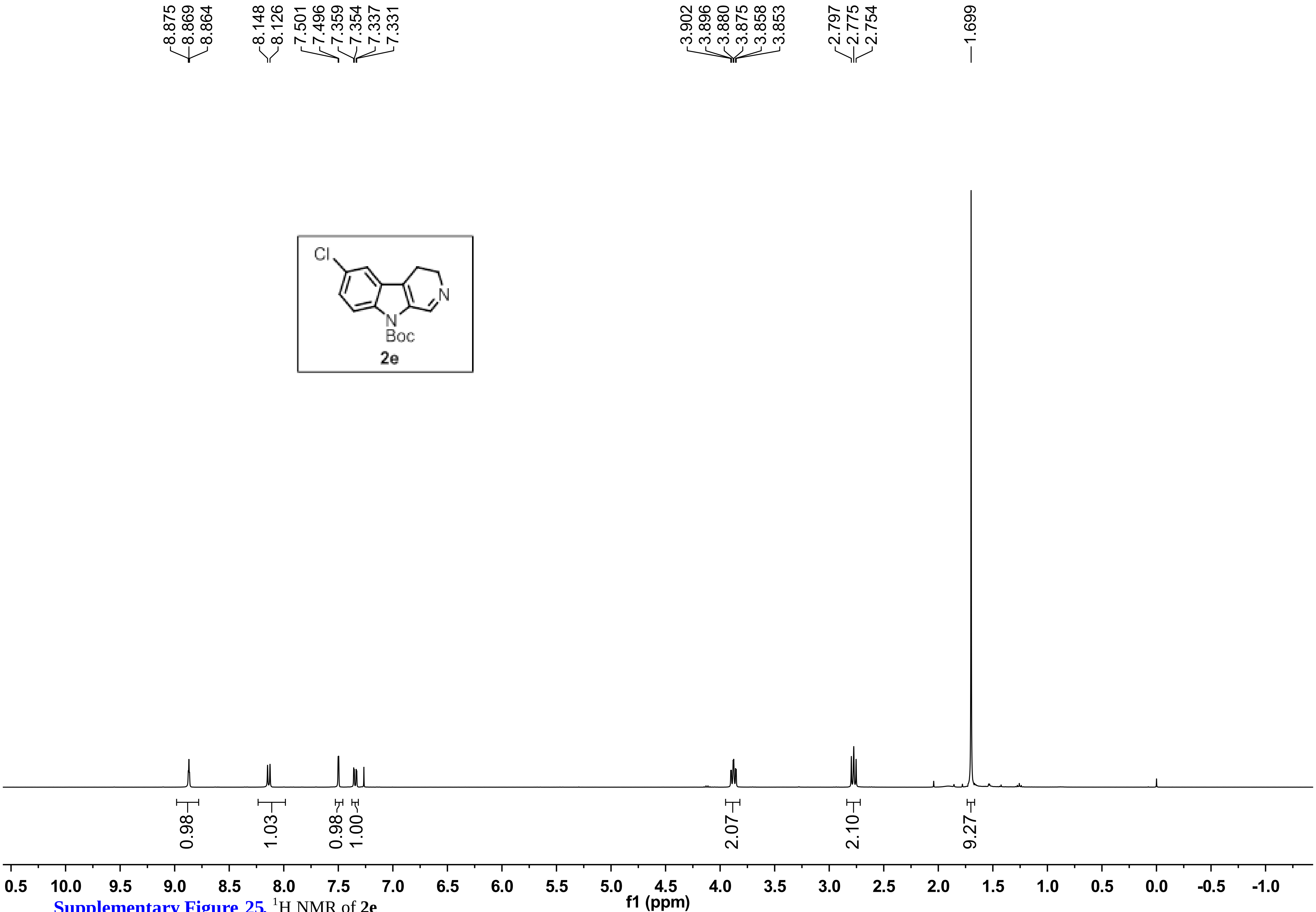

Supplementary Figure 25. ^{1}H NMR of **2e**

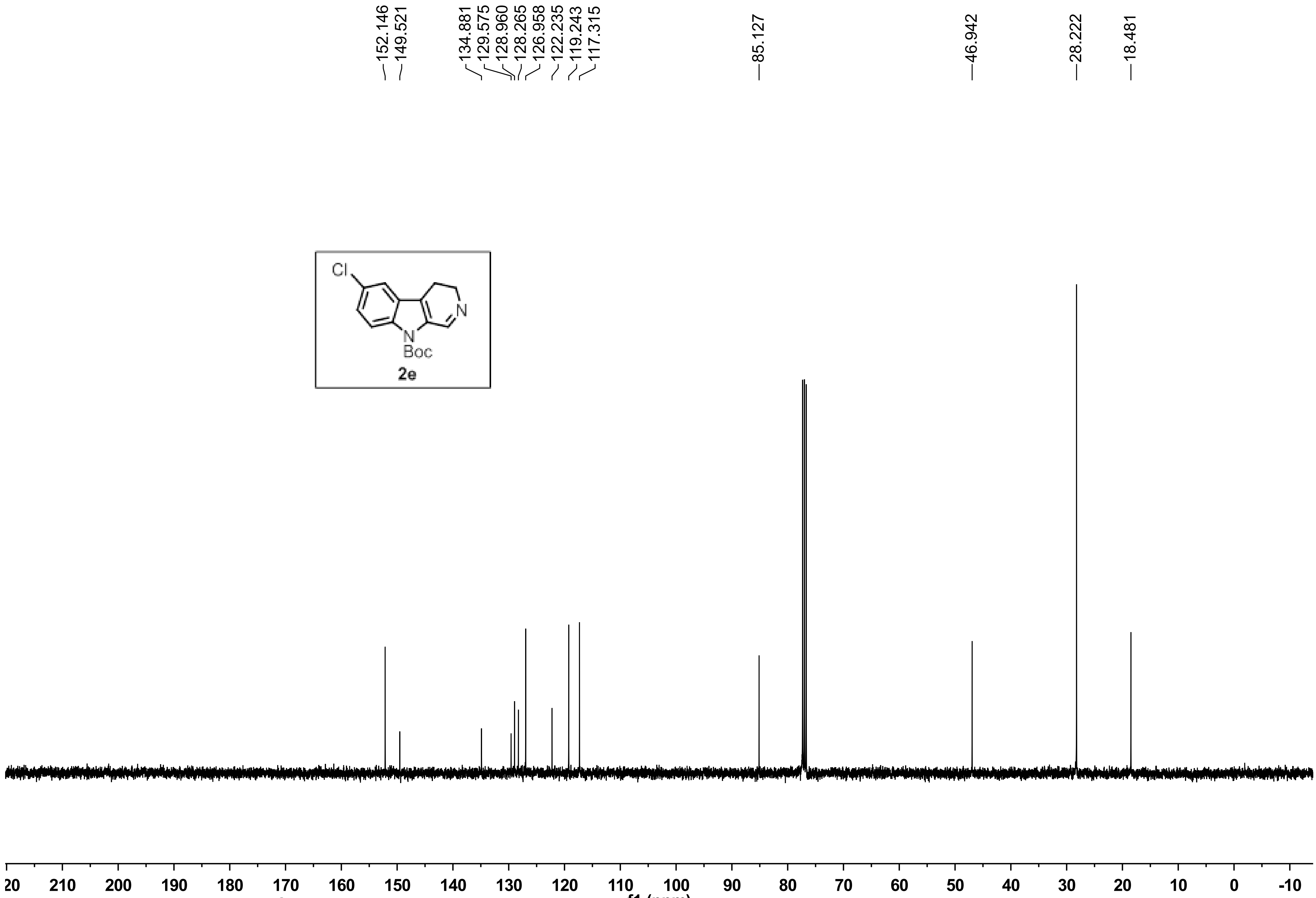

Supplementary Figure 26. ^{13}C NMR of **2e**

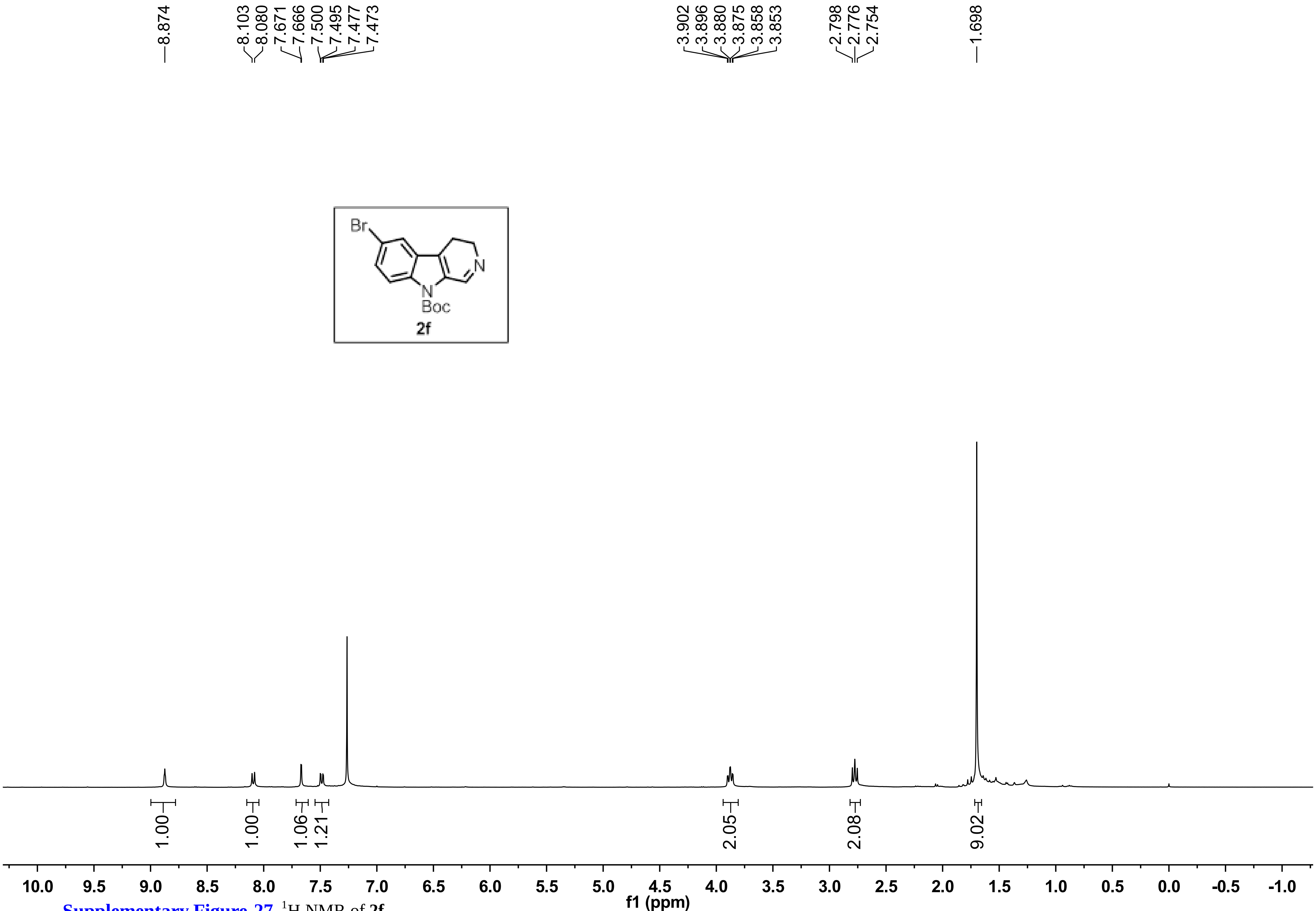

Supplementary Figure 27. ^{1}H NMR of **2f**

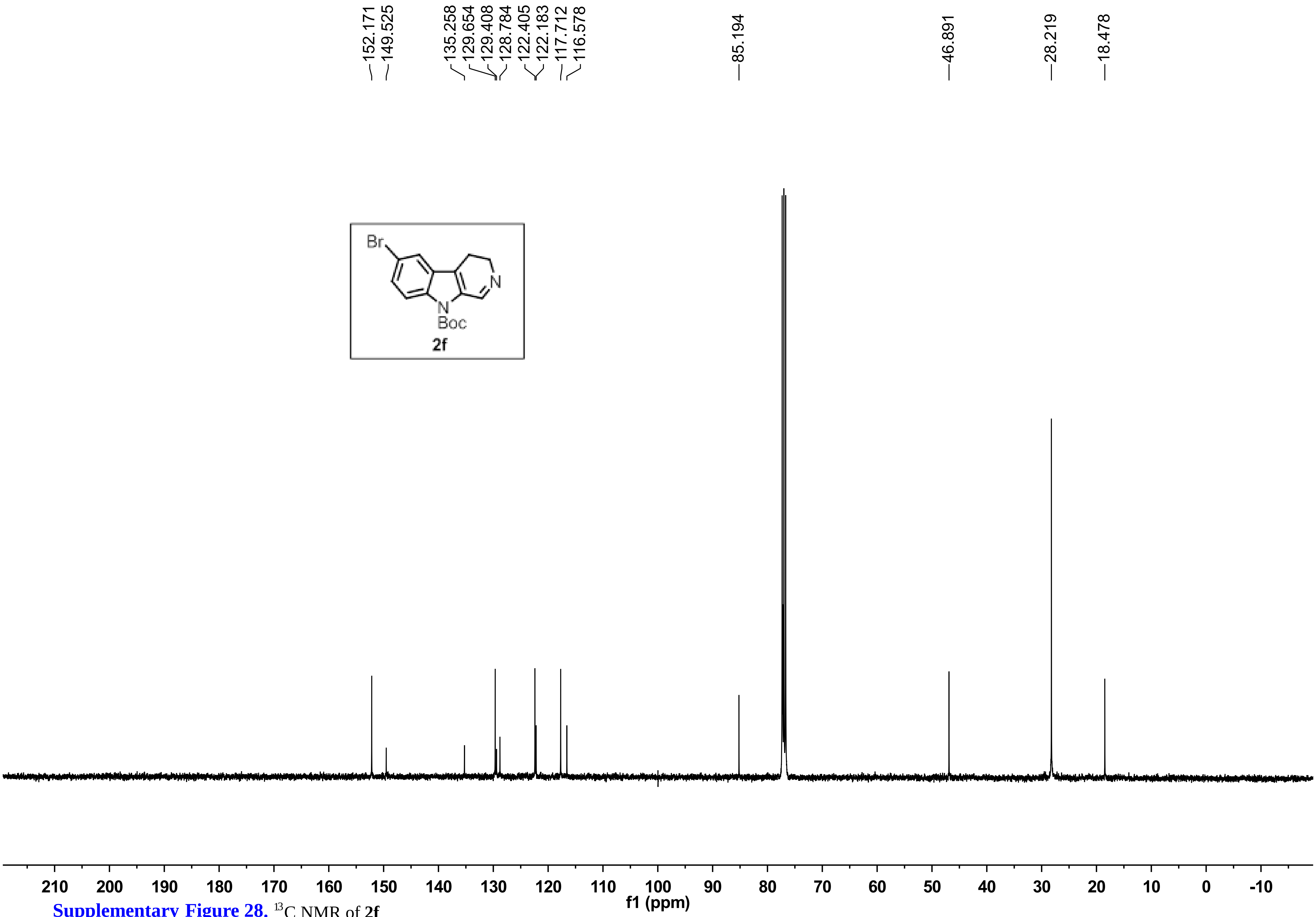

Supplementary Figure 28. ^{13}C NMR of **2f**

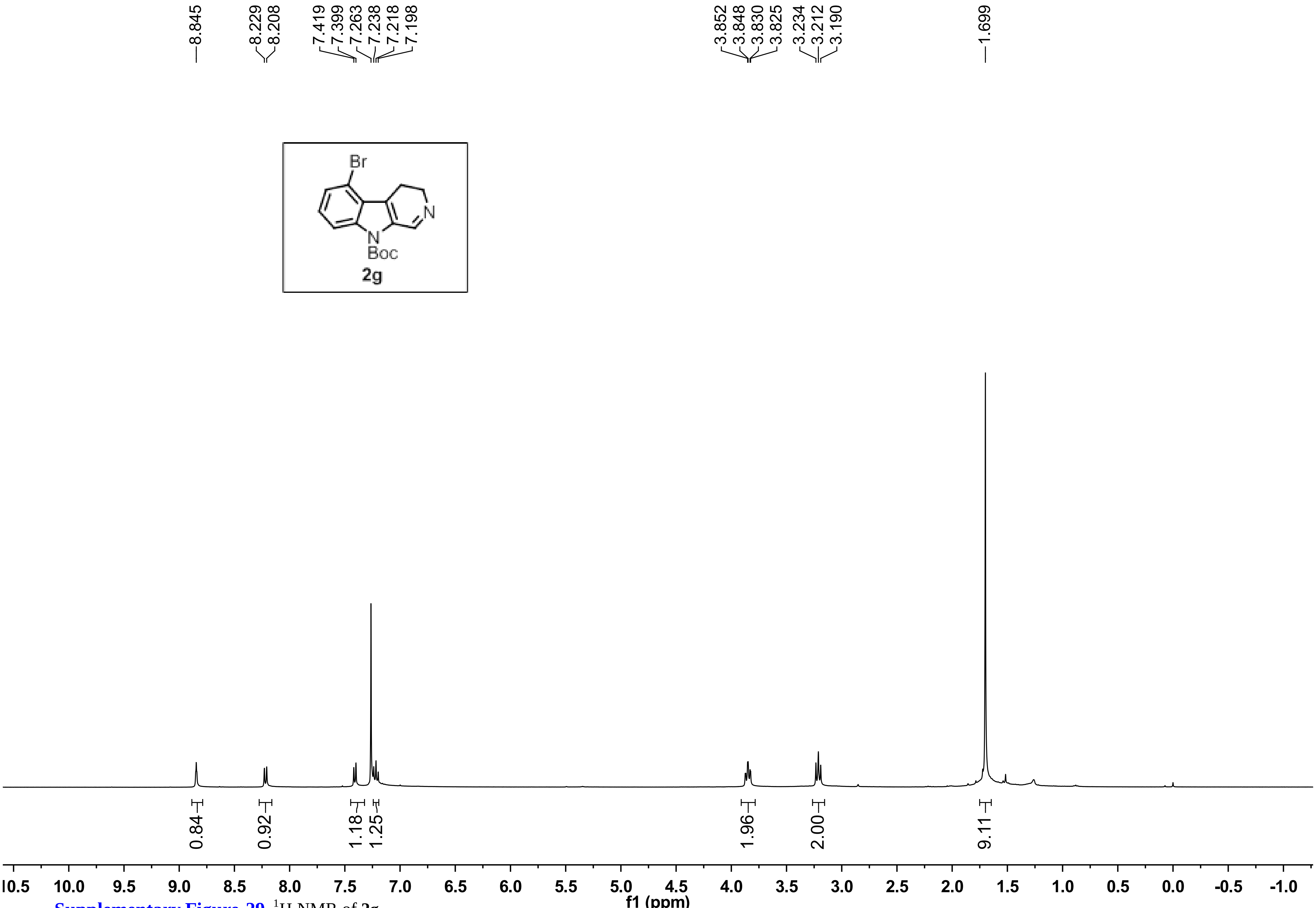

Supplementary Figure 29. ^{1}H NMR of **2g**

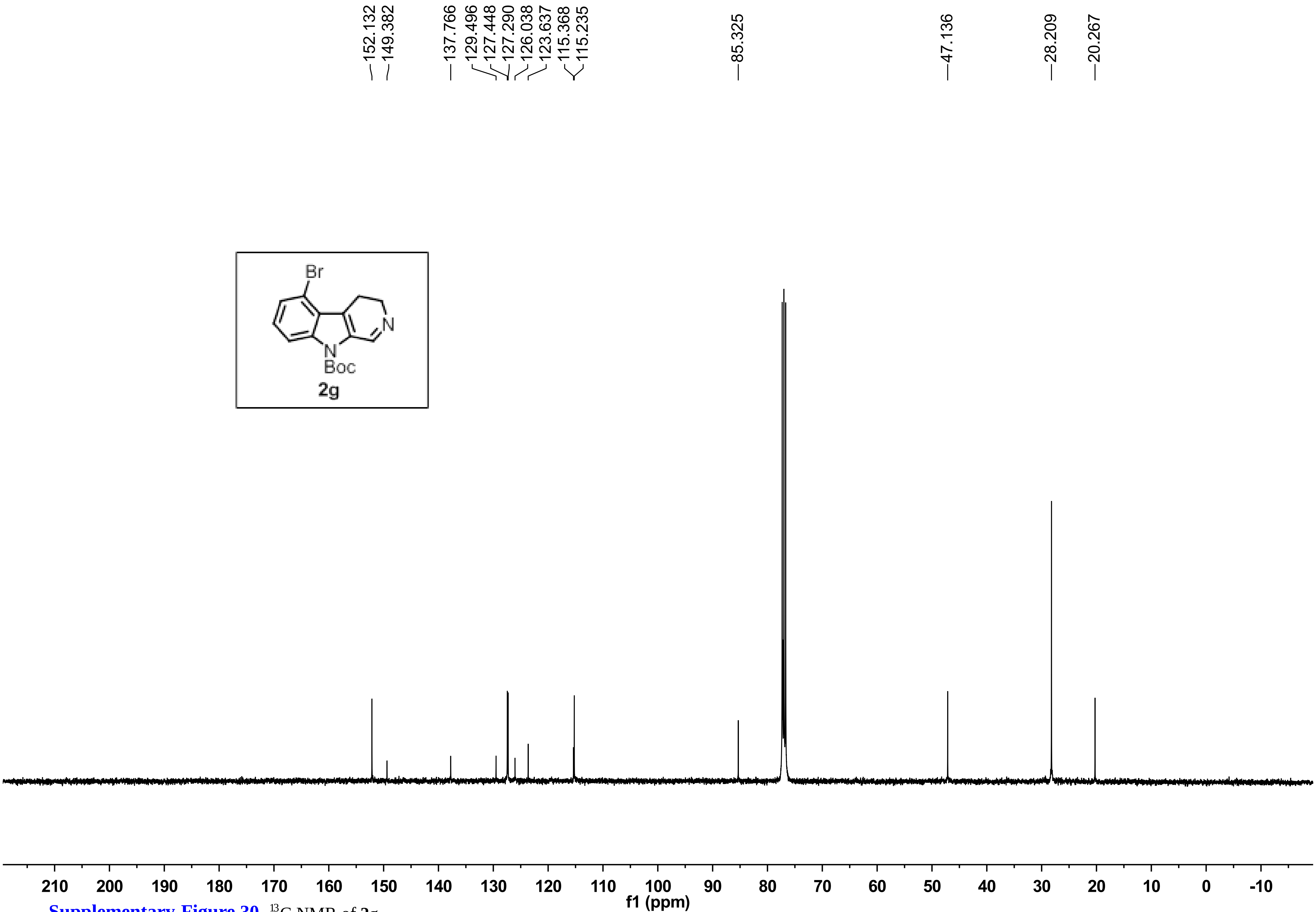

Supplementary Figure 30. ^{13}C NMR of **2g**

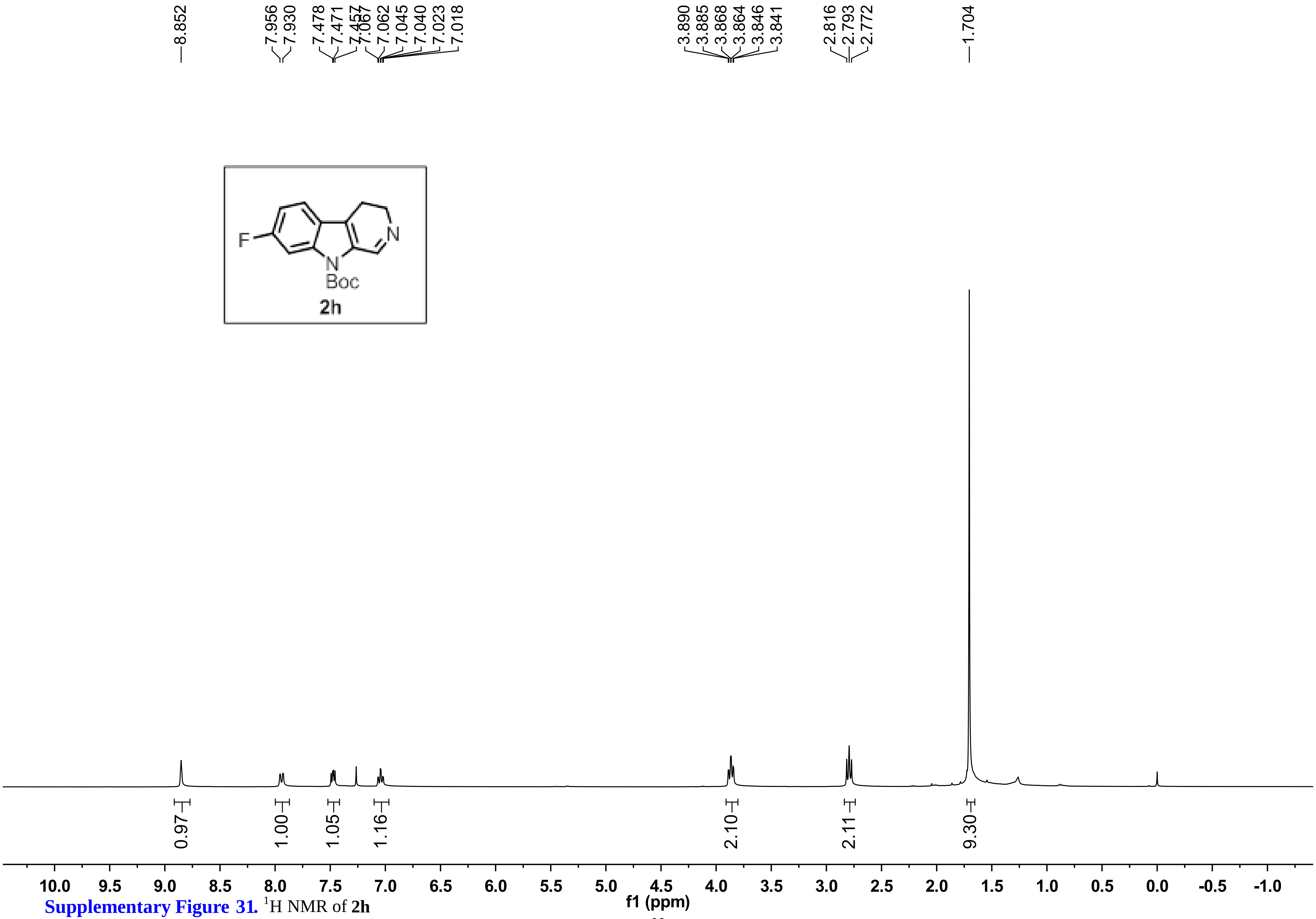

Supplementary Figure 31. ^{1}H NMR of **2h**

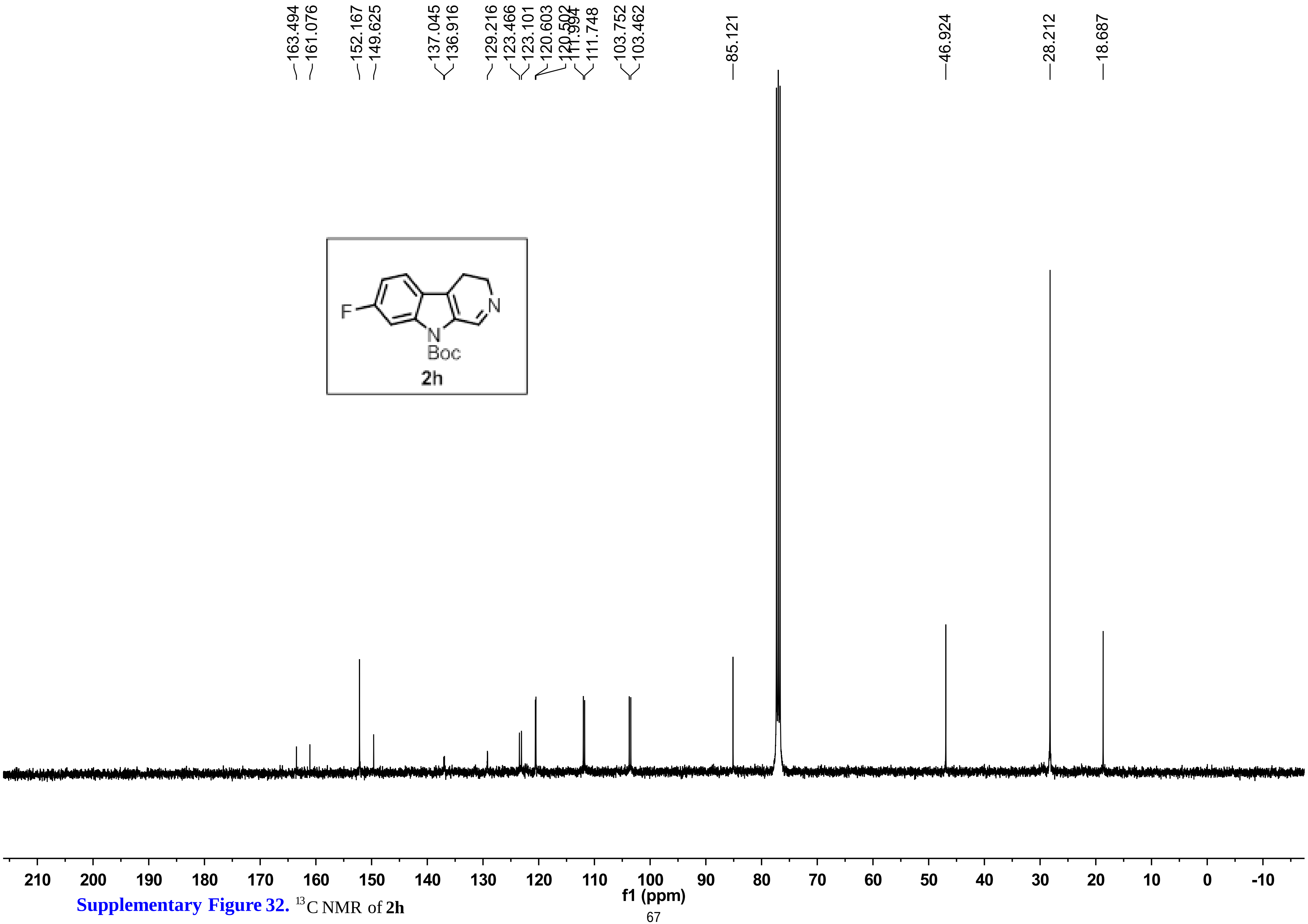

Supplementary Figure 32. ^{13}C NMR of **2h**

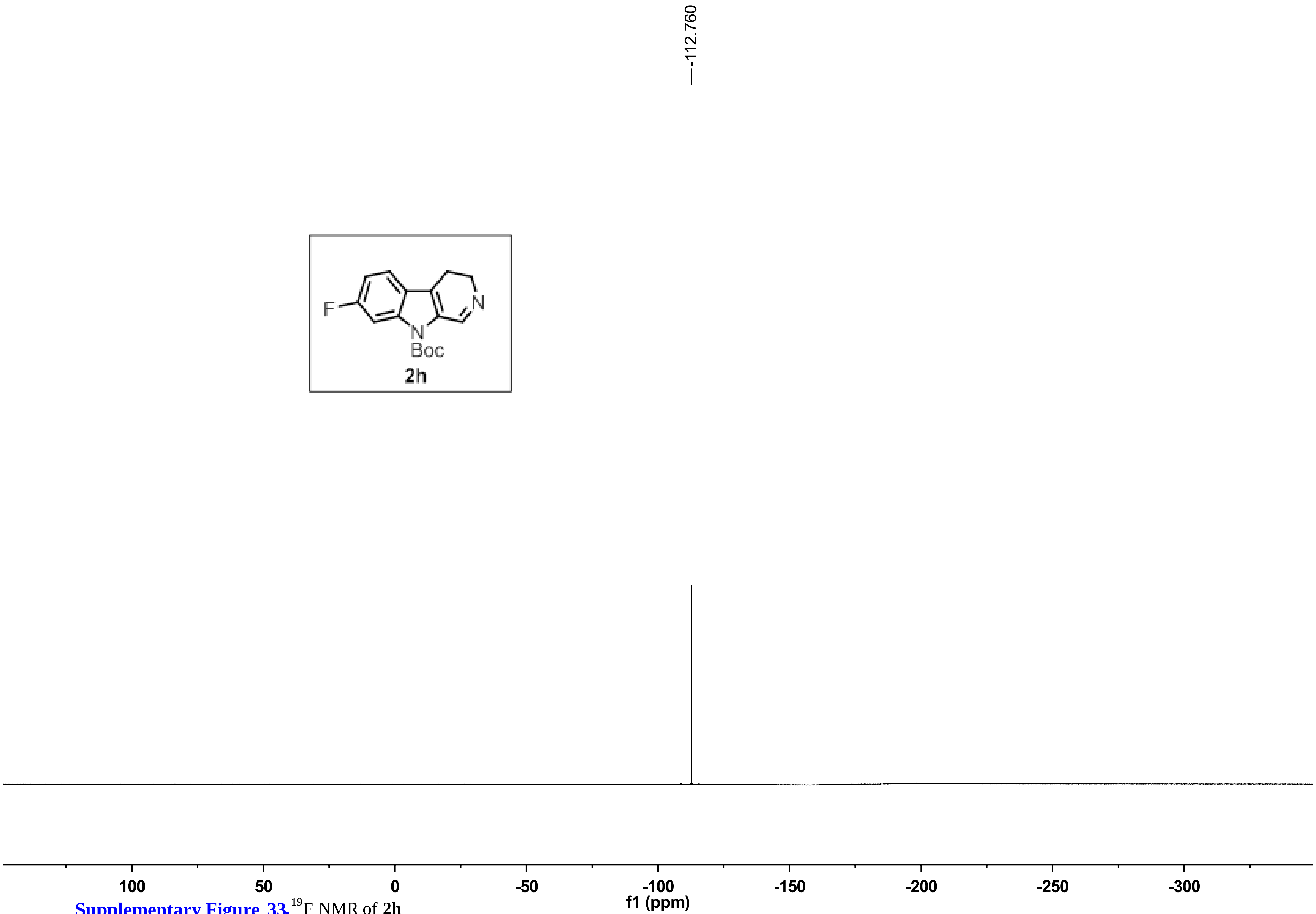

Supplementary Figure 33. ^{19}F NMR of **2h**

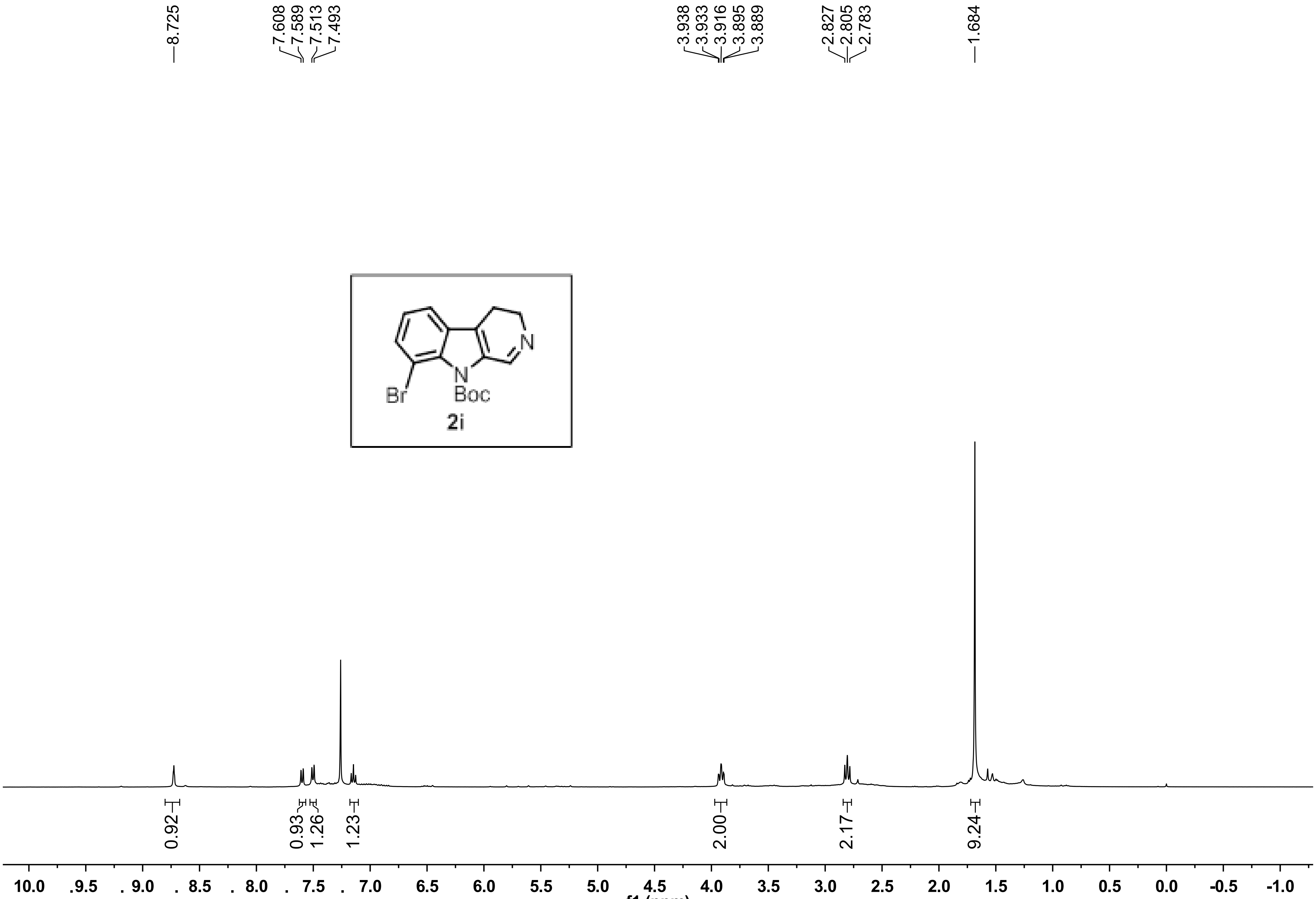

Supplementary Figure 34. ^{1}H NMR of **2i**

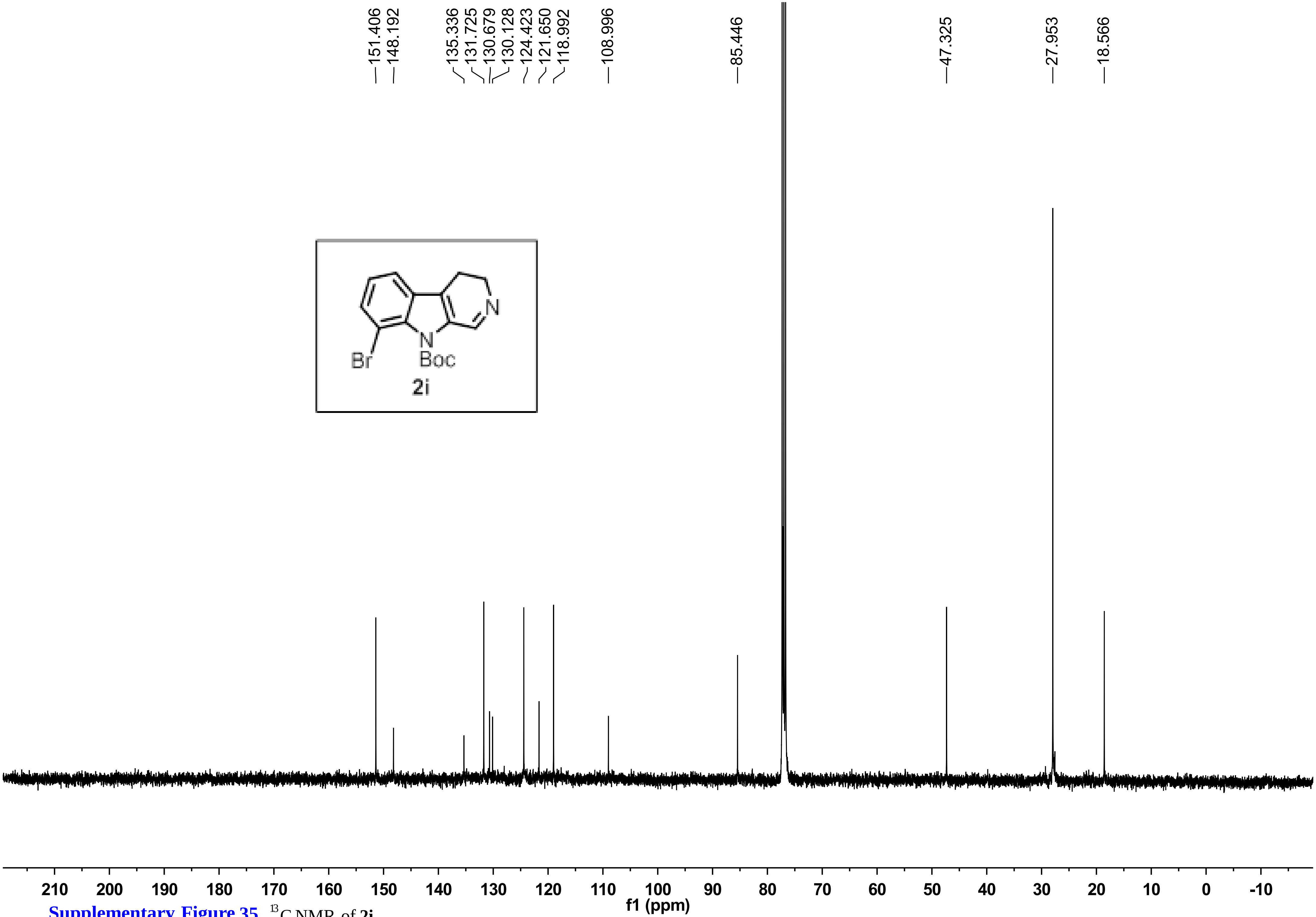

Supplementary Figure 35. ^{13}C NMR of **2i**

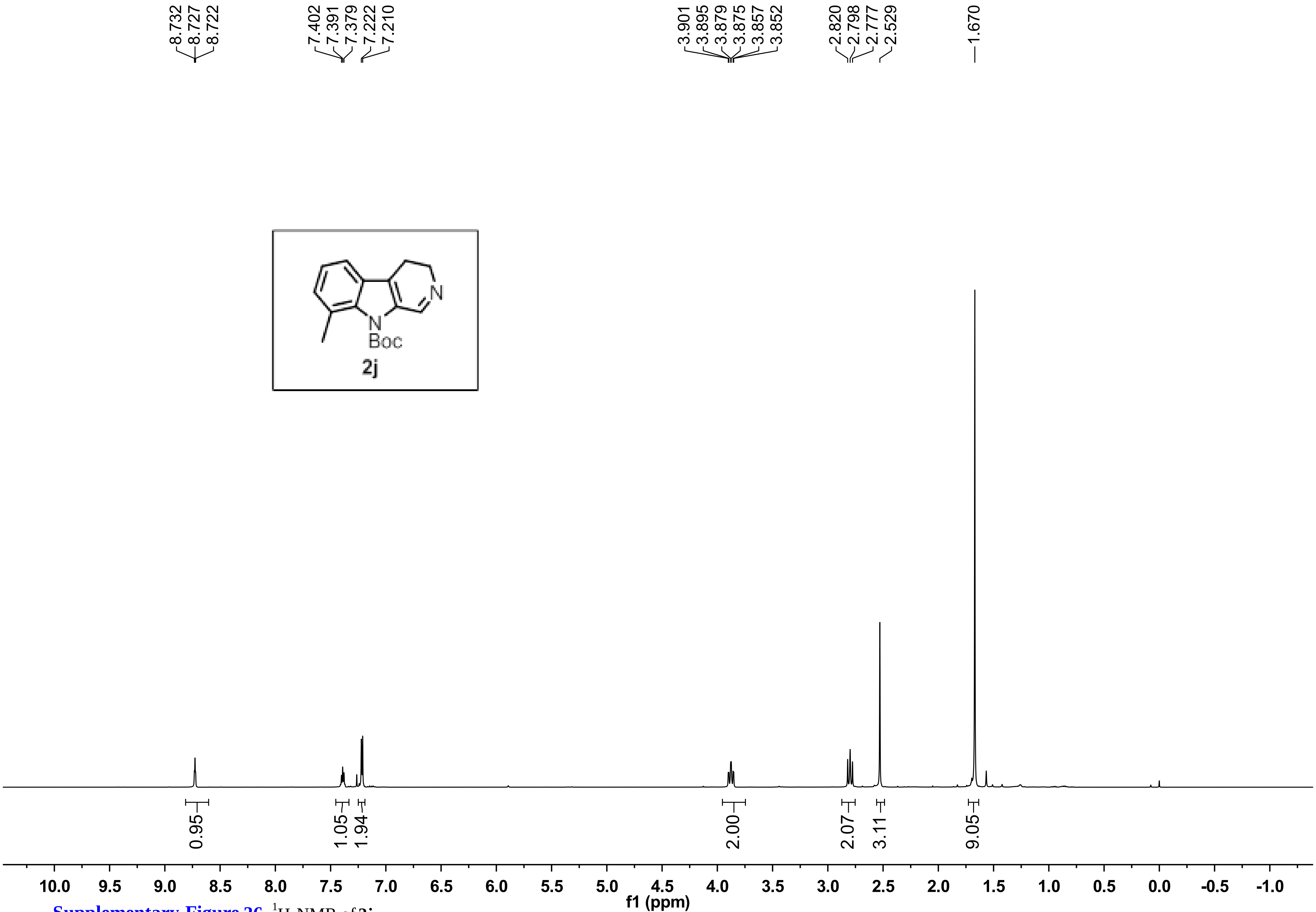

Supplementary Figure 36. ^{1}H NMR of **2j**

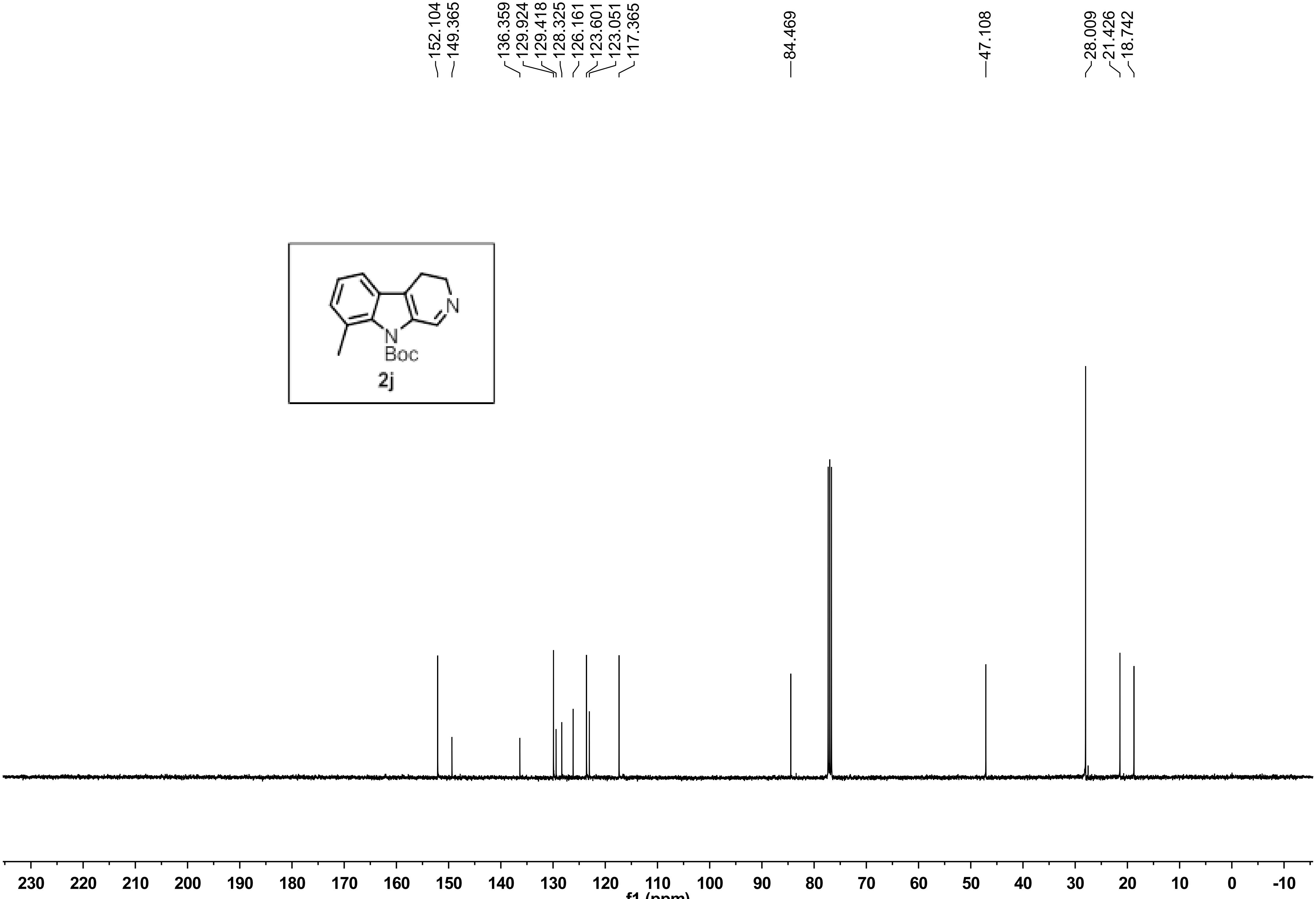

Supplementary Figure 37. ^{13}C NMR of **2j**

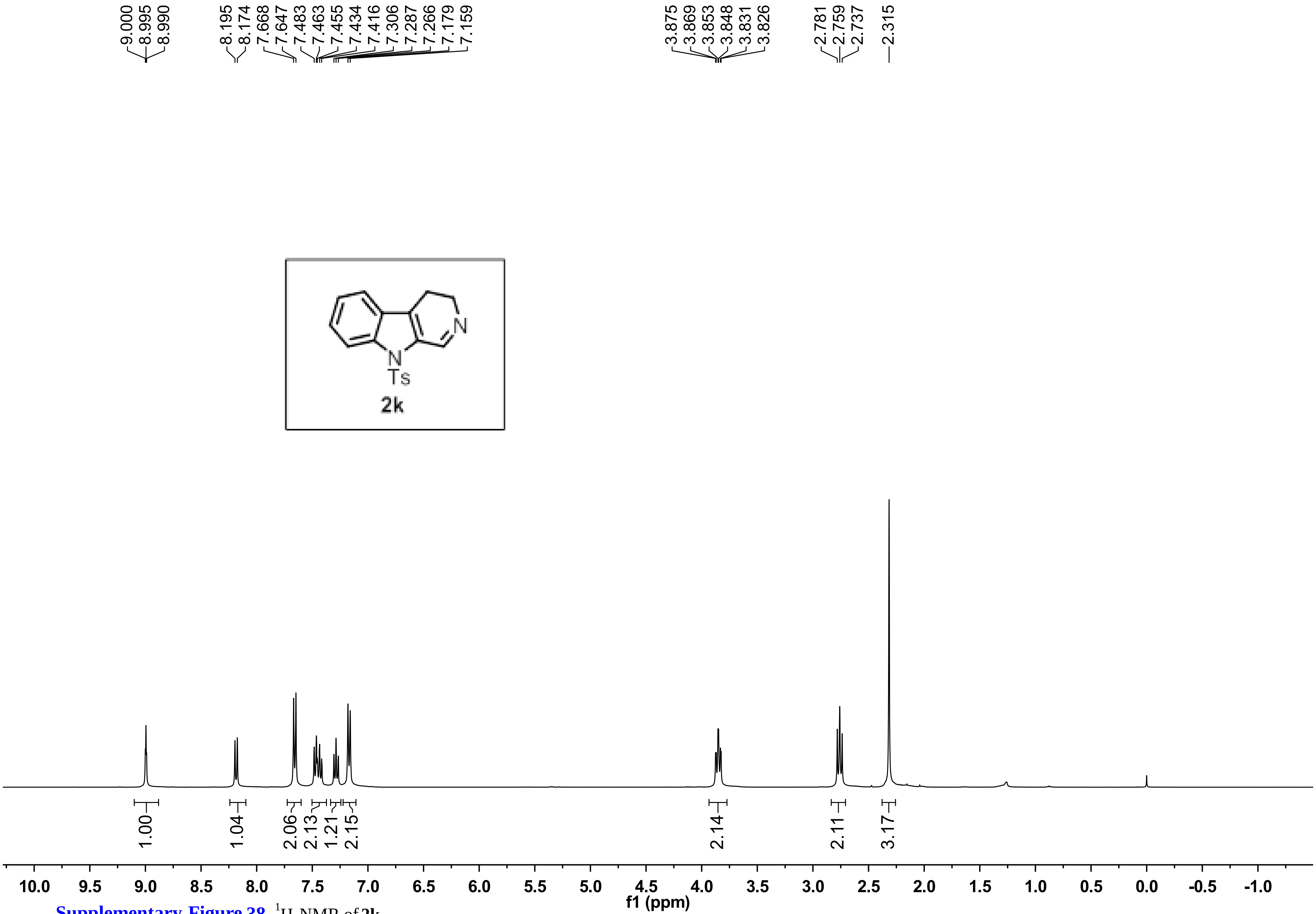

Supplementary Figure 38. ^{1}H NMR of **2k**

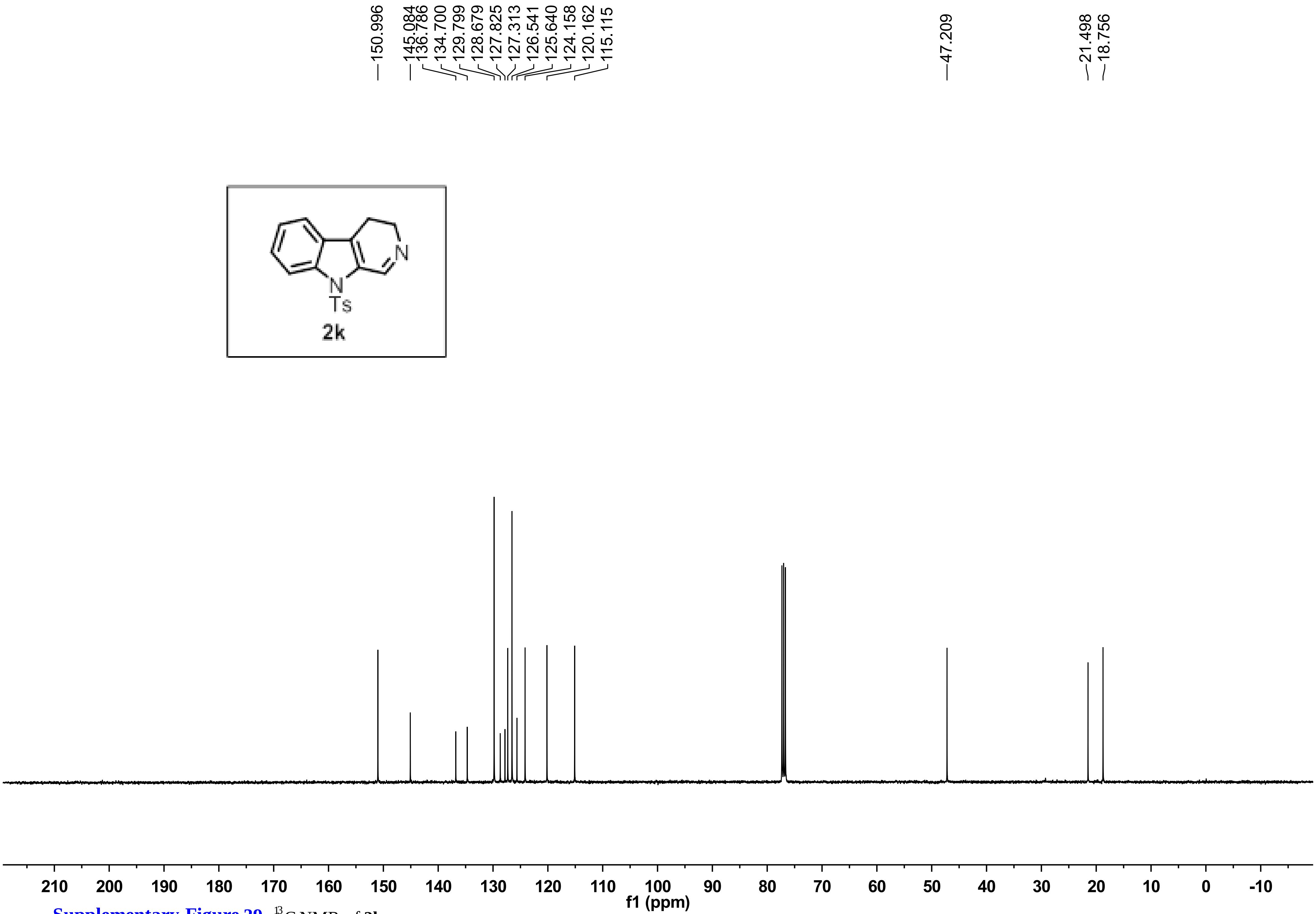

Supplementary Figure 39. ^{13}C NMR of **2k**

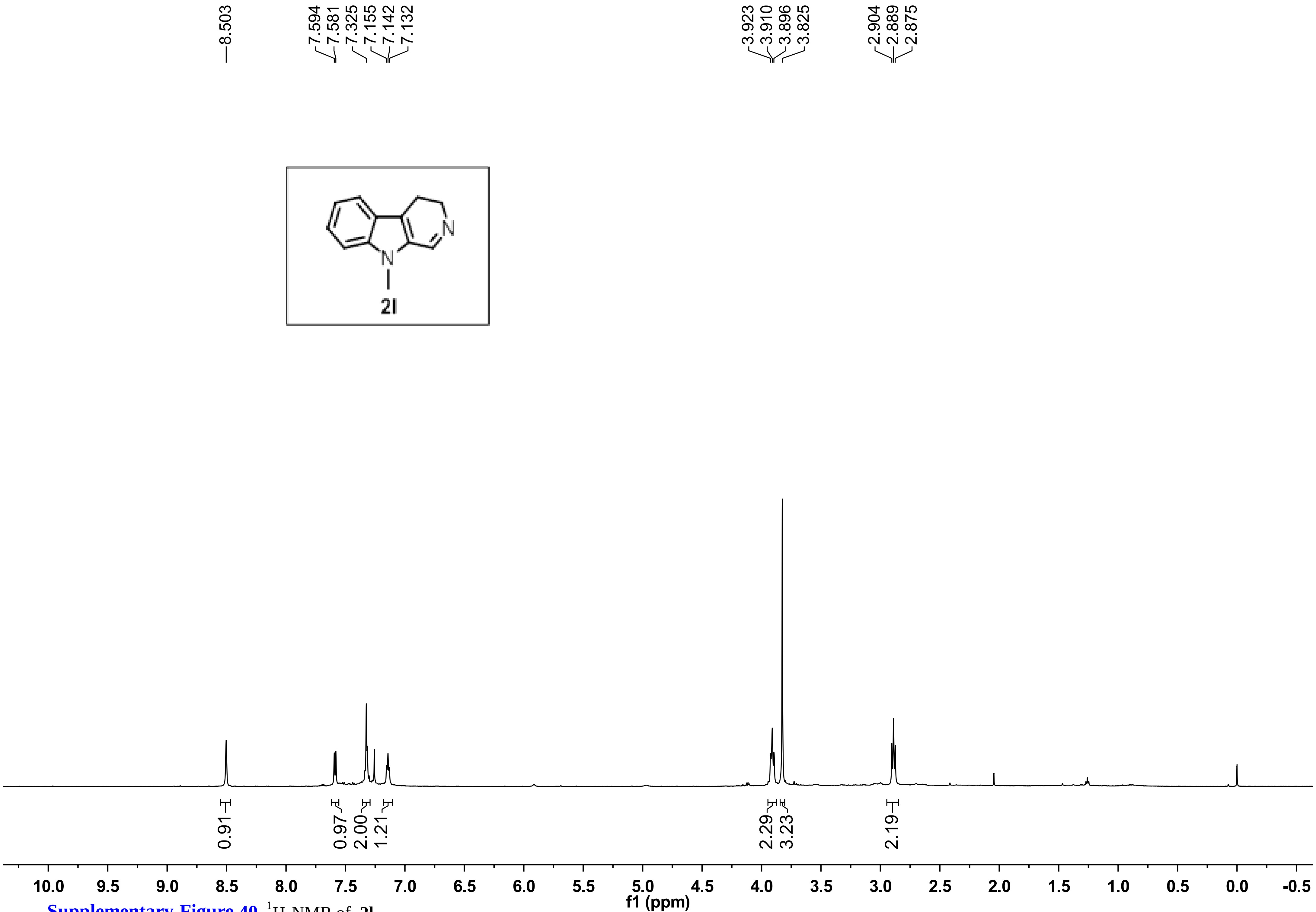

Supplementary Figure 40. ^{1}H NMR of **2l**

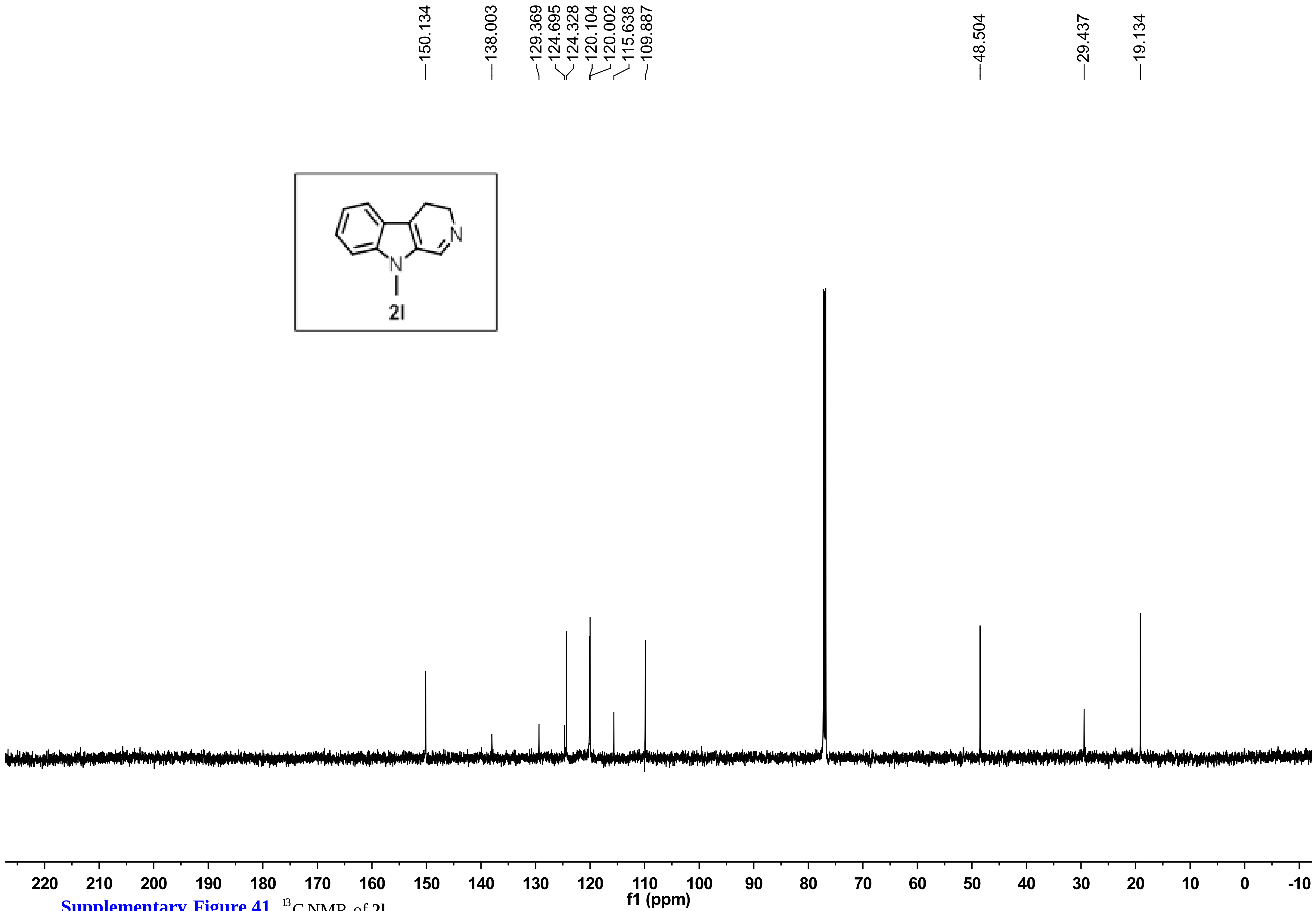

Supplementary Figure 41. ^{13}C NMR of **2l**

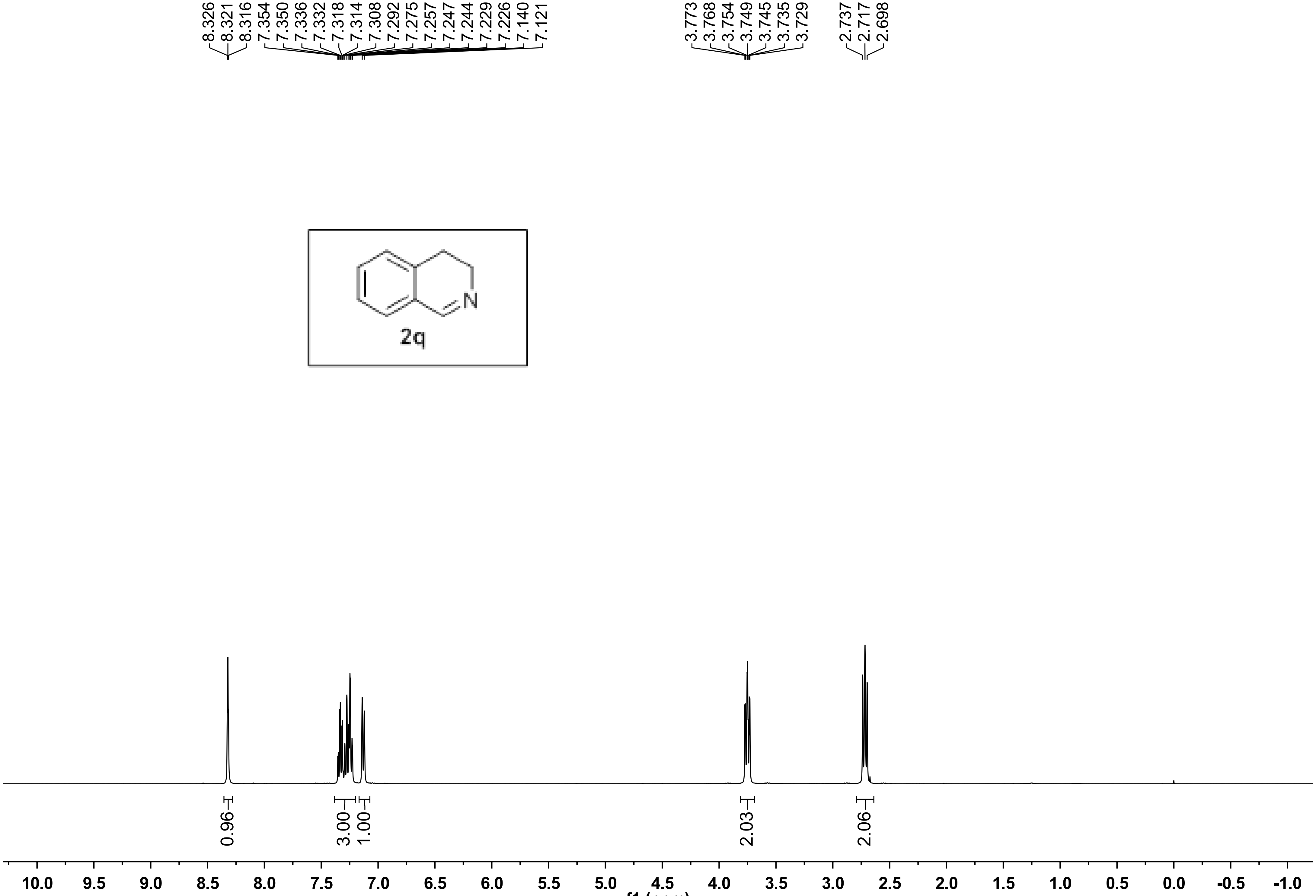

Supplementary Figure 42. ^{1}H NMR of **2q**

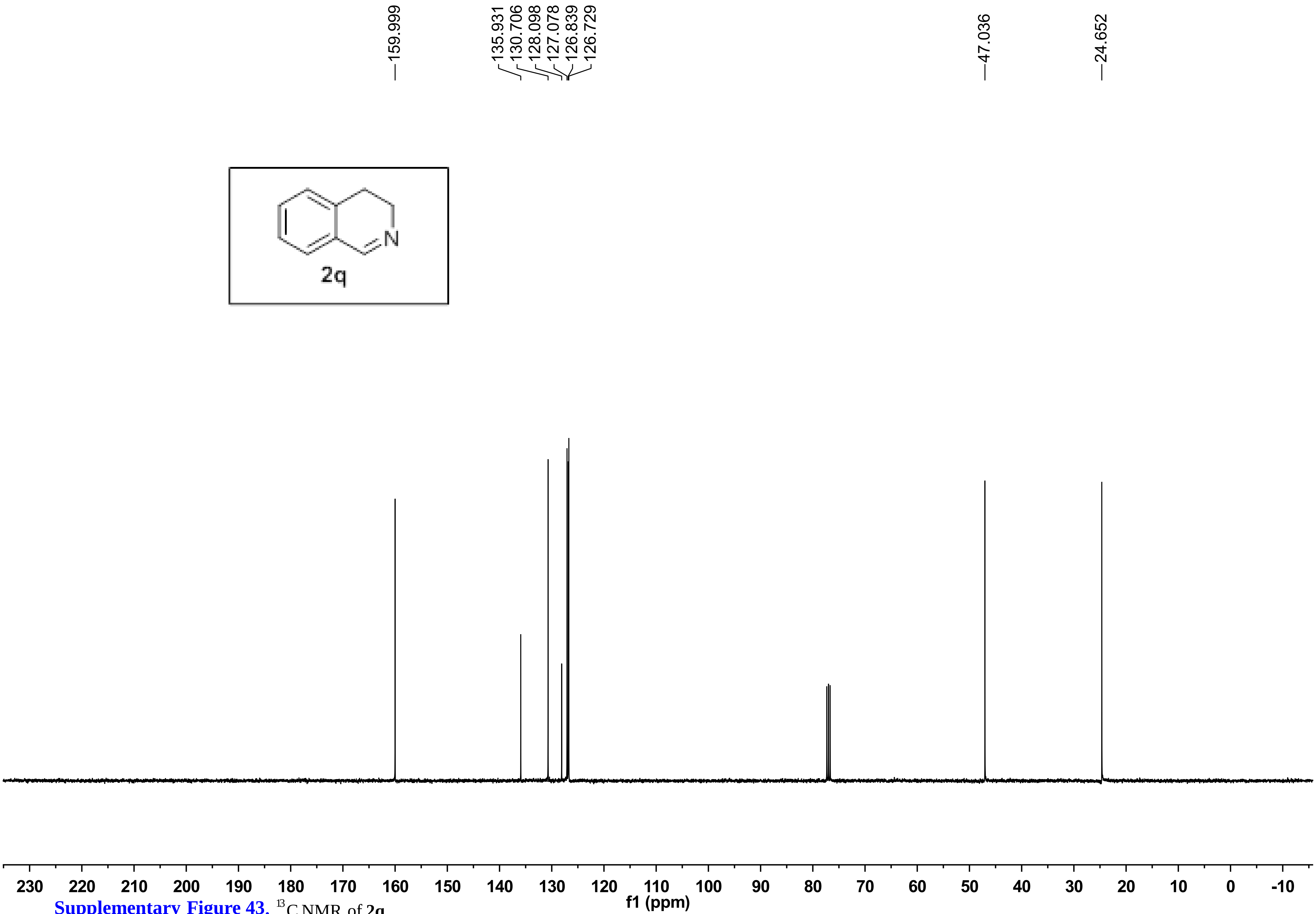

Supplementary Figure 43. ^{13}C NMR of **2q**

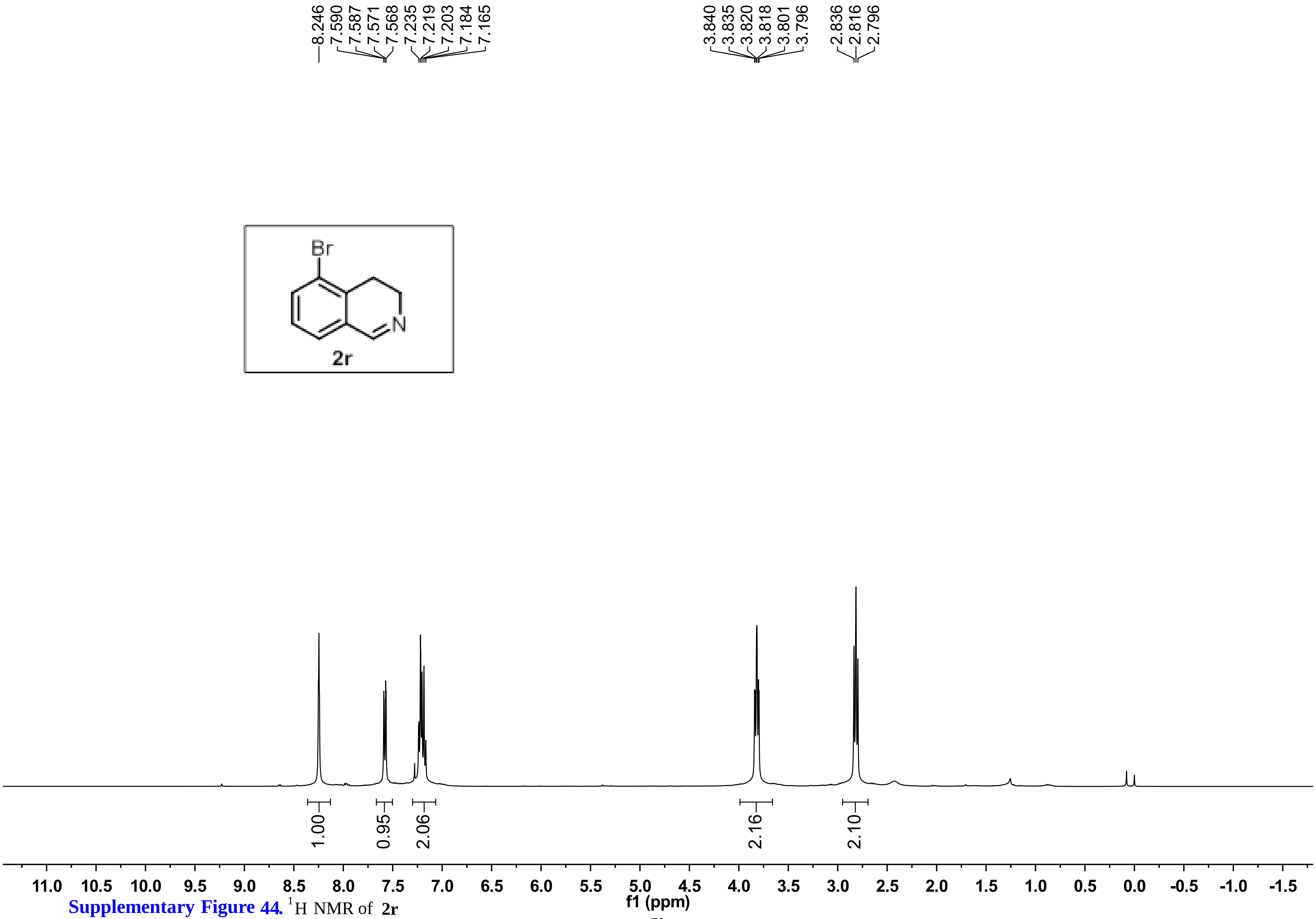

Supplementary Figure 44. ^{1}H NMR of **2r**

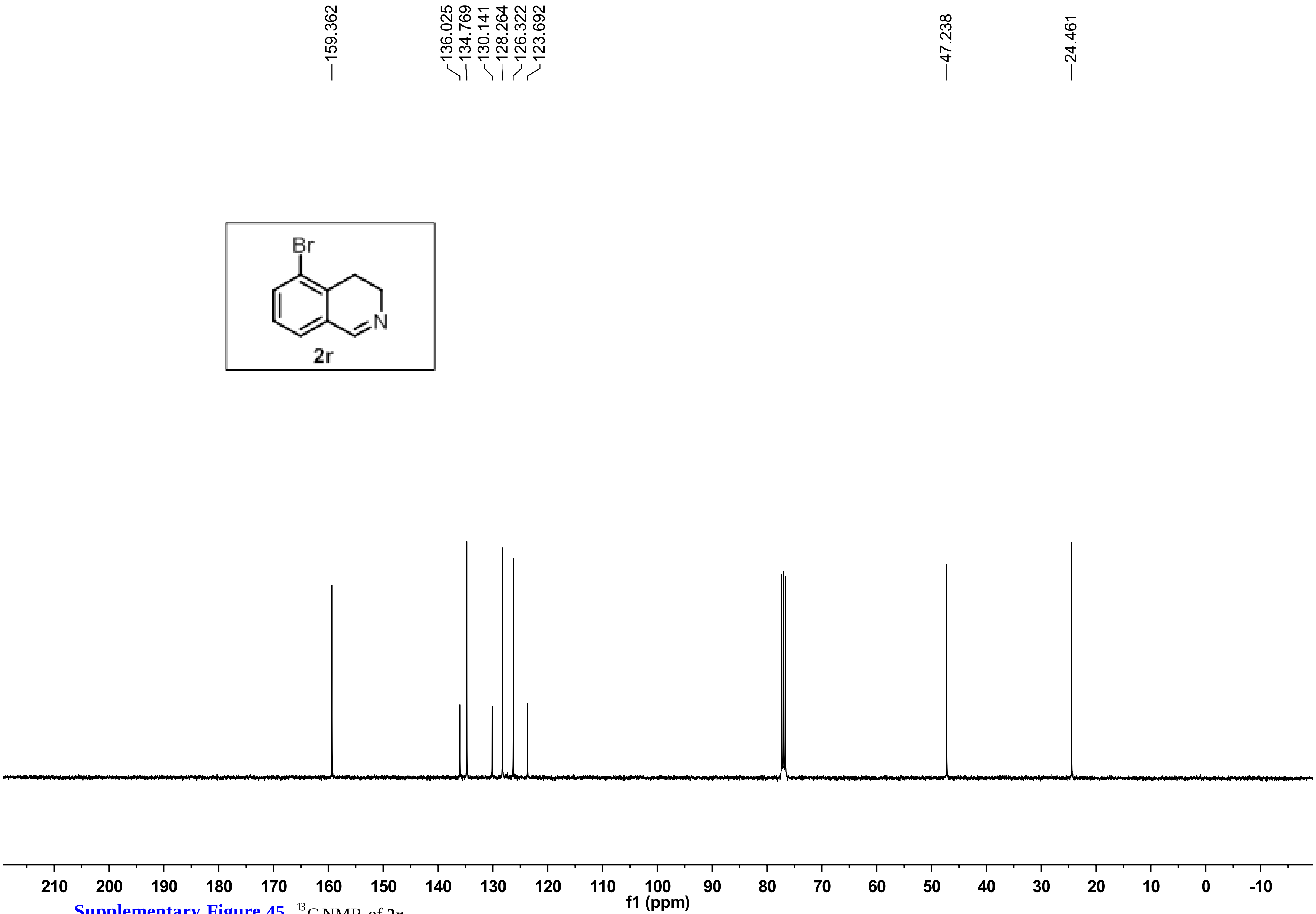

Supplementary Figure 45. ^{13}C NMR of **2r**

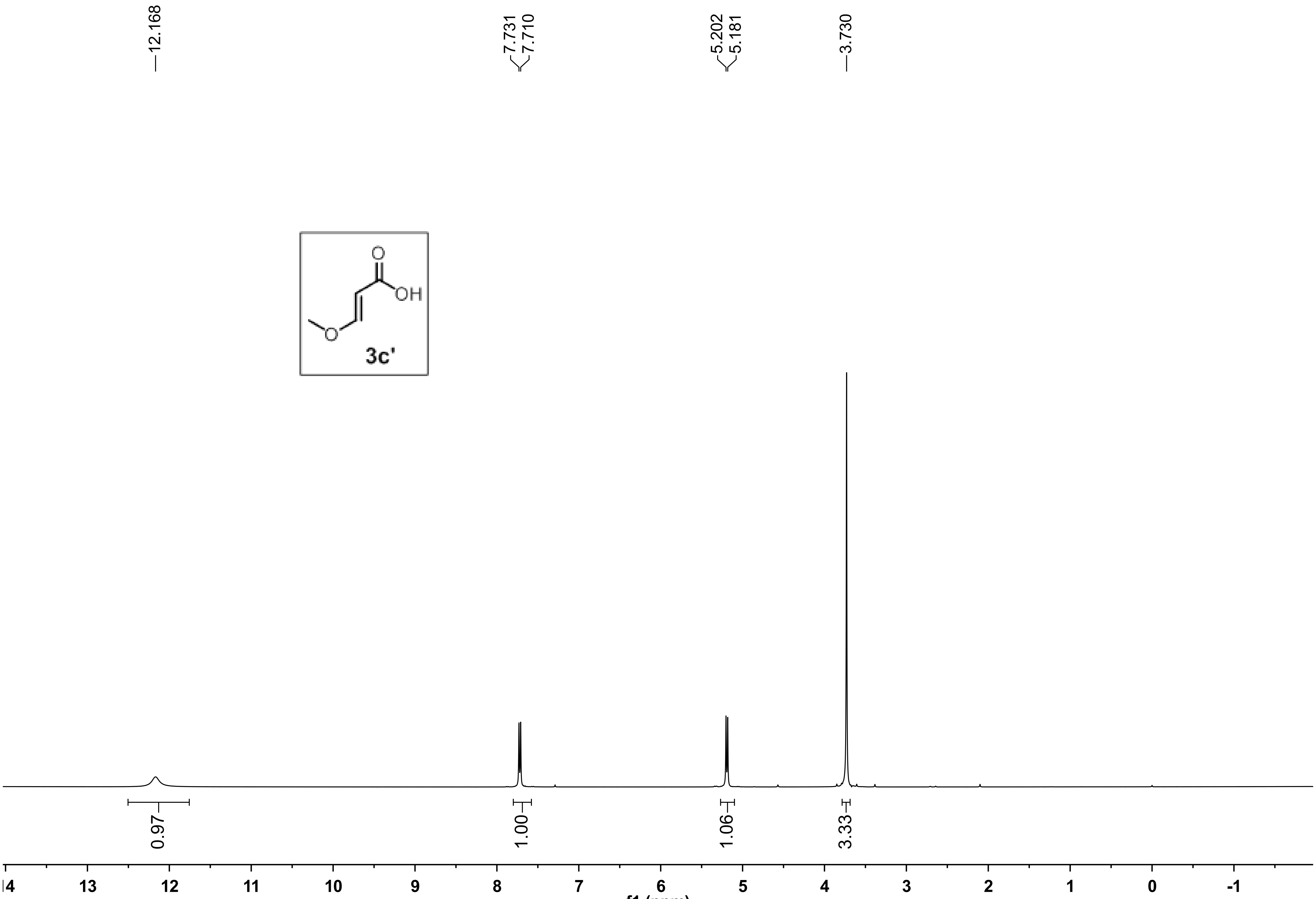

Supplementary Figure 46. ^{1}H NMR of **3c'**

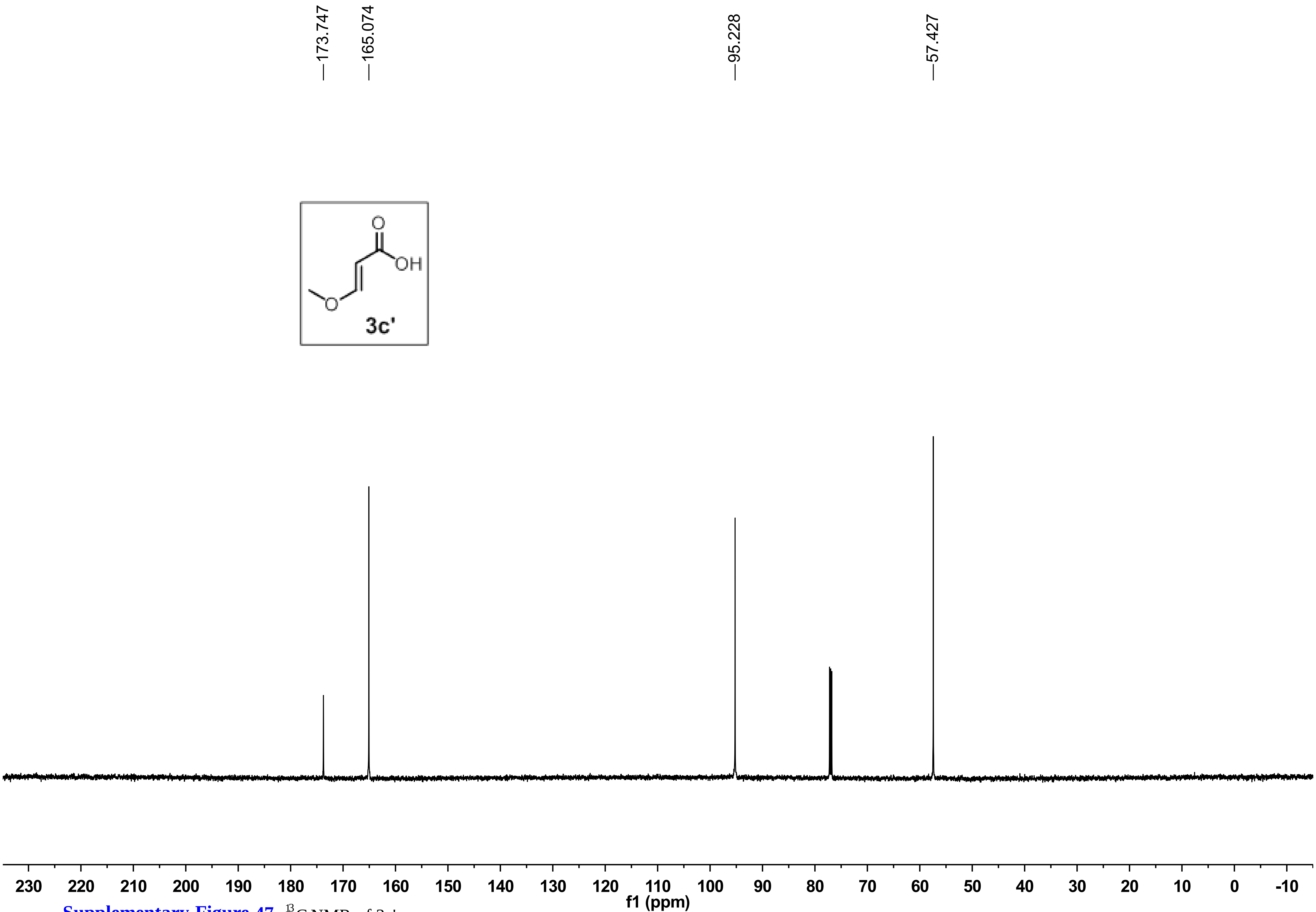

Supplementary Figure 47. ^{13}C NMR of 3c'

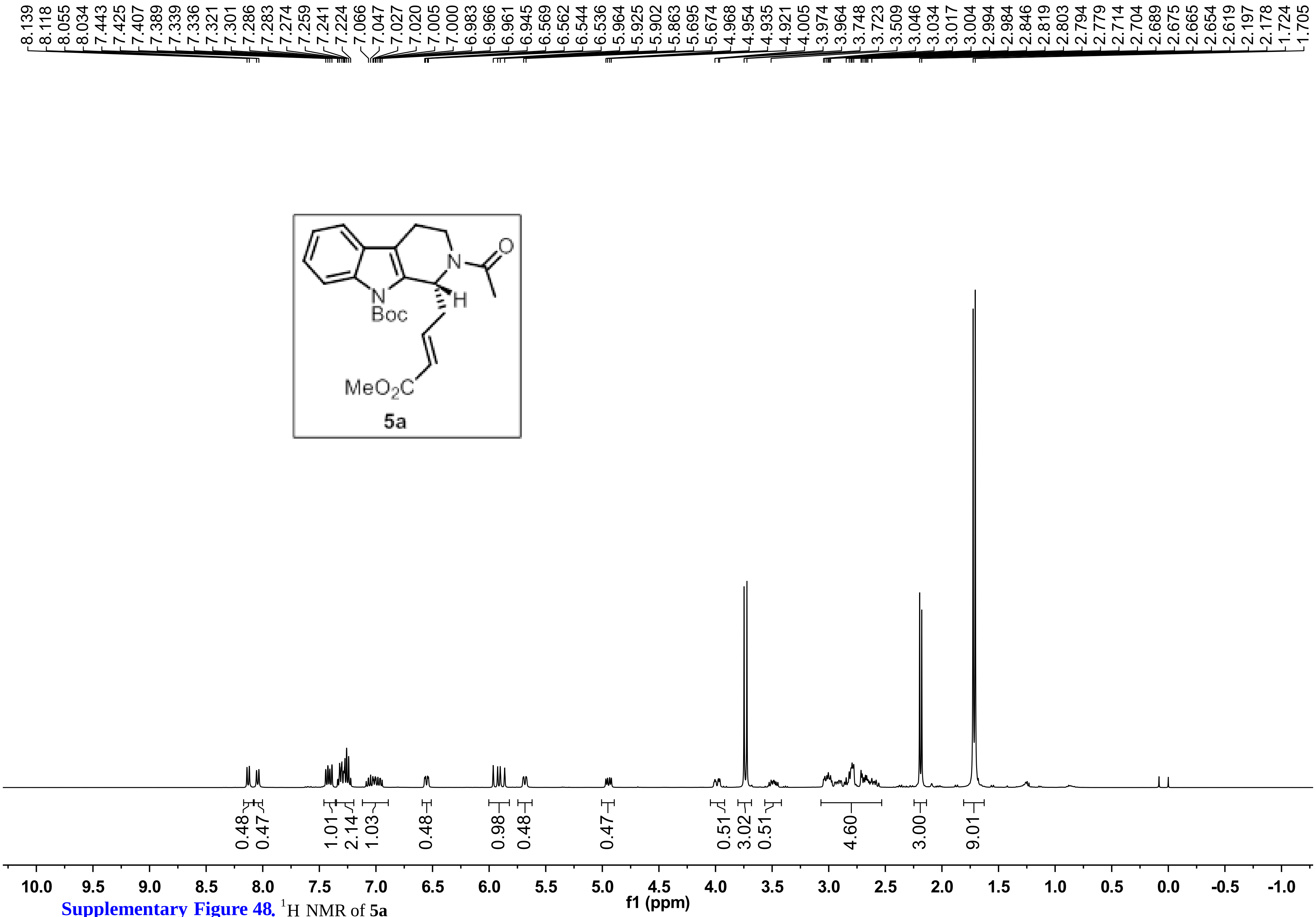

Supplementary Figure 48. ^{1}H NMR of **5a**

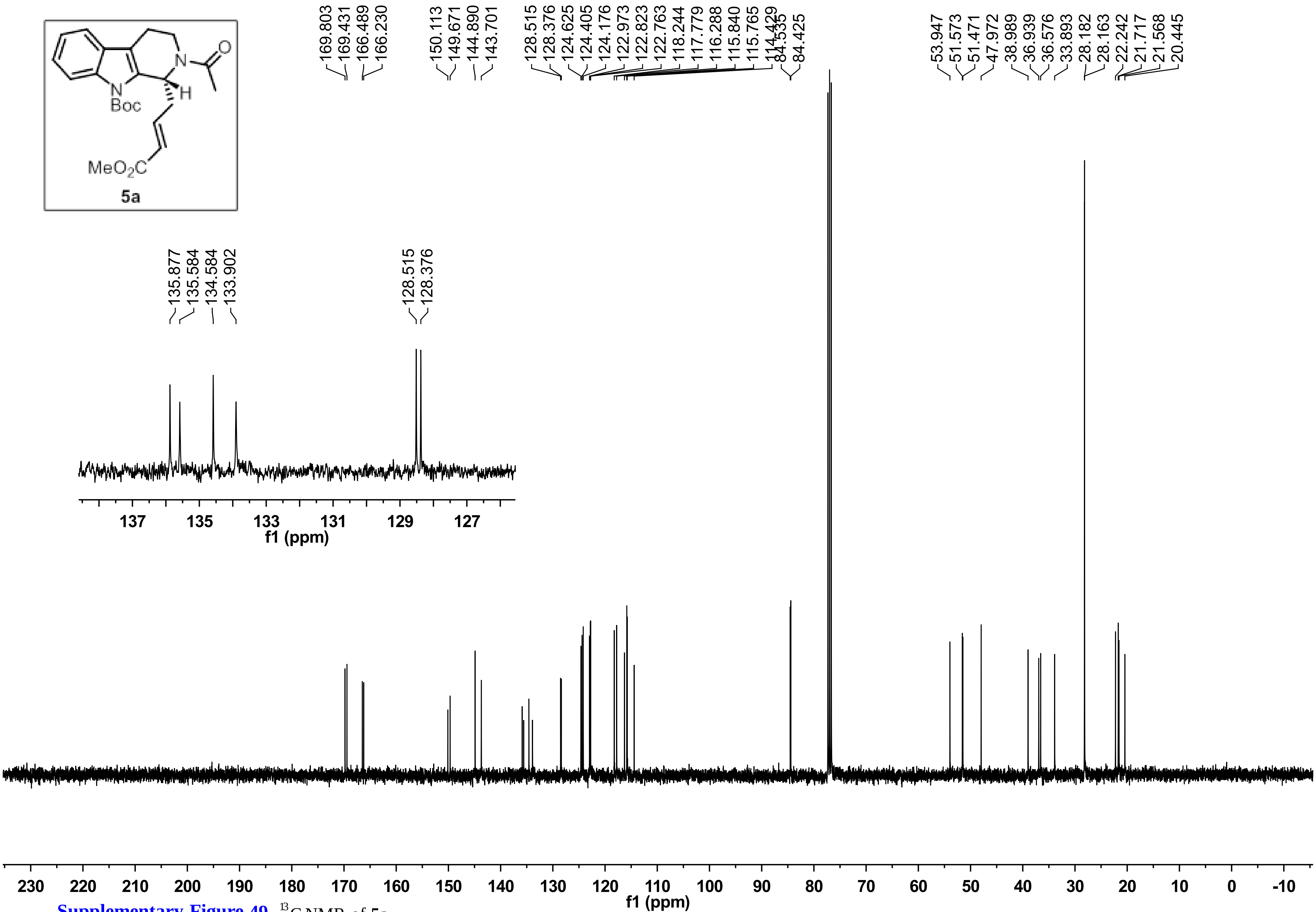

Supplementary Figure 49. ^{13}C NMR of **5a**

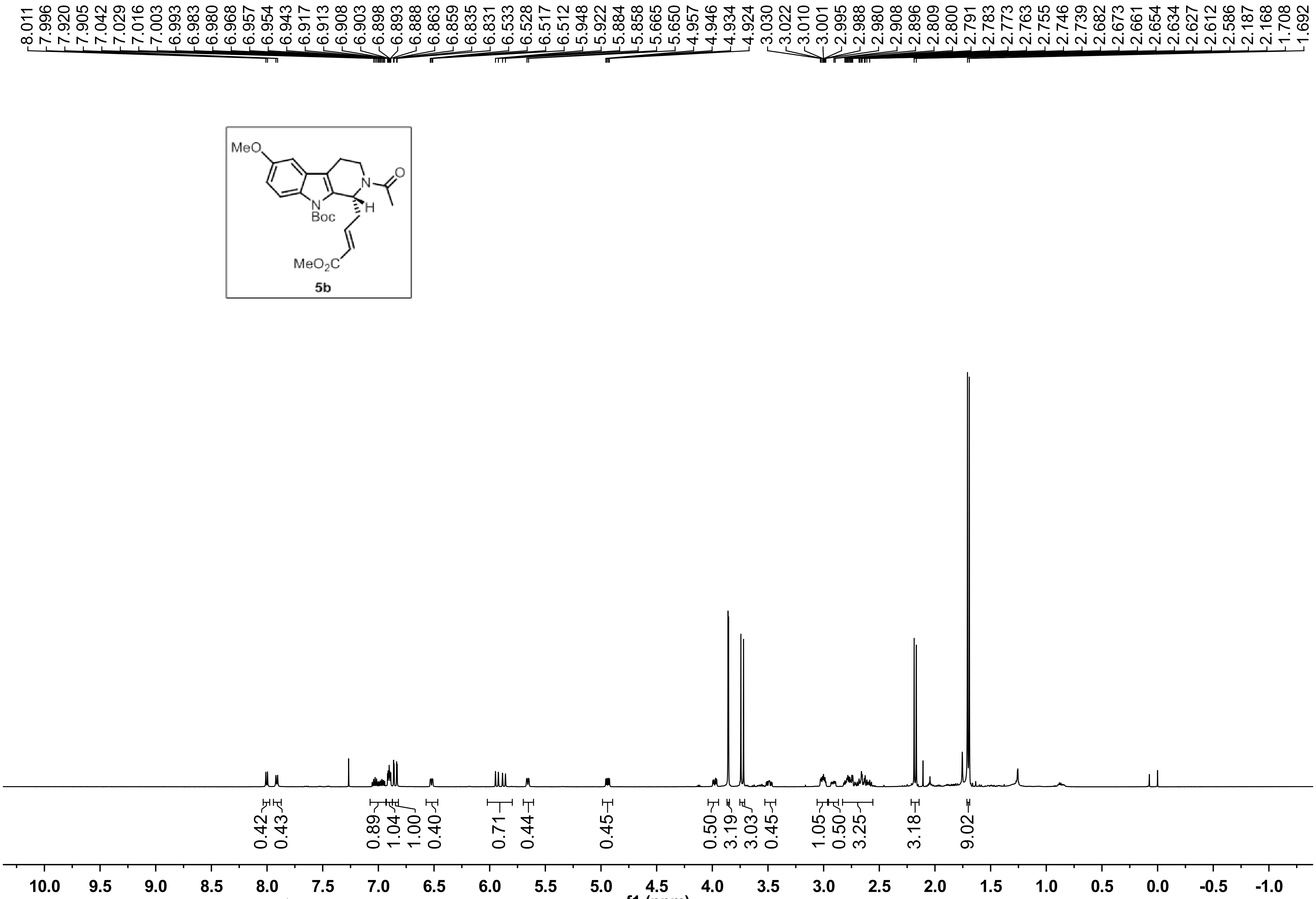

Supplementary Figure 50. ^{1}H NMR of **5b**

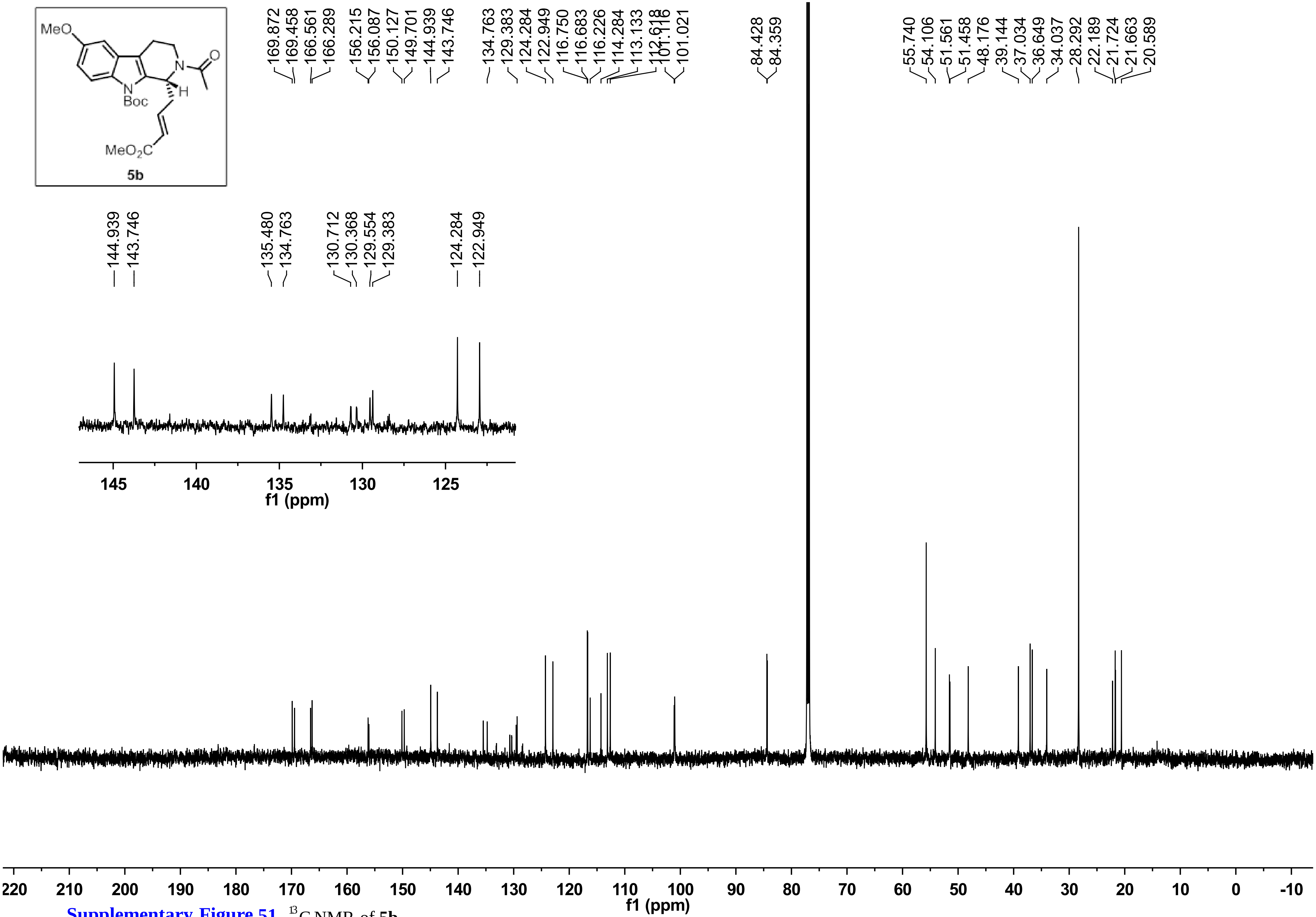

Supplementary Figure 51. ^{13}C NMR of **5b**

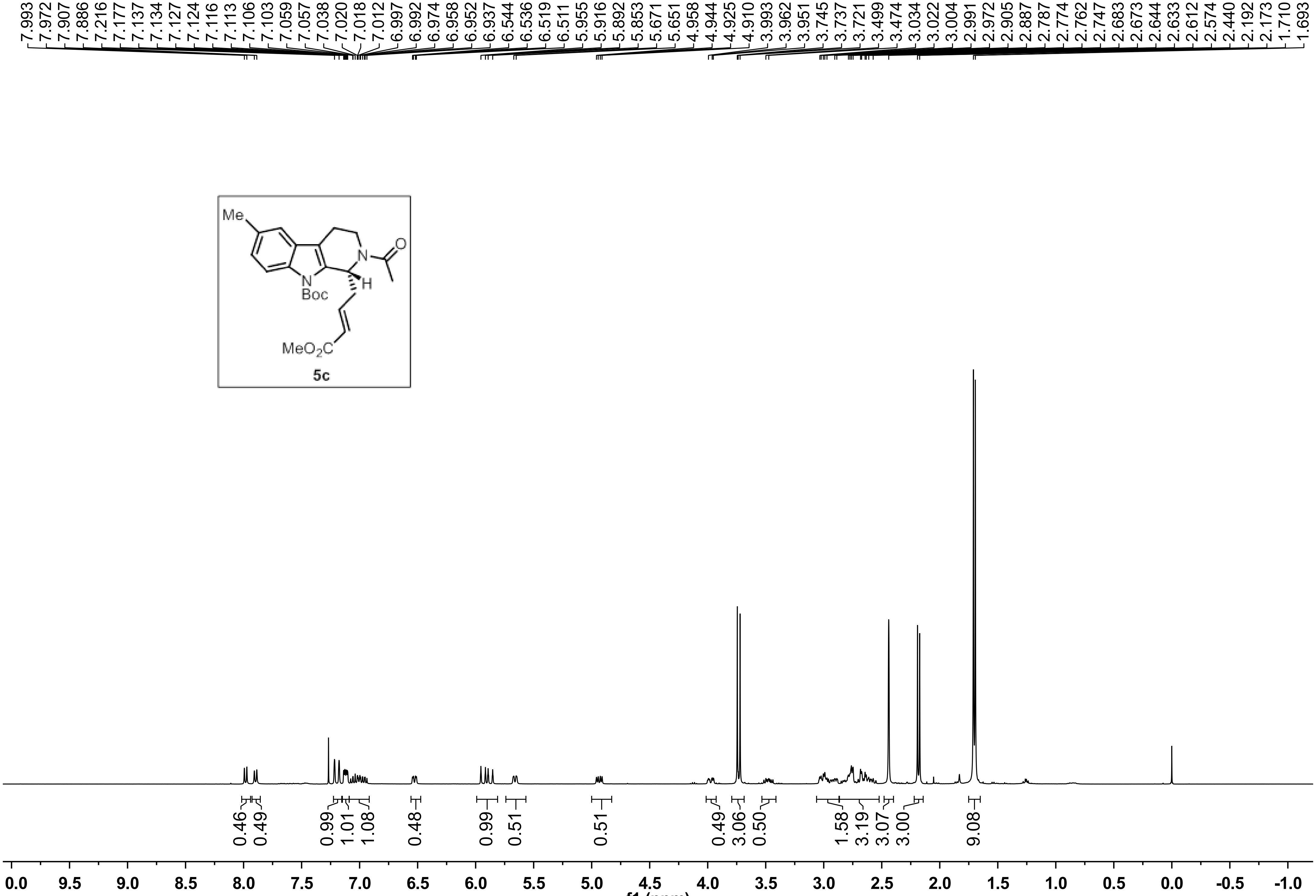

Supplementary Figure 52. ^{1}H NMR of **5c**

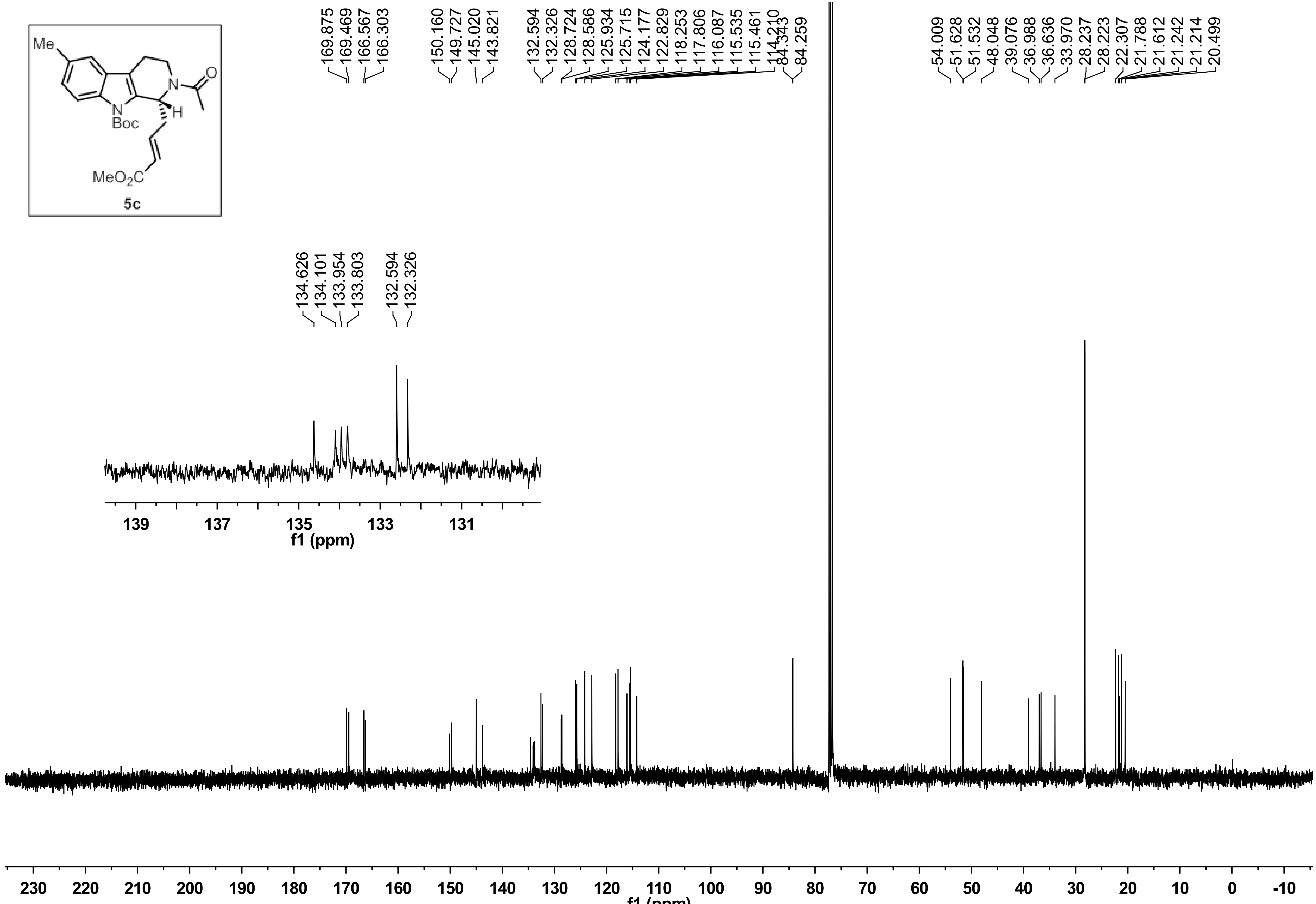

Supplementary Figure 53. ^{13}C NMR of **5c**

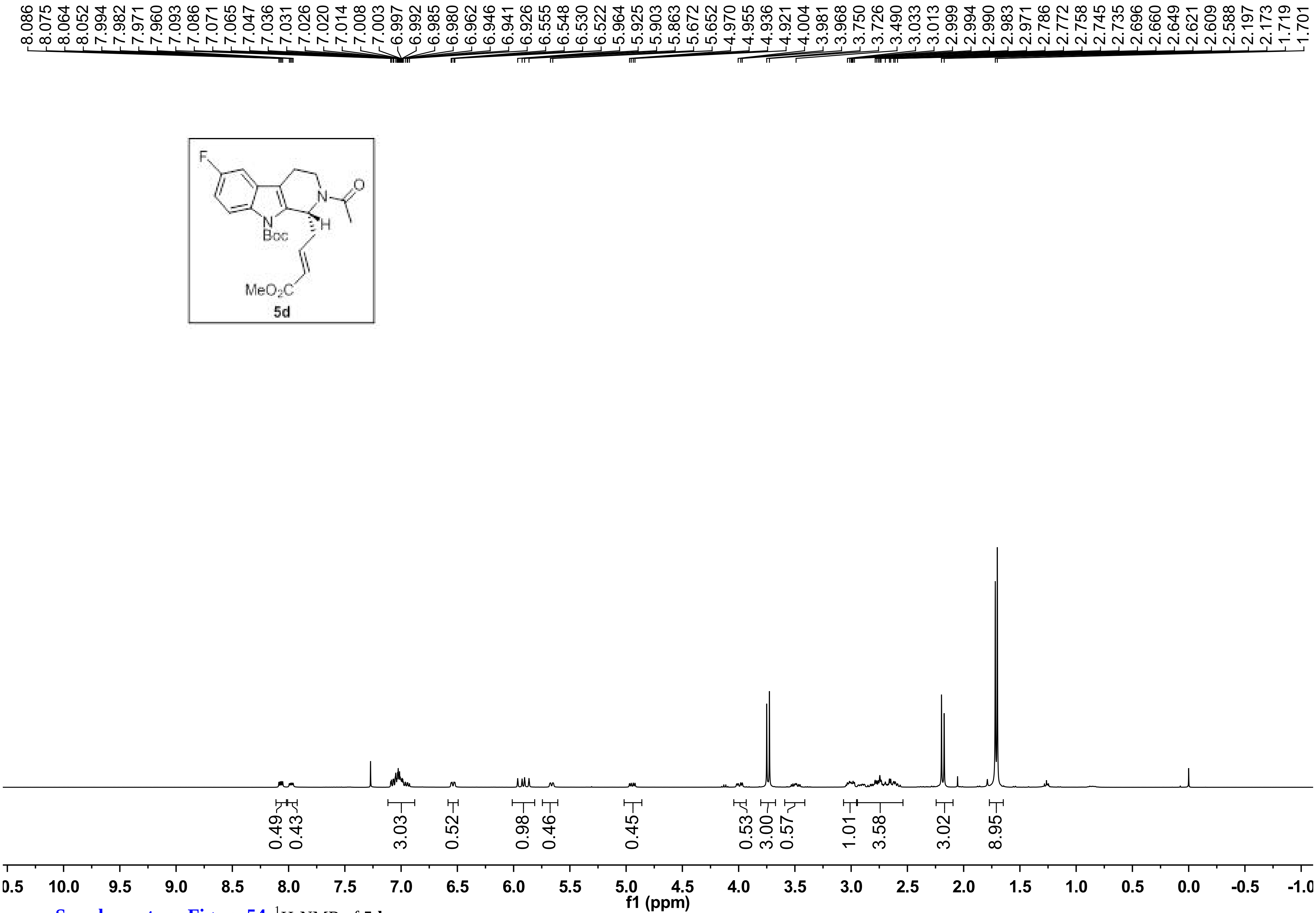

Supplementary Figure 54. ^{1}H NMR of **5d**

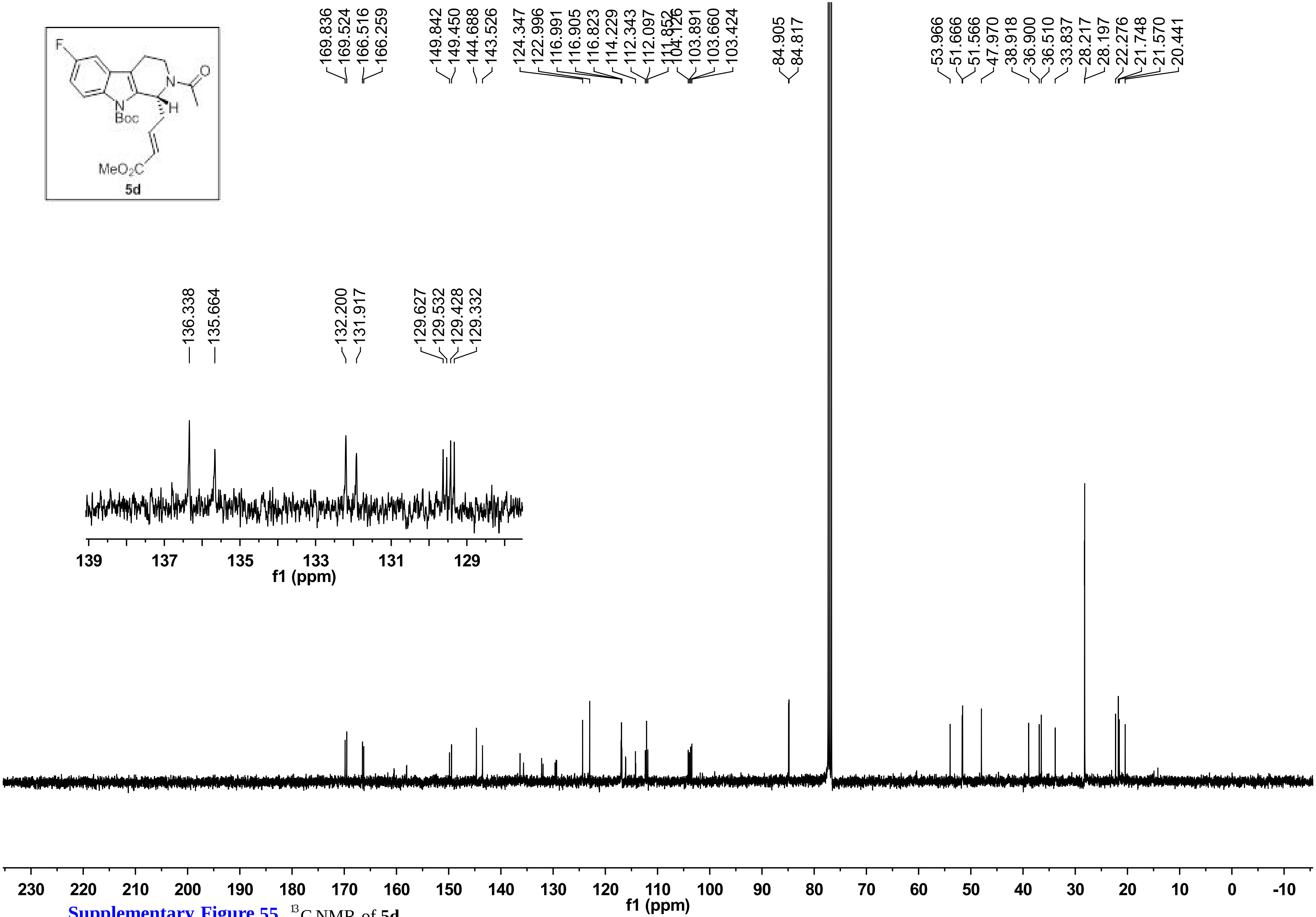

Supplementary Figure 55. ^{13}C NMR of **5d**

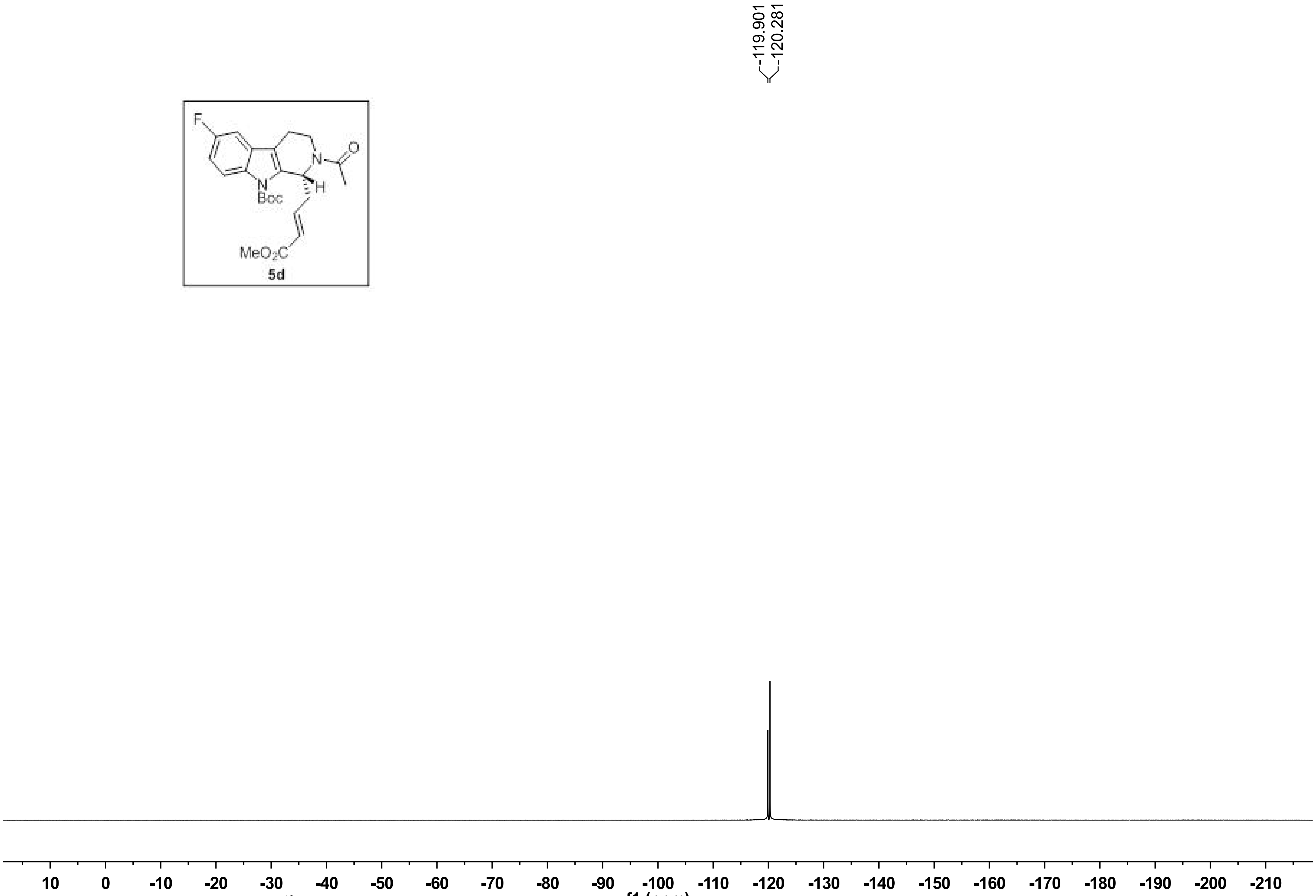

Supplementary Figure 56. ^{19}F NMR of **2h**

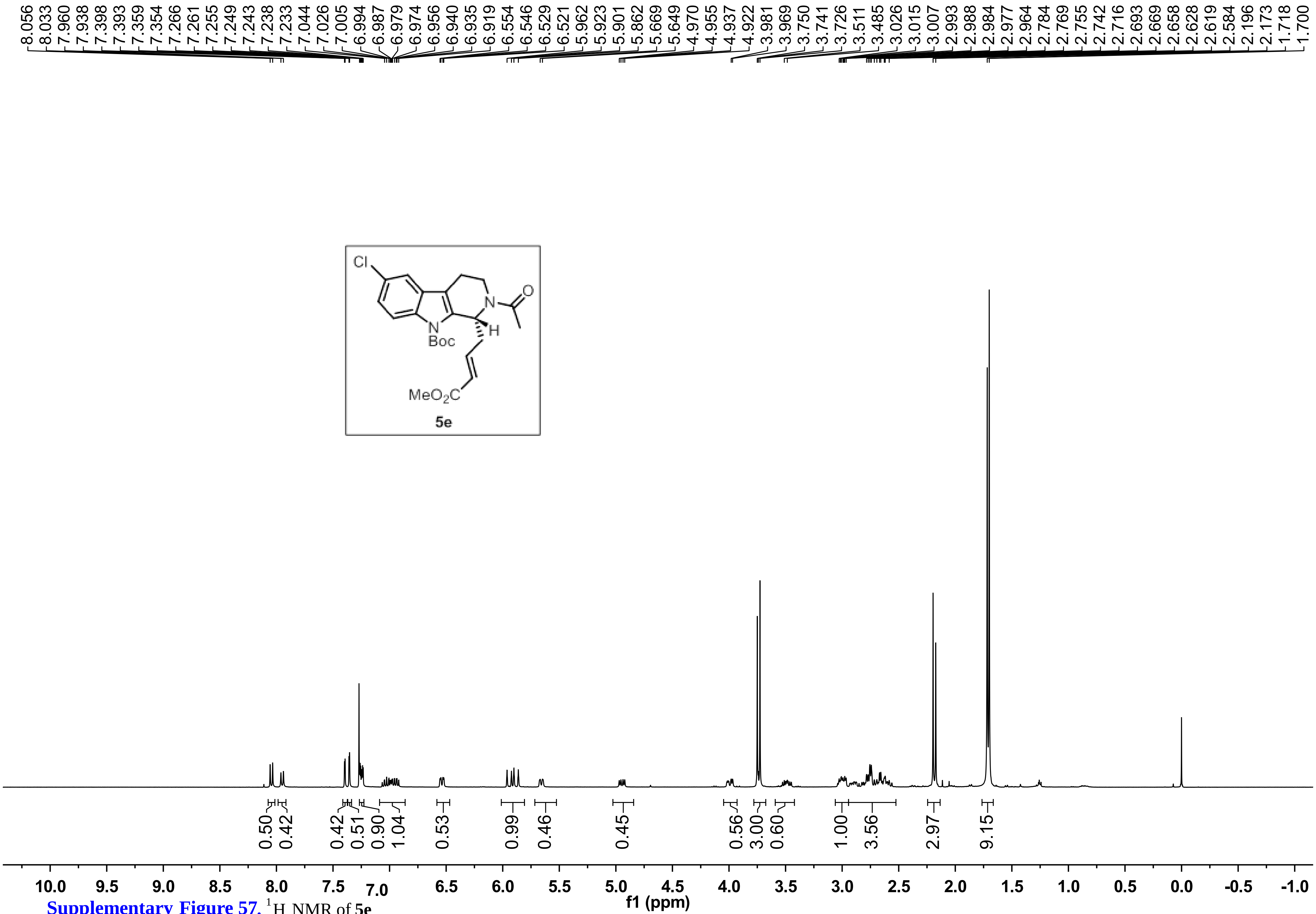

Supplementary Figure 57. ^{1}H NMR of **5e**

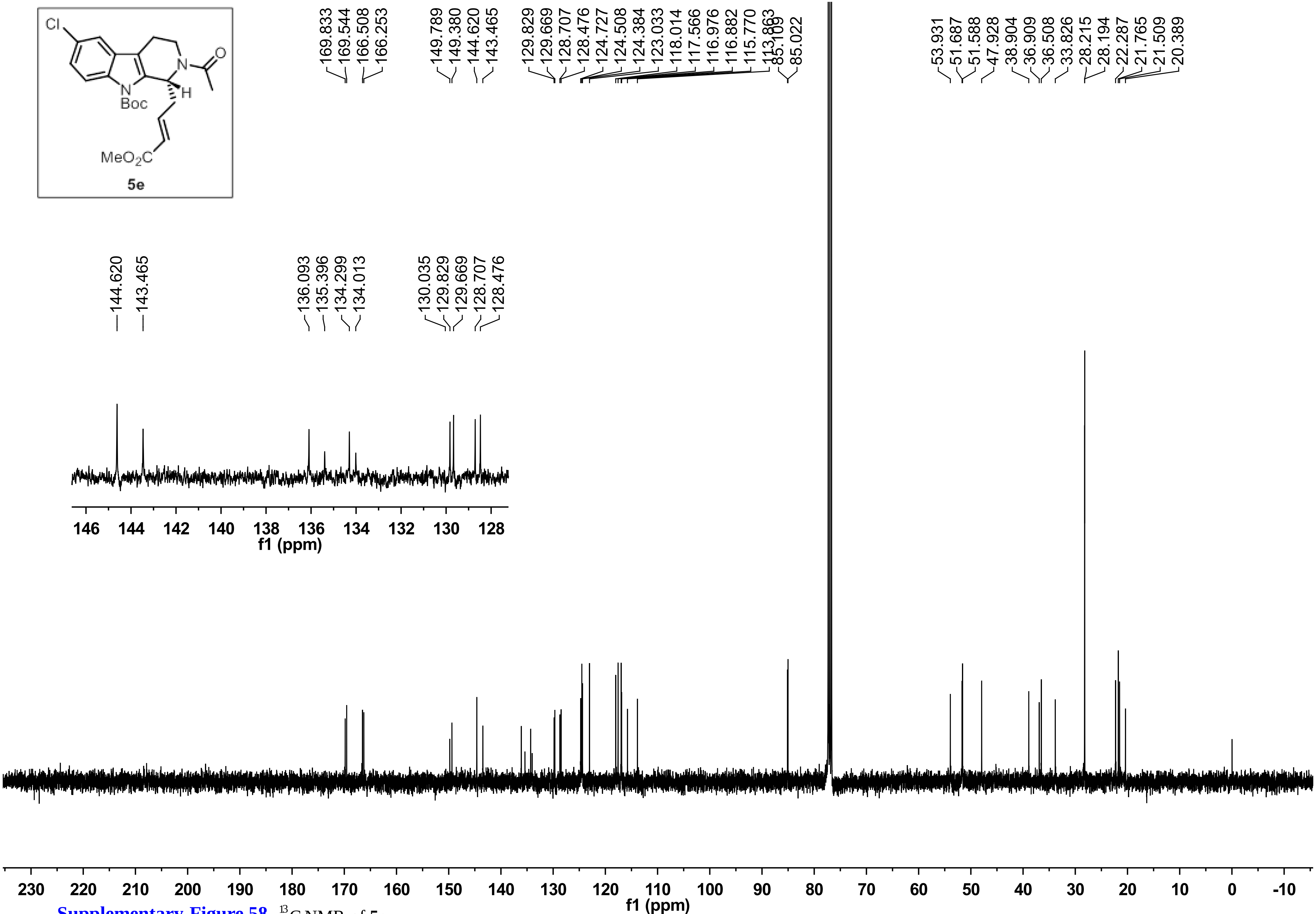

Supplementary Figure 58. ^{13}C NMR of **5e**

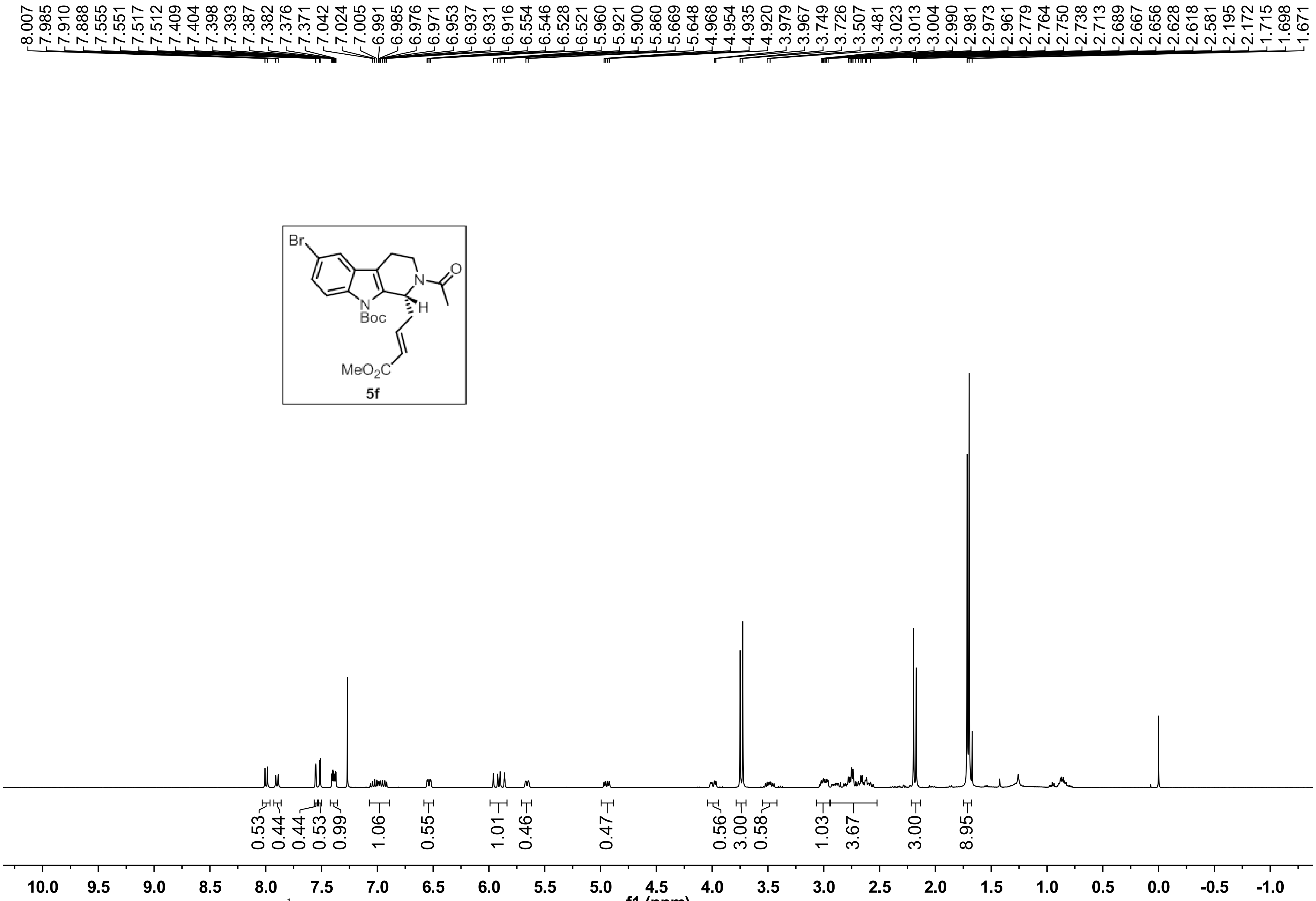

Supplementary Figure 59. ^{1}H NMR of **5f**

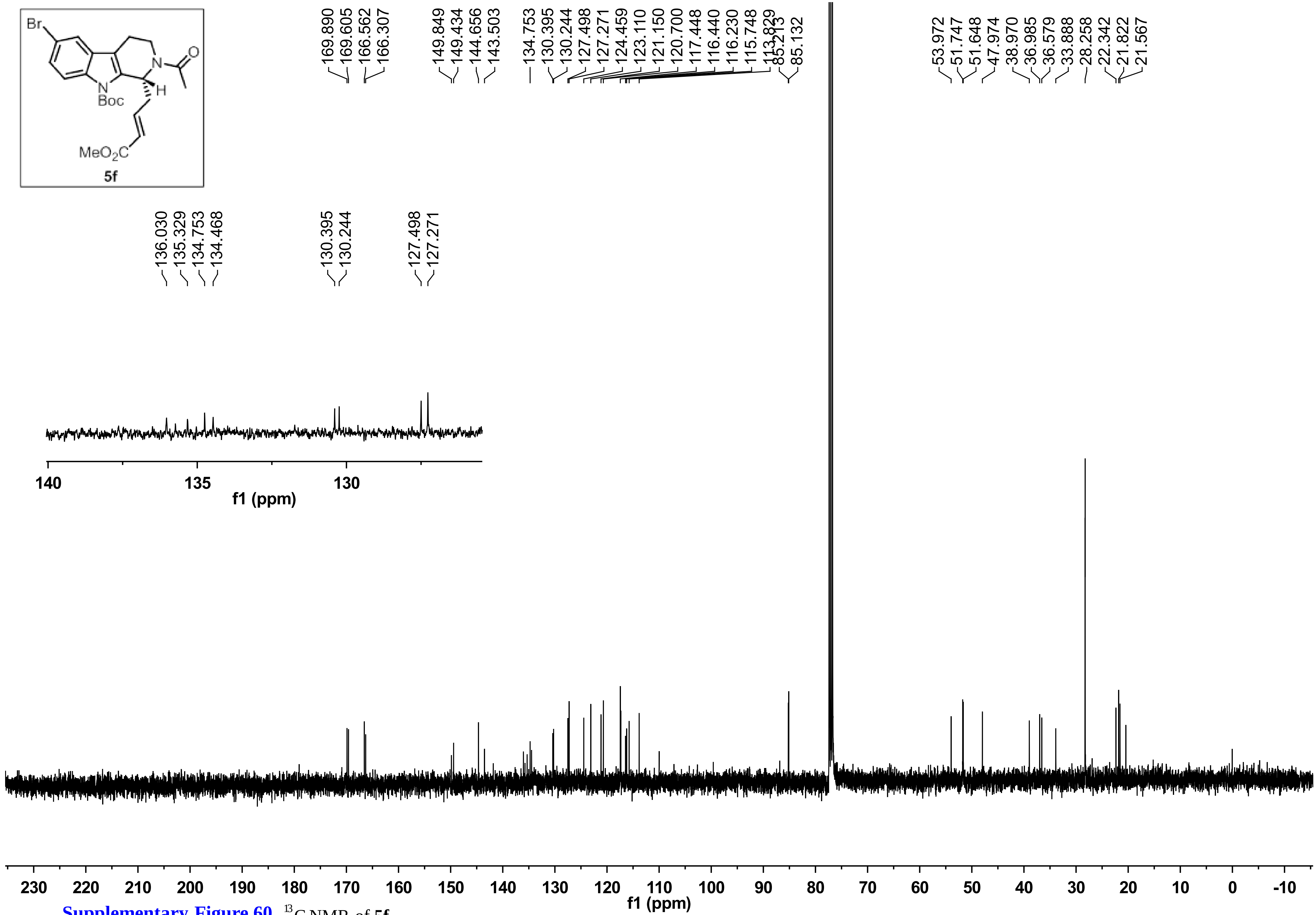

Supplementary Figure 60. ^{13}C NMR of **5f**

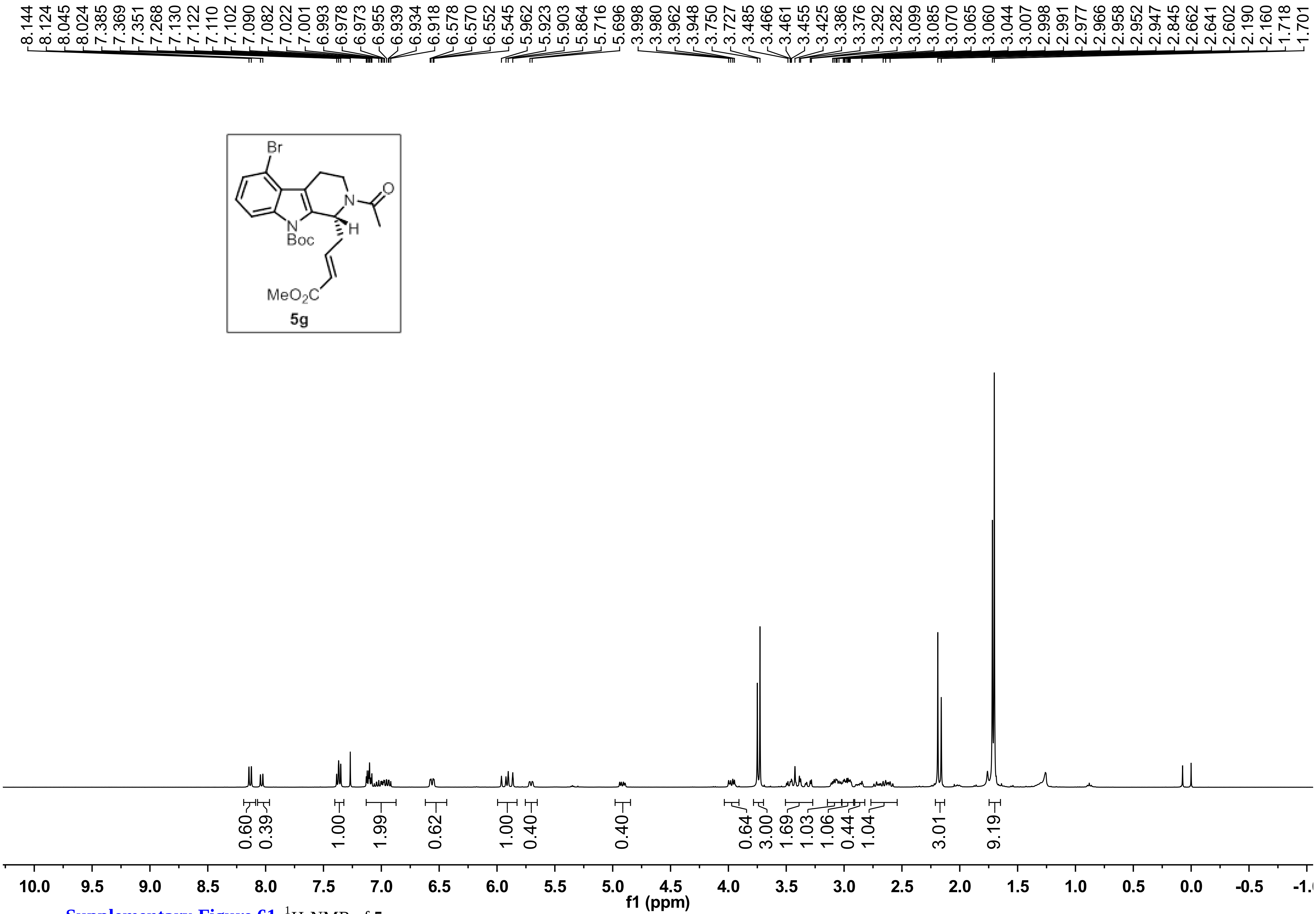

Supplementary Figure 61. ^{1}H NMR of **5g**

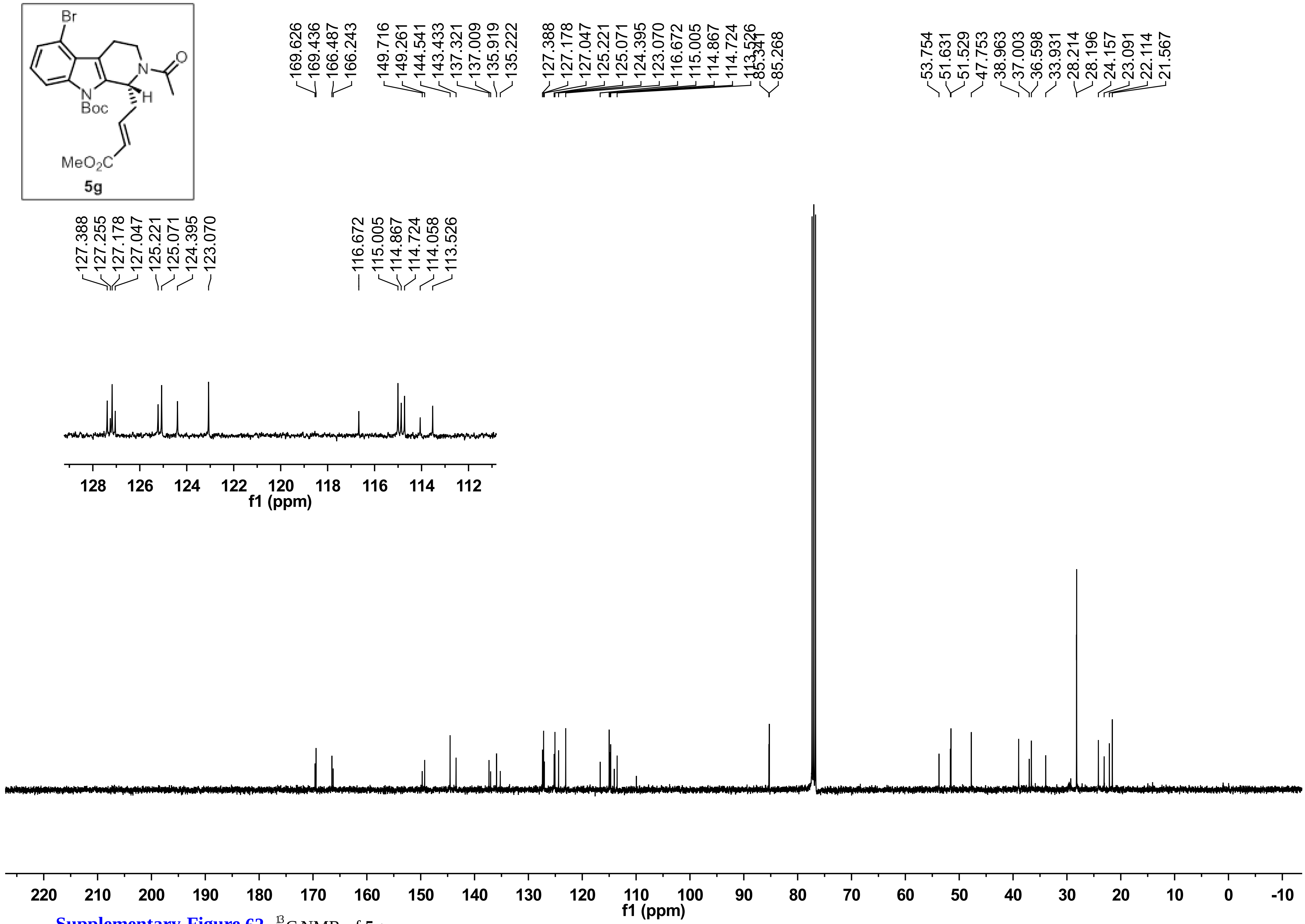

Supplementary Figure 62. ^{13}C NMR of **5g**

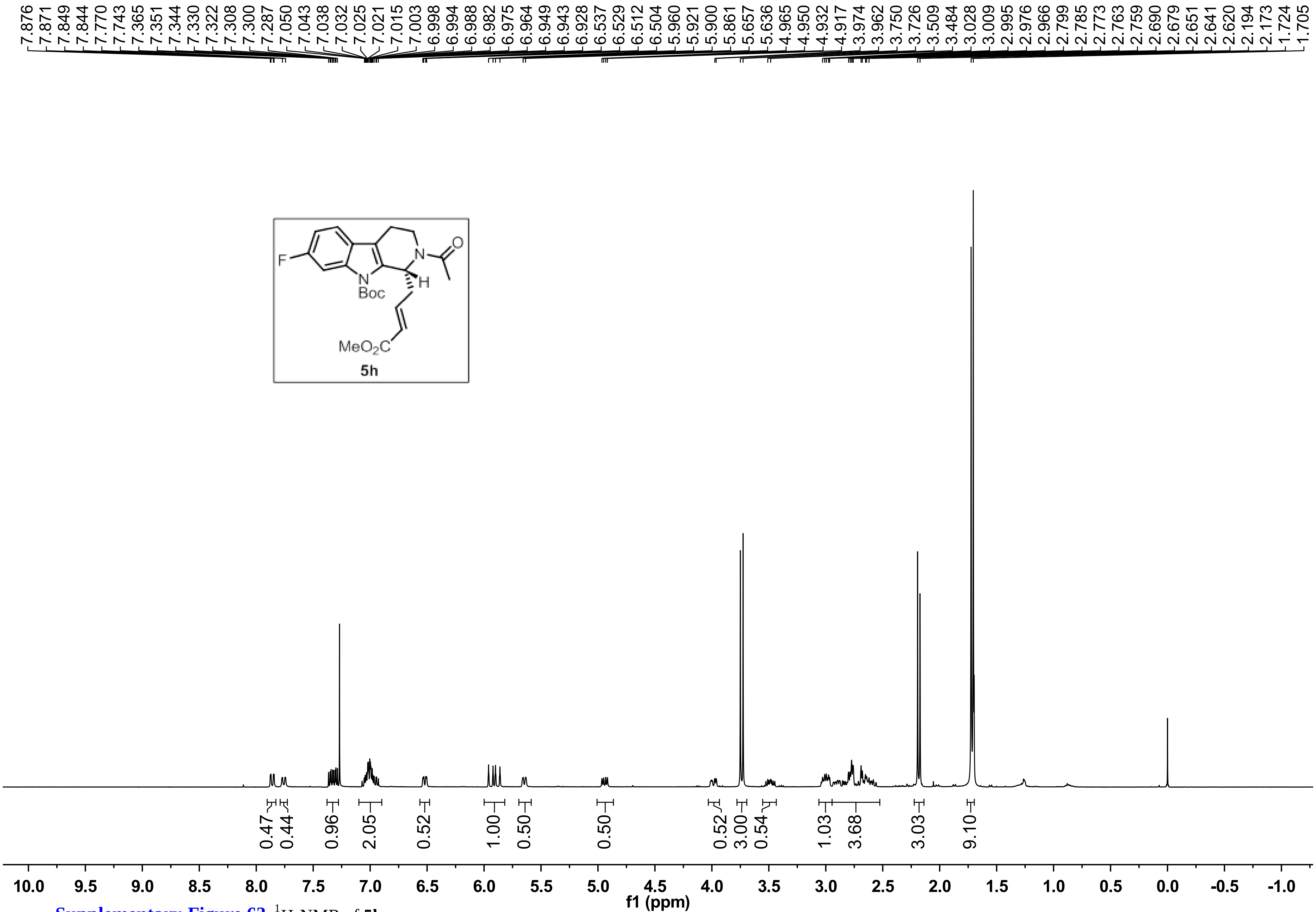

Supplementary Figure 63. ^{1}H NMR of **5h**

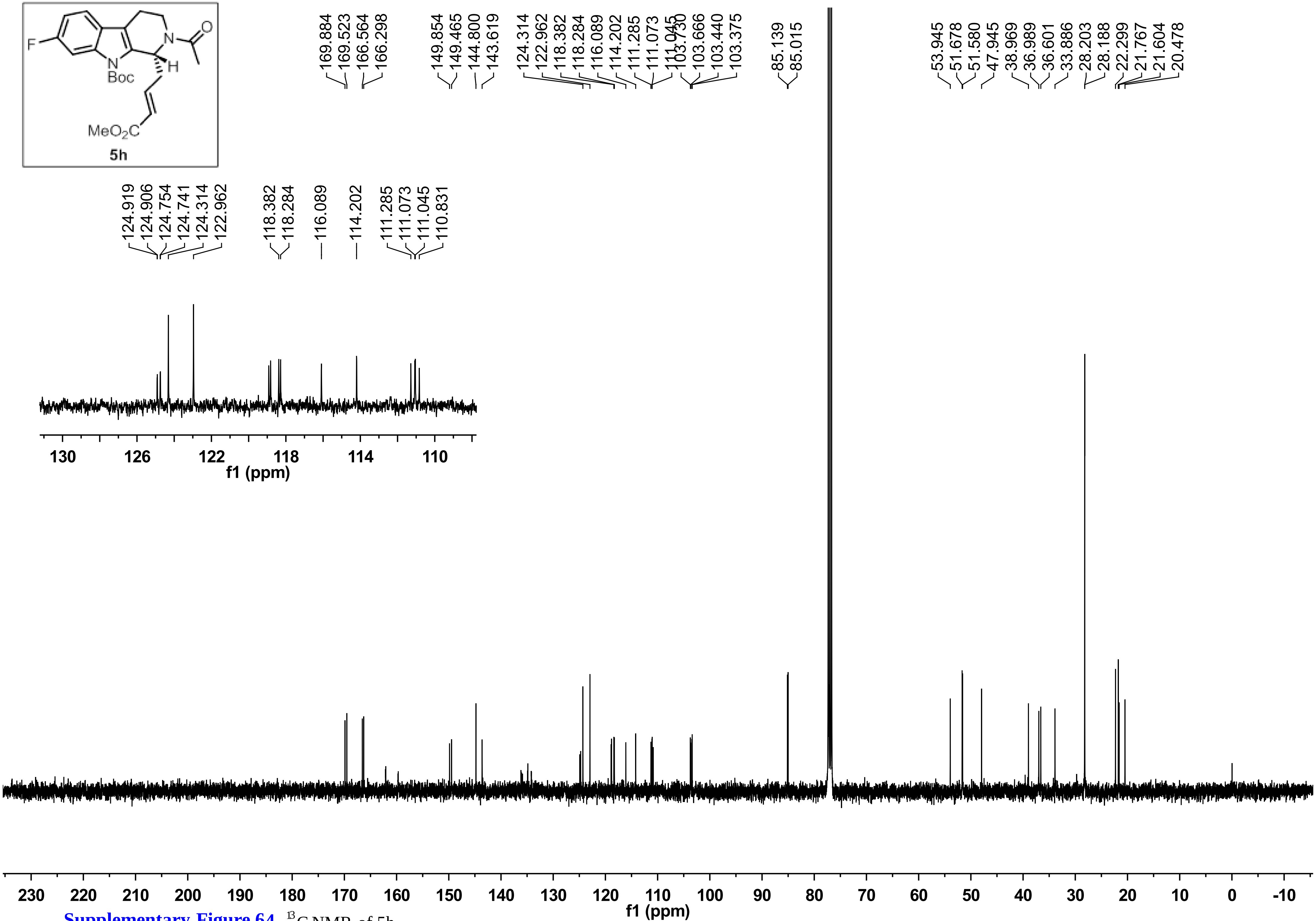

Supplementary Figure 64. ^{13}C NMR of 5h

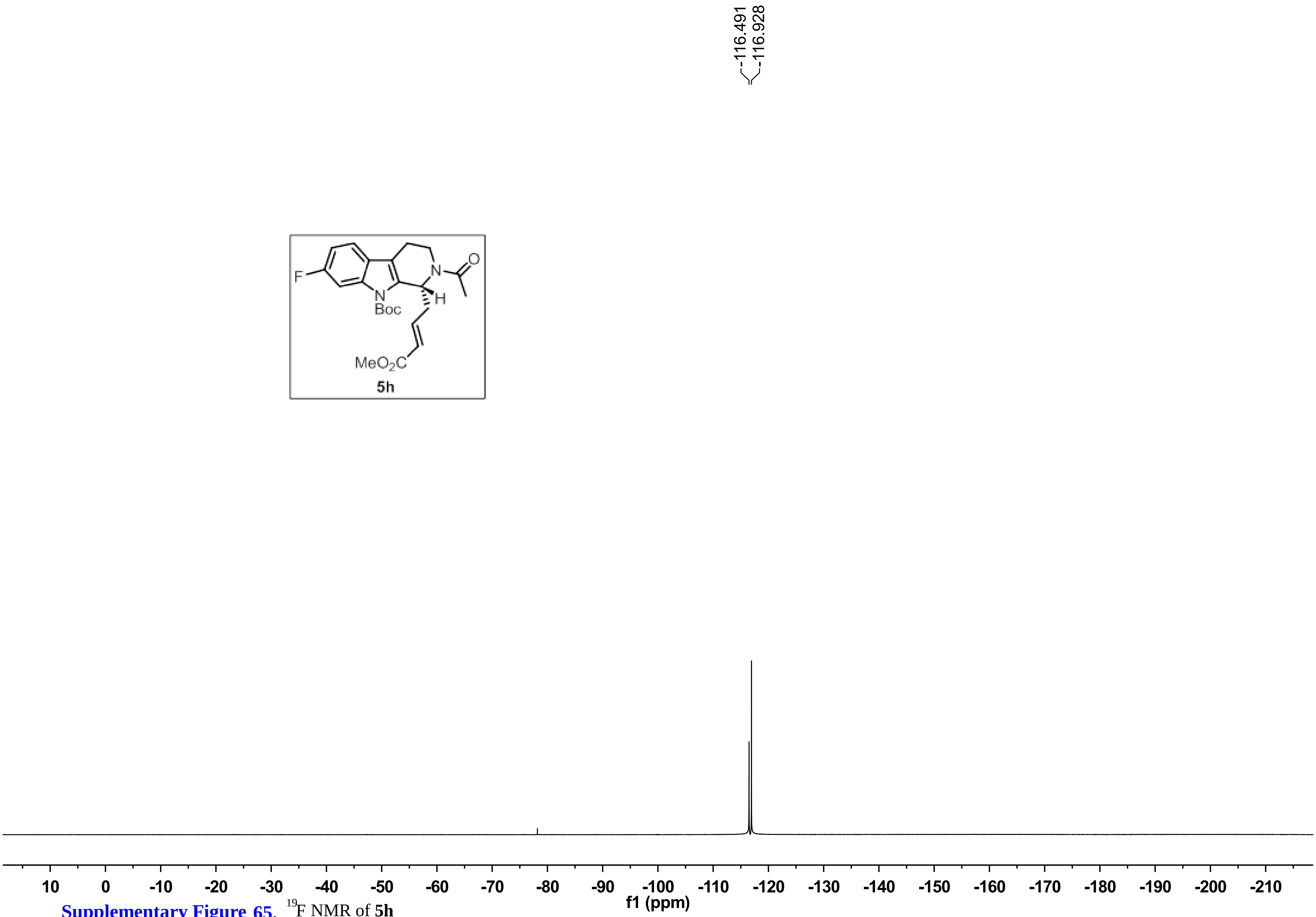

Supplementary Figure 65. ^{19}F NMR of **5h**

5h

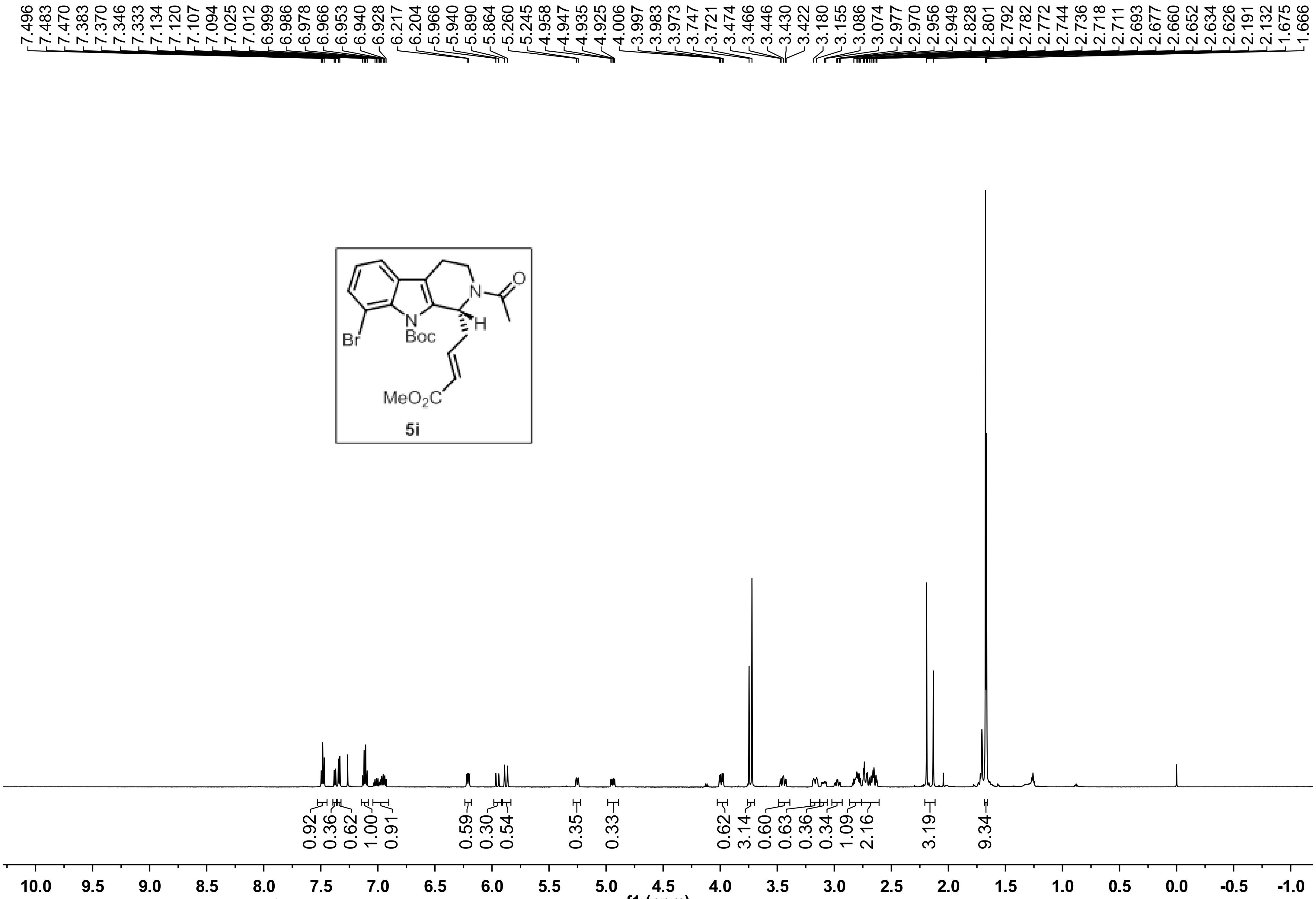

Supplementary Figure 66. ^{1}H NMR of **5i**

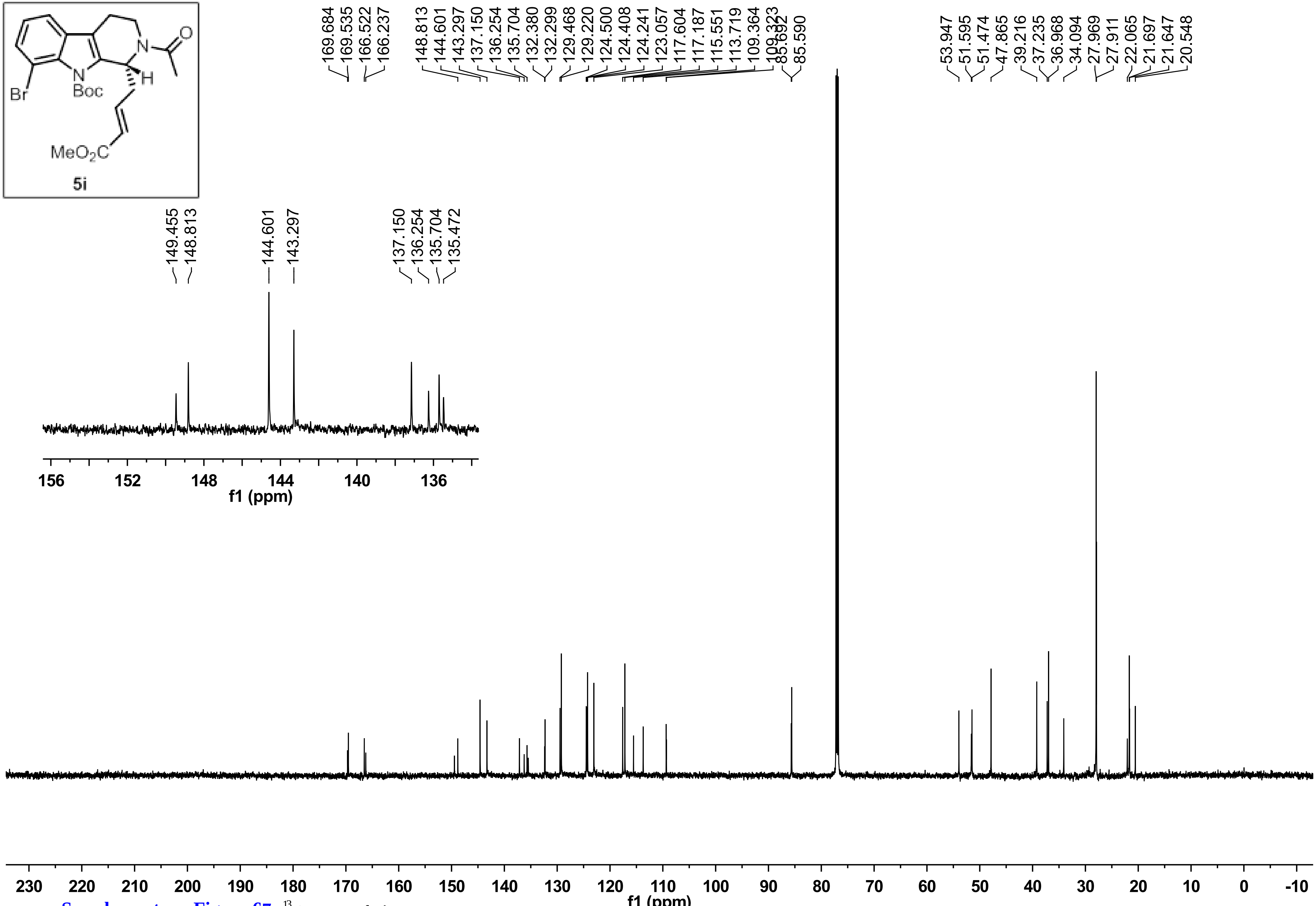

Supplementary Figure 67. ^{13}C NMR of **5i**

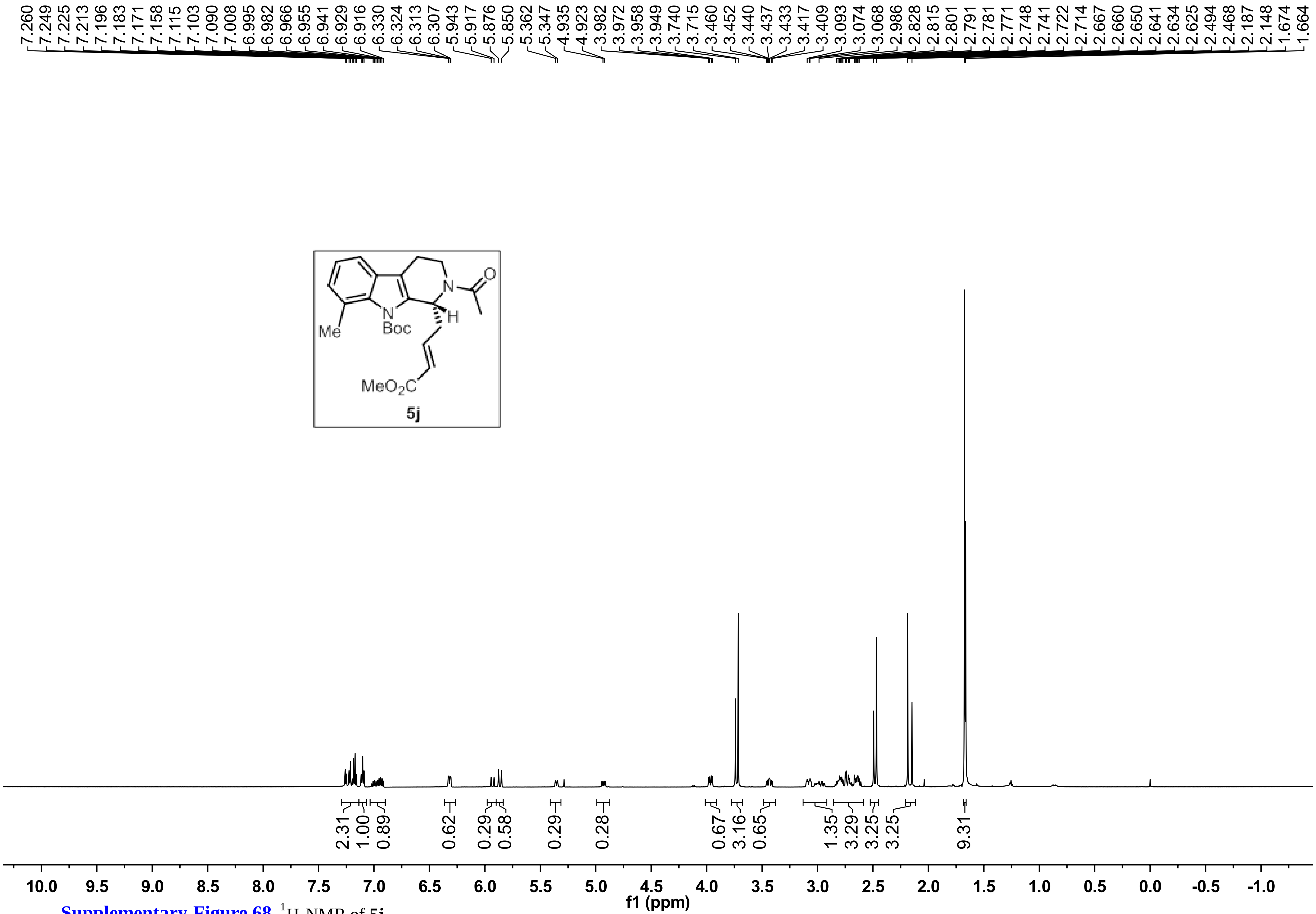

Supplementary Figure 68. ^{1}H NMR of **5j**

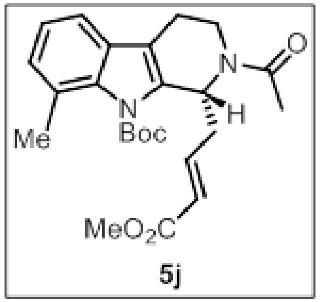

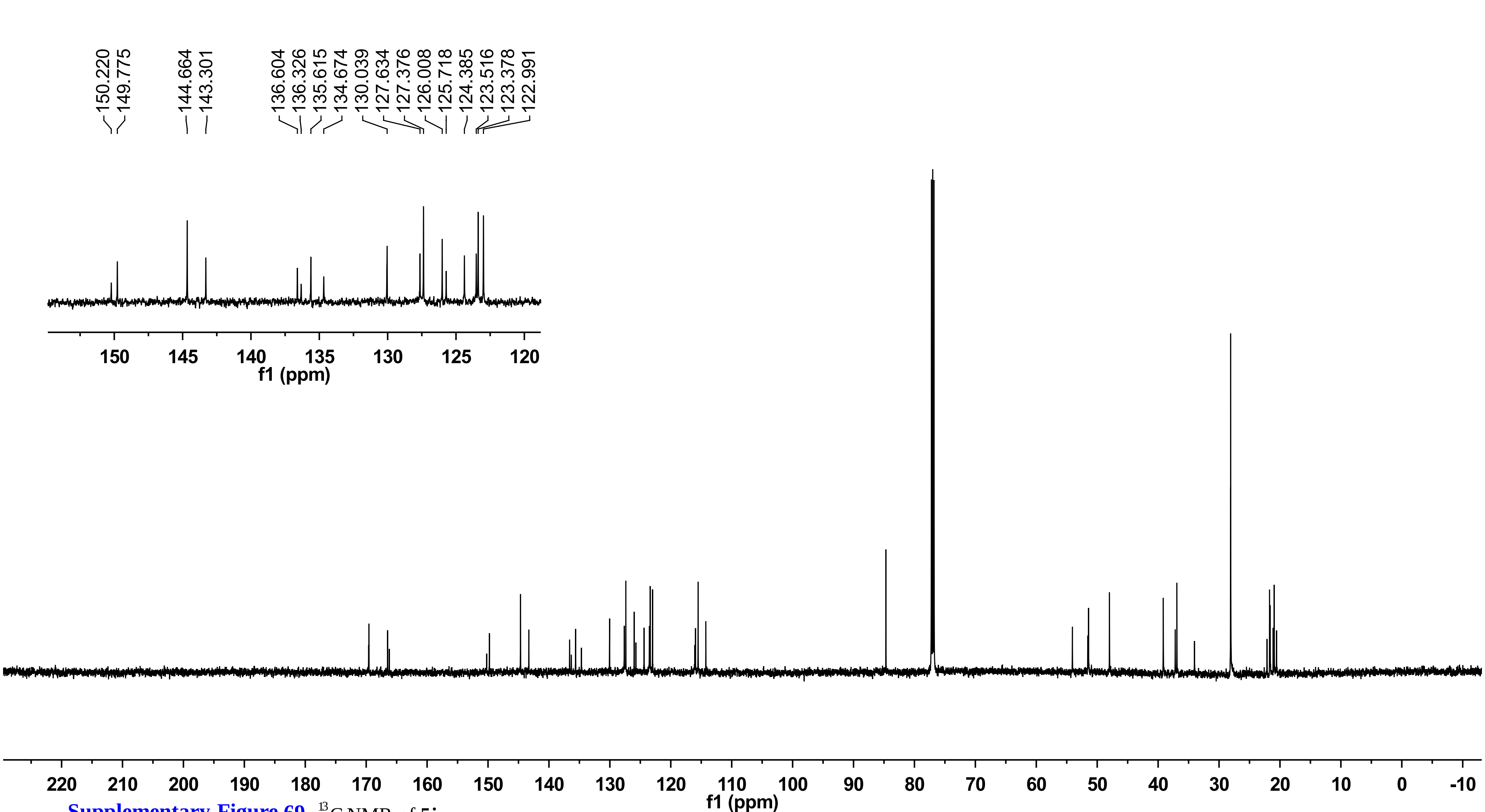

Supplementary Figure 69. ^{13}C NMR of **5j**

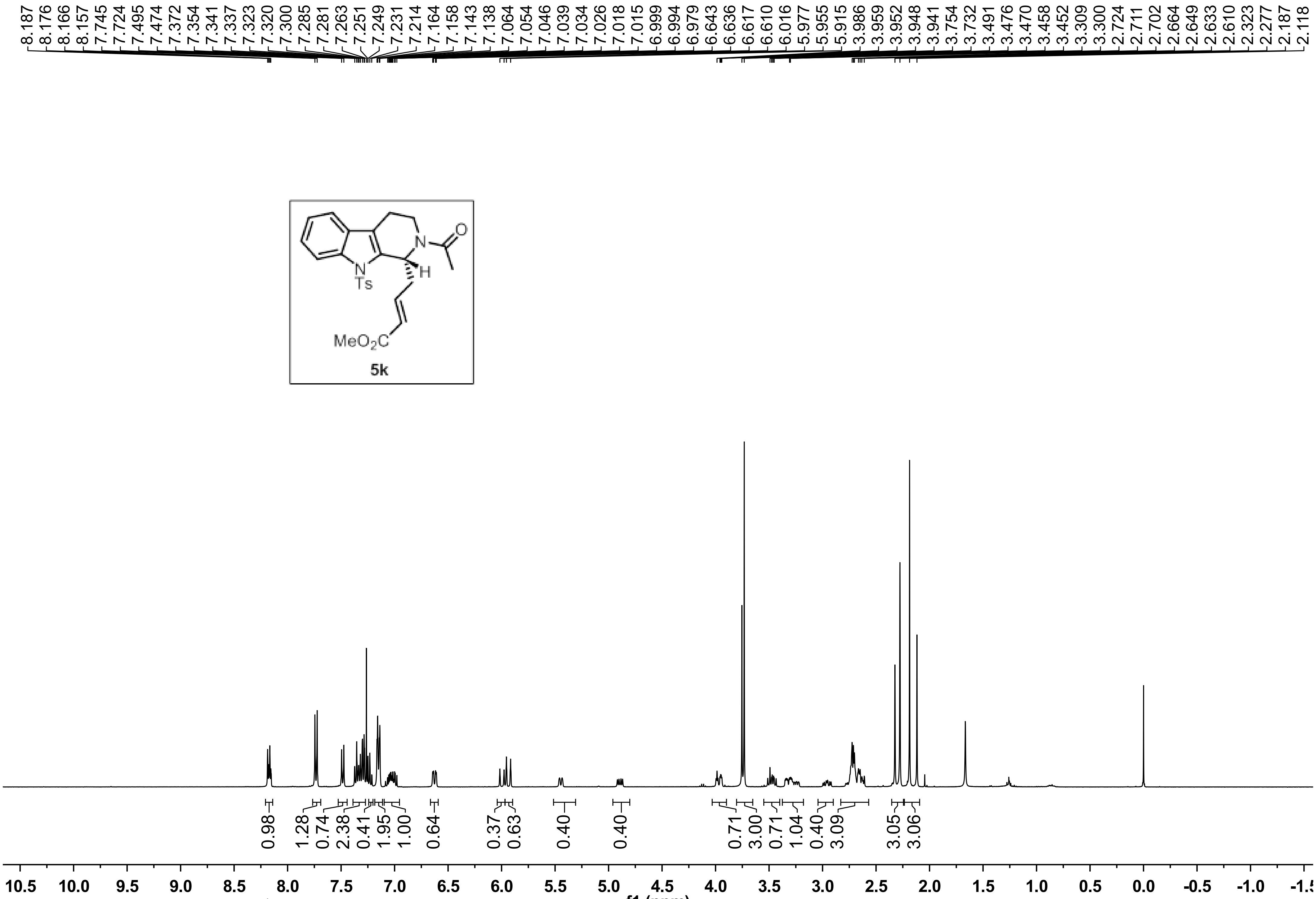

Supplementary Figure 70. ^{1}H NMR of **5k**

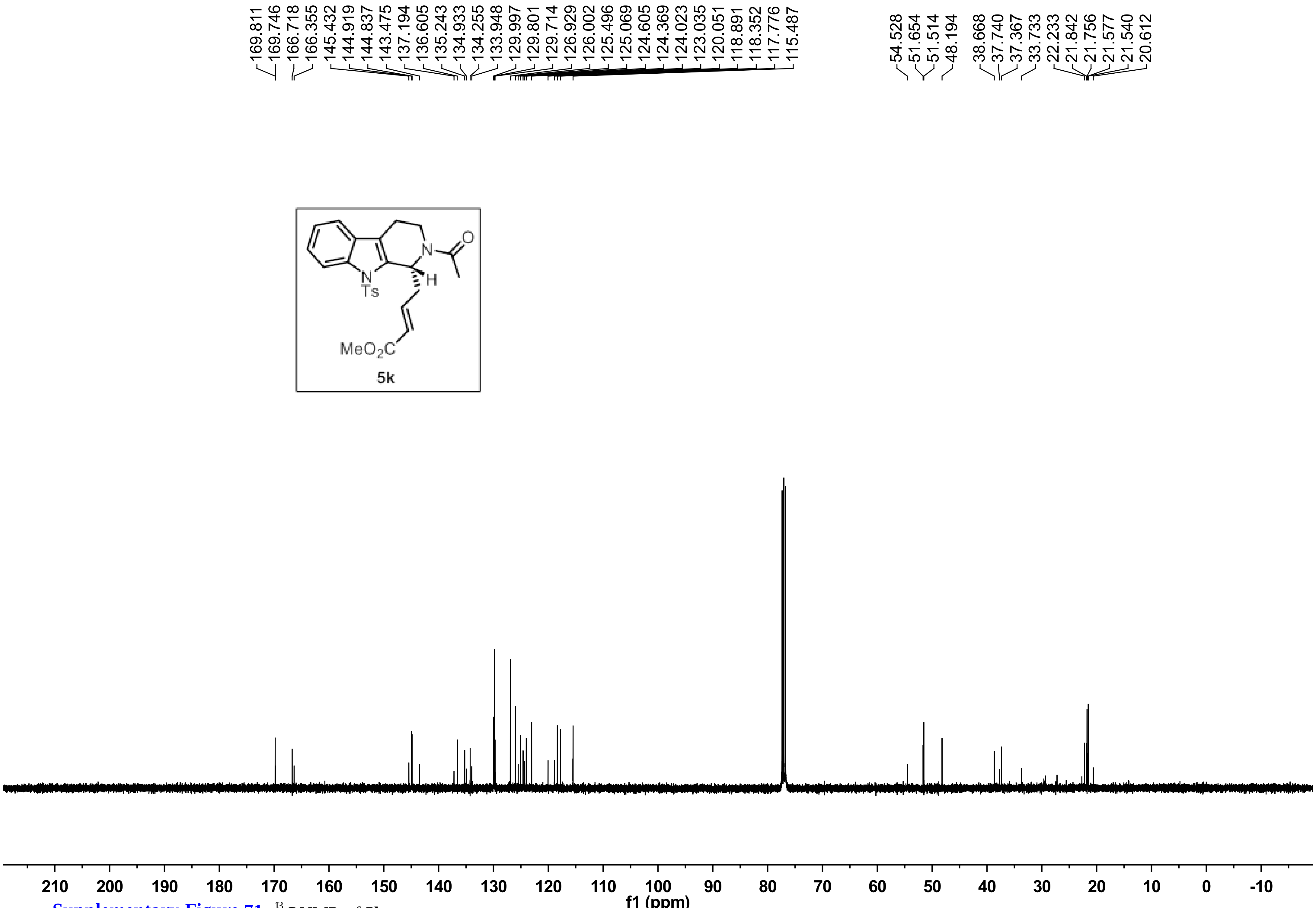

Supplementary Figure 71. ^{13}C NMR of **5k**

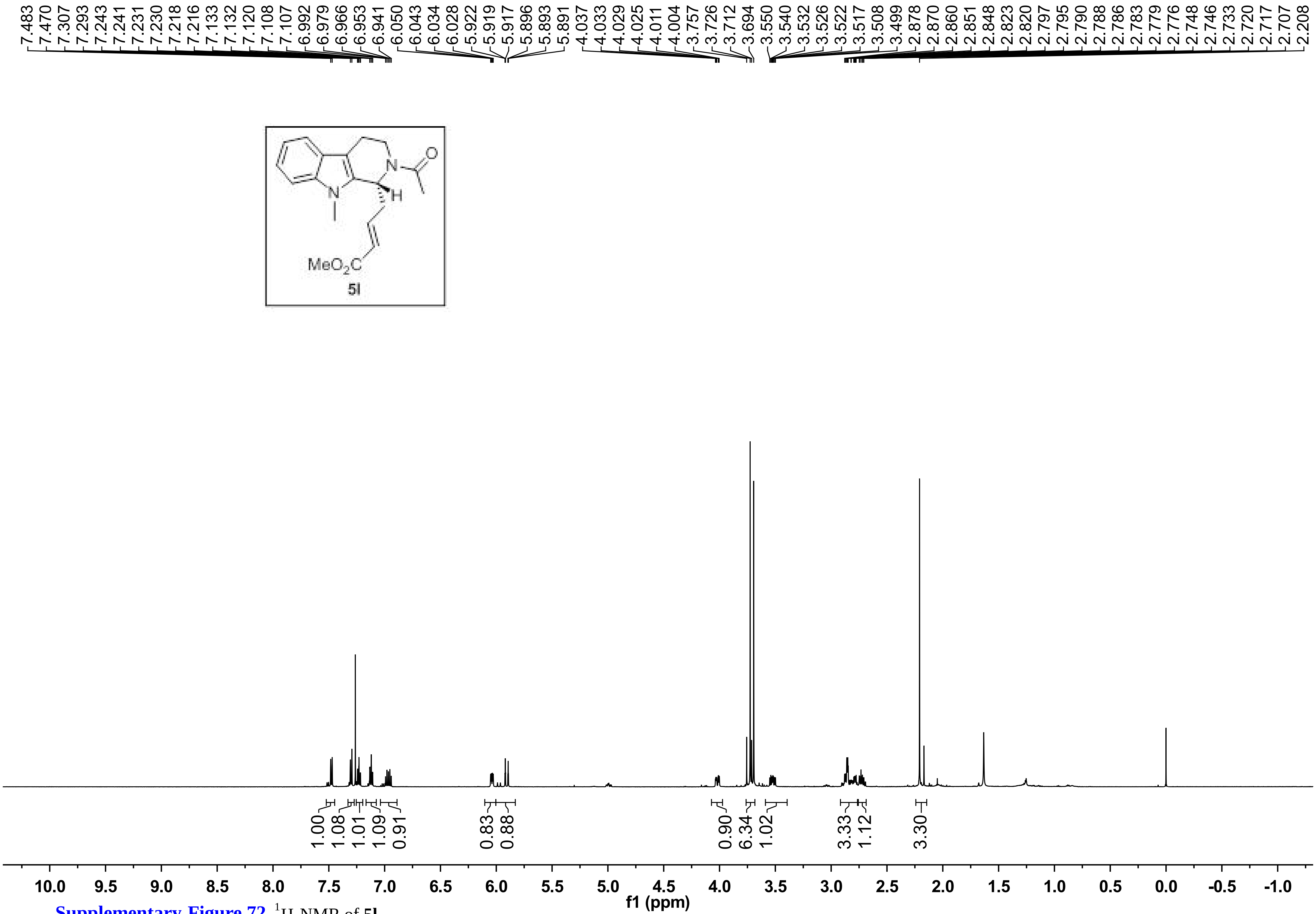

Supplementary Figure 72. ^{1}H NMR of **5l**

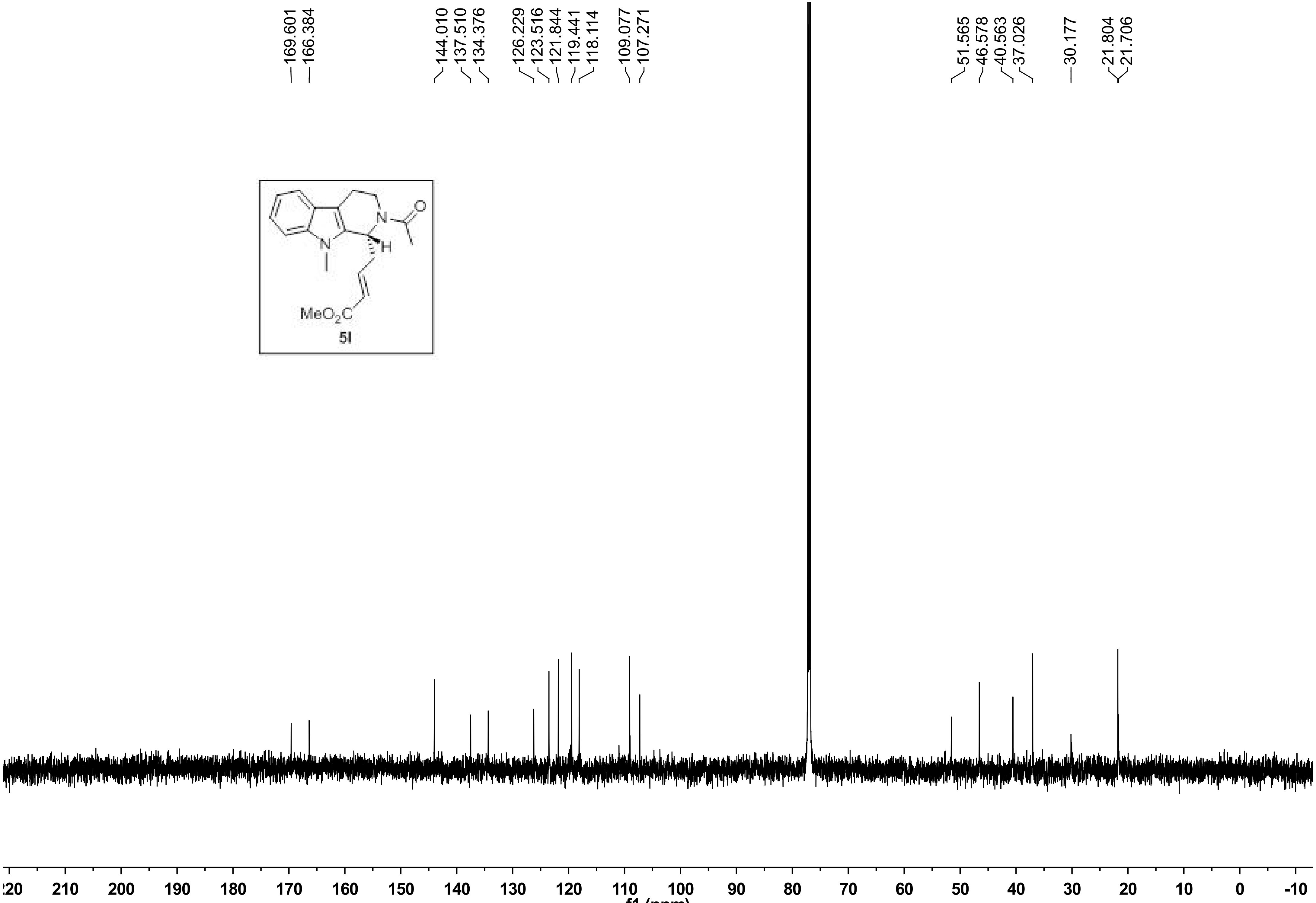

Supplementary Figure 73. ^{13}C NMR of **5l**

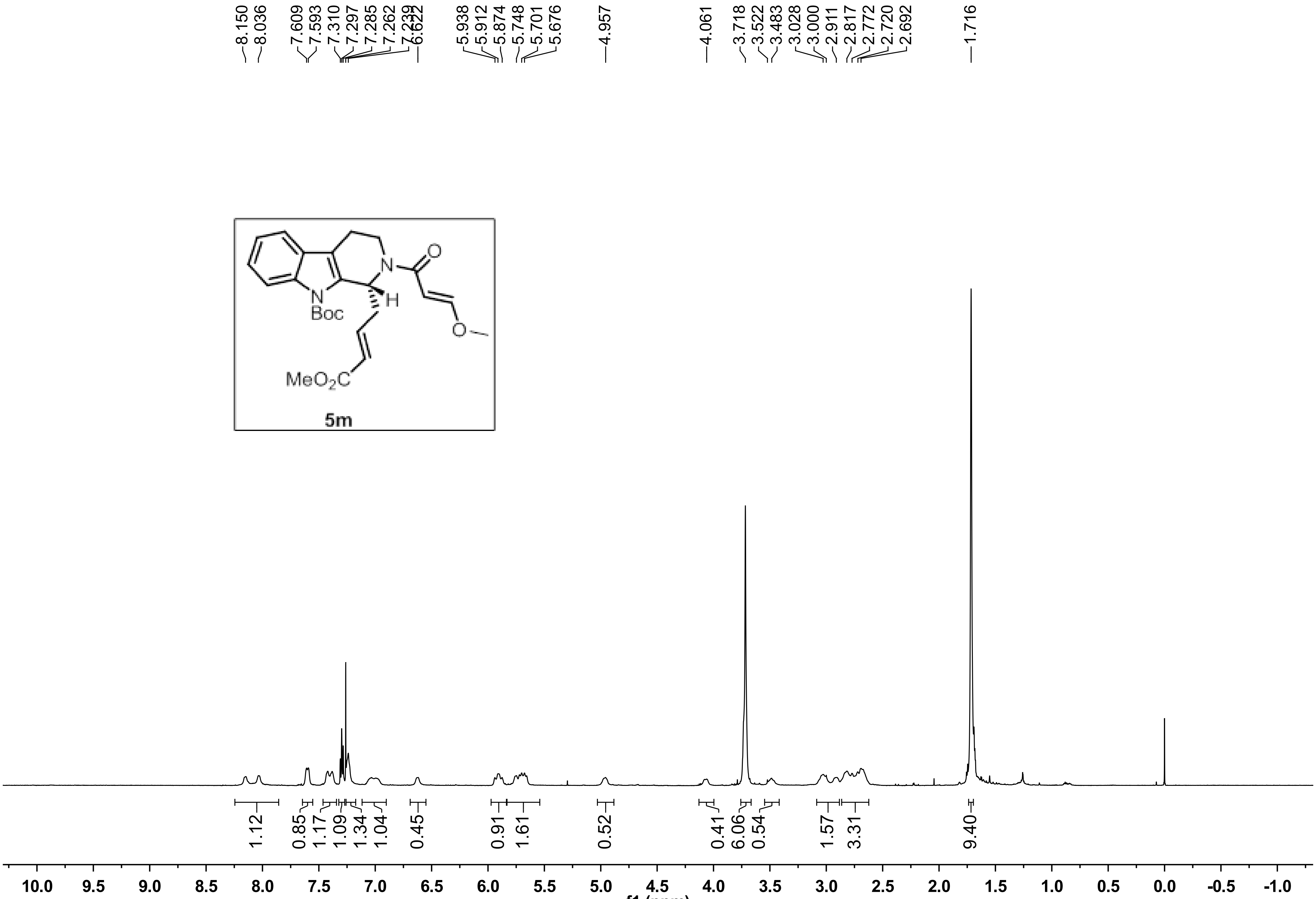

Supplementary Figure 74. ^{1}H NMR of **5m**

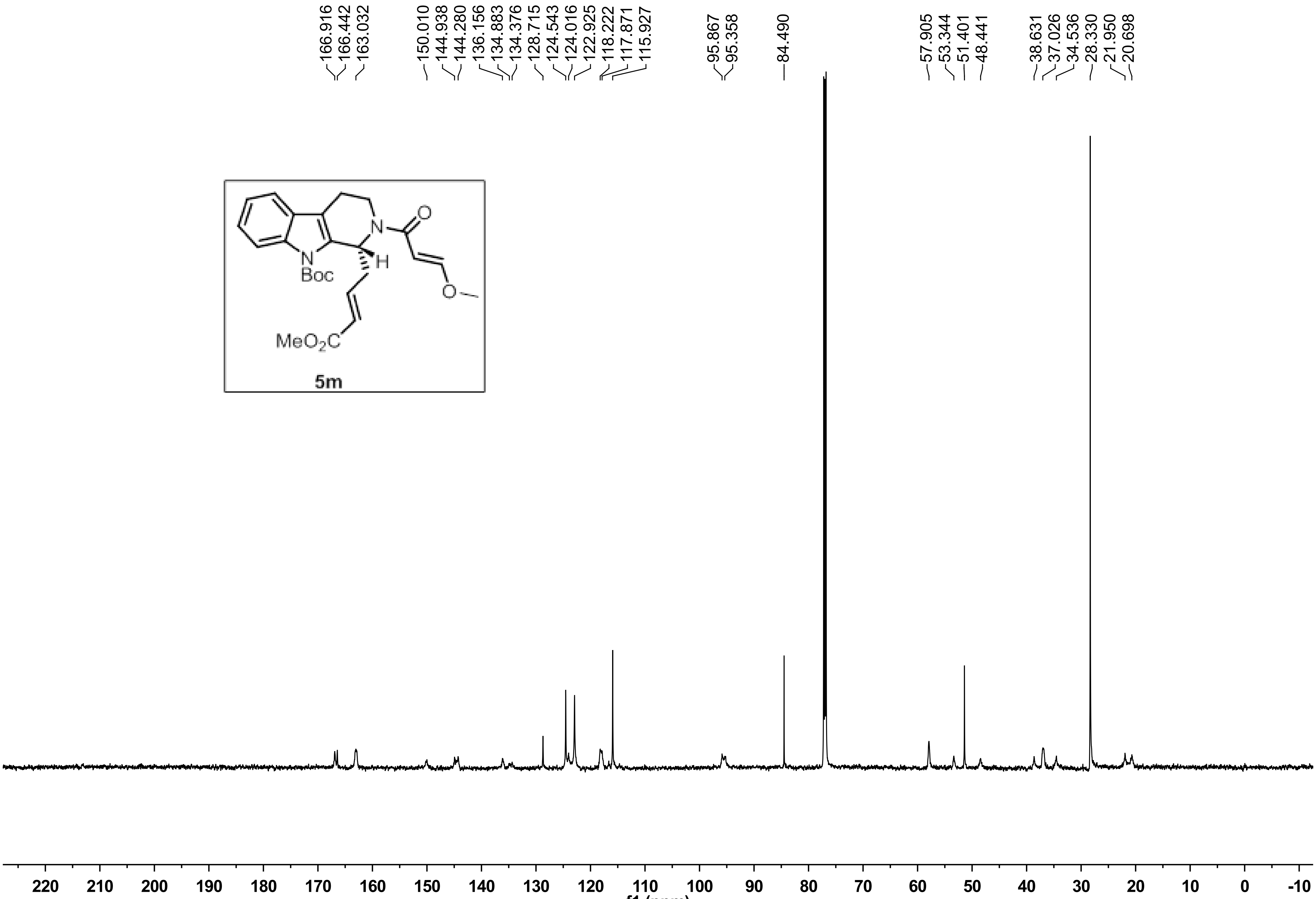

Supplementary Figure 75. ^{13}C NMR of **5m**

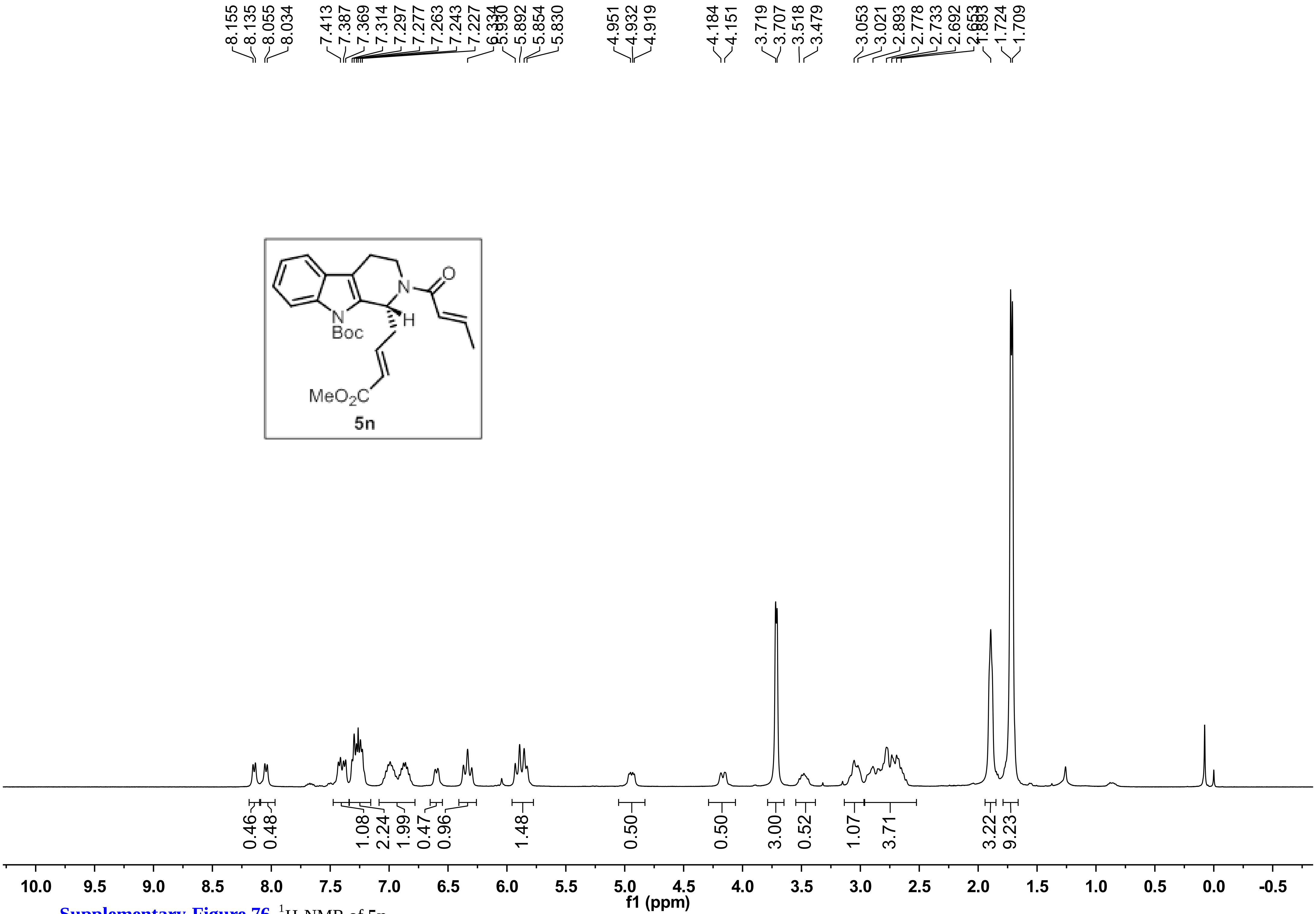

Supplementary Figure 76. ^{1}H NMR of **5n**

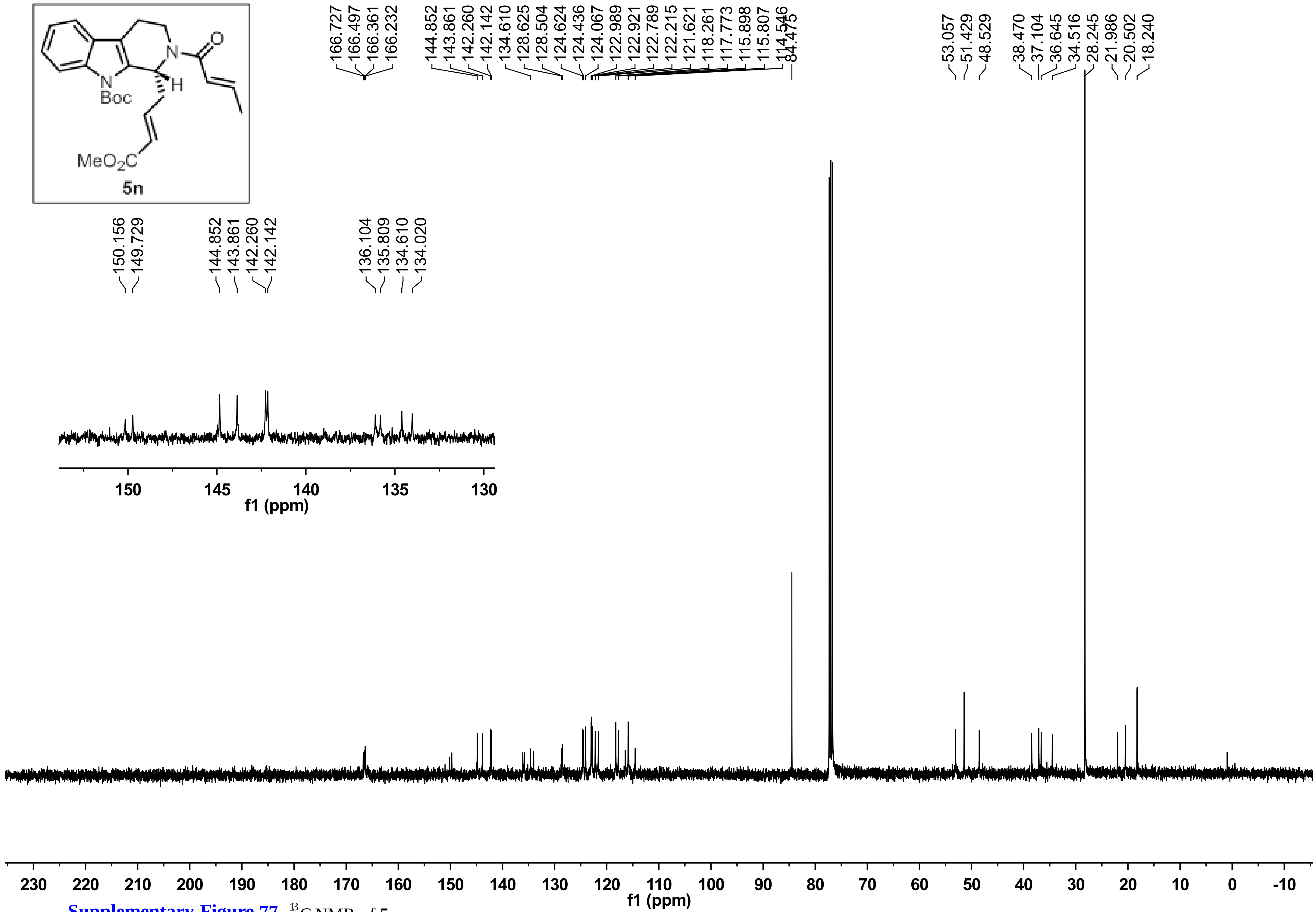

Supplementary Figure 77. ^{13}C NMR of **5n**

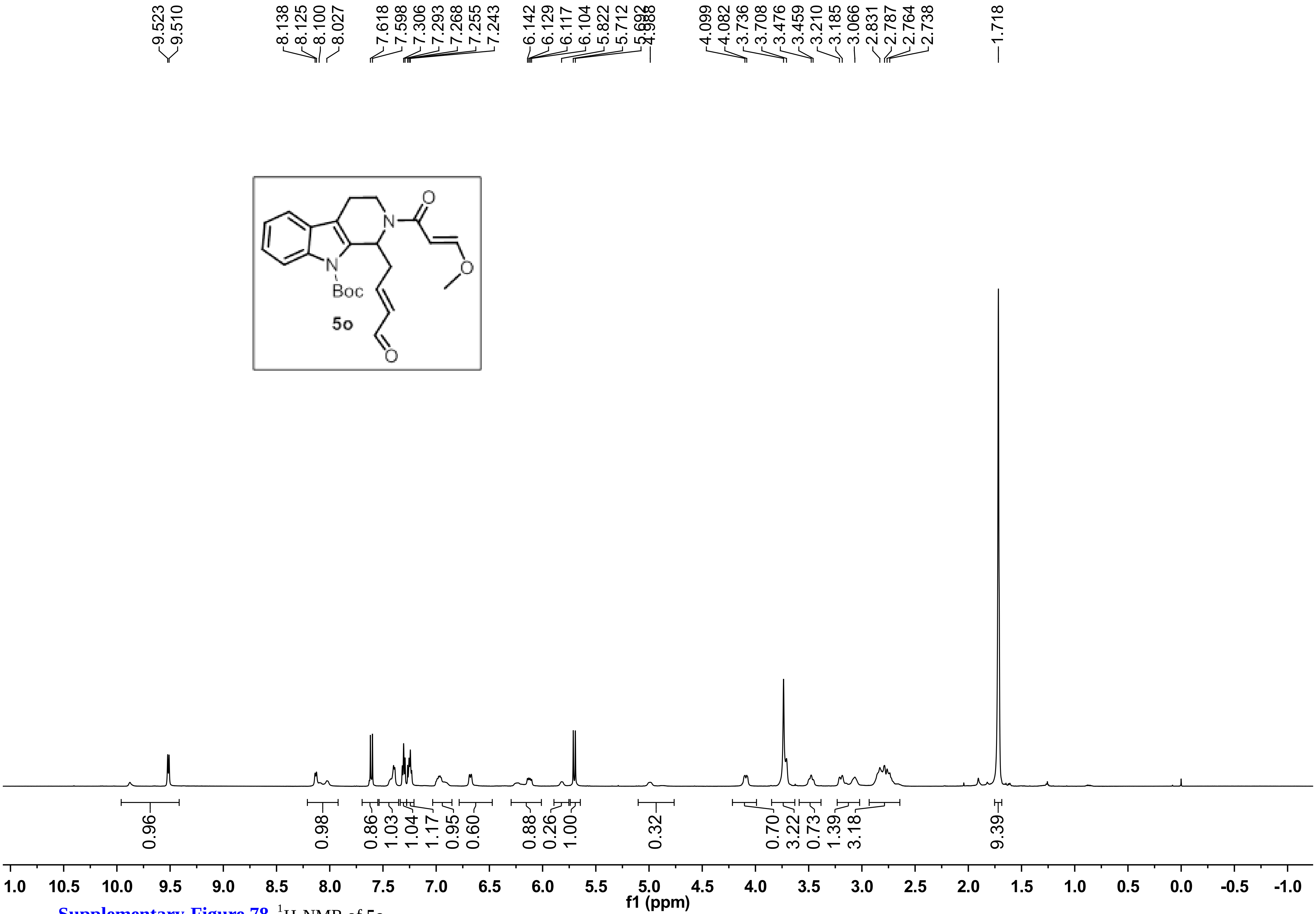

Supplementary Figure 78. ^{1}H NMR of **5o**

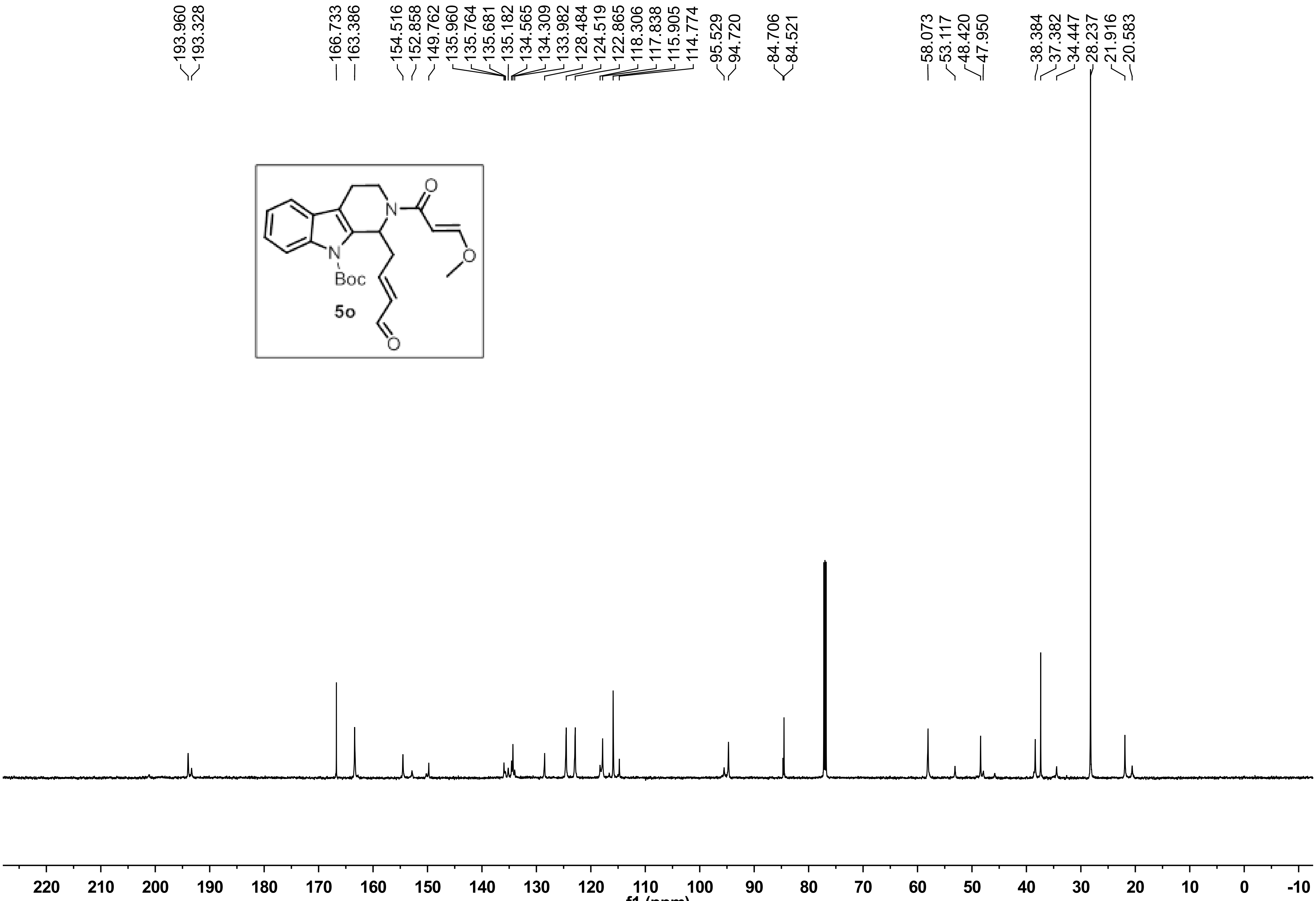

Supplementary Figure 79. ^{13}C NMR of **5o**

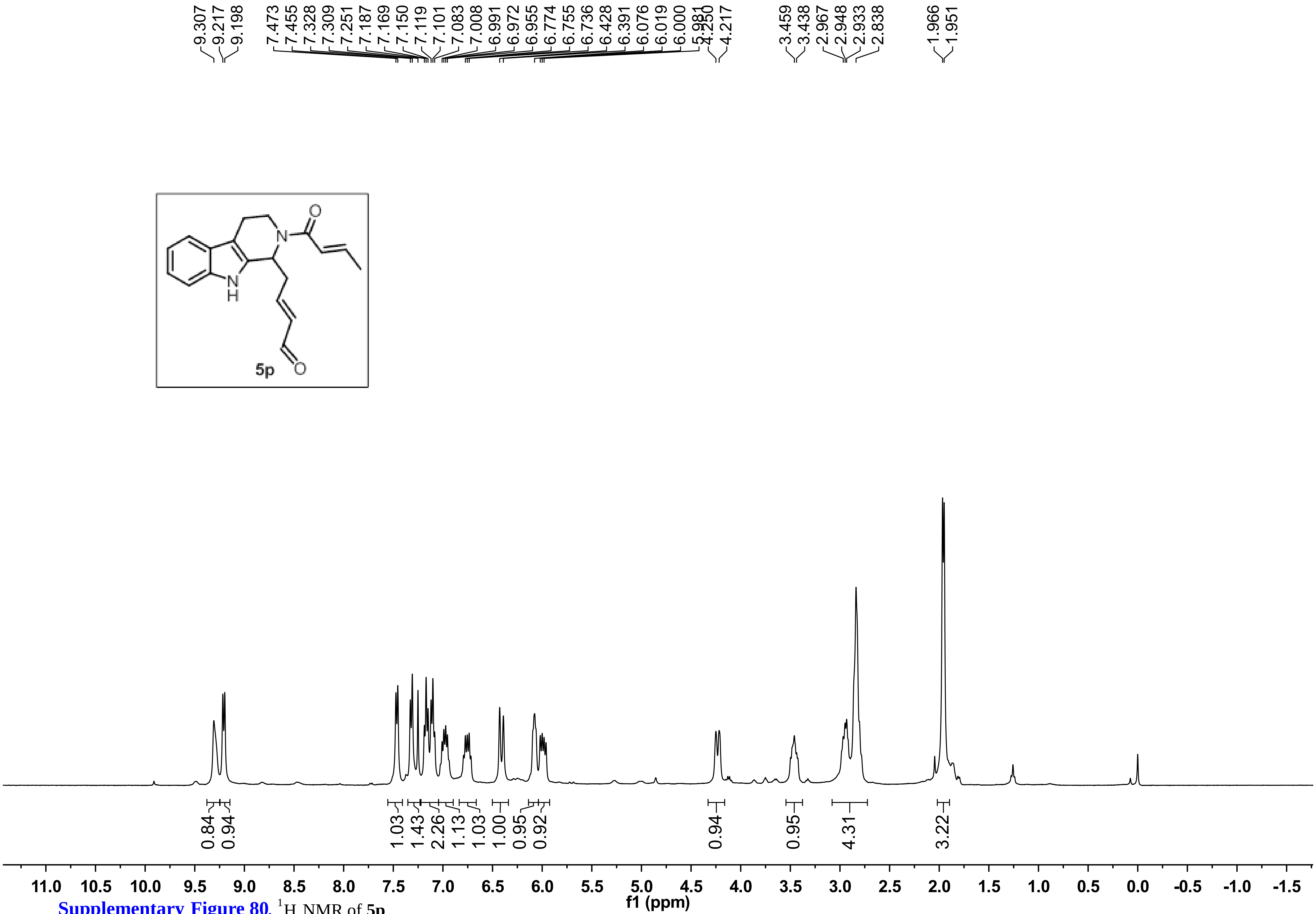

Supplementary Figure 80. ^{1}H NMR of **5p**

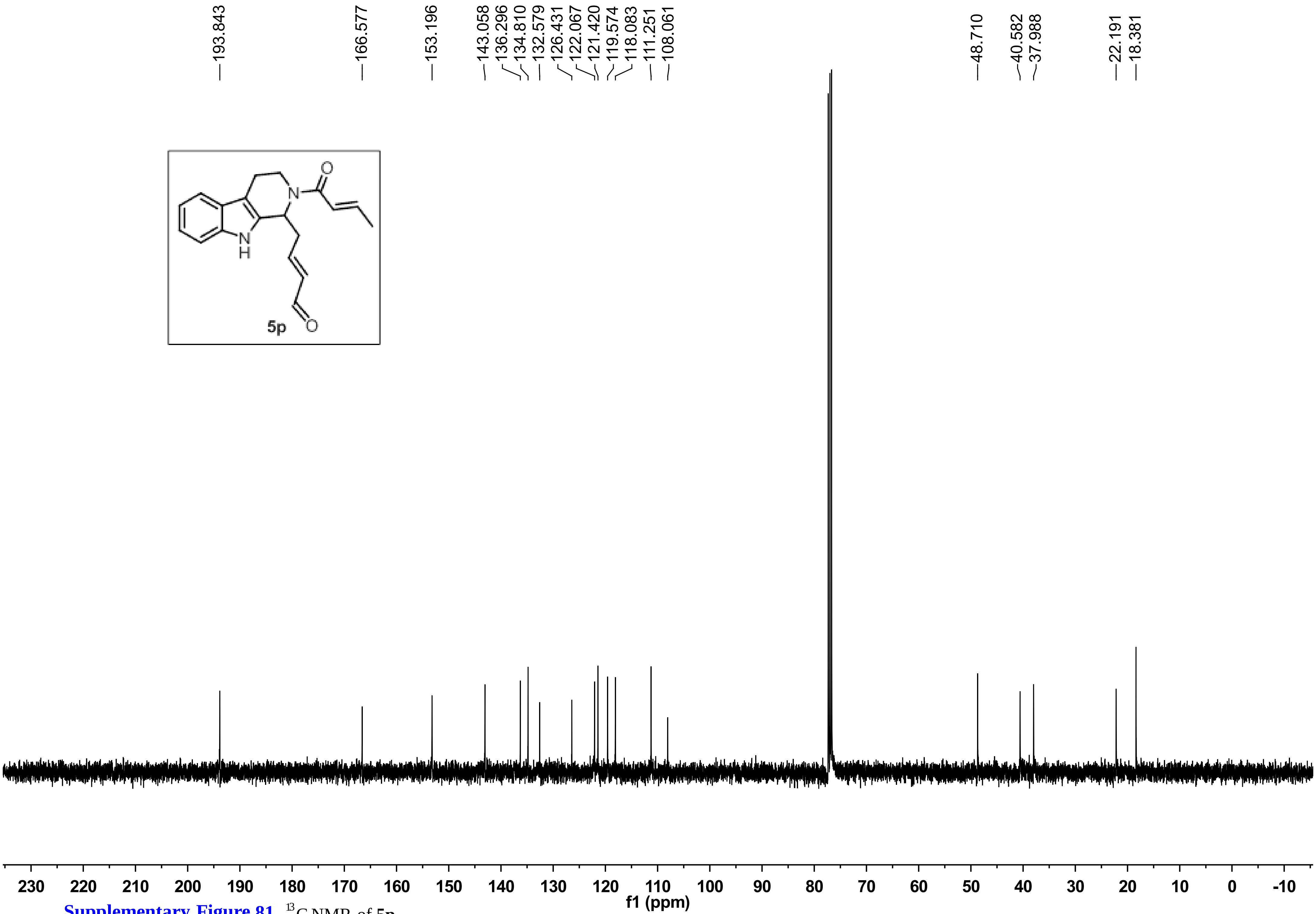

Supplementary Figure 81. ^{13}C NMR of **5p**

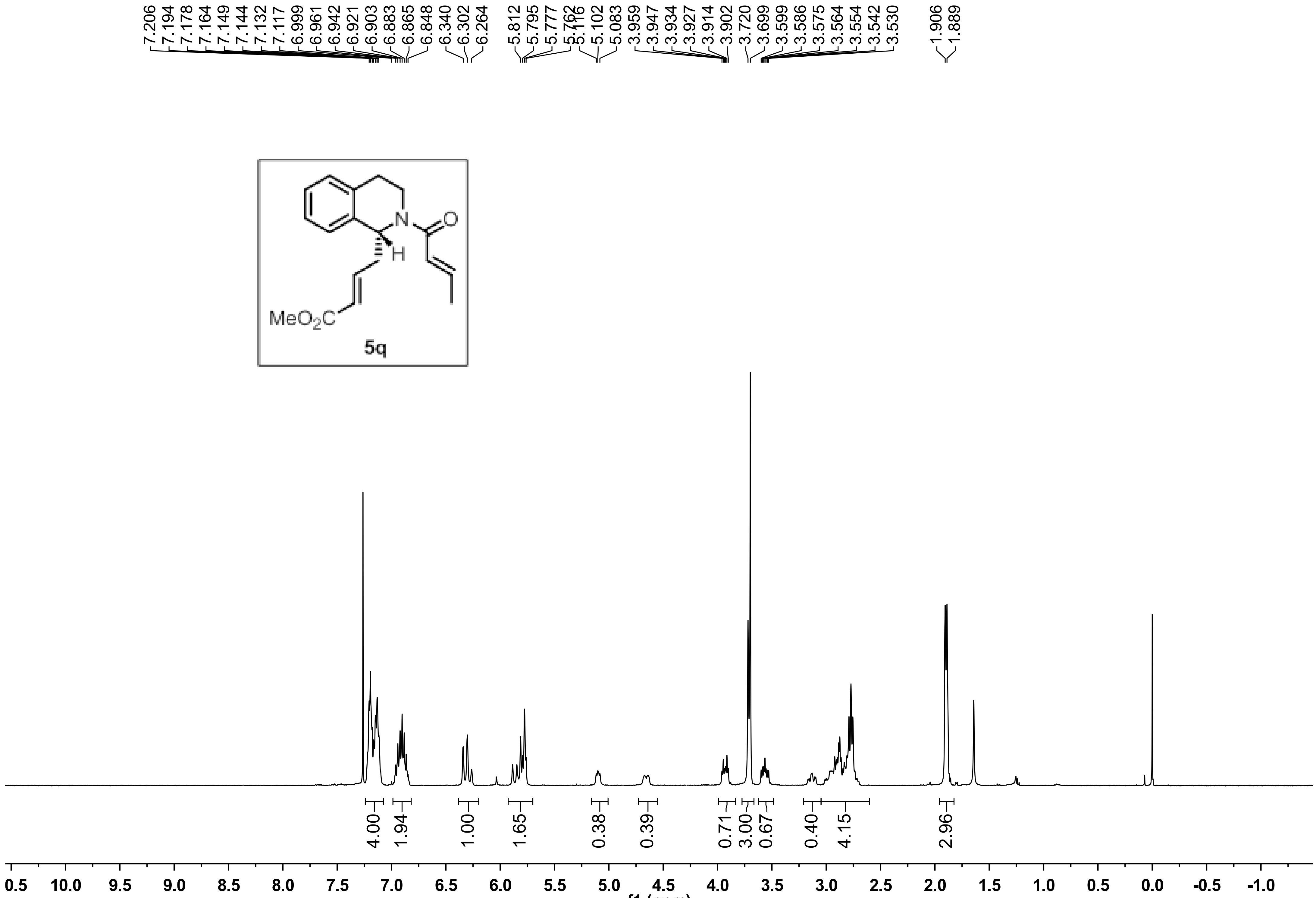

Supplementary Figure 82. ^{1}H NMR of **5q**

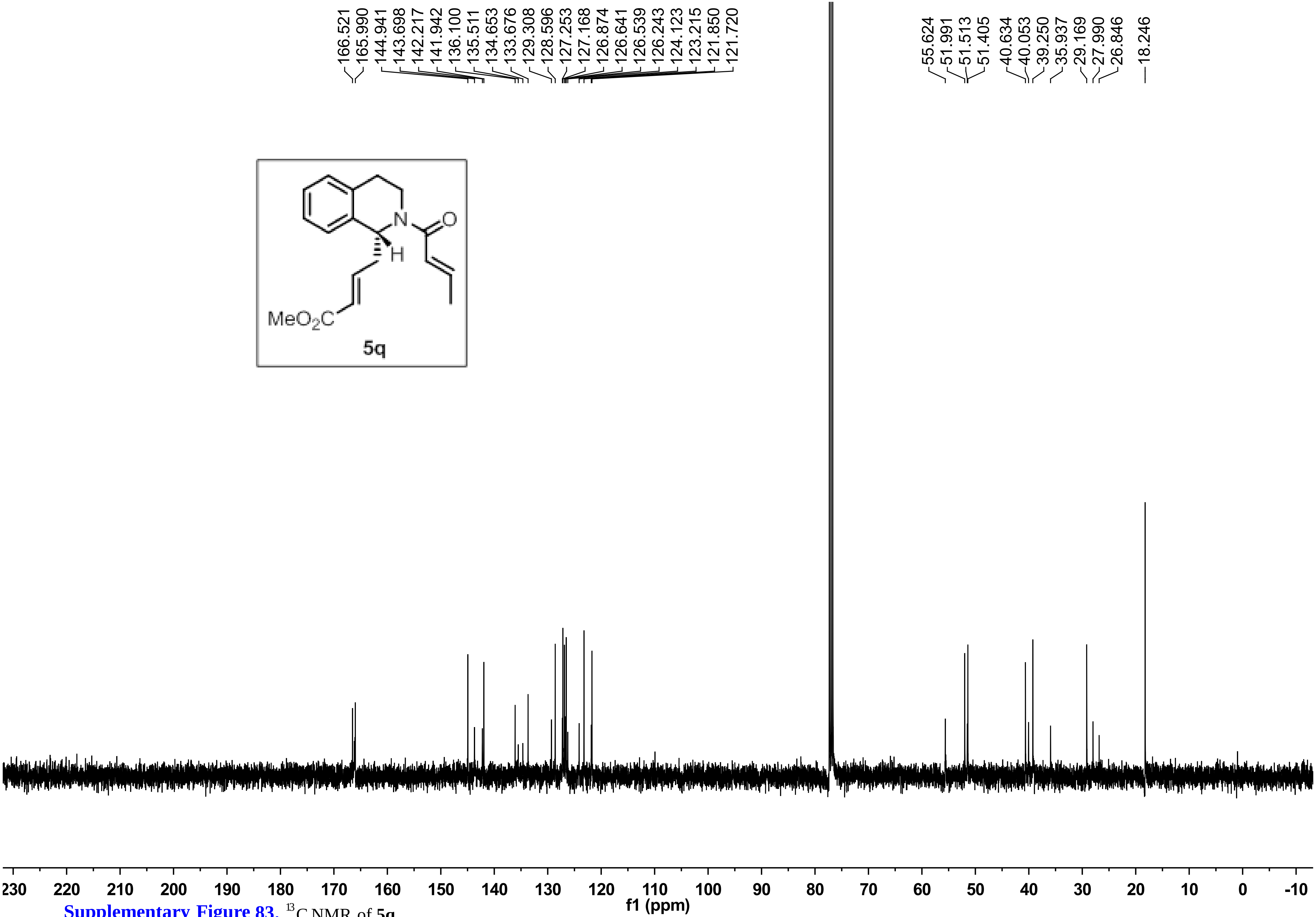

Supplementary Figure 83. ^{13}C NMR of **5q**

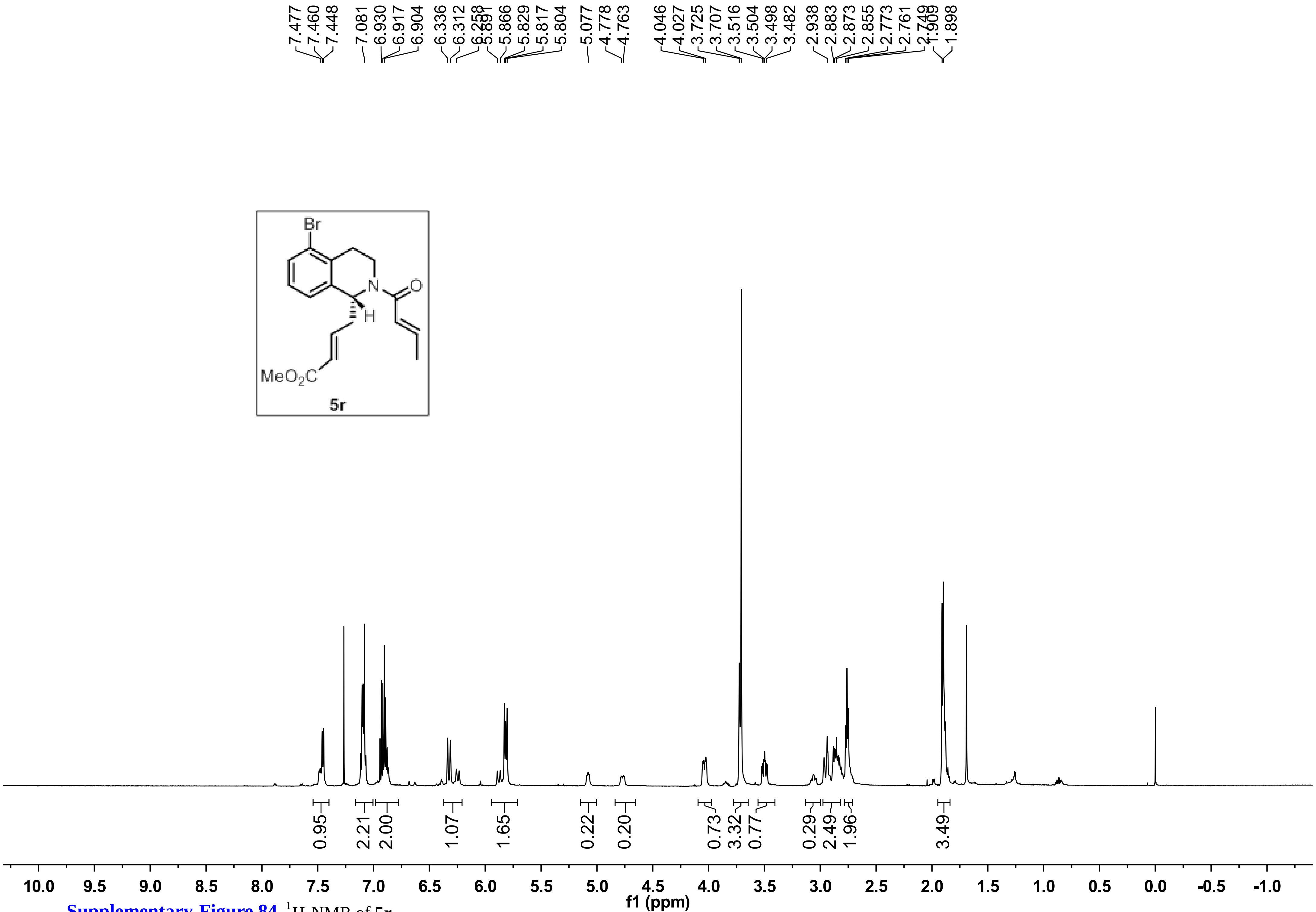

Supplementary Figure 84. ^{1}H NMR of **5r**

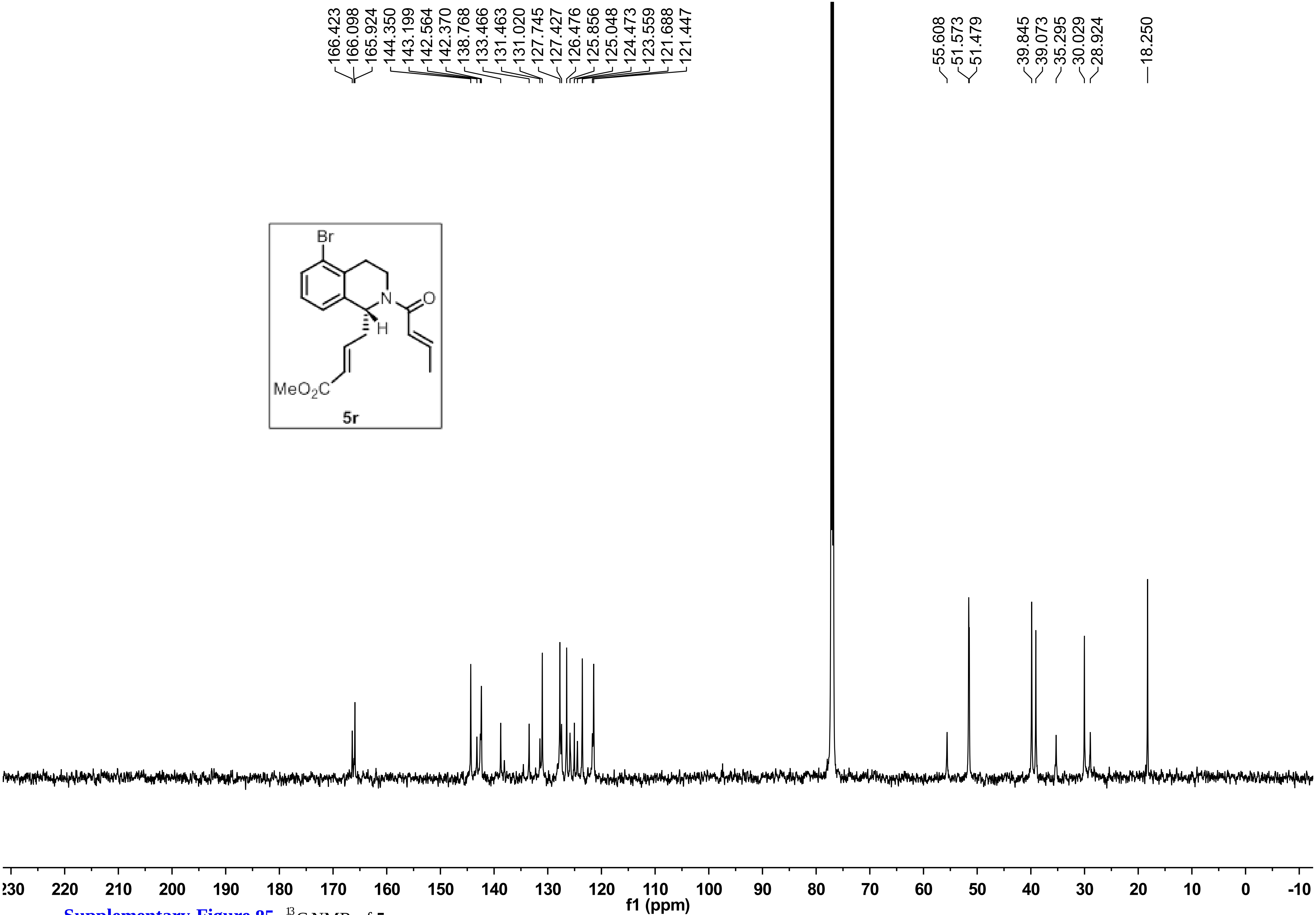

Supplementary Figure 85. ^{13}C NMR of **5r**

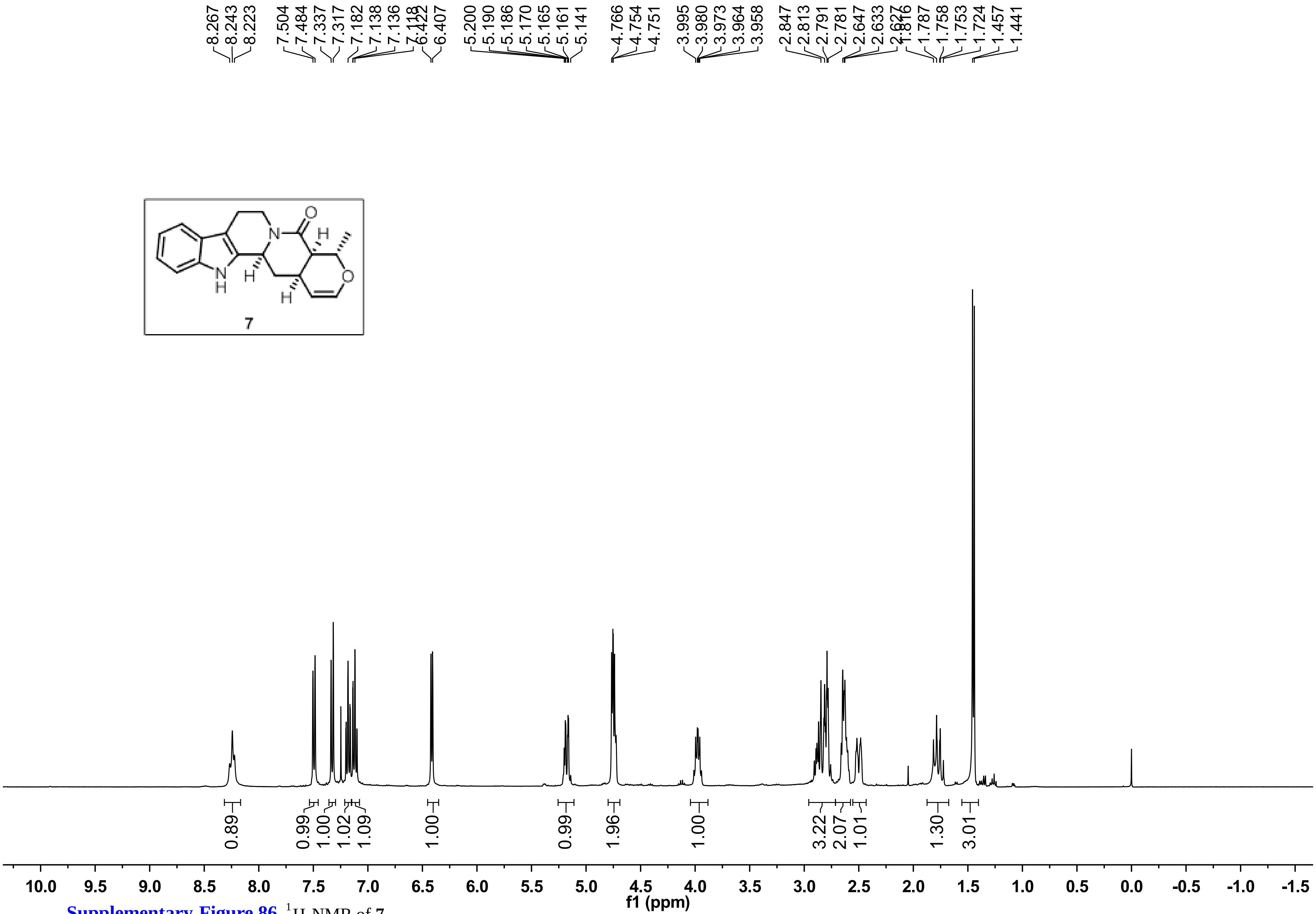

Supplementary Figure 86. ^{1}H NMR of **7**

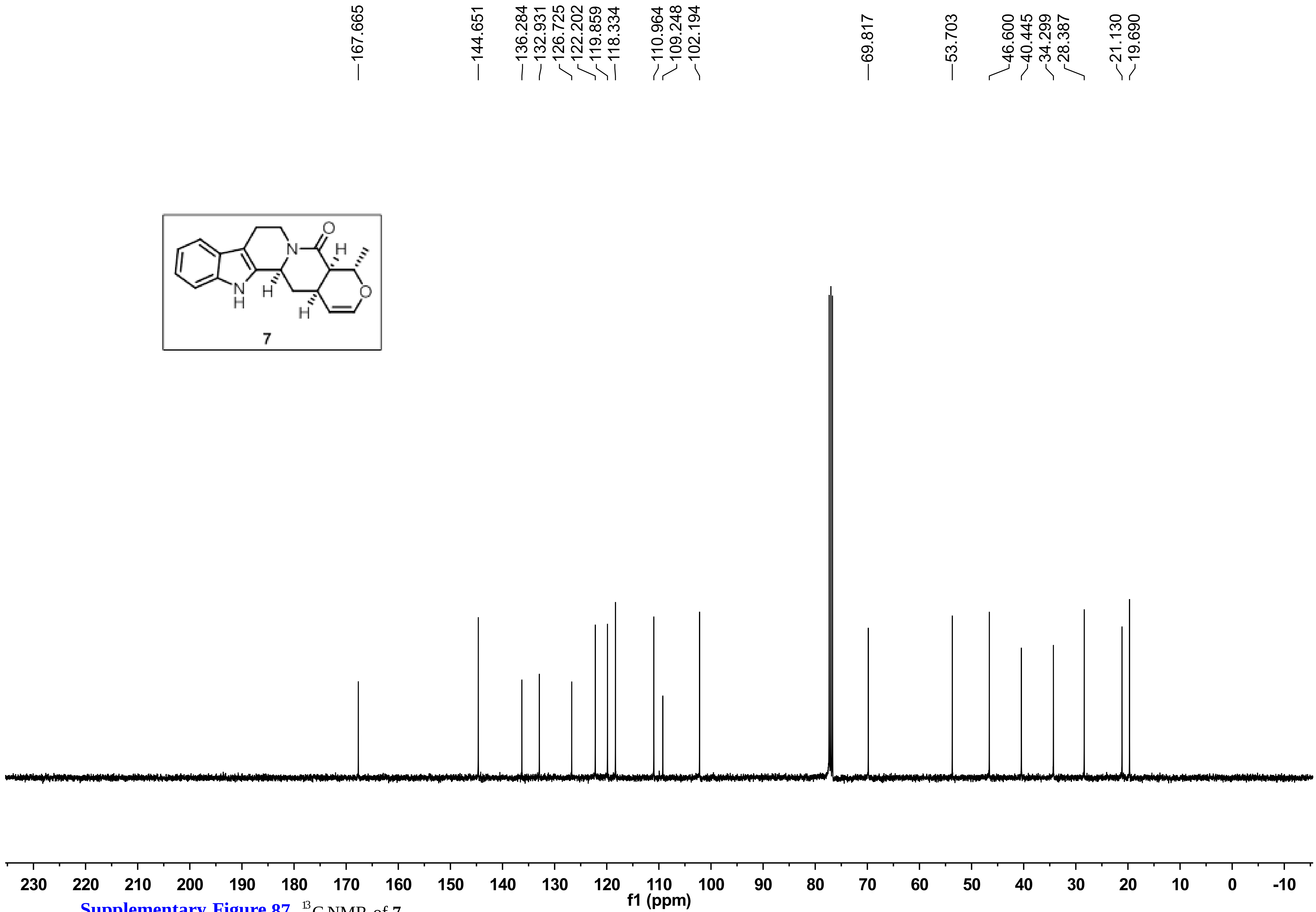

Supplementary Figure 87. ^{13}C NMR of **7**

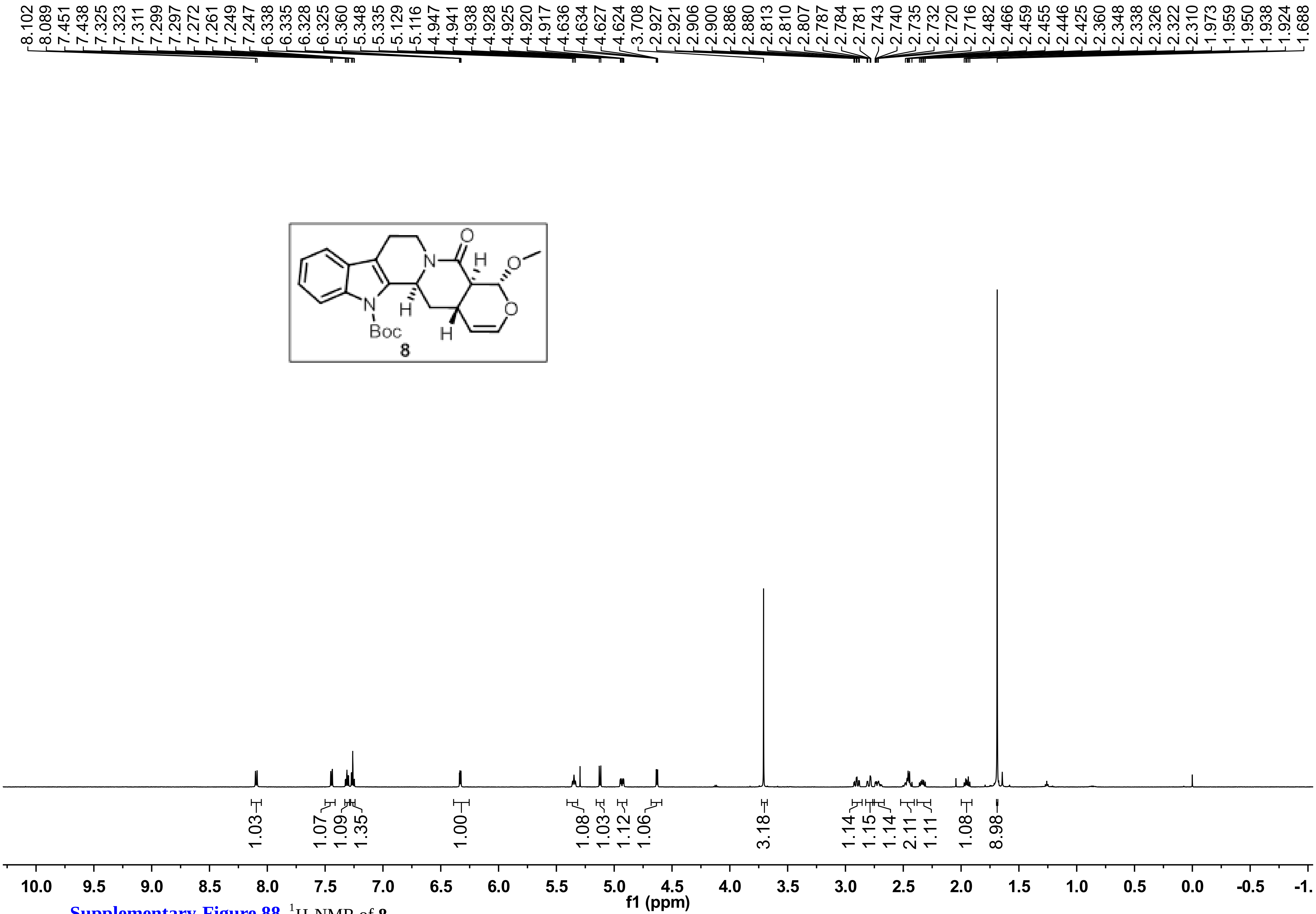

Supplementary Figure 88. ^{1}H NMR of **8**

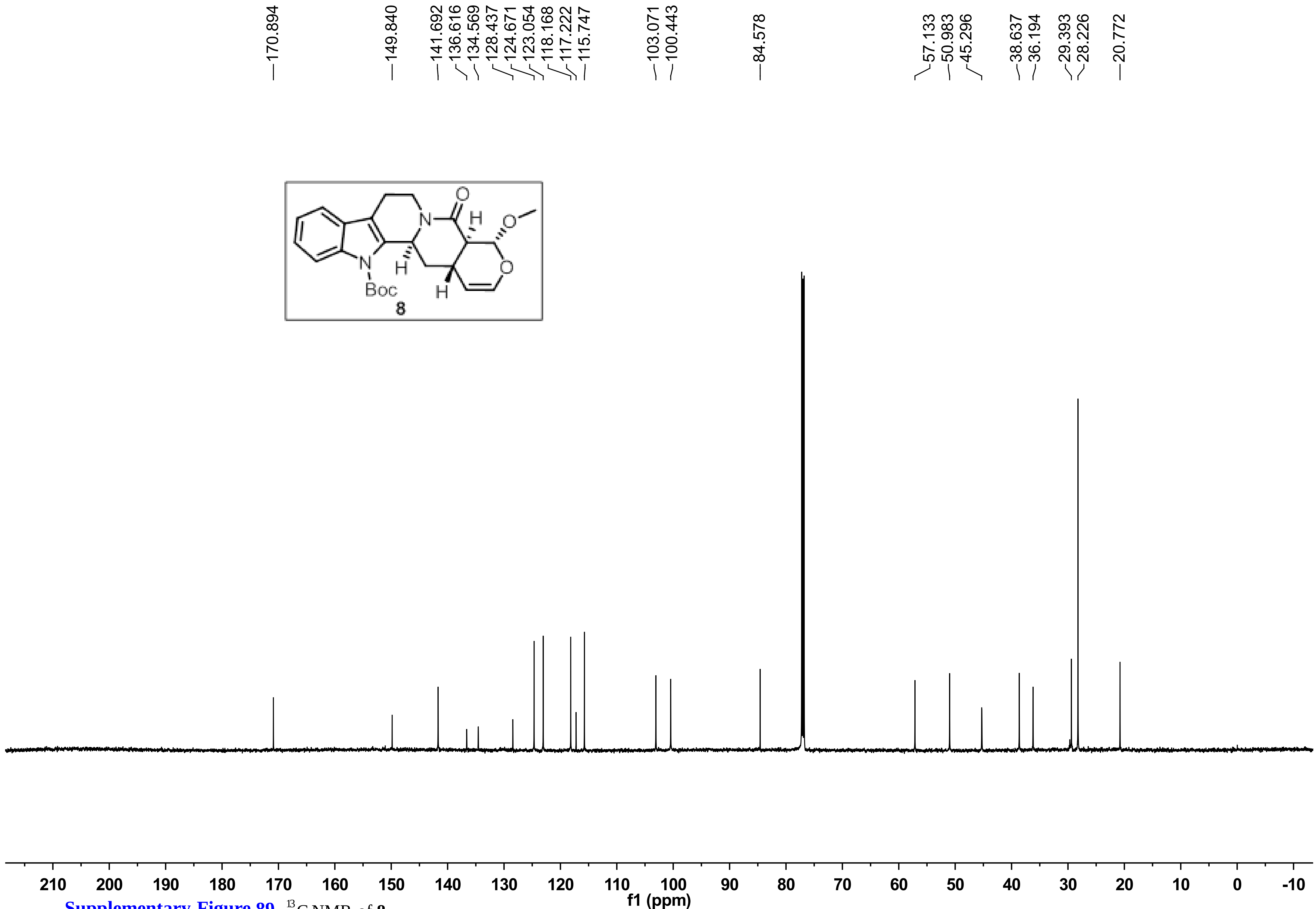

Supplementary Figure 89. ^{13}C NMR of **8**

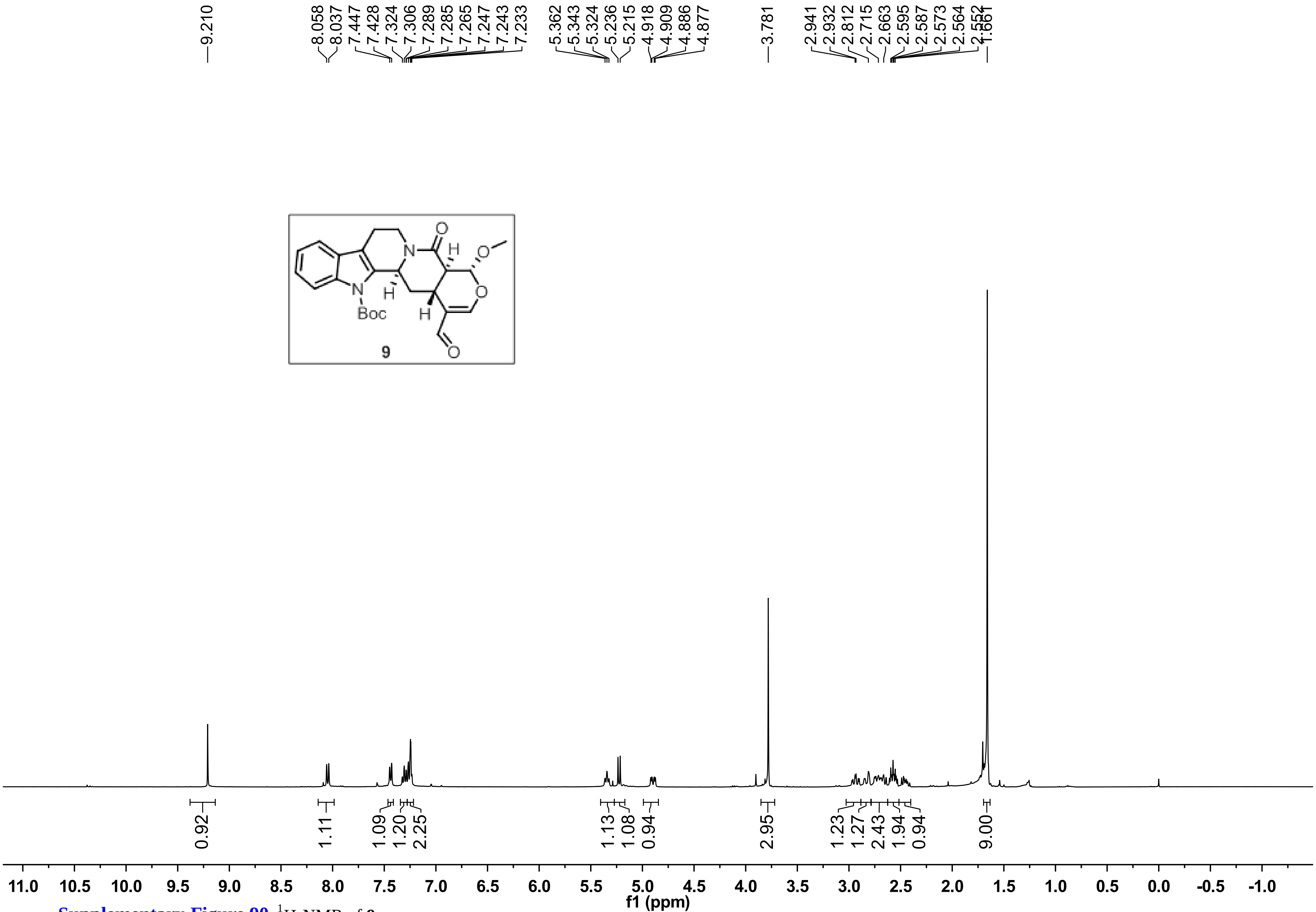

Supplementary Figure 90. ^{1}H NMR of **9**

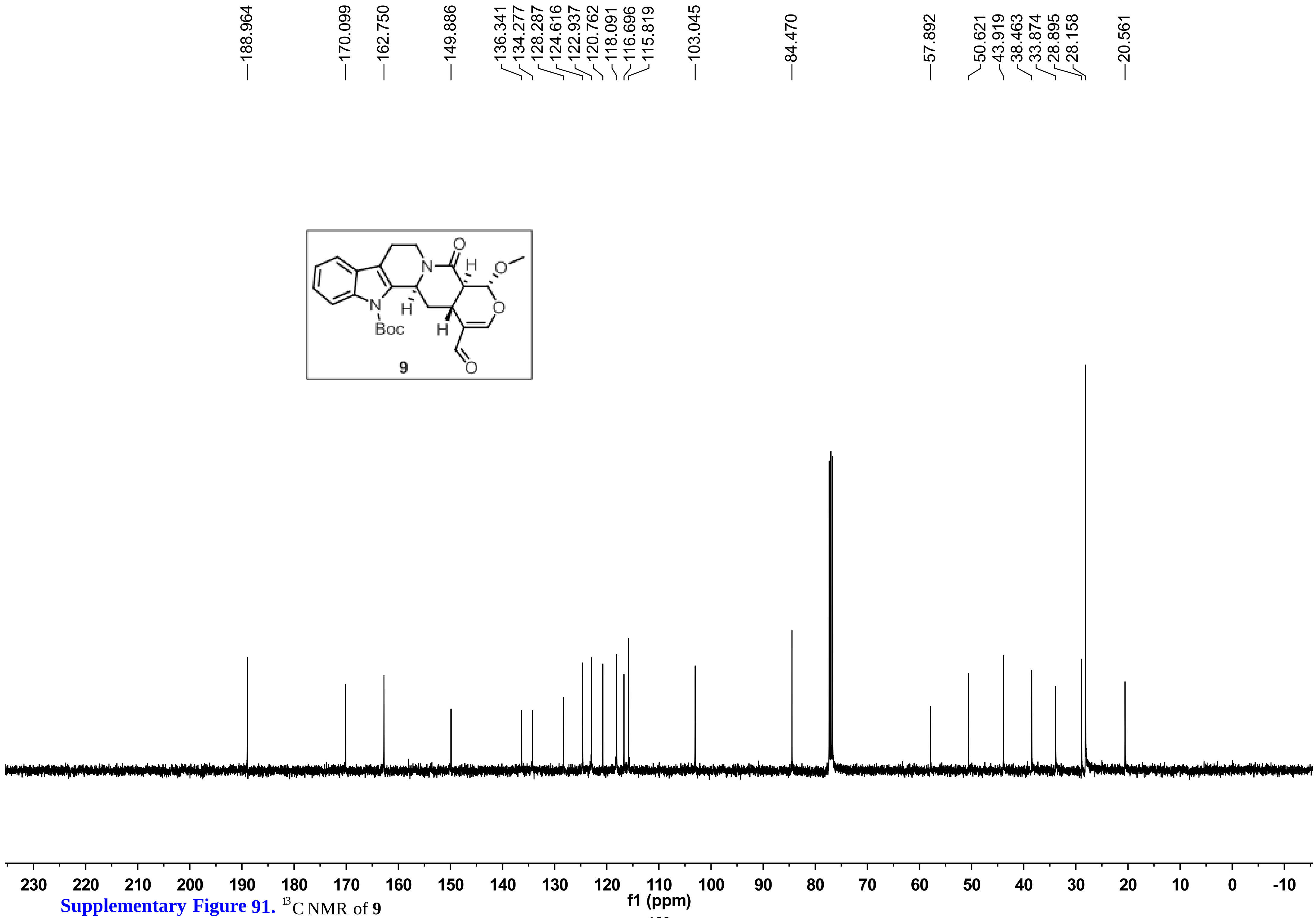

Supplementary Figure 91. ^{13}C NMR of **9**

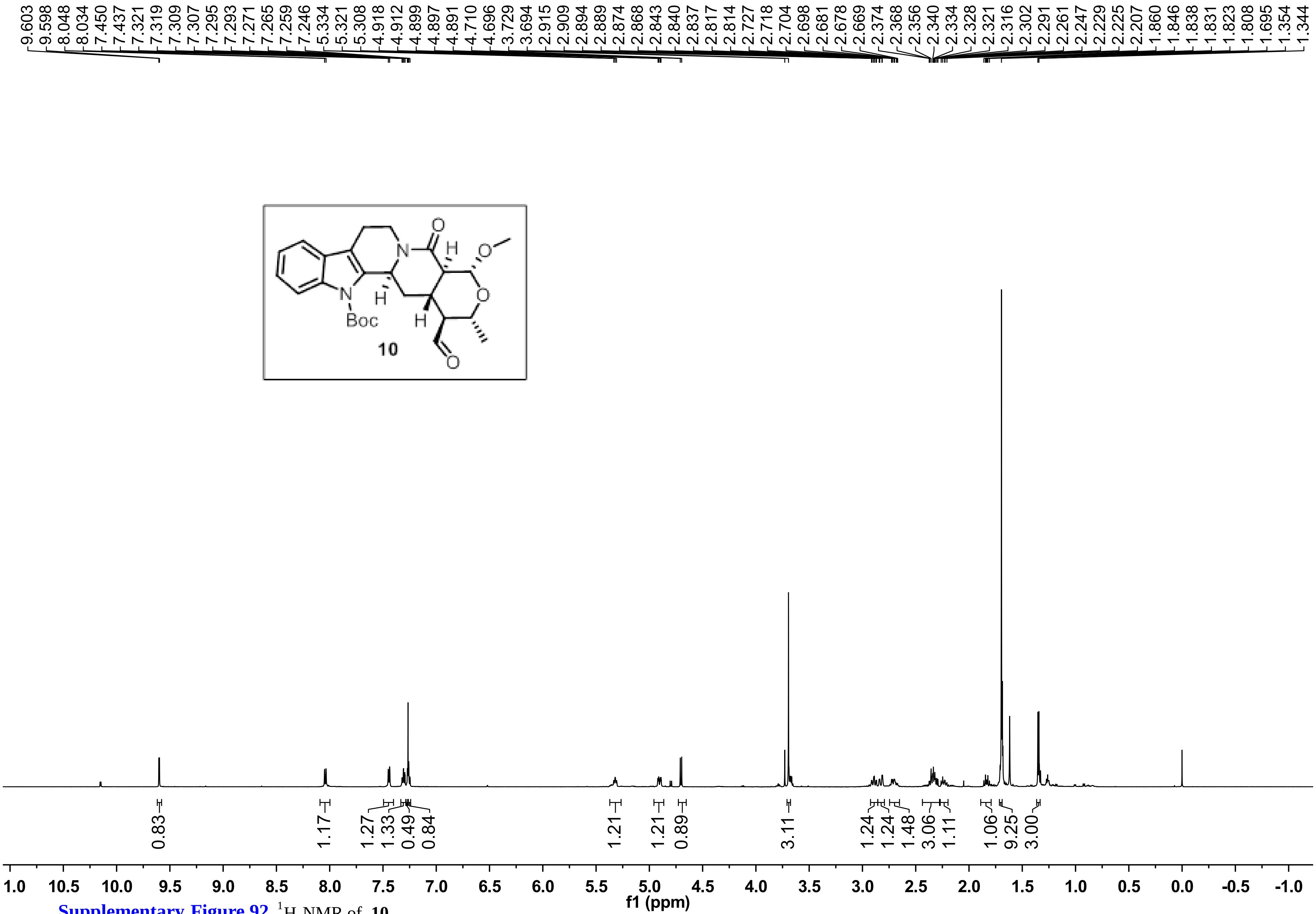

Supplementary Figure 92. ^{1}H NMR of **10**

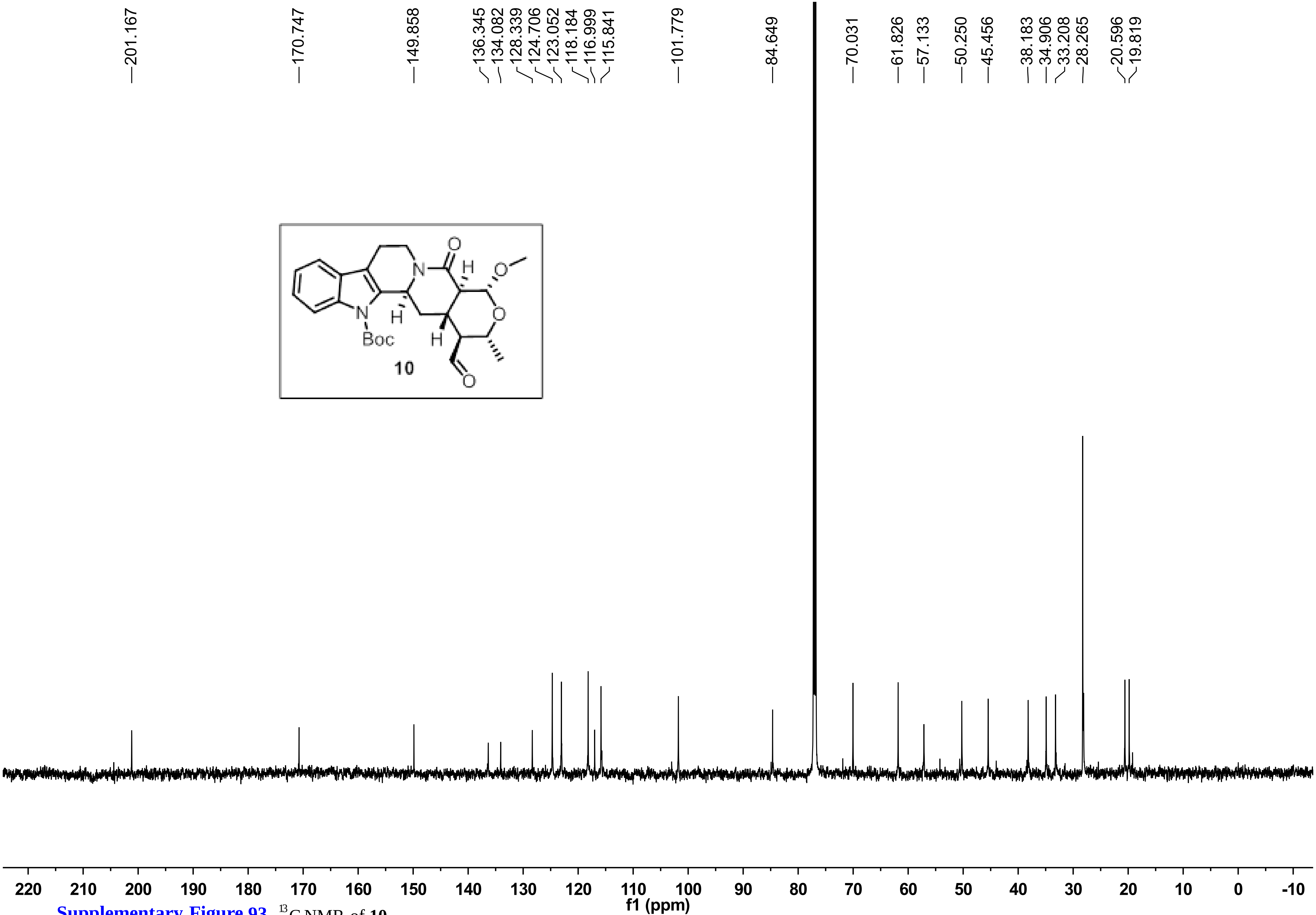

Supplementary Figure 93. ^{13}C NMR of **10**

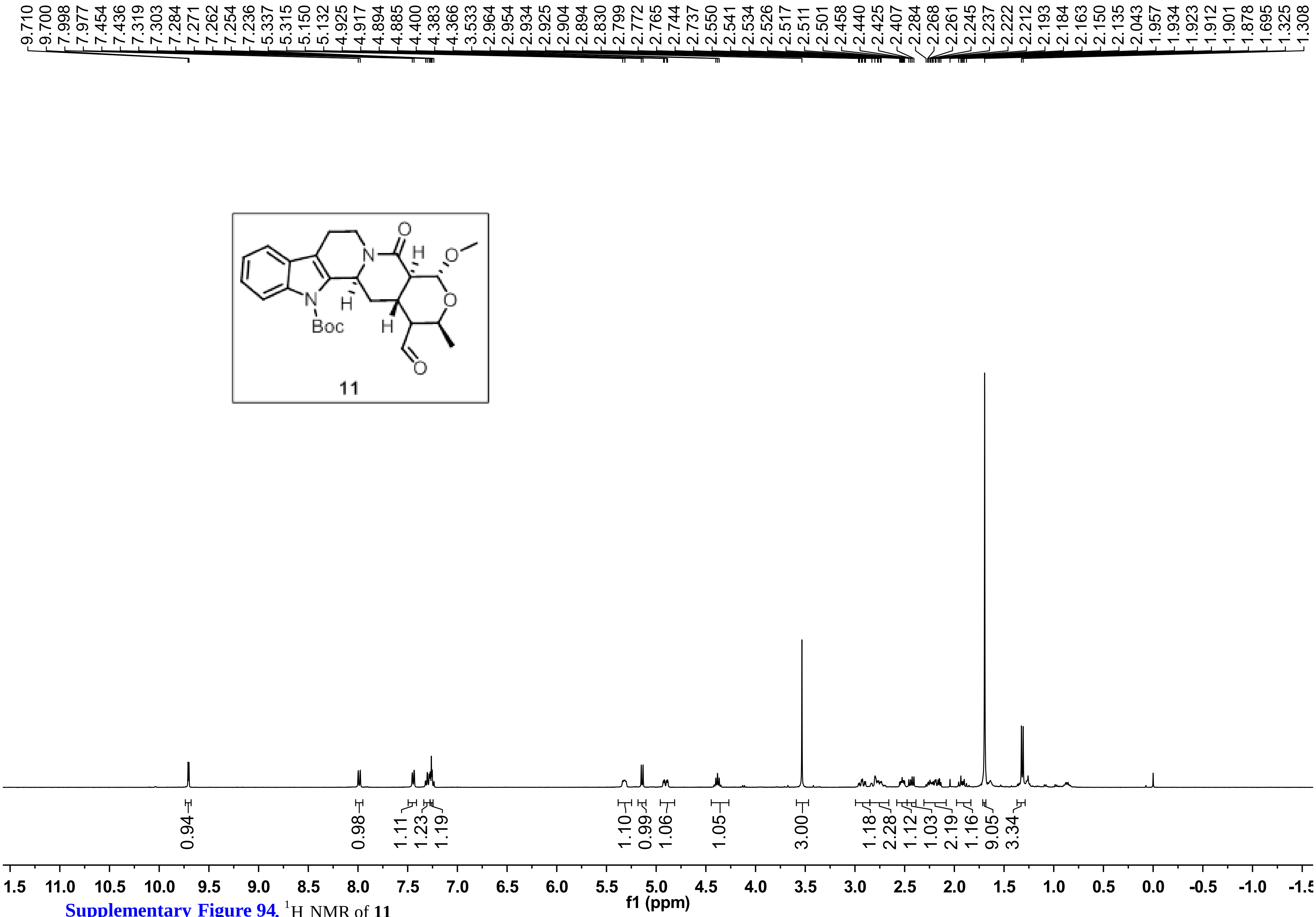

Supplementary Figure 94. ^{1}H NMR of **11**

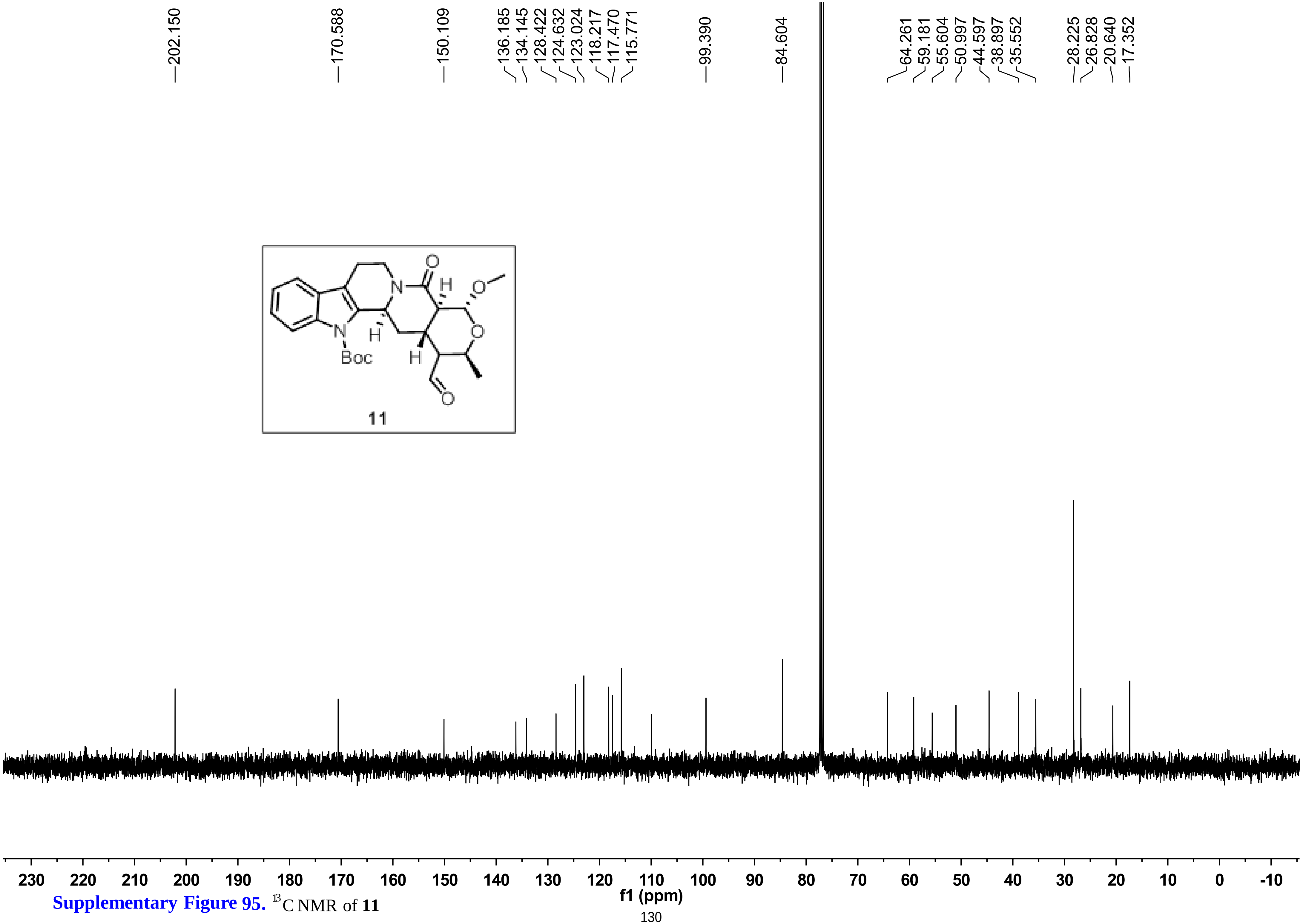

Supplementary Figure 95. ^{13}C NMR of **11**

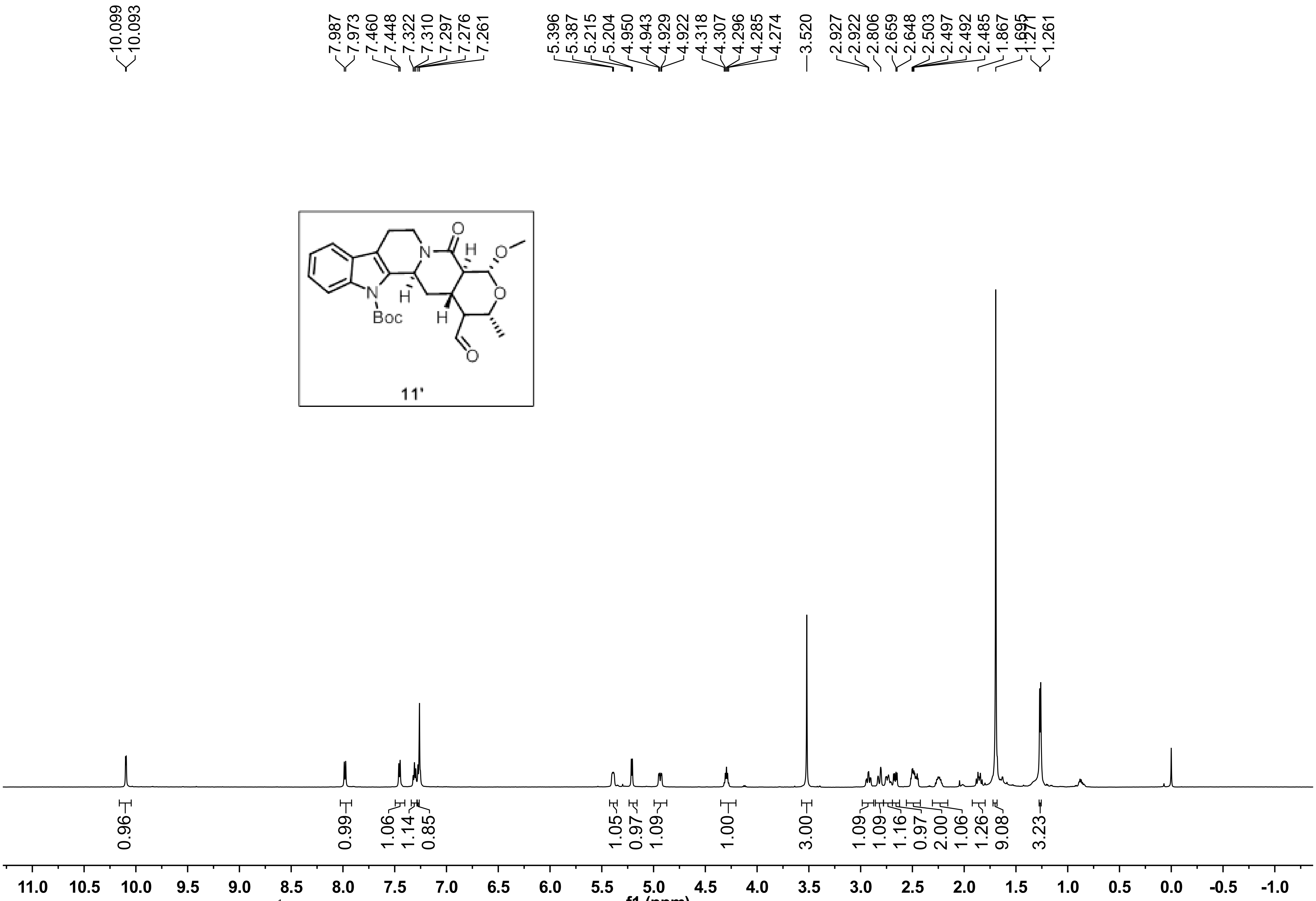

Supplementary Figure 96. ^{1}H NMR of **11'**

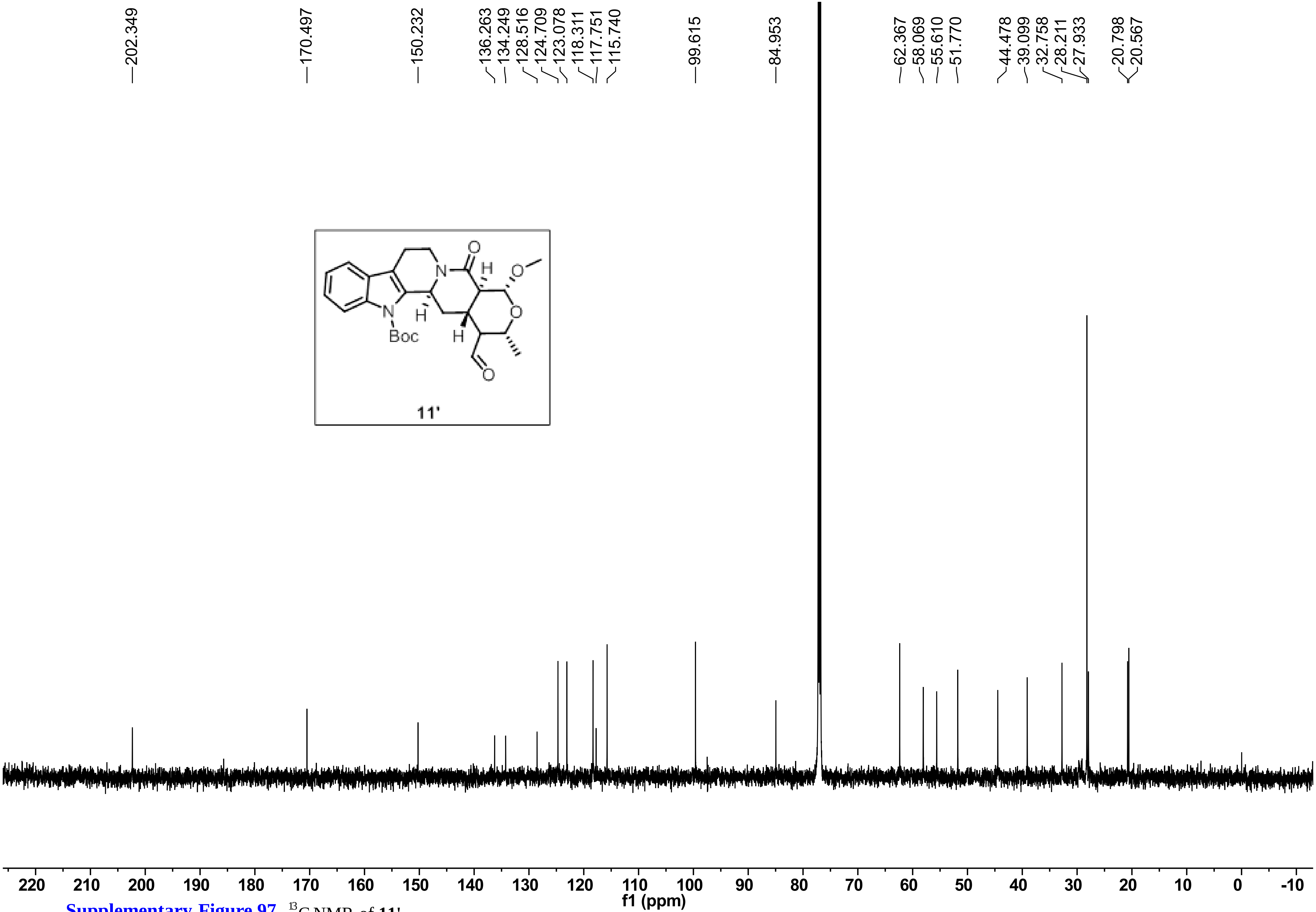

Supplementary Figure 97. ^{13}C NMR of **11'**

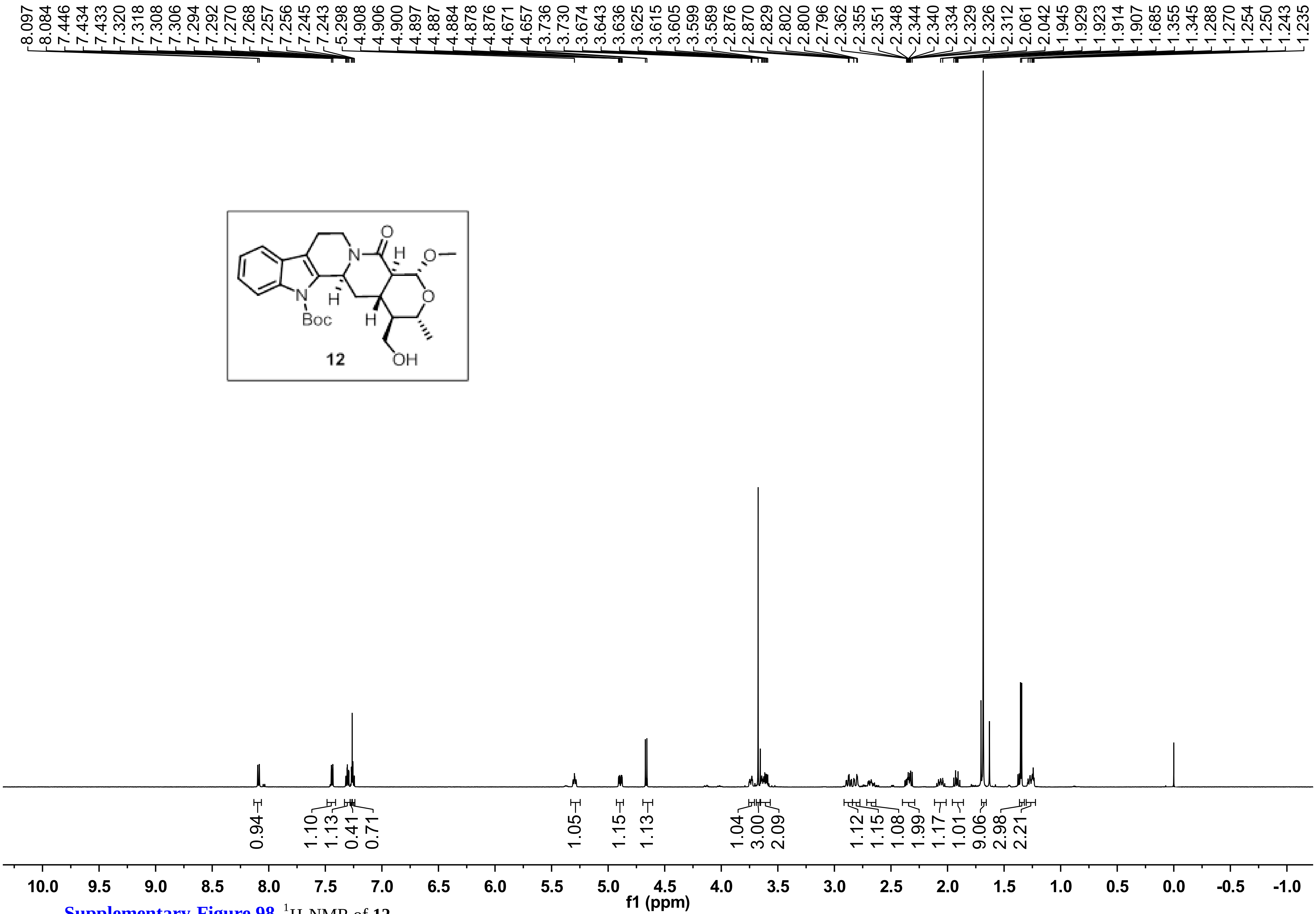

Supplementary Figure 98. ^{1}H NMR of **12**

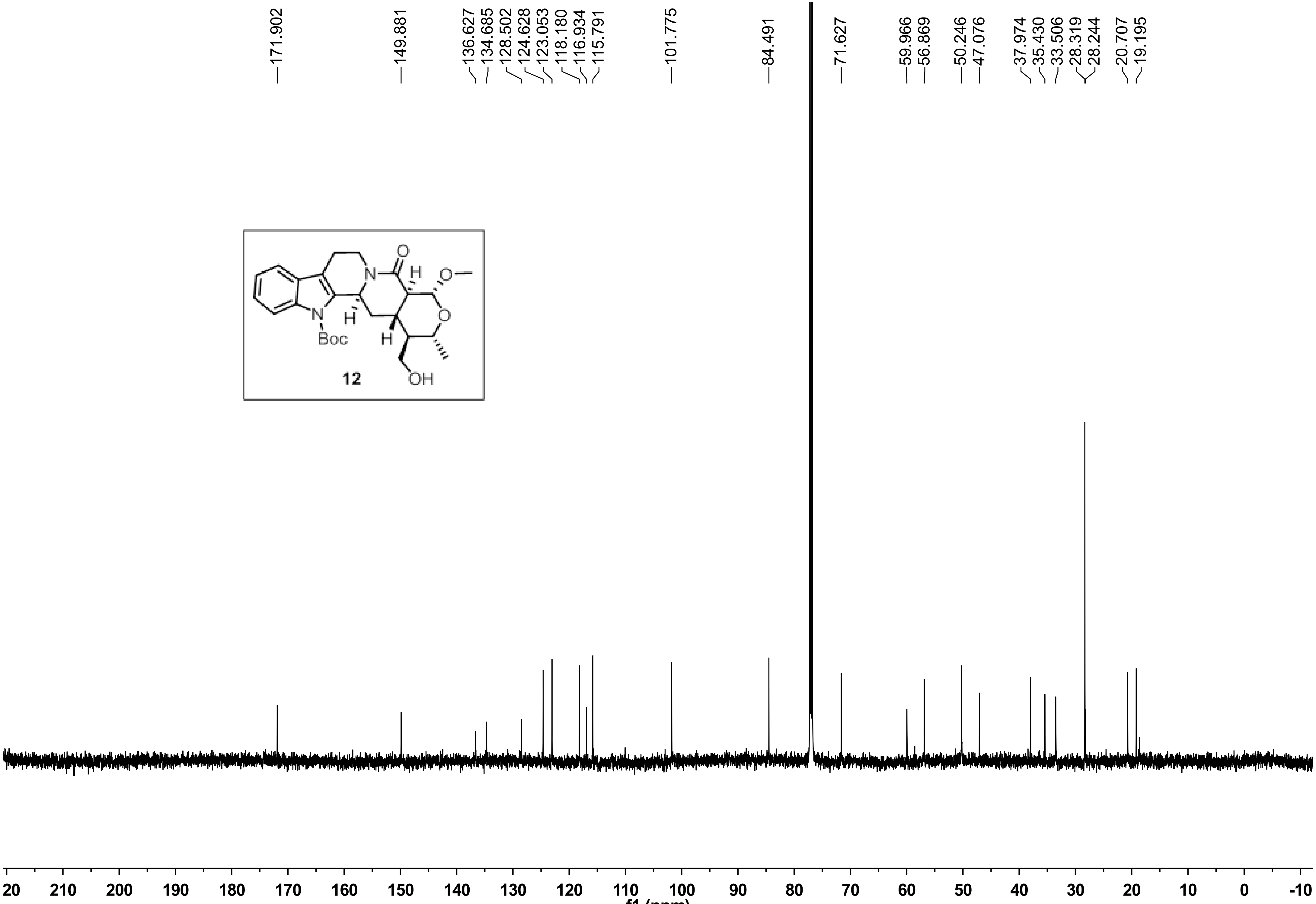

Supplementary Figure 99. ^{13}C NMR of **12**

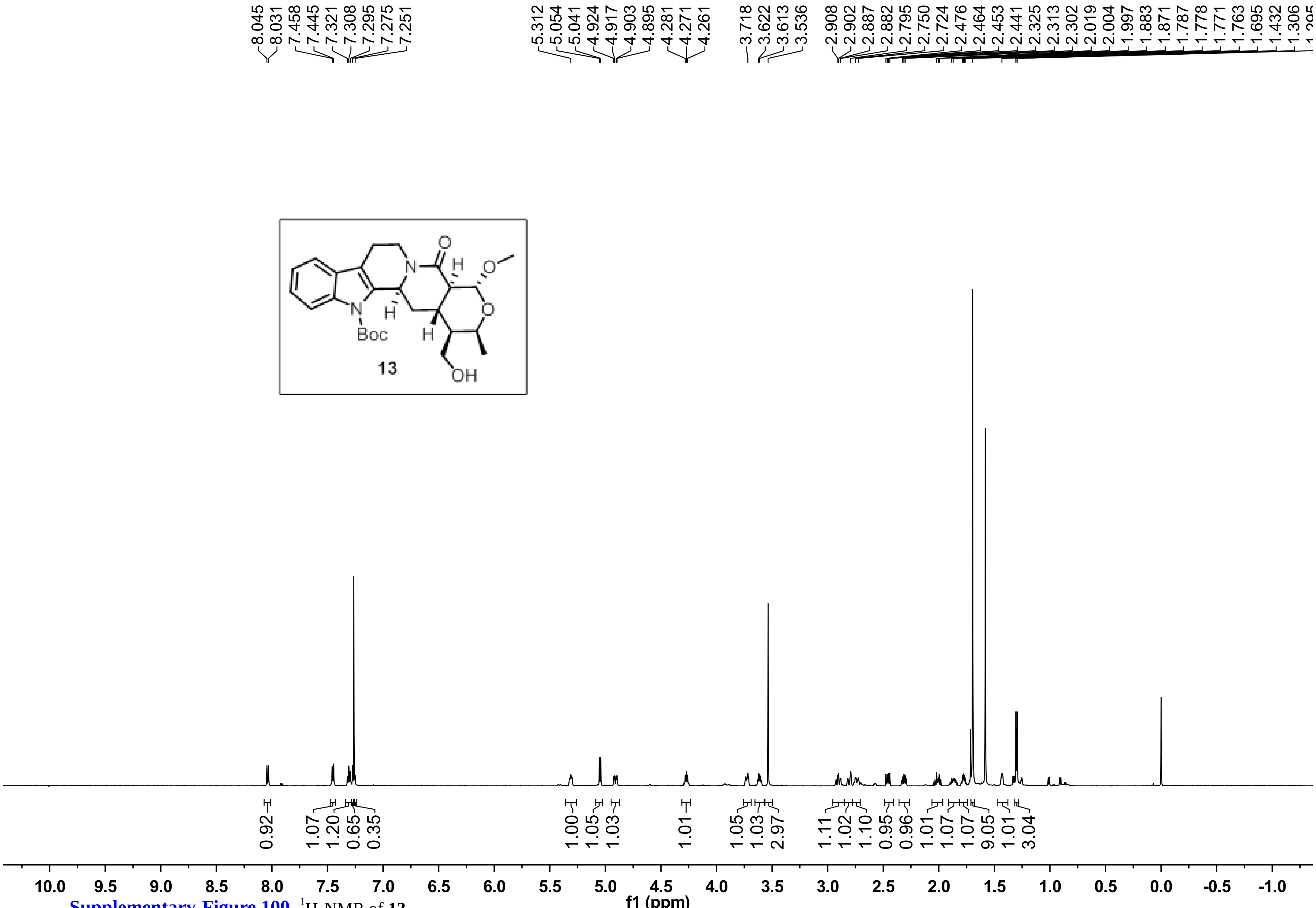

Supplementary Figure 100. ^{1}H NMR of **13**

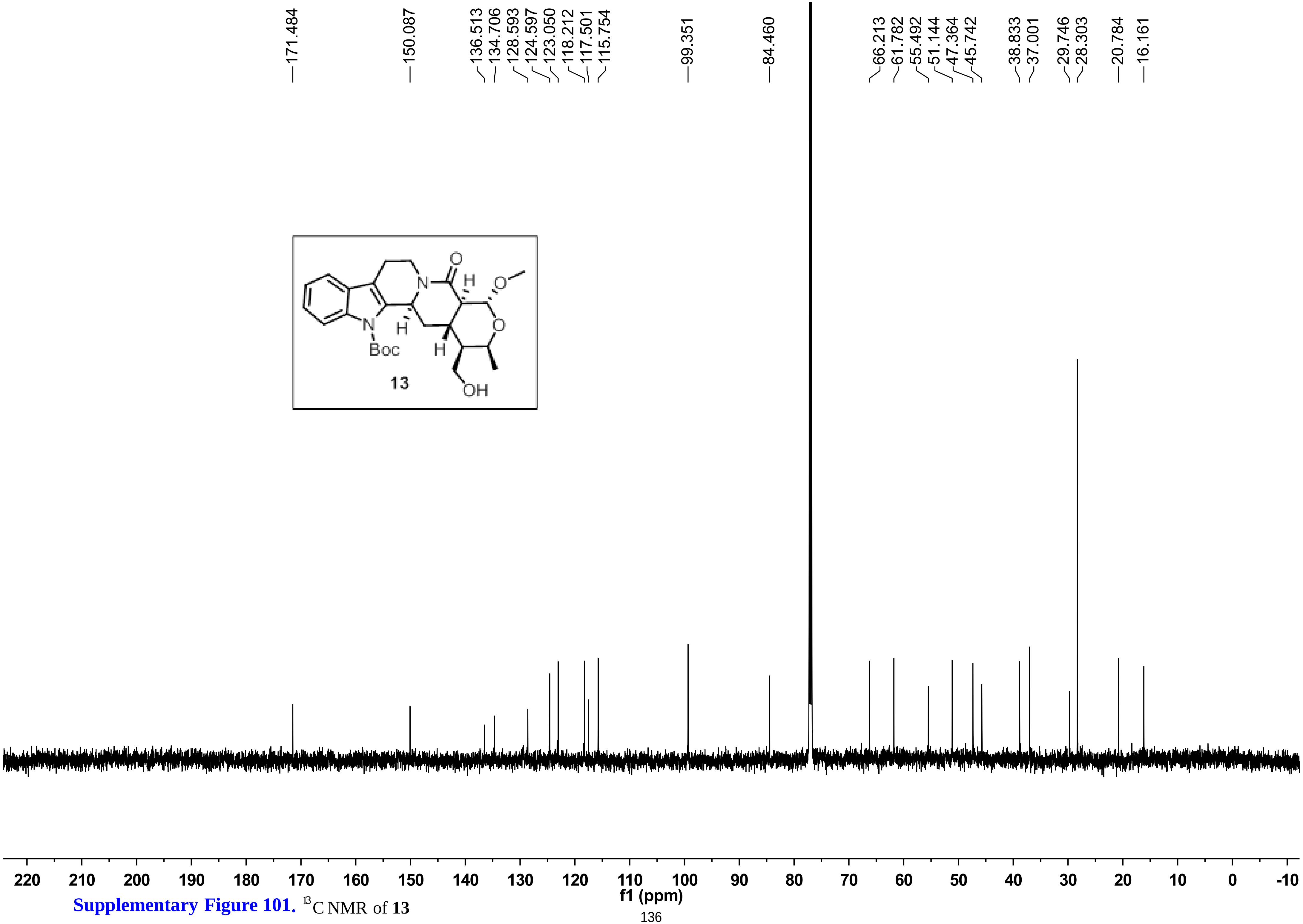

Supplementary Figure 101. ^{13}C NMR of **13**

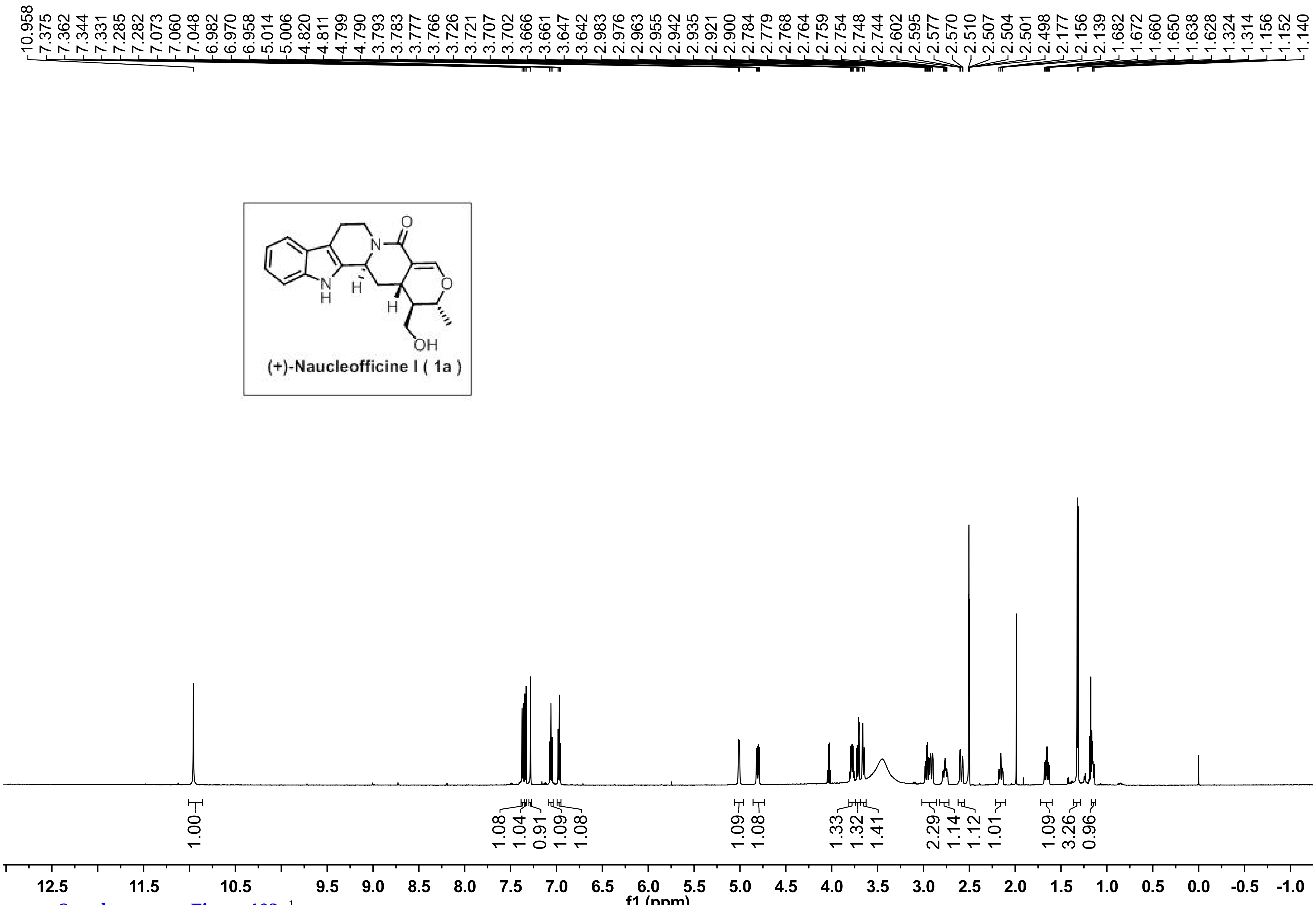

Supplementary Figure 102. ^{1}H NMR of **1a**

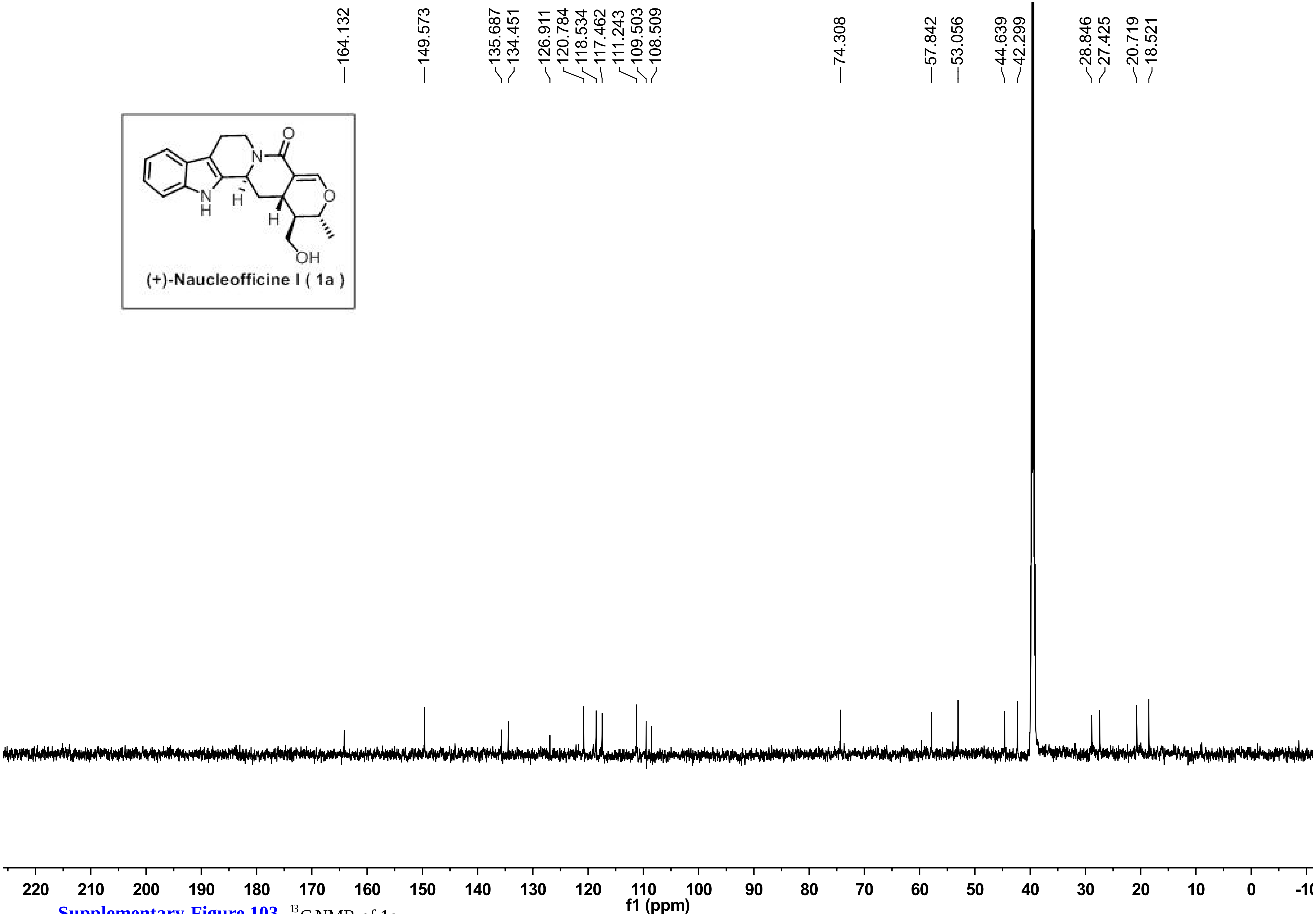

Supplementary Figure 103. ^{13}C NMR of **1a**

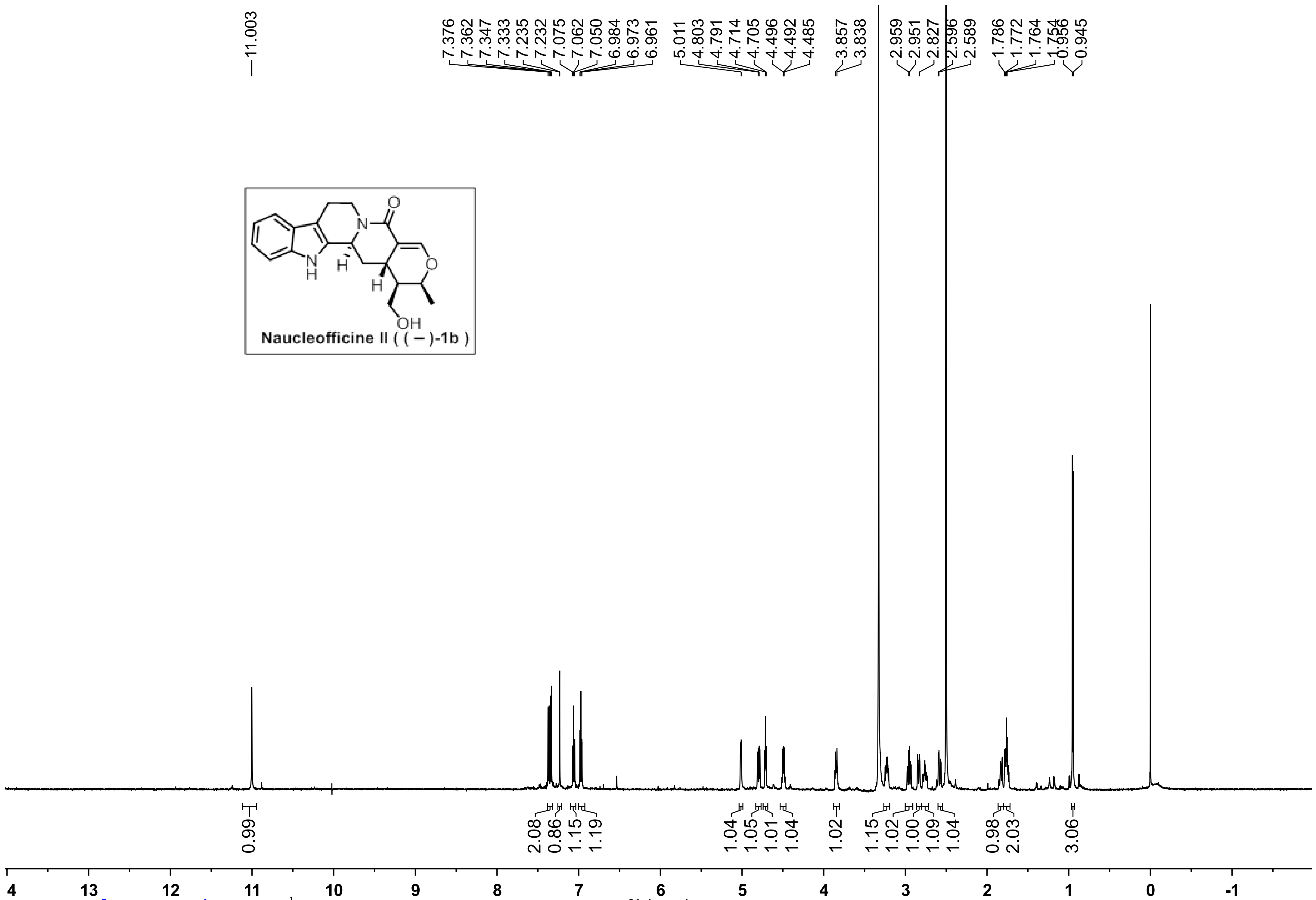

Supplementary Figure 104. ^{1}H NMR of **1b**

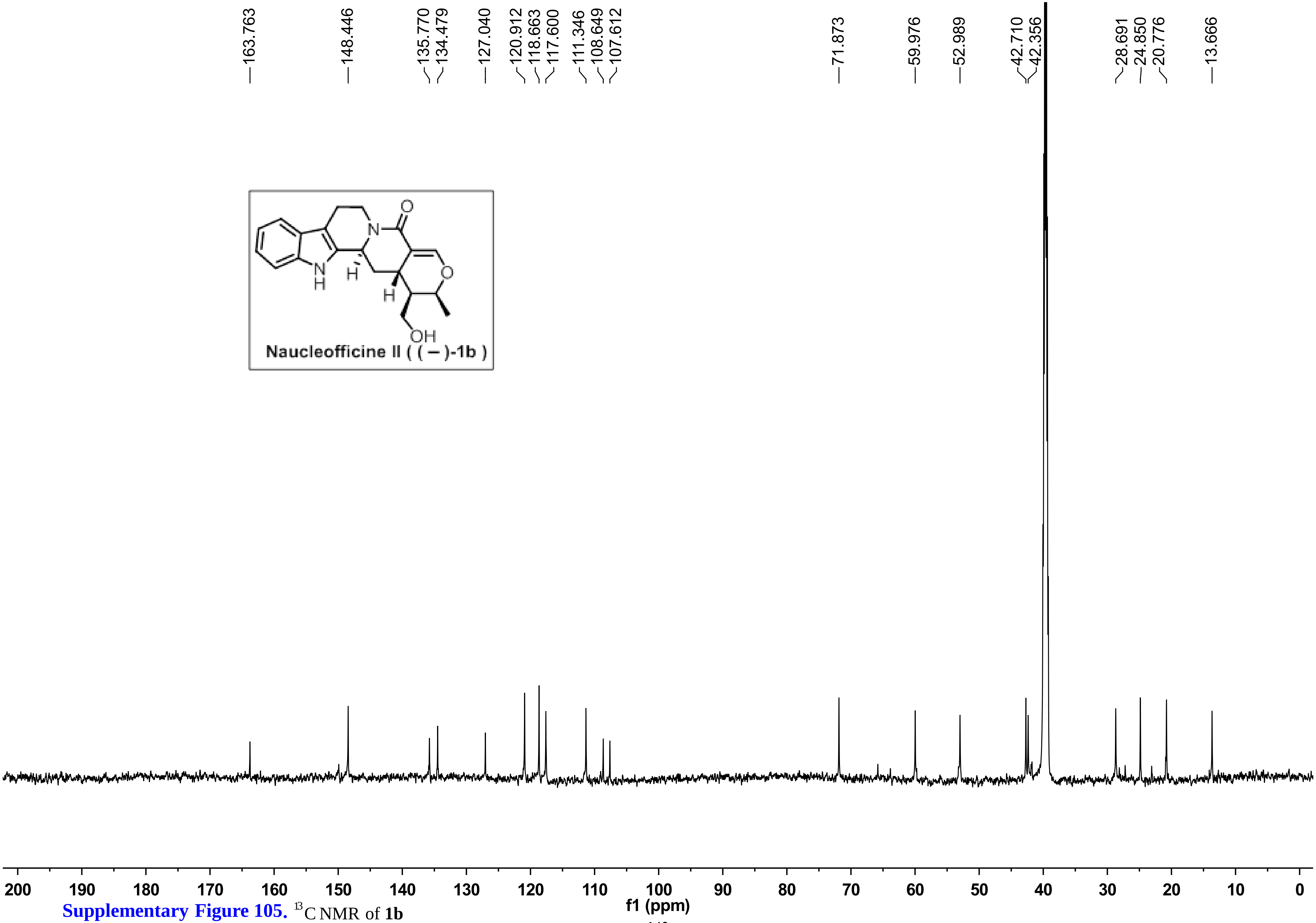

Supplementary Figure 105. ^{13}C NMR of **1b**

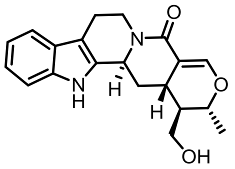

(+)-Naucleofficine I (1a)

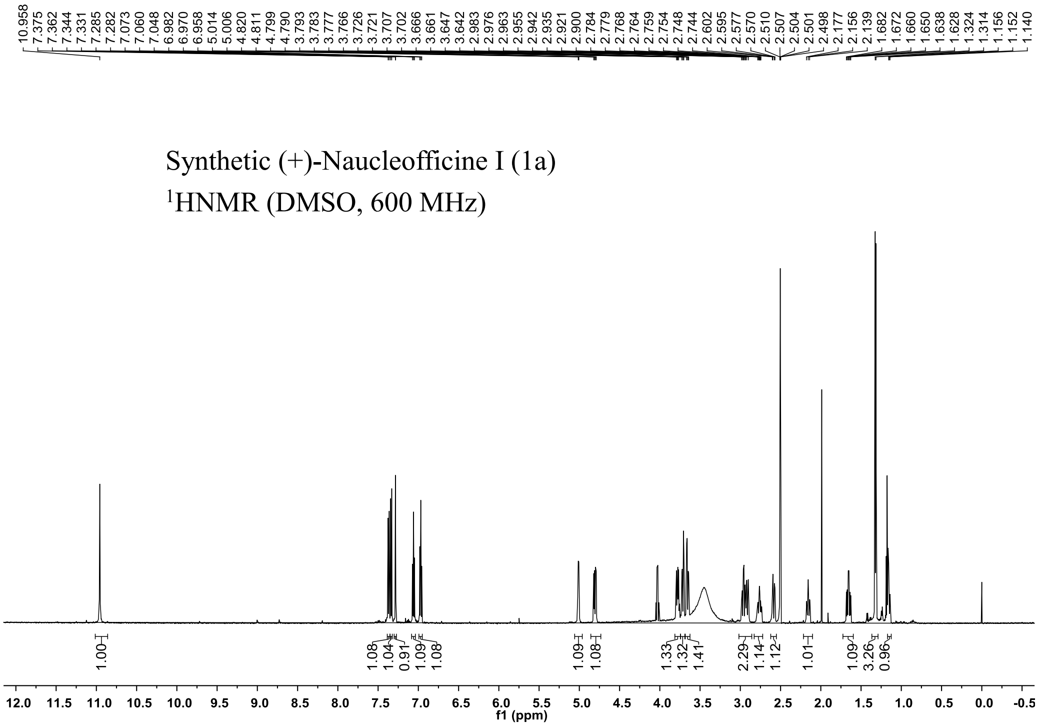

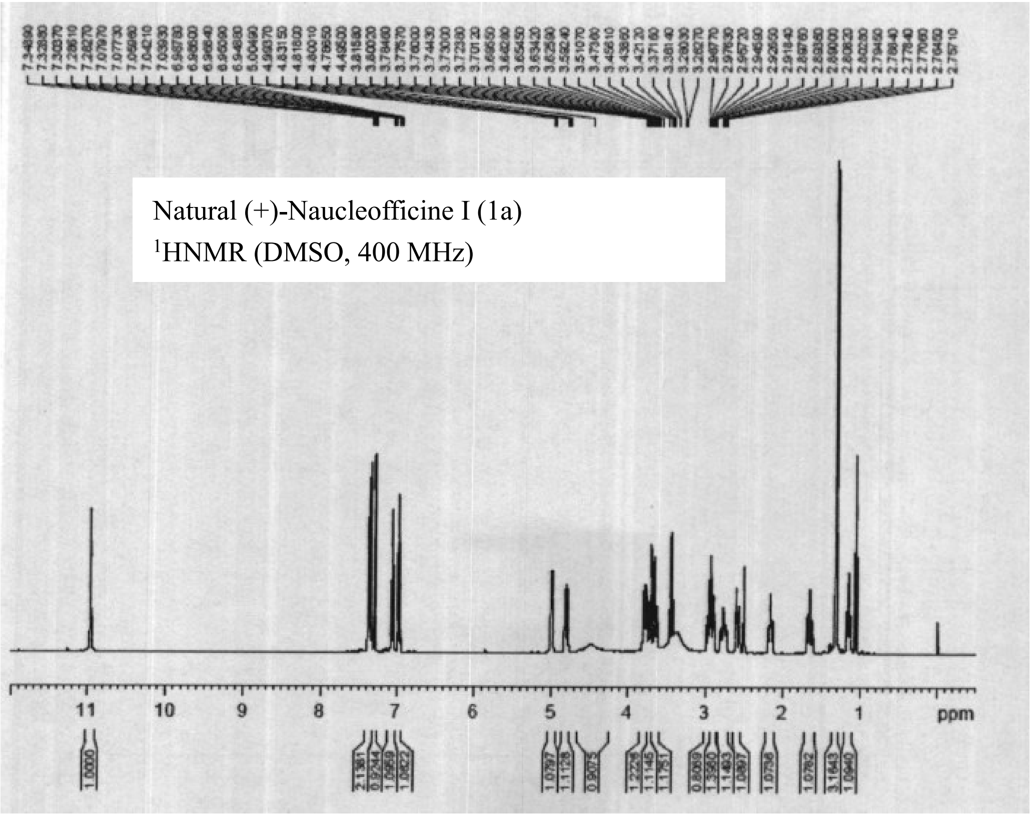

Supplementary Figure 106. Comparison of ^{1}H NMR of **1a**

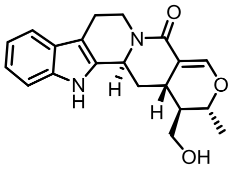

(+)-Naucleofficine I (1a)

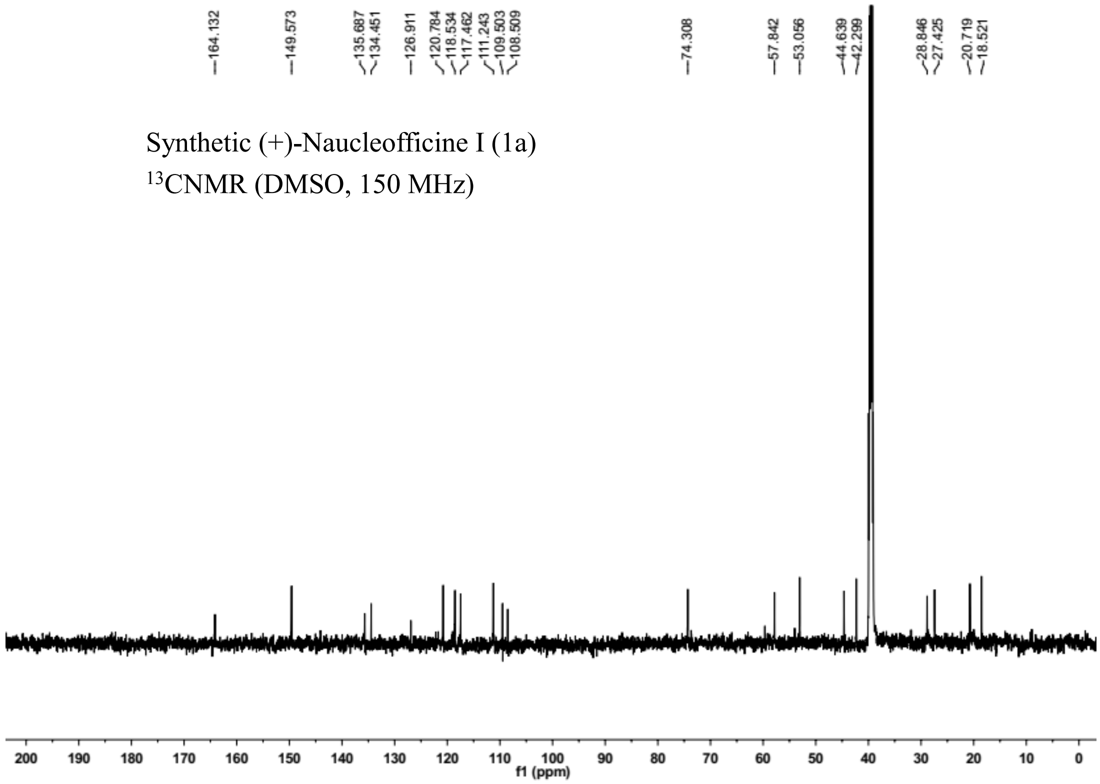

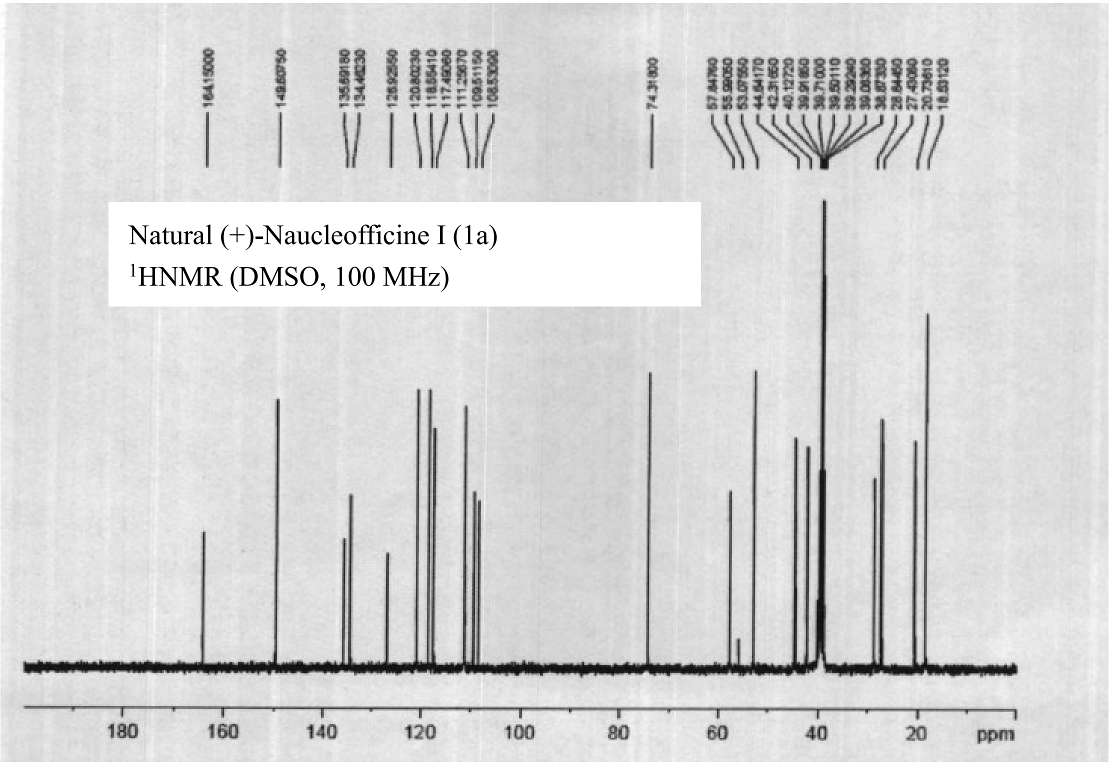

Supplementary Figure 107. Comparison of ^{13}C NMR of **1a**

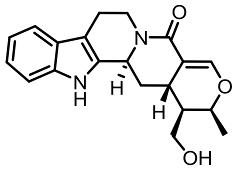

(–)-Naucleofficine II (1b)

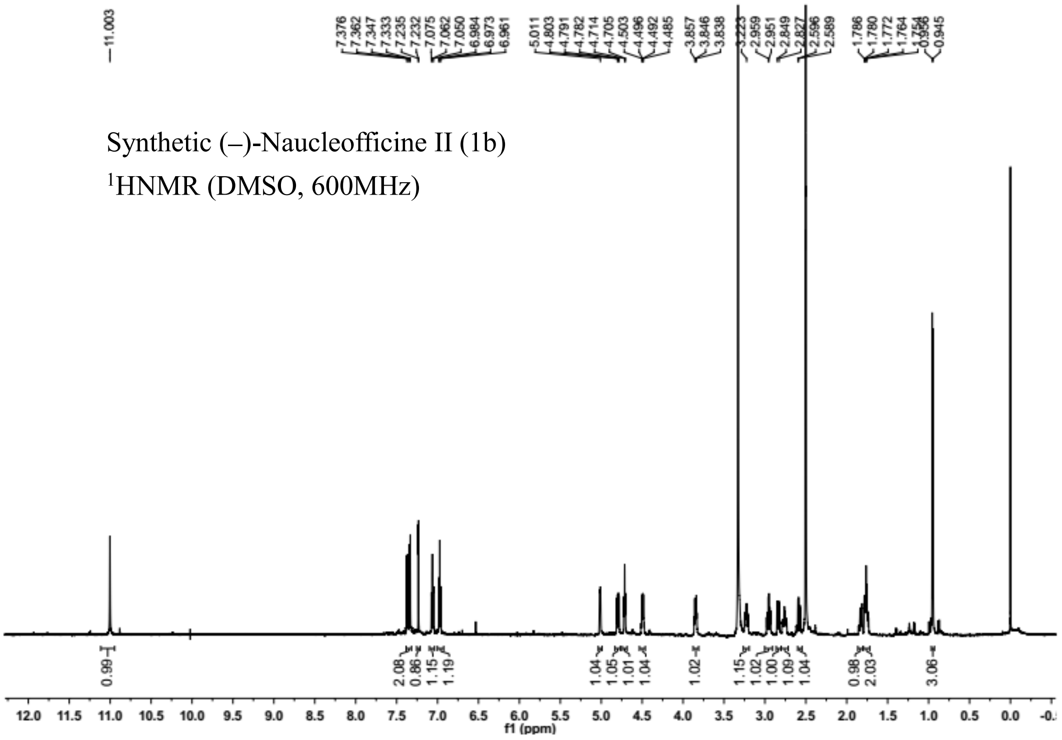

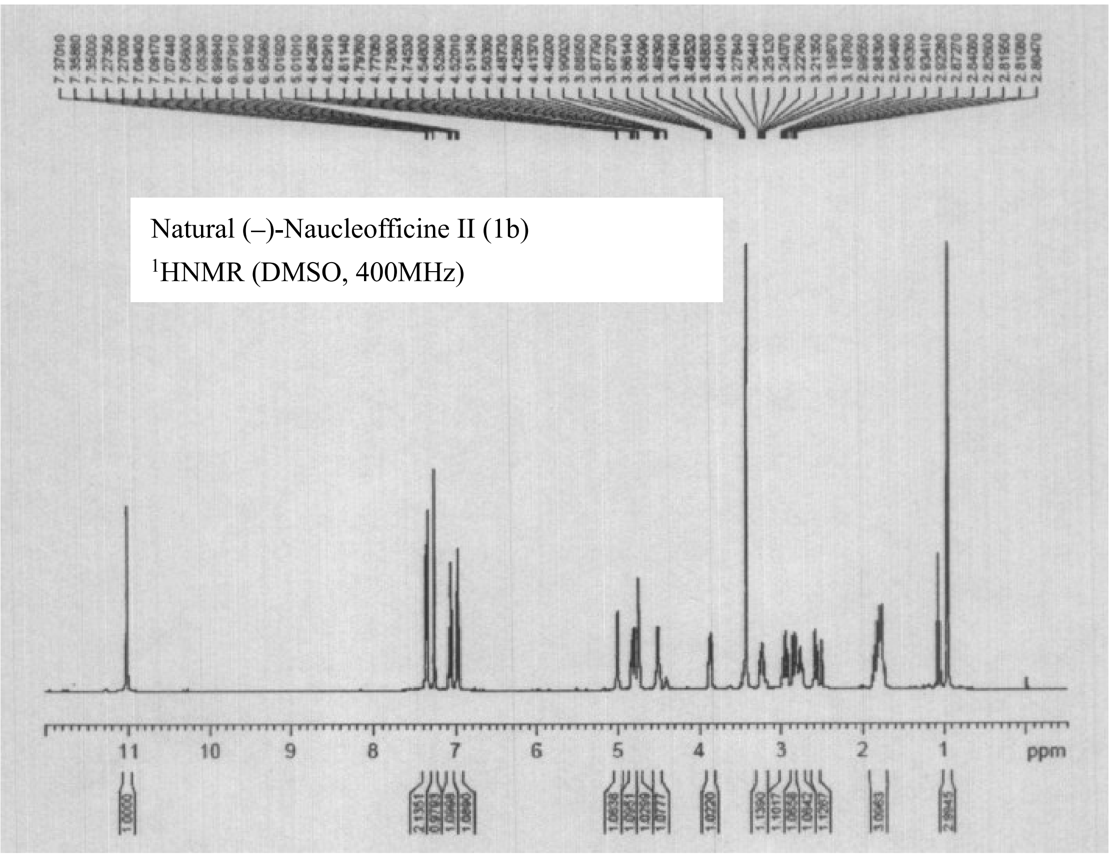

Supplementary Figure 108. Comparison of ^{1}H NMR of **1b**

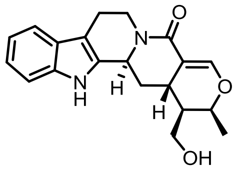

(–)-Naucleofficine II (1b)

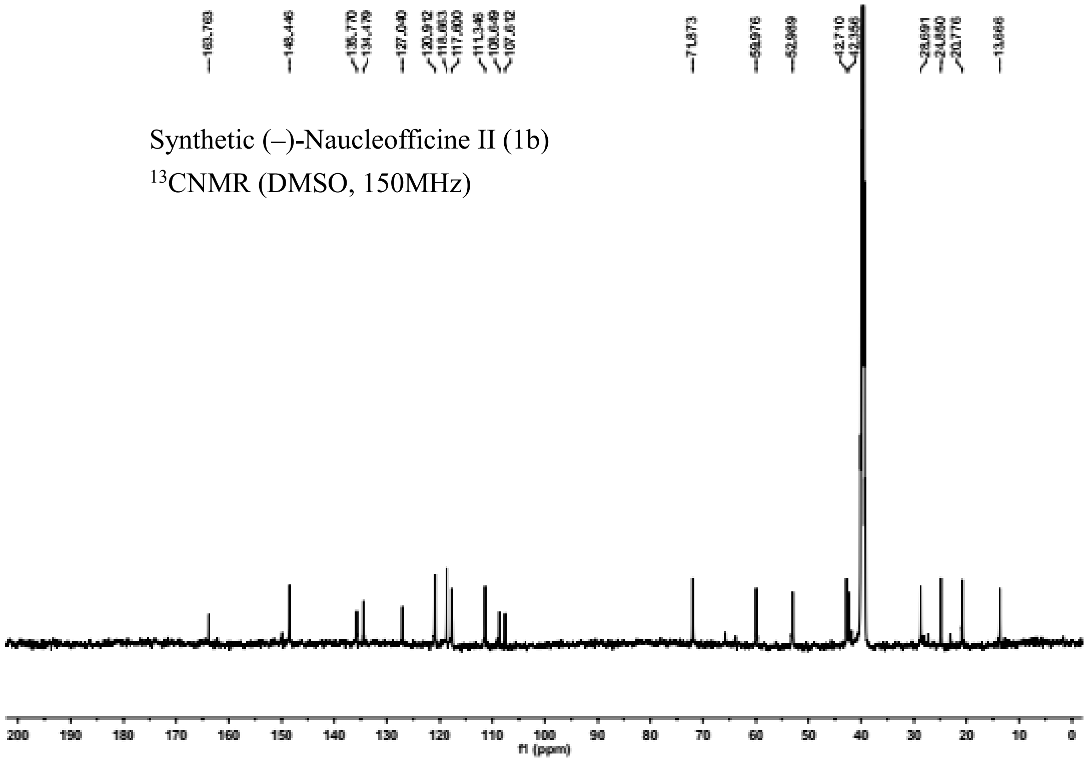

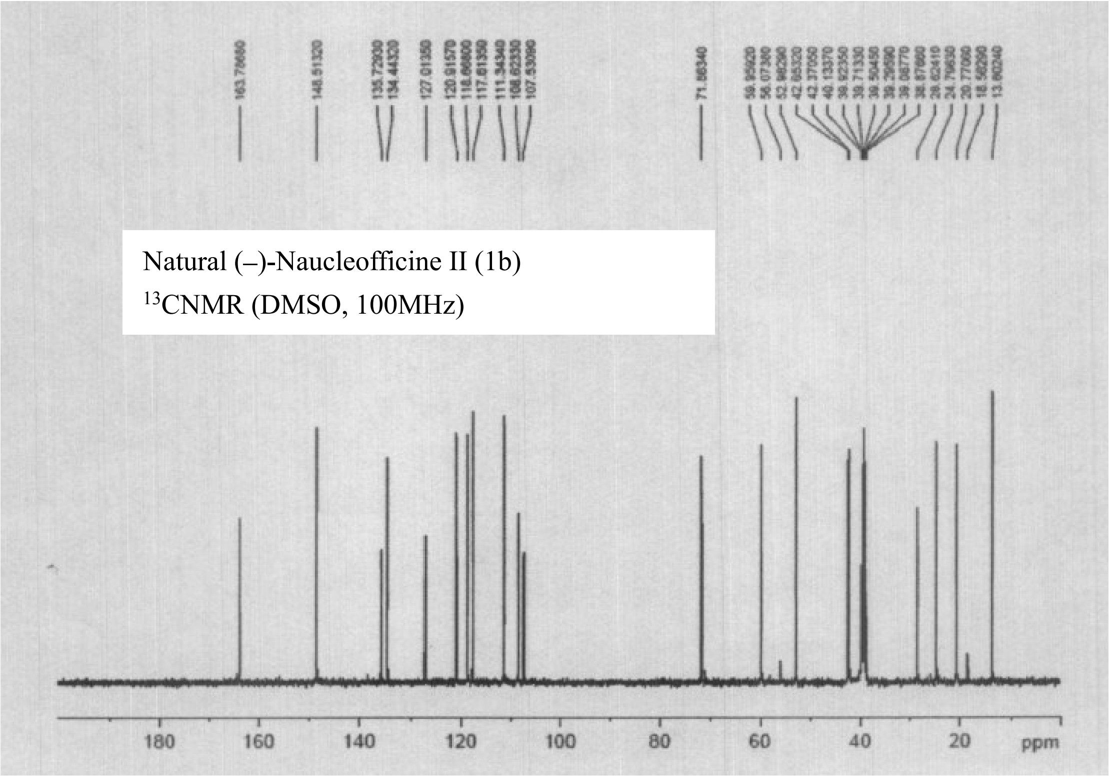

Supplementary Figure 109. Comparison of ^{13}C NMR of **1b**

Sample Information

Sample Name: 5a-rac   Wave Length: PDA 253nm
Column: IA-3

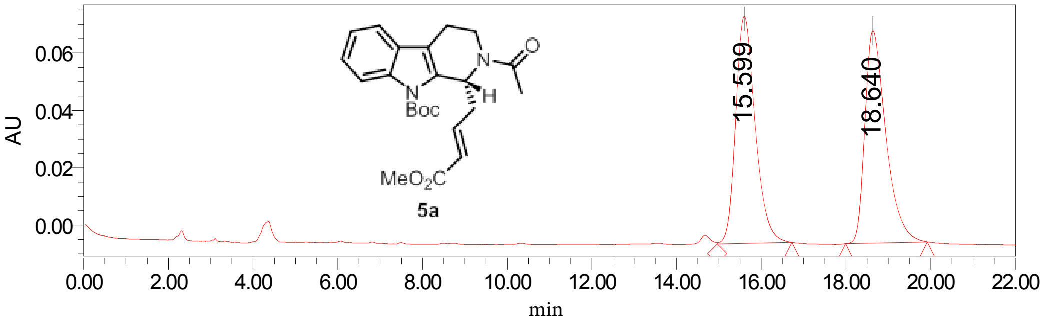

Peak Information:

	RetTime (min)	Area (μV*s)	Area (%)	Height (μV)
1	15.599	2532527.222	49.843	79165
2	18.640	2548479.143	50.157	73970

Sample Name: 5a-ch   Wave Length: PDA 253nm
Column: IA-3

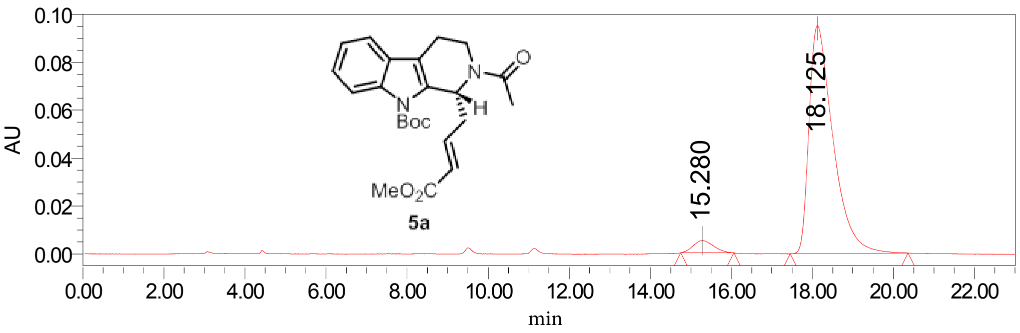

Peak Information:

	RetTime (min)	Area (μV*s)	Area (%)	Height (μV)
1	15.280	181748.335	4.445	5166
2	18.125	3907398.799	95.555	95192

Supplementary Figure 110. HPLC spectrum of **5a**

Sample Information

Sample Name: 5b-rac
Column: IA-3
Wave Length: PDA 250nm

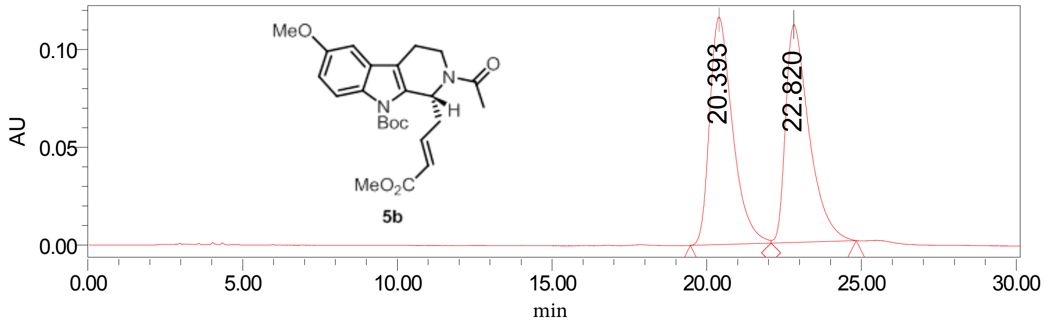

Peak Information:

	RetTime (min)	Area (μV*s)	Area (%)	Height (μV)
1	20.393	5996815.985	50.396	116439
2	22.820	5902665.539	49.604	111391

Sample Name: 5b-ch
Column: IA-3
Wave Length: PDA 250nm

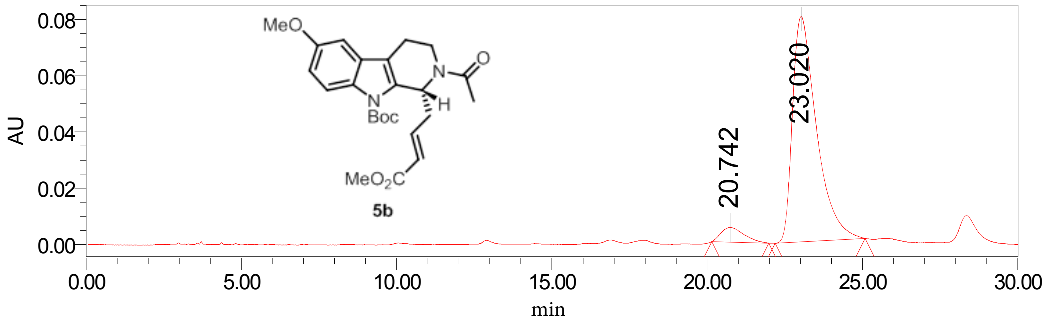

Peak Information:

	RetTime (min)	Area (μV*s)	Area (%)	Height (μV)
1	20.742	265698.993	5.809	5170
2	23.020	4308435.791	94.191	80108

Supplementary Figure 111. HPLC spectrum of **5b**

Sample Information

Sample Name: 5c-rac
Column: IA-3
Wave Length: PDA 270nm

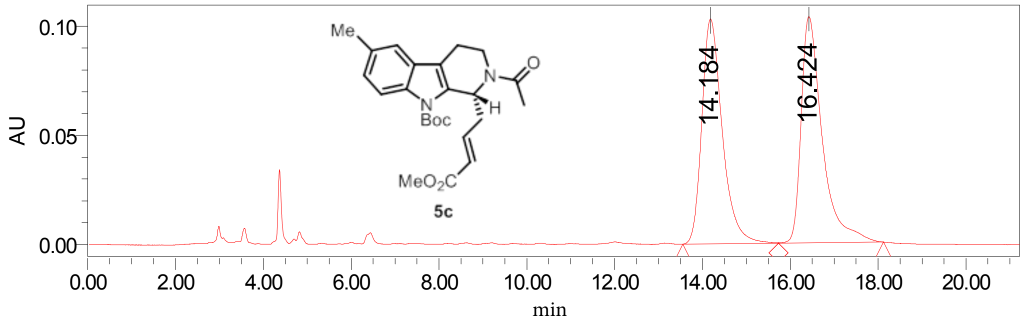

Peak Information:

	RetTime (min)	Area (μV*s)	Area (%)	Height (μV)
1	14.184	3322750.295	49.106	103006
2	16.424	3443738.316	50.894	103706

Sample Name: 5c-ch
Column: IA-3
Wave Length: PDA 270nm

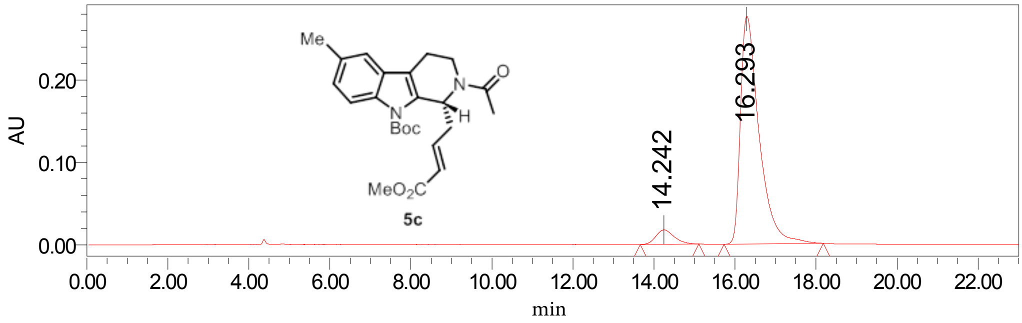

Peak Information:

	RetTime (min)	Area (μV*s)	Area (%)	Height (μV)
1	14.242	556003.989	5.934	17557
2	16.293	8814399.525	94.066	276561

Supplementary Figure 112. HPLC spectrum of **5c**

Sample Information

Sample Name: 5d-rac　　Wave Length: PDA 250nm
Column: IA-3

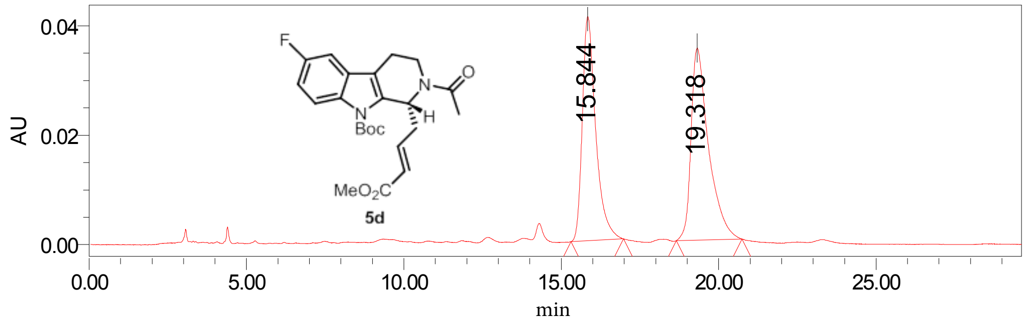

Peak Information:

	RetTime (min)	Area (μV*s)	Area (%)	Height (μV)
1	15.844	1214768.886	47.203	41057
2	19.318	1358747.039	52.797	35148

Sample Name: 5d-ch　　Wave Length: PDA 250nm
Column: IA-3

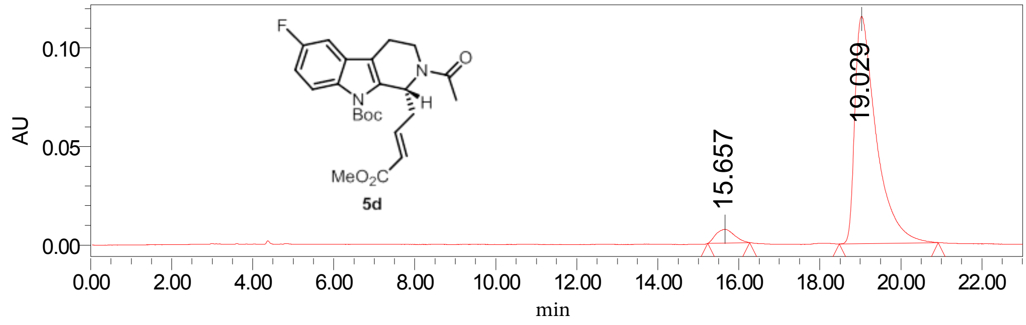

Peak Information:

	RetTime (min)	Area (μV*s)	Area (%)	Height (μV)
1	15.657	219335.678	4.932	6914
2	19.029	4228045.902	95.068	115348

Supplementary Figure 113. HPLC spectrum of **5d**

Sample Information

Sample Name: 5e-rac
Column: IA-3
Wave Length: PDA 250nm

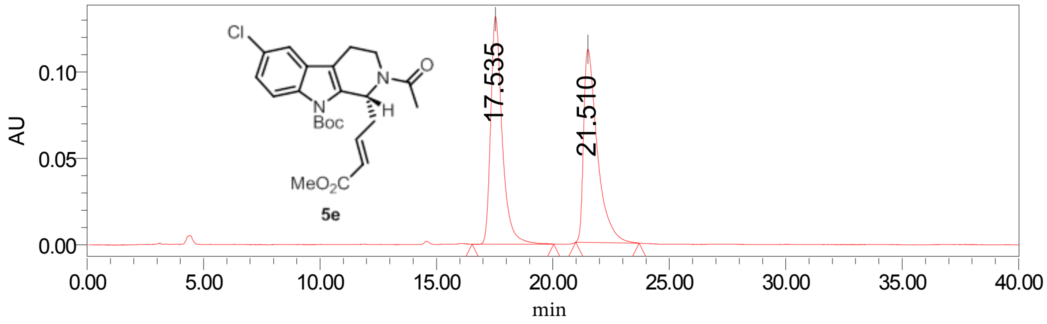

Peak Information:

	RetTime (min)	Area (μV*s)	Area (%)	Height (μV)
1	17.535	4526218.536	49.855	131695
2	21.510	4552616.134	50.145	111726

Sample Name: 5e-ch
Column: IA-3
Wave Length: PDA 250nm

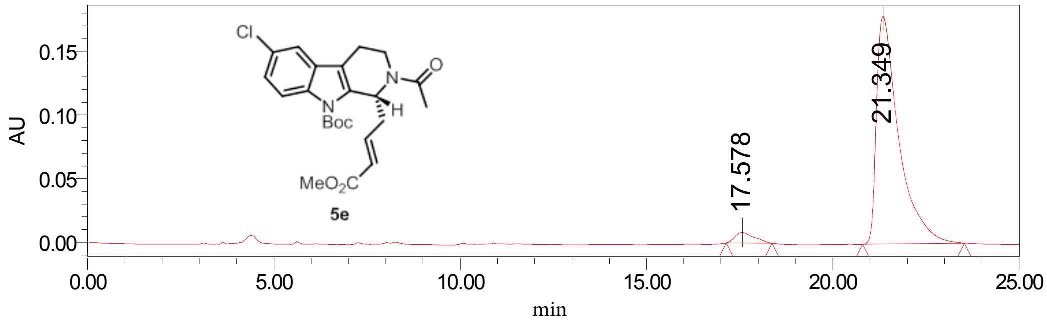

Peak Information:

	RetTime (min)	Area (μV*s)	Area (%)	Height (μV)
1	17.578	303734.983	3.921	8433
2	21.349	7442192.785	96.079	178812

Supplementary Figure 114. HPLC spectrum of **5e**

Sample Information

Sample Name: 5f-rac   Wave Length: PDA 275nm
Column: IA-3

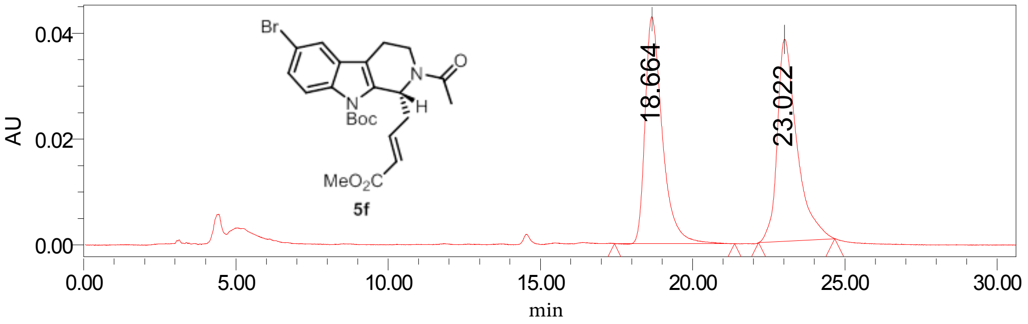

Peak Information:

	RetTime (min)	Area (μV*s)	Area (%)	Height (μV)
1	18.664	1684009.141	49.579	42889
2	23.022	1712638.659	50.421	38160

Sample Name: 5f-ch   Wave Length: PDA 275nm
Column: IA-3

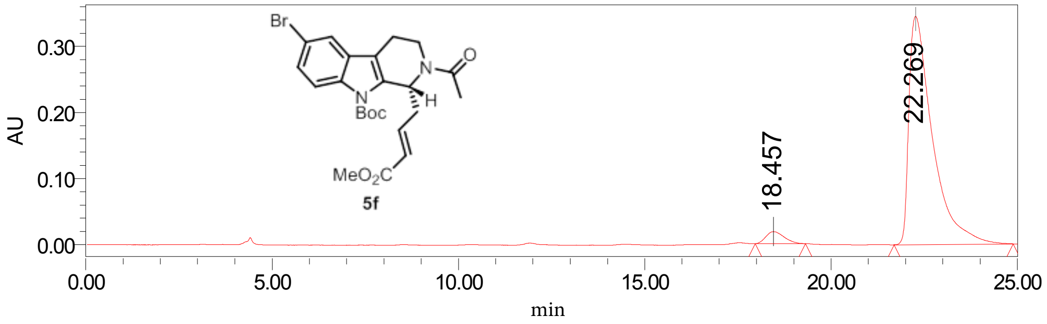

Peak Information:

	RetTime (min)	Area (μV*s)	Area (%)	Height (μV)
1	18.457	628129.348	3.971	18418
2	22.269	15187818.942	96.029	345666

Supplementary Figure 115. HPLC spectrum of **5f**

Sample Information

Sample Name: 5g-rac   Wave Length: PDA 280nm
Column: IA-3

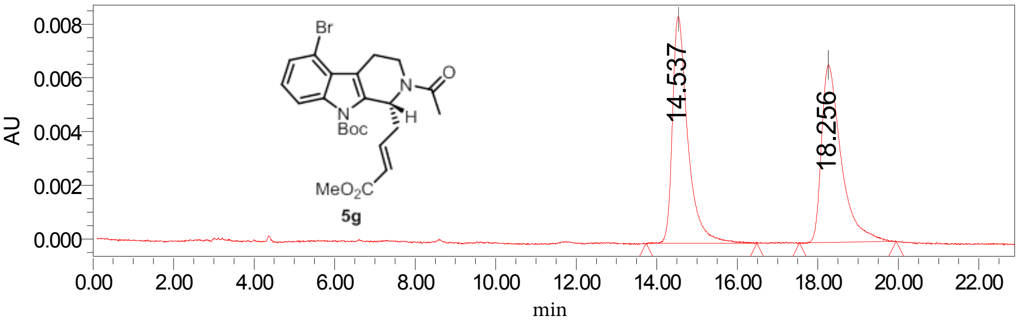

Peak Information:

	RetTime (min)	Area (μV*s)	Area (%)	Height (μV)
1	14.537	223389.576	49.883	8439
2	18.256	224434.885	50.117	6619

Sample Name: 5g-ch   Wave Length: PDA 280nm
Column: IA-3

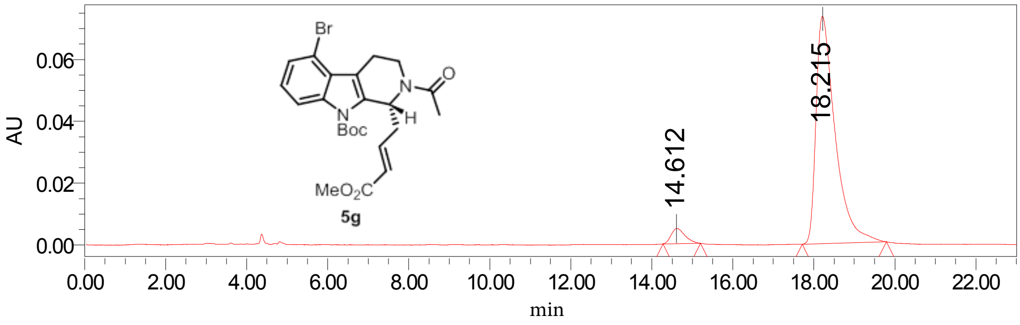

Peak Information:

	RetTime (min)	Area (μV*s)	Area (%)	Height (μV)
1	14.612	116574.347	4.598	4913
2	18.215	2418661.196	95.402	73584

Supplementary Figure 116. HPLC spectrum of **5g**

Sample Information

Sample Name: 5h-rac
Column: IA-3

Wave Length: PDA 250nm

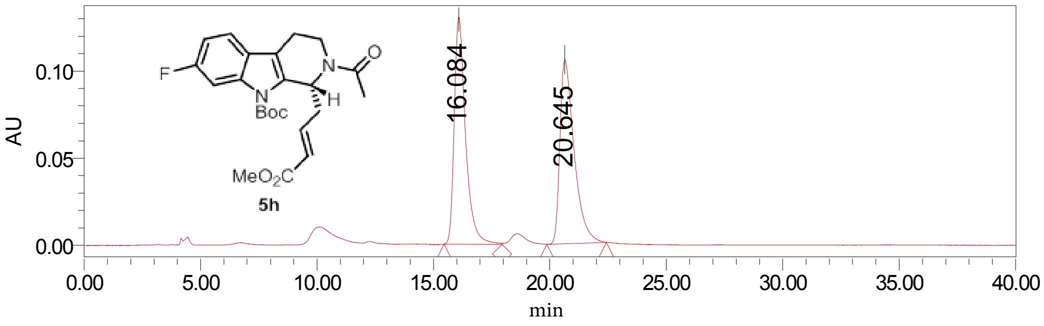

Peak Information:

	RetTime (min)	Area (μV*s)	Area (%)	Height (μV)
1	16.084	4437520.439	49.645	130400
2	20.645	4501012.506	50.355	105601

Sample Name: 5h-ch
Column: IA-3

Wave Length: PDA 250nm

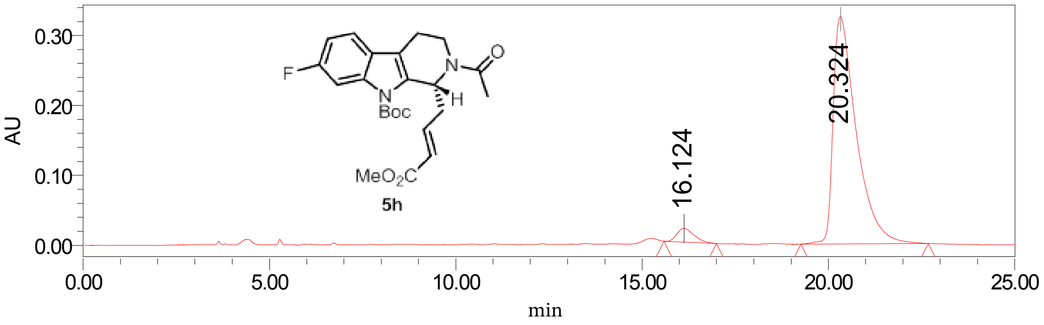

Peak Information:

	RetTime (min)	Area (μV*s)	Area (%)	Height (μV)
1	16.124	633713.459	4.304	19539
2	20.324	14088518.852	95.696	325501

Supplementary Figure 117. HPLC spectrum of **5h**

Sample Information

Sample Name: 5i-rac Wave Length: PDA 250nm
Column: IA-3

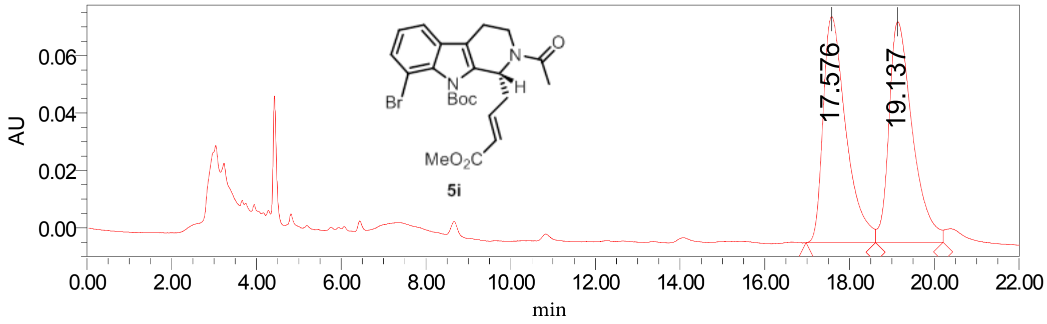

Peak Information:

	RetTime (min)	Area (μV*s)	Area (%)	Height (μV)
1	17.576	2955898.262	49.835	78750
2	19.137	2975475.501	50.165	76875

Sample Name: 5i-ch Wave Length: PDA 250nm
Column: IA-3

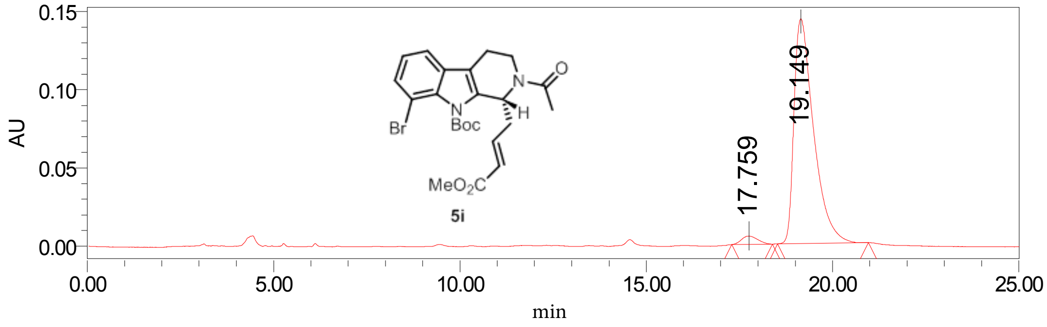

Peak Information:

	RetTime (min)	Area (μV*s)	Area (%)	Height (μV)
1	17.759	161878.706	2.994	5386
2	19.149	5245528.569	97.006	143691

Supplementary Figure 118. HPLC spectrum of **5i**

Sample Information

Sample Name: 5j-rac Wave Length: PDA 245nm
Column: IA-3

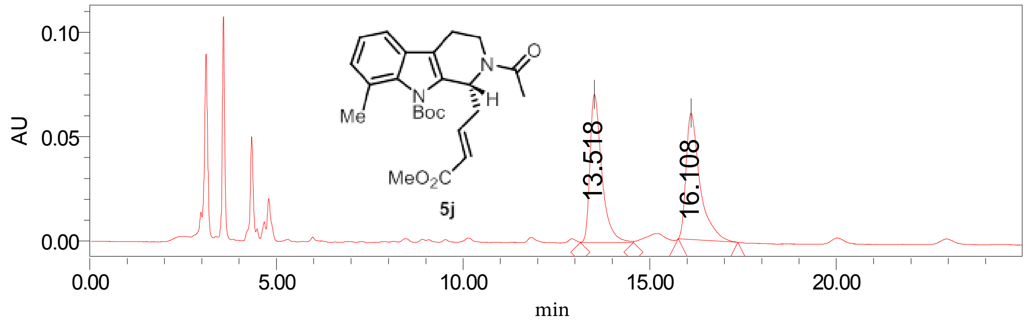

Peak Information:

	RetTime (min)	Area (µV*s)	Area (%)	Height (µV)
1	13.518	1562566.167	49.781	71048
2	16.108	1576324.008	50.219	60554

Sample Name: 5j-ch Wave Length: PDA 245nm
Column: IA-3

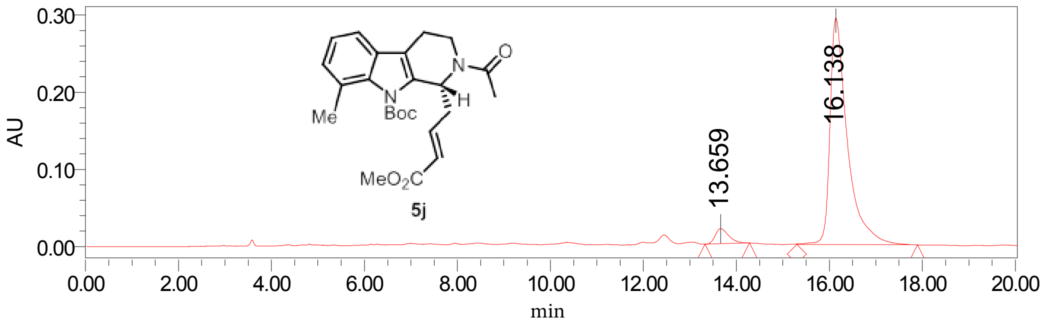

Peak Information:

	RetTime (min)	Area (µV*s)	Area (%)	Height (µV)
1	13.659	400700.145	4.954	19750
2	16.138	7688029.332	95.046	293711

Supplementary Figure 119. HPLC spectrum of **5j**

Sample Information

Sample Name: 5k-rac Wave Length: PDA 250nm
Column: OD-H

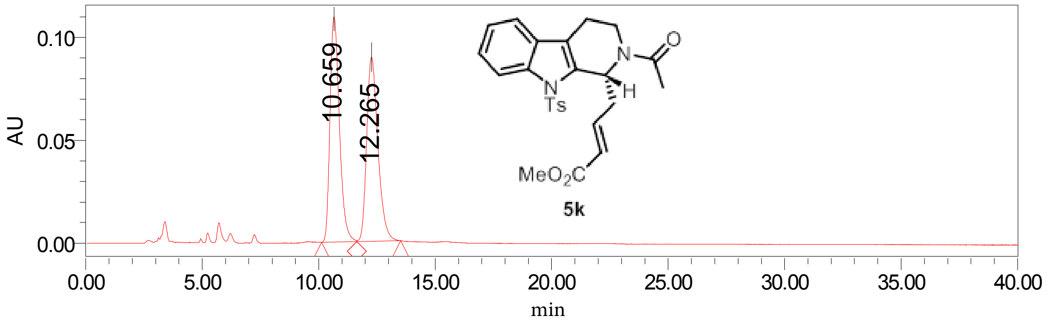

Peak Information:

	RetTime (min)	Area (μV*s)	Area (%)	Height (μV)
1	10.659	3044523.857	50.330	109672
2	12.265	3004577.603	49.670	89598

Sample Name: 5k-ch Wave Length: PDA 250nm
Column: OD-H

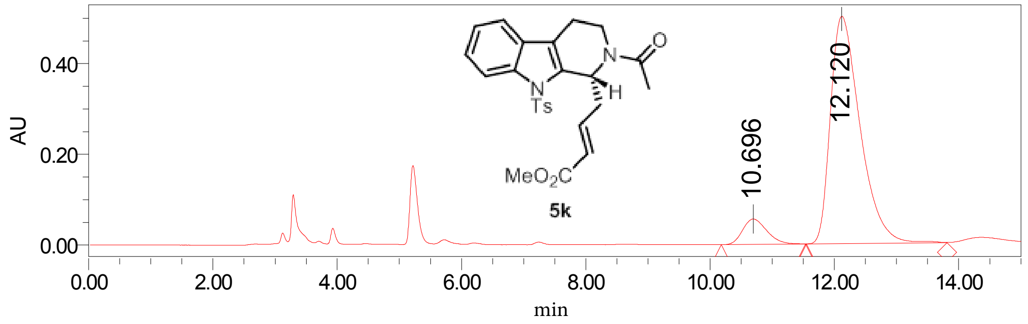

Peak Information:

	RetTime (min)	Area (μV*s)	Area (%)	Height (μV)
1	10.696	1515501.299	8.363	56050
2	12.120	16604961.294	91.637	501184

Supplementary Figure 120. HPLC spectrum of **5k**

Sample Information

Sample Name: 5l-rac Wave Length: PDA 280nm
Column: IA-3

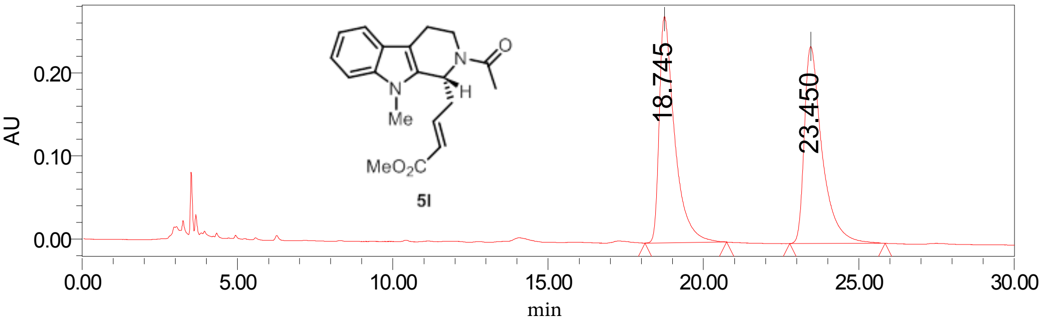

Peak Information:

	RetTime (min)	Area (μV*s)	Area (%)	Height (μV)
1	18.745	9367784.185	49.778	272462
2	23.450	9451224.814	50.222	237318

Sample Name: 5l-ch Wave Length: PDA 280nm
Column: IA-3

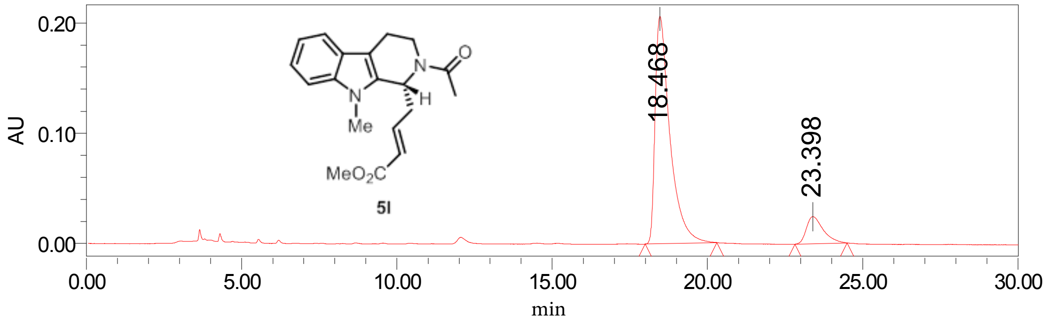

Peak Information:

	RetTime (min)	Area (μV*s)	Area (%)	Height (μV)
1	18.468	6809936.585	88.051	206405
2	23.398	924151.060	11.949	24577

Supplementary Figure 121. HPLC spectrum of **5l**

Sample Name: 5m-rac Wave Length: PDA 246nm
Column: IF-3

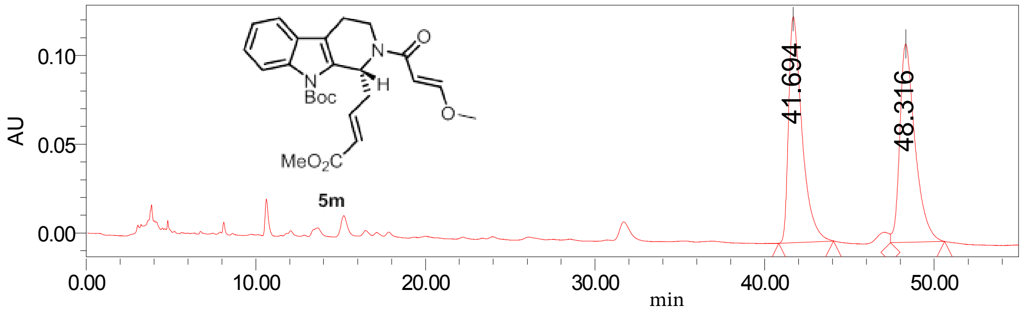

Peak Information:

	RetTime (min)	Area (μV*s)	Area (%)	Height (μ V)
1	41.694	6991663.768	50.006	127381
2	48.316	6989883.341	49.994	111847

Sample Name: 5m-ch Wave Length: PDA 246nm
Column: IF-3

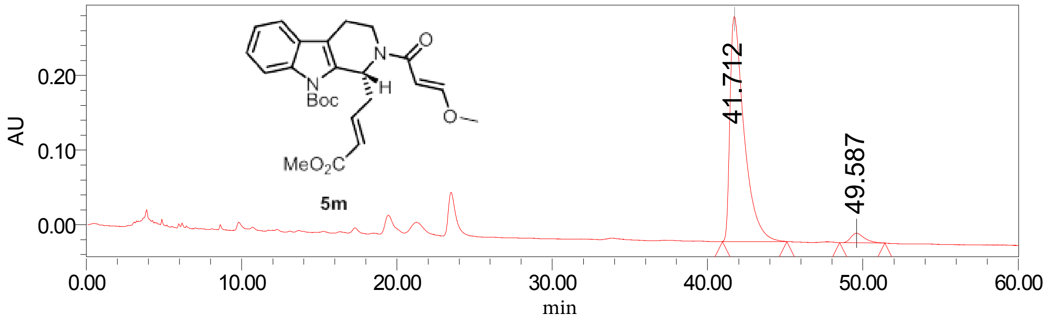

Peak Information:

	RetTime (min)	Area (μV*s)	Area (%)	Height (μ V)
1	41.712	19122851.376	95.969	301064
2	49.587	803300.797	4.031	12914

Supplementary Figure 122. HPLC spectrum of **5m**

Sample Information

Sample Name: 5n-rac Wave Length: PDA 220nm
Column: IA-3

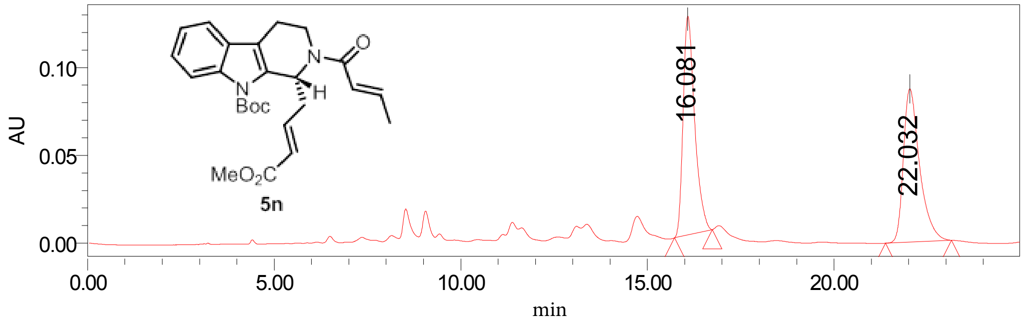

Peak Information:

	RetTime (min)	Area (μV*s)	Area (%)	Height (μV)
1	16.081	2672719.772	50.335	124584
2	22.032	2637182.540	49.665	87362

Sample Name: 5n-ch Wave Length: PDA 220nm
Column: IA-3

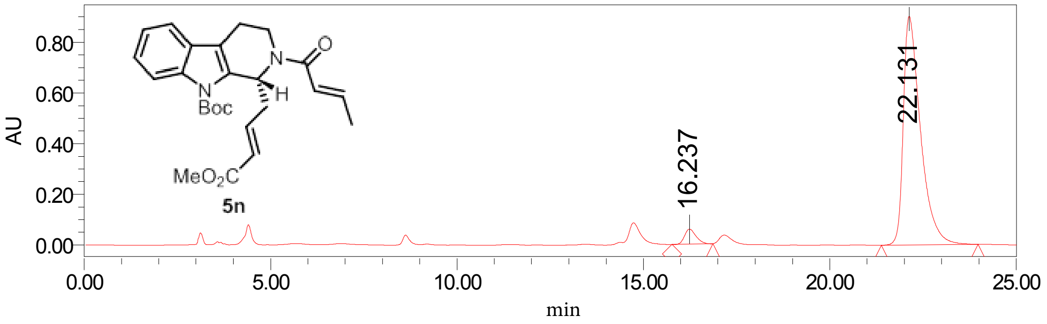

Peak Information:

	RetTime (min)	Area (μV*s)	Area (%)	Height (μV)
1	16.237	1250735.179	4.143	58703
2	22.131	28940912.583	95.857	902406

Supplementary Figure 123. HPLC spectrum of **5n**

Sample Information

Sample Name: 5q-rac Wave Length: PDA 220nm
Column: OD-H

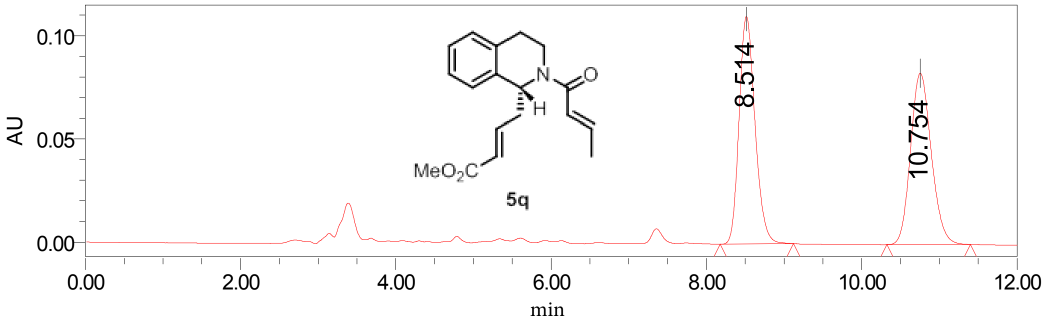

Peak Information:

	RetTime (min)	Area (μV*s)	Area (%)	Height (μV)
1	8.514	1572058.587	50.470	110278
2	10.754	1542759.384	49.530	83093

Sample Name: 5q-ch Wave Length: PDA 220nm
Column: OD-H

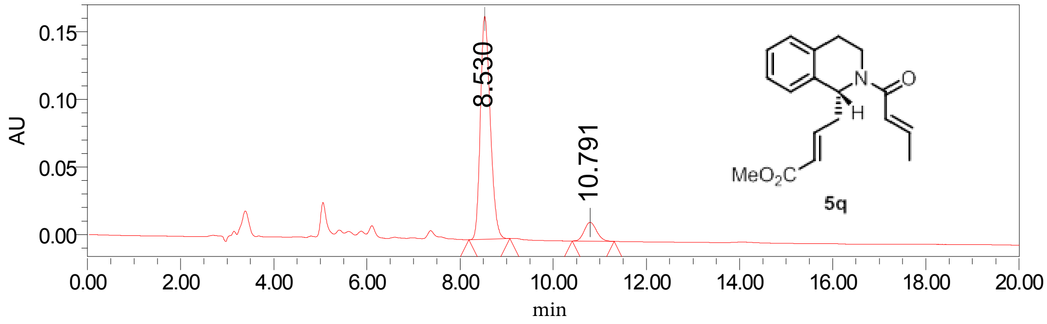

Peak Information:

	RetTime (min)	Area (μV*s)	Area (%)	Height (μV)
1	8.530	2424593.629	90.404	165043
2	10.791	257362.216	9.596	13925

Supplementary Figure 124. HPLC spectrum of **5q**

Sample Information

Sample Name: 5r-rac   Wave Length: PDA 240nm
Column: IA-3

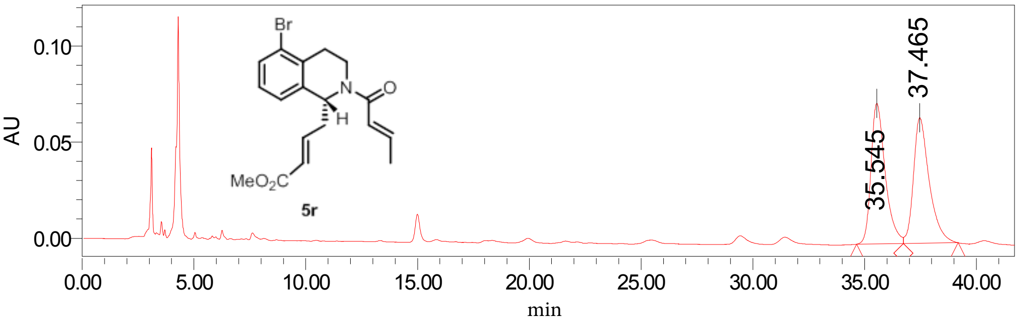

Peak Information:

	RetTime (min)	Area (µV*s)	Area (%)	Height (µV)
1	35.545	3239934.086	50.119	73141
2	37.465	3224582.328	49.881	65312

Sample Name: 5r-ch   Wave Length: PDA 240nm
Column: IA-3

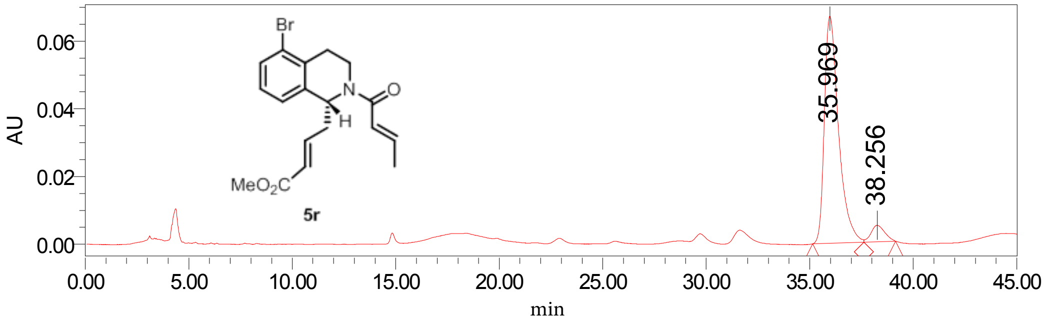

Peak Information:

	RetTime (min)	Area (µV*s)	Area (%)	Height (µV)
1	35.969	3181029.936	93.451	67134
2	38.256	222934.059	6.549	4859

Supplementary Figure 125. HPLC spectrum of **5r**

Sample Information

Sample Name: 7-rac
Column: IA-3
Wave Length: PDA 275nm

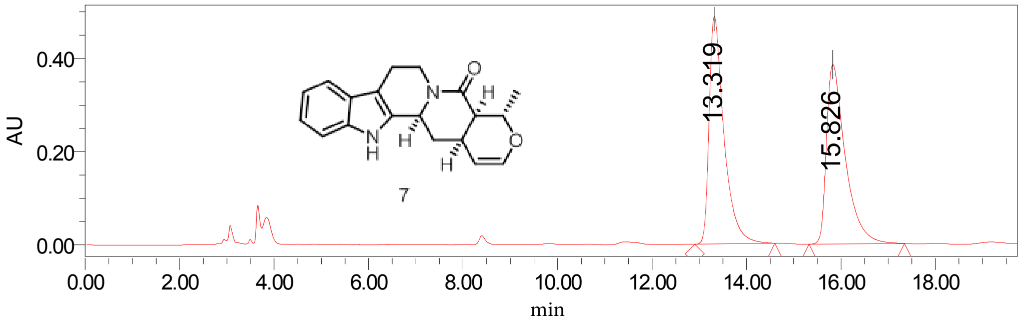

Peak Information:

	RetTime (min)	Area (μV*s)	Area (%)	Height (μV)
1	13.319	10602148.110	49.858	488230
2	15.826	10662605.694	50.142	385288

Sample Name: 7-ch
Column: IA-3
Wave Length: PDA 275nm

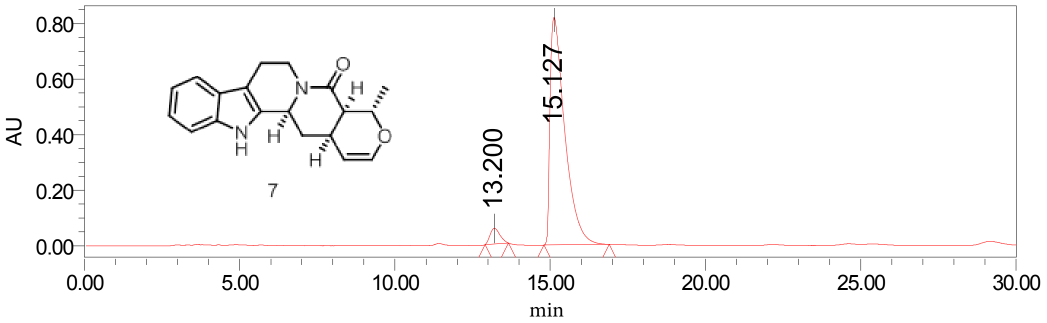

Peak Information:

	RetTime (min)	Area (μV*s)	Area (%)	Height (μV)
1	13.200	1258878.598	4.480	56226
2	15.127	26839939.260	95.520	820062

Supplementary Figure 126. HPLC spectrum of **7**

Sample Information

Sample Name: 8-rac Wave Length: PDA 260nm
Column: IF-3

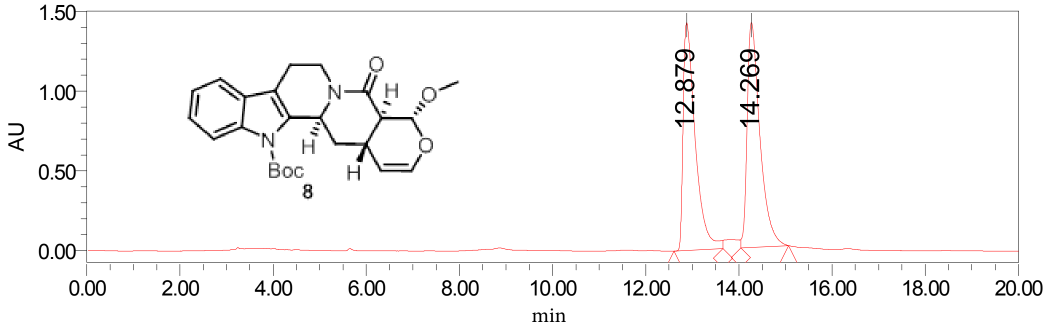

Peak Information:

	RetTime (min)	Area (μV*s)	Area (%)	Height (μV)
1	12.879	25982423.255	49.277	1430097
2	14.269	26745119.473	50.723	1410575

Sample Name: 8-ch Wave Length: PDA 260nm
Column: IF-3

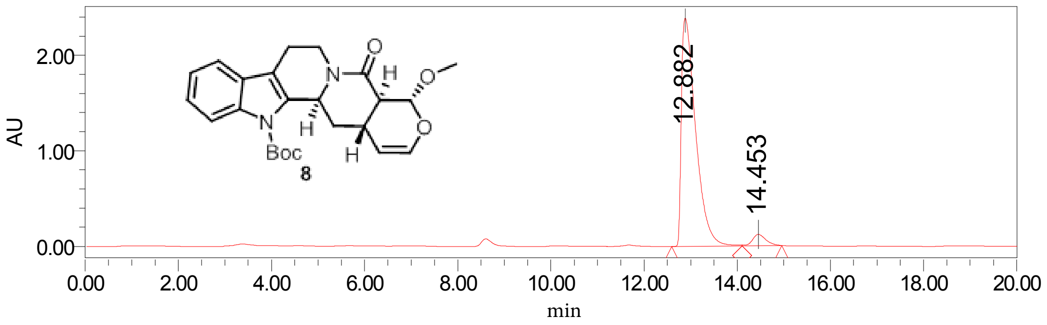

Peak Information:

	RetTime (min)	Area (μV*s)	Area (%)	Height (μV)
1	12.882	50688281.023	95.577	2389864
2	14.453	2345474.536	4.423	117757

Supplementary Figure 127. HPLC spectrum of **8**

Sample Information

After one recrystallization:

Sample Name: 8-rac
Column: IF-3

Wave Length: PDA 260nm

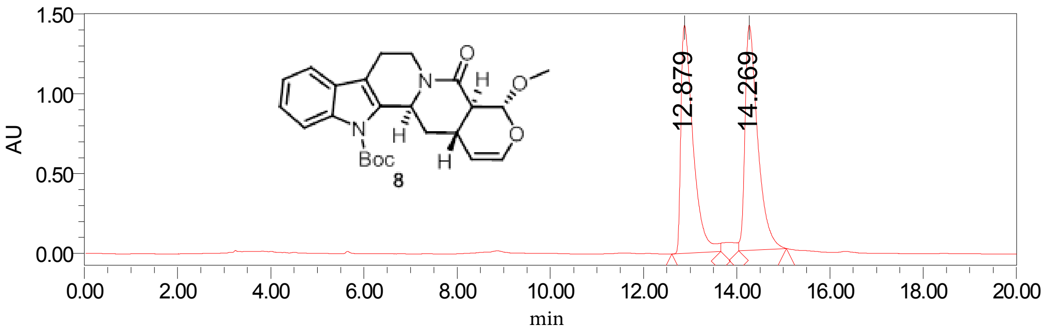

Peak Information:

	RetTime (min)	Area (μV*s)	Area (%)	Height (μV)
1	12.879	25982423.255	49.277	1430097
2	14.269	26745119.473	50.723	1410575

Sample Name: 8-ch
Column: IF-3

Wave Length: PDA 260nm

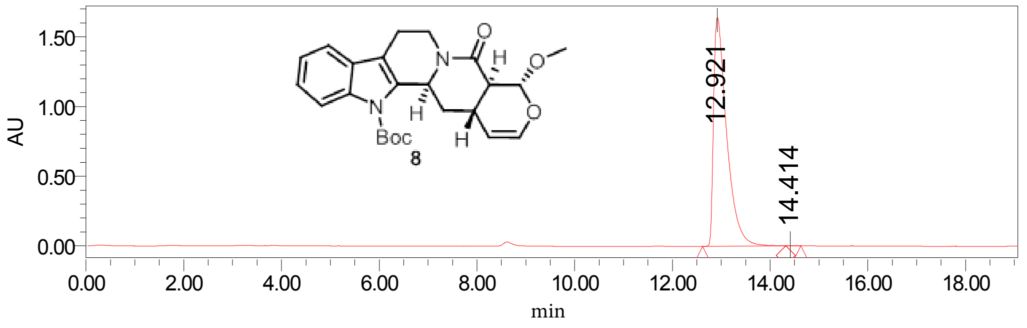

Peak Information:

	RetTime (min)	Area (μV*s)	Area (%)	Height (μV)
1	12.921	30741654.011	99.940	1642153
2	14.414	18457.576	0.060	1471

Supplementary Figure 128. HPLC spectrum of **8** after one recrystallization

Sample Information

Sample Name: Naucleofficine I ((±) -1a) Wave Length: PDA 245nm
Column: IA-3

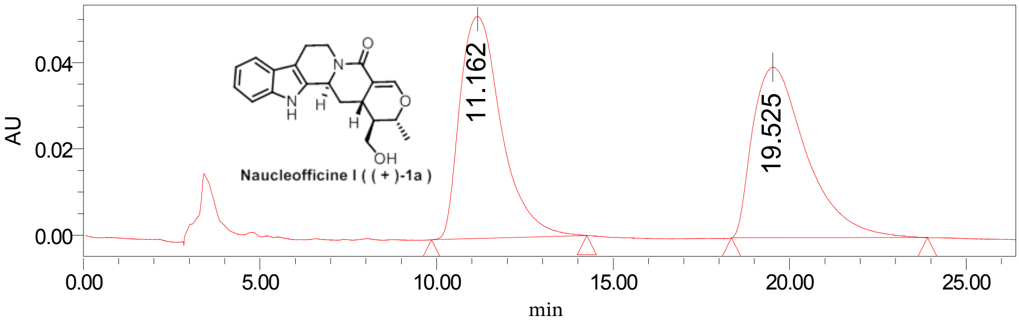

Peak Information:

	RetTime (min)	Area (μV*s)	Area (%)	Height (μV)
1	11.162	4219232.331	50.212	51456
2	19.525	4183549.634	49.788	39517

Sample Name: Naucleofficine I ((+)-1a) Wave Length: PDA 245nm
Column: IA-3

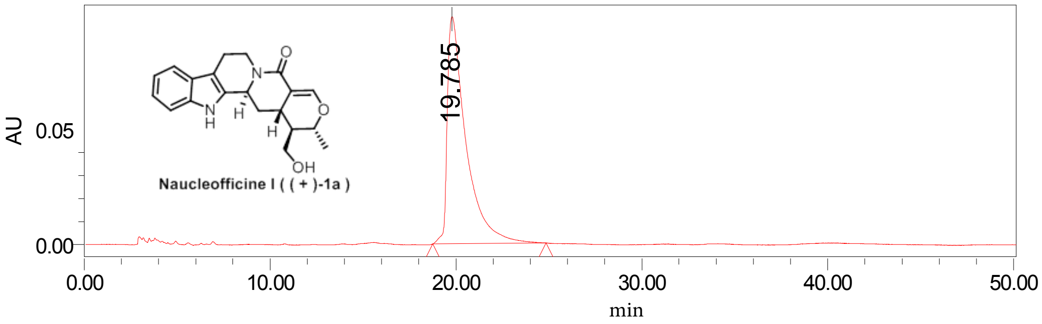

Peak Information:

	RetTime (min)	Area (μV*s)	Area (%)	Height (μV)
1	19.785	6905009.942	100.000	98821

Supplementary Figure 129. HPLC spectrum of **1a**

Sample Information

Sample Name: Naucleofficine II ((±)-1b) Wave Length: PDA 215nm
Column: IA-3

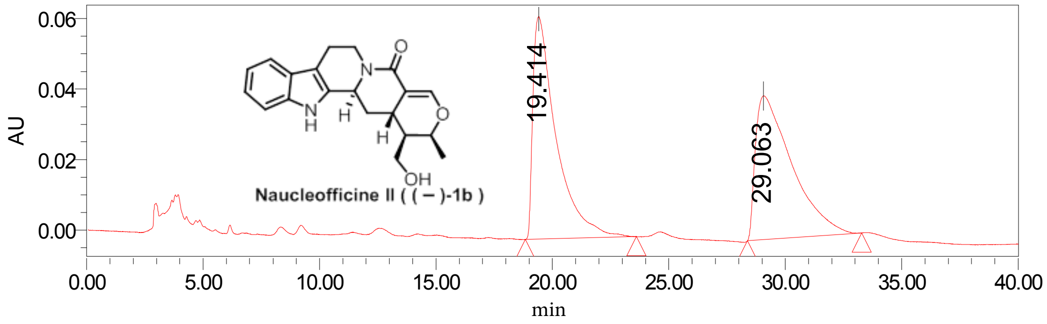

Peak Information:

	RetTime (min)	Area (μV*s)	Area (%)	Height (μV)
1	19.414	4383993.090	49.704	63125
2	29.063	4436244.092	50.296	40806

Sample Name: Naucleofficine II ((-)-1b) Wave Length: PDA 215nm
Column: IA-3

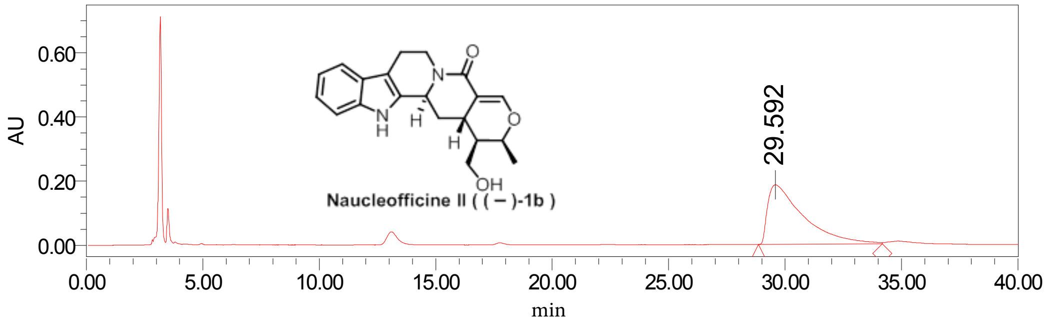

Peak Information:

	RetTime (min)	Area (μV*s)	Area (%)	Height (μV)
1	29.592	19888338.912	100.000	185494

Supplementary Figure 130 HPLC spectrum of **1b**

Supplementary References:

1. The compound **S1** was prepared using the same procedure as reference: Tian, J.-M., *et al.* The design of a spiro-pyrrolidine organocatalyst and its application to catalytic asymmetric Michael addition for the construction of all-carbon quaternary centers. *Chem. Commun.* **51**, 9979-9982 (2015).
2. Lalonde, M. P., McGowan, M. A., Rajapaksa, N. S. & Jacobsen, E. N. Enantioselective Formal Aza-Diels–Alder Reactions of Enones with Cyclic Imines Catalyzed by Primary Aminothioureas. *J. Am. Chem. Soc.* **135**, 1891-1894 (2013).
3. Langlois Andriamialisoa, R. Z.; Langlois, N. & Langlois, Y. A new efficient total synthesis of vindorosine and vindoline. *J. Org. Chem.* **50**, 961-967 (1985).
4. Pelletier, J. C. & Cava, M. P. Synthesis of the marine alkaloids aaptamine and demethyloxyaaptamine and of the parent structure didemethoxyaaptamine. *J. Org. Chem.*, **52**, 616-622 (1987).
5. Hameed, P. S., *et al.* Novel N-Linked Aminopiperidine-Based Gyrase Inhibitors with Improved hERG and in Vivo Efficacy against *Mycobacterium tuberculosis*. *Journal of medicinal chemistry*, **57**, 4889-4905 (2014).
6. Wang, H.-Y., Wang, R.-X., Zhao, Y.-X., Liu, K., Wang, F.-L., Sun, J.-Y. Three New Isomeric Indole Alkaloids from Nauclea officinalis. *Chem. Biodiversity.* **12**, 1256-1262 (2015).
